# One-Pot Synthesis of *N*-Iodo Sulfoximines from Sulfides

**DOI:** 10.1021/acs.joc.1c00292

**Published:** 2021-03-25

**Authors:** Anže Zupanc, Marjan Jereb

**Affiliations:** Faculty of Chemistry and Chemical Technology, University of Ljubljana, Večna pot 113, 1000 Ljubljana, Slovenia

## Abstract

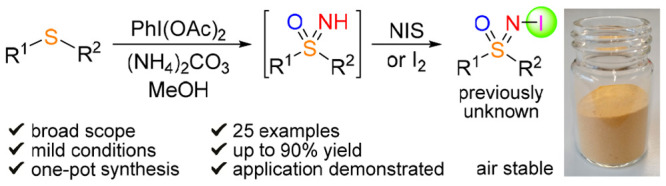

This is the first report on the synthesis and characterization of *N*-iodo sulfoximines. The synthesis was designed as a room temperature one-pot cascade reaction from readily available sulfides as starting compounds, converted into sulfoximines by reaction with ammonium carbonate and (diacetoxyiodo)benzene, followed by iodination with *N*-iodosuccinimide or iodine *in situ*, in up to 90% isolated yields, also at a multigram scale. Iodination of aryls with *N*-iodo sulfoximines, oxidation, and conversion to *N*-SCF_3_ congeners have been demonstrated.

The increasing interest in sulfoximines^1−9^ in drug discovery,^10−14^ medicine,^15−18^ and agrochemistry^19,20^ has led to a rapidly growing number of publications^21−34^ in these and other research areas.^35^ These compounds are of great importance as ligands, auxiliaries, and catalysts, e.g., in asymmetric synthesis and catalysis.^36,37^ Recently, a large number of reports on their synthesis and transformations have appeared in the literature,^38−41^ including halo- and chalcogenations. Sulfoximines undergo chlorinations with NCS (Figure 1a),^42^ brominations with NBS (Figure 1b),^43^ trifluoromethylthiolations with AgSCF_3_ (Figure 1c),^43^ trifluoromethylations (Figure 1d),^44−48^ halocyclizations with (diacetoxyiodo)benzene (DIB)/KI (Figure 1e),^49^ reactions with (DIB)/I_2_ under visible light (Figure 1f),^50^ chlorinations and *N*-sulfonylations in the presence of I_2_/H_2_O_2_ (Figure 1g),^51^ for example.

**Figure 1:**
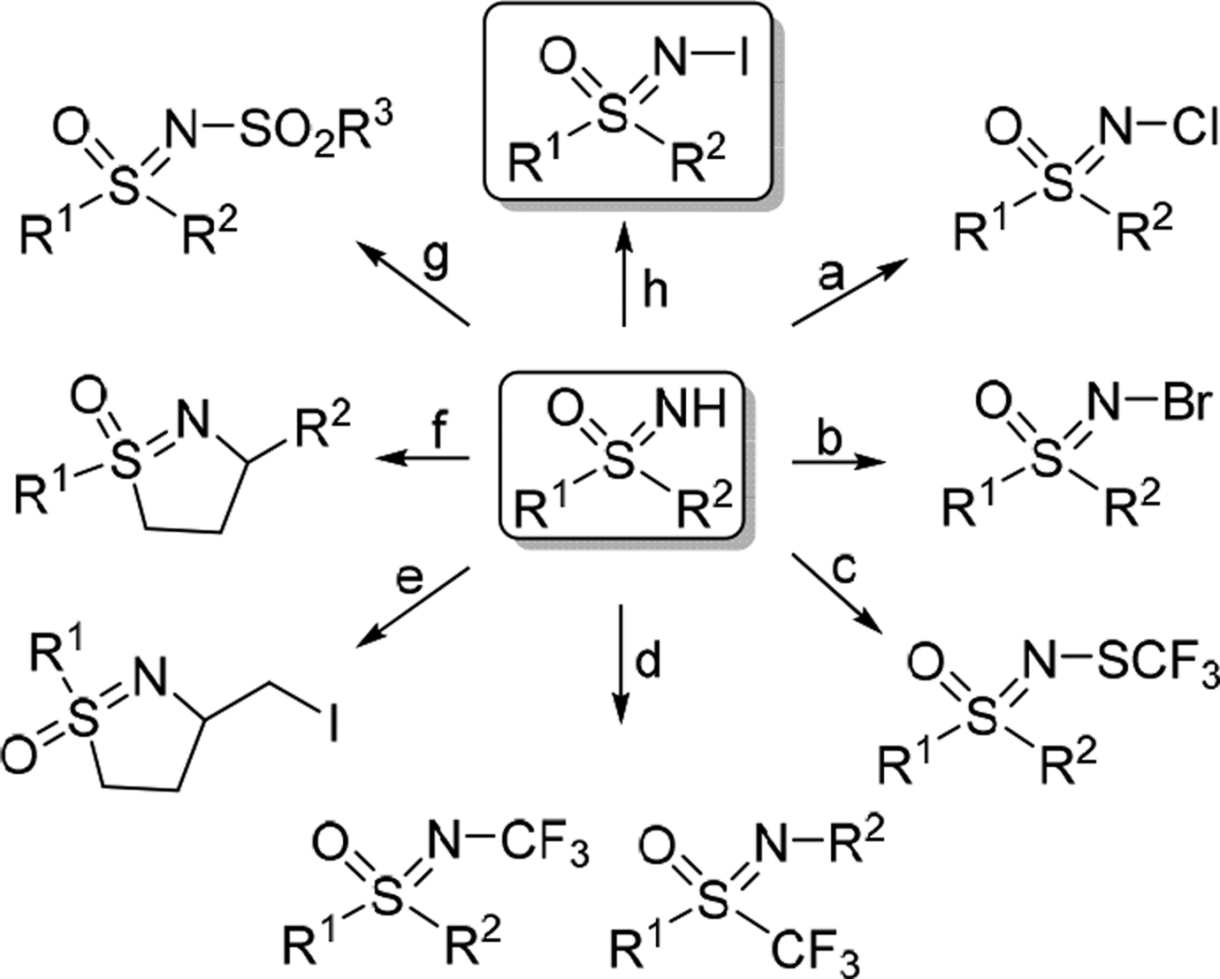
(a–g) Transformations of sulfoximines with halo- and chalcogenating agents, (h) this work.

Surprisingly, unlike the halogen and chalcogenide derivatives, the *N*-iodo sulfoximines remain elusive. These compounds have been proposed as reactive intermediates in the iodine-mediated Hofmann–Löffler–Freytag reaction of sulfoximines, leading to dihydroisothiazole oxides (Figure 1f).^50^ Five decades ago, two *N*-iodo sulfoximines were mentioned in the patent literature, but without any characterization data.^52^ All subsequent attempts to prepare these compounds were unsuccessful.^43,51^ Our experience with halogenations of organic compounds and green chemistry^53−57^ prompted us to develop a reliable method for the synthesis of *N*-iodo sulfoximines. With simplicity and sustainability in mind, we developed a one-pot cascade protocol using sulfides as starting materials. Selected transformations were also demonstrated.

In initial screening experiments, PhSONHMe was reacted with I_2_/K_2_CO_3_, giving supposedly **2a** with 95% conversion in MeCN and DCM. High conversion stimulated us to develop the one-pot protocol from sulfides. Thioanisole (**1a**, 1 mmol) was allowed to react with (NH_4_)_2_CO_3_ (1.5 equiv) and (diacetoxyiodo)benzene (DIB, 2.3 equiv) in MeOH (10 mL) to give PhSONHMe. Then the reaction solvent was replaced by DCM (10 mL), followed by the addition of *N*-iodosuccinimide (NIS, 1.2 equiv) for iodination. Although complete conversion to a product tentatively identified as **2a** by ^1^H NMR (see below) was observed after 16 h of stirring at room temperature, attempts to isolate the product failed. Repeating both steps in MeOH (10 mL) as the sole reaction solvent also resulted in complete conversion to the same product, but with the same failure to isolate as described above (Table 1, entry 1). Shortening the reaction time for the iodination step proved to be beneficial. After 2 h, the precipitate formed in the reaction mixture was collected by filtration, and NMR analysis showed the desired product **2a** in 57% yield, accompanied by unreacted NIS and succinimide (Table 1, entry 2).

**Table 1:**
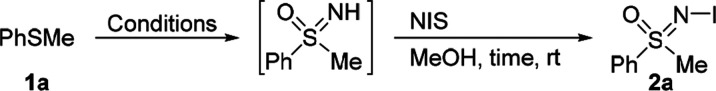
Optimization of the Reaction Conditions Using NIS^a^

entry	MeOH (mL)	time (h)	yield (%)^b^
1	10	16	
2	10	2	57^c^
3	10	0.33	67^c^
4	5	0.33	74
5	3	0.33	68^c^
6	2	0.33	78^c^
7	2	1	80^c^
8	3	1	80^c^
9	5	1	74

a Conditions: **1a** (1.0 mmol), (NH_4_)_2_CO_3_ (1.5 mmol), DIB (2.3 mmol), MeOH (mL), 1 h, rt, then NIS (1.1 equiv), time, rt.

b Yield.

c NIS/succinimide accompanied the product in 3%/4% (entry 2), 9%/7% (entry 3), 9%/9% (entry 5), 12%/12% (entry 6), 0%/6% along with unidentified side products (entry 7), 0%/5% along with unidentified side products (entry 8).

Further reduction of the reaction time to 0.33 h yielded 67% of impure **2a** (Table 1, entry 3). Finally, halving the volume of MeOH from 10 to 5 mL at this point allowed the isolation of pure **2a** in good 74% yield (Table 1, entry 4). Further reduction in the volume of reaction solvent did not prove beneficial (Table 1, entries 5–8). It is noteworthy that the success of crystallization of **2a** from the reaction mixture depends strongly on the surface area of the reaction vessel, as can be concluded from several successive repeating of the above experiments. The best yields of the isolated product were obtained when the reaction was carried out in a worn (scratched) glass round-bottom flask, in a polyethylene vessel, or in the presence of a glass frit that aided the nucleation process (*vide infra*).

Product **2a** showed a characteristic singlet resonance (δ = 3.33 ppm) for the S-CH_3_ group in ^1^H NMR spectra (CDCl_3_), which was deshielded as compared to both thioanisole (**1a**, δ = 2.44 ppm) and PhSONHMe (δ = 3.12 ppm). HRMS analysis in positive ESI+ mode confirmed the ion formula of C_7_H_9_INOS^+^ for [M + H]^+^ (*m*/*z* calcd 281.9445, found for [M + H]^+^ 281.9431). The molecular formula was corroborated by CHN elemental analysis (calcd for C_7_H_8_INOS: C, 29.91; H, 2.87; N, 4.98. Found: C, 30.00; H, 2.70; N, 4.81.

Having identified the optimal reaction conditions, we focused on screening the substrate scope (Scheme 1). Mixed phenyl alkyl sulfides **1b**–**1d** gave the corresponding *N*-iodo sulfoximines **2b**–**2d** regardless of alkyl chain length or branching. Electron-rich and electron-poor aryl alkyl sulfides gave the desired products **2e**–**2o** in good to high yields. Phenyl alkyl and pentafluorophenyl alkyl sulfides **1p**–**1s** reacted smoothly and gave the expected products **2p**–**2s** in up to 90% yields. The C_6_F_5_ motif in product **2r** is notable for its intrinsic properties that allow molecular recognition and improved structural ordering.^58,59^ Relatively challenging substrates, diphenyl sulfide **1t** and dibenzothiophene **1u**, afforded the corresponding products **2t** and **2u** in reasonable yields. Benzyl methyl-, symmetric and unsymmetric dialkyl sulfides **1v**–**1y** afforded products **2v**–**2y** in moderate yields. The method was also suitable for heteroaromatic sulfides, as shown by the formation of the product **2z** formation in 66% yield. In some cases, those of **2d**, **2e**, **2g**, **2j**–**2s**, **2v**, **2x**, and **2y**, the product did not precipitate from the reaction mixture as described above for **2a**. Since attempts to precipitate pure products by addition of a co-solvent (EtOAc, Et_2_O, DCM, AcOH, PE, or hexanes) failed, MeOH was evaporated and the residue was subjected to rapid flash chromatography through a short silica gel plug with DCM as eluent. Prolonged contact with SiO_2_, Al_2_O_3_ (neutral or basic), activated carbon, or extractive workup was detrimental to the *N*-iodo sulfoximines.

**Scheme 1:**
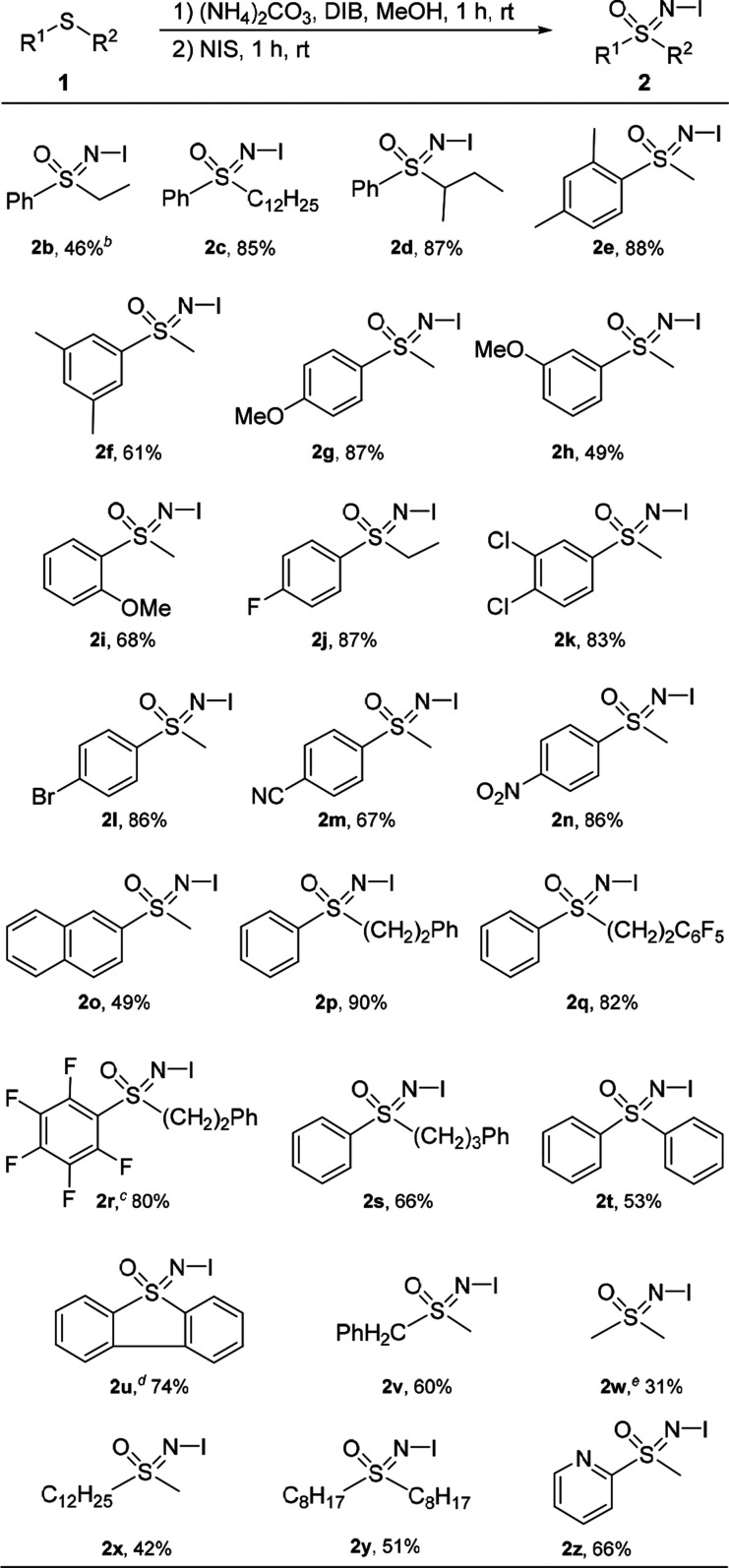
Substrate Scope Reaction conditions: (1) **1** (1 mmol), (NH_4_)_2_CO_3_ (1.5 equiv), DIB (2.3 equiv), MeOH (5 mL), then (2) NIS (1.1 equiv). Yield. (NH_4_)_2_CO_3_ (2.625 equiv) and DIB (4.025 equiv) were used. (NH_4_)_2_CO_3_ (1.875 equiv) and DIB (2.875 equiv) were used. From DMSO as starting compound, with (NH_4_)_2_CO_3_ (1.5 equiv), DIB (1.3 equiv) and I_2_ (1.1 equiv) in MeOH (2 mL).

The scalability of the protocol was tested using sulfide **1a** as a model substrate. A mixture of **1a** (10 mmol), (NH_4_)_2_CO_3_ (15 mmol), and DIB (23 mmol) in MeOH (50 mL) was stirred in a used polyethylene flask for 1 h at room temperature. After addition of NIS (11 mmol) and further stirring of the reaction mixture for 1 h, the precipitate was collected by filtration to afford pure **2a** in 78% yield. Repeating the above procedure in a new round-bottom glass flask gave **2a** in only a modest 44% yield, consistently indicating the importance of the surface area of the reaction vessel for nucleation.

In addition to NIS, I_2_ was tested as an iodinating agent. Under the same reaction conditions as in Scheme 1, sulfide **1a** was reacted *in situ* to give PhSONHMe and then treated with I_2_ to give **2a**. The iodination proceeded smoothly without the need for a catalyst or promoter. The optimization process of the reaction conditions is summarized in Table 2.

**Table 2:**
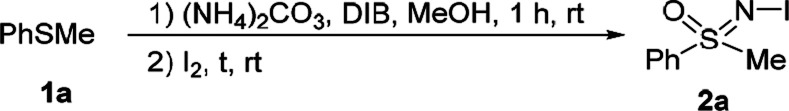
Optimization and Scale-Up by Using I_2_^a^

entry	MeOH (mL)	I_2_ (equiv)	time (h)	yield (%)^b^
1	5	1.1	1	44
2	5	1.1	16	52
3	1	1.1	1	62^c^
4	2	1.5	1	79
5	2	1.1	1	75
6	2	1.25	1	77
7^d^	20	1.1	1	74
8^e^	50	1.1	1	75

a Reaction conditions: (1) **1a** (1 mmol), (NH_4_)_2_CO_3_ (1.5 mmol), DIB (2.3 mmol), MeOH (mL), 1 h, rt, then (2) I_2_ (equiv), time, rt, then filtration of precipitate, washing with a small amount of MeOH and an excess of *n*-hexane.

b Yield.

c Impure product.

d The reaction was conducted with 10 mmol of **1a**.

e The reaction was conducted with 25 mmol of **1a**.

Reaction of the sulfoximines generated *in situ* from **1a** with I_2_ (1.1 equiv) in MeOH (5 mL) gave **2a** in moderate yield (Table 2, entries 1 and 2). Reducing the amount of reaction solvent to 1 mL gave impure **2a** (Table 2, entry 3). Optimal results were obtained with 2 mL of MeOH and 1.1–1.25 equiv of I_2_ (Table 2, entries 5 and 6). On a larger scale, experiments using I_2_ as the iodinating agent were performed with 10 and 25 mmol amounts of **1a** to give pure **2a** in consistent 74% (2.09 g) and 75% (5.31 g) yields, respectively (Table 2, entries 7 and 8).

Having in hand the one-pot protocol for the *N*-iodination of sulfoximines formed *in situ*, we decided to briefly extend it to the preparation of *N*-bromo sulfoximine **3** and *N*-chloro sulfoximine **4** (Scheme 2). The reactions were carried out with **1a** (1 mmol) in MeOH (5 mL) at room temperature with variable amounts of *N*-bromosuccinimide (NBS) and *N*-chlorosuccinimide (NCS). The yields of products **3** and **4** depended strongly on the amount of halogenating agent.

**Scheme 2:**
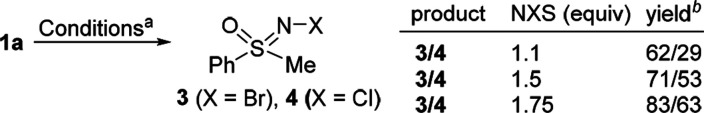
*N*-Bromo and *N*-Chloro Sulfoximines Reaction conditions: (1) **1a** (1 mmol), MeOH (5 mL), (NH_4_)_2_CO_3_ (1.5 mmol), DIB (2.3 mmol), then (2) NBS or NCS (equiv), rt. Yield (%).

Finally, to demonstrate the applicability of the *N*-iodo sulfoximines, we decided to test their potential in the electrophilic aromatic substitution reaction with activated benzene derivatives (Table 3). Treatment of **5** with **2a** in AcOH for 1.5 h at 22 °C resulted in full conversion to 4-iodoanisole (**6**, entry 1). At elevated temperature (60 °C), product **6** formed in 1.5 h and was isolated in 86% yield (entry 2). No reaction was observed in DCM, MeOH, or MeCN as reaction solvent, with unconsumed **5** being regenerated (entries 3–5). This is in sharp contrast to NIS, which proved to work best in MeCN^60^ and TFA,^61^ suggesting potential orthogonality of these two reagents in iodinations. Phenol (**7**) was triiodinated with **2a** at room temperature to give **8** (entry 6), while 1-methoxynaphthalene (**9**) gave the 4-iodo derivative **10** (entry 7). Iodination also proceeded with less activated 4-nitrophenol (**11**) to give the diiodinated derivative **12** in excellent yield (entry 8). Thiophenol (**13**), on the other hand, gave diphenyl disulfide quantitatively (**14**, entry 9). After completion of the reactions from Table 3, the resulting PhSONHMe was simply removed by extractive workup (HCl_*aq*_/DCM), yielding a pure product that required no further purification.

**Table 3:**
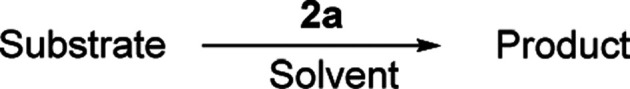

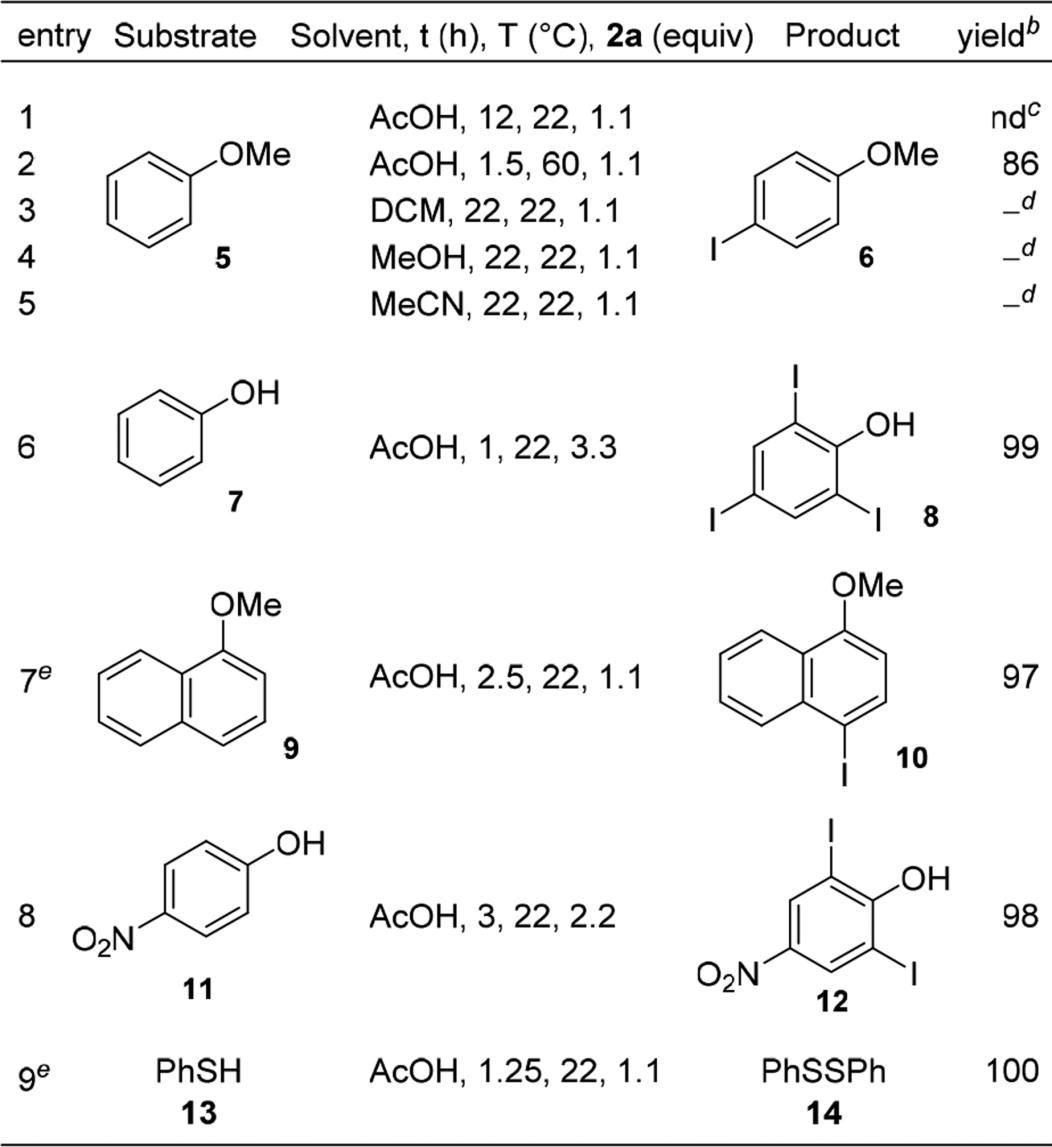
Iodination and Oxidation with **2a**^a^

a Reaction conditions: **5**–**13** (1 mmol), AcOH (5 mL), **2a** (equiv).

b Yield.

c Full conversion according to ^1^H NMR analysis; the product was not isolated.

d Starting **5** remined unconsumed.

e Slow addition of **2a** and additional stirring: entry 7, 2 h and 0.5 h; entry 9, 1 h and 0.25 h.

We also tested the reactivity of *N*-iodo sulfoximines toward silver(I) trifluoromethanethiolate (AgSCF_3_). The desired *N*-SCF_3_-substituted sulfoximines **15** were obtained in good to excellent yields (Table 4). This is complementary to the procedure of Bohnen and Bolm,^43^ who developed the synthesis of *N*-trifluoromethylthiolated sulfoximines from sulfoximines via the corresponding *N*–Br derivatives, and will add to the chemistry of this specific type of compounds.^43^

**Table 4:**
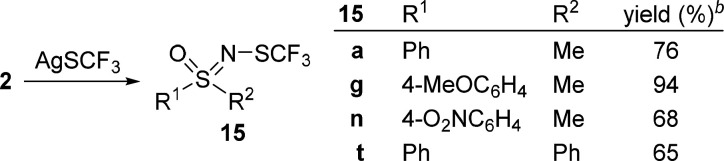
Synthesis of *N*-SCF_3_-Substituted Sulfoximines^a^

a Reaction conditions: **2** (0.3–1 mmol), AgSCF_3_ (1.2 equiv), Ar, MeCN (5 mL/1 mmol of **2**), 0.33–1 h, rt.

b Yield.

In summary, we have developed a one-pot telescoped synthesis of *N*-iodo sulfoximines from sulfides using NIS or I_2_. The reaction proceeds via sulfoximines as reaction intermediates. The protocol is simple and suitable for obtaining a series of structurally diverse (hetero)aryl-, alkyl-, and benzyl-substituted products that can be easily isolated as stable compounds in pure form and in high yields. A multigram scale synthesis was also demonstrated. The reactivity of *N*-iodo sulfoximines was preliminarily investigated, revealing interesting properties as iodinating and oxidizing agents. For example, unlike NIS, which performs best in MeCN, no iodination of activated anisole occurs with **2a**. In contrast, iodination in acetic acid is readily possible, even with 4-nitrophenol as an example of a deactivated substrate. *N*-Iodo sulfoximines were also found to be valuable intermediates in the synthesis of *N*-SCF_3_-substituted sulfoximines. An in-depth study of the chemistry of *N*-iodo sulfoximines is underway.

## Experimental Section

### General Considerations

Chemicals and solvents were obtained from commercial sources. TLC was performed on Merck-60-F_254_ plates using mixtures of petroleum ether (PE), hexane, dichloromethane (DCM), diethyl ether, ethyl acetate, and methanol. For flash chromatography, silica gel (63–200 μm, 70–230 mesh ASTM; Fluka) was used. The glass frit MPLC Büchi was utilized for induction of nucleation of the products. Products were characterized by ^1^H, ^13^C, and ^19^F NMR spectroscopy, IR spectroscopy, HRMS, and melting points of solids. All NMR spectra were recorded in CDCl_3_ using Me_4_Si as an internal standard. Chemical shifts are reported in δ (ppm) values relative to δ = 0.00 ppm (Me_4_Si) for ^1^H NMR, and to the central line of CDCl_3_ (δ = 77.16 ppm) for ^13^C NMR. ^19^F spectra were referenced to CFCl_3_ as an external standard at δ = 0.00 ppm. ^1^H, ^13^C, and ^19^F NMR spectra were recorded with a Bruker Avance III 500 instrument at 500, 126, and 471 MHz, respectively. IR spectra were recorded with a Bruker FTIR Alpha Platinum spectrophotometer. LC-HRMS analyses were performed on a Shimadzu LCMS-IT-TOF system (Kyoto, Japan), composed of a liquid chromatograph Nexera XR hyphenated to a mass spectrometer with an ion trap and time-of-flight tube equipped with an electrospray ionization (ESI) source. The melting points were determined with an OptiMelt MPA100. By heating, all *N*-iodo sulfoximines first changed color from orange or yellow to brown and then melted into brown oily liquids. A change of color could imply partial degradation. Elemental combustion analyses were performed with a PerkinElmer analyzer 2400 CHN.

### Synthesis of *N*-Iodo Sulfoximines

A mixture of organic sulfide (**1a**–**q**, **1s**, **1t**, **1v**, **1x**–**z**, 1 mmol), 5 mL of MeOH, 1.5 equiv of (NH_4_)_2_CO_3_ (1.5 mmol, 144 mg), and 2.3 equiv of PhI(OAc)_2_ (DIB, 2.3 mmol, 741 mg) was charged into a 10 mL round-bottom flask equipped with a magnetic stirrer. The flask was sealed with a glass stopper, and the reaction mixture was let to stir vigorously for 1 h.

In the case of **1r** (1 mmol), 2.625 equiv of (NH_4_)_2_CO_3_ (2.625 mmol, 252 mg) and 4.025 equiv of DIB (4.025 mmol, 1296 mg) were used.

In the case of **1u** (1 mmol), 1.875 equiv of (NH_4_)_2_CO_3_ (1.875 mmol, 180 mg) and 2.875 equiv of DIB (2.875 mmol, 926 mg) were used.

Then, 1.1 equiv of NIS (1.1 mmol, 248 mg) was added, and the stirring was continued for another hour.

The method of isolation was chosen depending on whether the product **2** precipitated or not.

Method A for products **2** that precipitated from the reaction mixture (**2a**–**2c**, **2f**, **2h**, **2i**, **2t**, **2u**, **2z**).

The precipitate was collected by vacuum filtration using a Büchner funnel, washed with a small amount of MeOH, and dried under reduced pressure (vacuum pump) to obtain pure product **2**.

Method B for products **2**, not precipitating from the reaction mixture (**2d**, **2e**, **2g**, **2j**–**2s**, **2v**, **2x**, **2y**).

The reaction solvent was removed under reduced pressure; the residue was redissolved in small amounts of DCM and subjected to flash chromatography under pressure (nitrogen gas) through a short plug of SiO_2_ as a stationary phase and DCM as eluant. The elution was performed in less than 3 min to avoid decomposition of the product. The progress of separation was monitored visually and, if necessary, by TLC analysis. Pink-colored fractions that eluted first contained I_2_ and PhI and were disposed of. Orange-colored fractions containing product **2** were collected into 50–100 mL flasks, and the solvent was removed under reduced pressure to obtain a brown-orange semisolid. It was redissolved in small amounts of DCM, triturated with large amounts of PE (or hexane) to induce solidification (for solid products), and evaporated to dryness under reduced pressure. The process was repeated 2–3 times to remove any residual I_2_ or PhI, resulting in pure product **2**.

#### Synthesis of **2w**

A mixture of DMSO (78 mg, 1 mmol), 2 mL of MeOH, 1.5 equiv of (NH_4_)_2_CO_3_ (1.5 mmol, 144 mg), and 1.3 equiv of DIB (1.3 mmol, 419 mg) were charged into a 10 mL round-bottom flask equipped with a magnetic stirrer. The flask was closed with a glass stopper, and the reaction mixture was let to stir vigorously for 1 h. Then, 1.1 equiv of I_2_ (1.1 mmol, 279 mg) was added, and stirring was continued for another hour. The reaction mixture was cooled to −5 °C (using an ice/NaCl cooling bath); the precipitate was collected by vacuum filtration using a Büchner funnel and washed with small amounts of cold (−5 °C) MeOH, followed by large amounts of hexane. The product was dried under reduced pressure (vacuum pump) to obtain an orange-brown solid of **2w** (68 mg, 0.31 mmol, 31%).

### Synthesis of **2a** Using NIS (10 mmol Scale)

A mixture of methyl phenyl sulfide (**1a**, 10 mmol, 1242 mg), 50 mL of MeOH, 1.5 equiv of (NH_4_)_2_CO_3_ (15 mmol, 1440 mg), and 2.3 equiv of DIB (23 mmol, 7410 mg) was charged into a 250 mL polyethylene container equipped with a large magnetic stirrer. The container was closed with a plastic screw top, and the reaction mixture was let to stir vigorously for 1 h. Then, 1.1 equiv of NIS (11 mmol, 2480 mg) was added, and the stirring was continued for another hour. The precipitate was collected by vacuum filtration using a Büchner funnel, was washed with small amounts of MeOH, and dried under reduced pressure (vacuum pump) to obtain a pale yellow solid of **2a** (2182 mg, 7.8 mmol, 78%).

Repeating the synthesis under the same reaction conditions in a flawless 250 mL round-bottom flask, equipped with a magnetic stirrer, afforded **2a** in 44% yield (1244 mg, 4.4 mmol).

### Synthesis of **2a** Using I_2_ (10 mmol Scale)

A mixture of methyl phenyl sulfide (**1a**, 10 mmol, 1242 mg), 20 mL of MeOH, 1.5 equiv of (NH_4_)_2_CO_3_ (15 mmol, 1440 mg), and 2.3 equiv of DIB (23 mmol, 7410 mg) was charged into a 250 mL polyethylene container equipped with a large magnetic stirrer. The container was sealed with a plastic screw top, and the reaction mixture was let to stir vigorously for 1 h. Then, 1.1 equiv of I_2_ (11 mmol, 2794 mg) was added, and the stirring was continued for another hour. The precipitate was collected by vacuum filtration using a Büchner funnel, was washed with small amounts of MeOH and large amounts of hexane, and dried under reduced pressure (vacuum pump) to obtain **2a** (2090 mg, 7.4 mmol, 74%) as an orange solid.

### Synthesis of **2a** Using I_2_ (25 mmol Scale)

A mixture of methyl phenyl sulfide (**1a**, 25 mmol, 3105 mg), 50 mL of MeOH, 1.5 equiv of (NH_4_)_2_CO_3_ (37.5 mmol, 3603 mg), and 2.3 equiv of DIB (57.5 mmol, 18521 mg) was charged into a 250 mL polyethylene container equipped with a large magnetic stirrer. The container was sealed with a plastic screw top, and the reaction mixture was let to stir vigorously for 1 h. Then, 1.1 equiv of I_2_ (27.5 mmol, 6980 mg) was added, and the stirring was continued for another hour. The precipitate was collected by vacuum filtration using a Büchner funnel, was washed with small amounts of MeOH and large amounts of hexane, and dried under reduced pressure (vacuum pump) to obtain **2a** (5305 mg, 18.75 mmol, 75%) as an orange solid.

#### *N*-Iodo-*S*-methyl-*S*-phenyl Sulfoximine (**2a**)

**1a** (1 mmol, 124 mg), 1.5 equiv of (NH_4_)_2_CO_3_ (1.5 mmol, 144 mg), 2.3 equiv of DIB (2.3 mmol, 741 mg), 1.1 equiv of NIS (1.1 mmol, 248 mg), isolation method A: yellow solid (208 mg, 74%). ^1^H NMR (500 MHz, CDCl_3_): δ 7.82–7.89 (m, 2H), 7.66–7.71 (m, 1H), 7.57–7.64 (m, 2H), 3.33 (s, 3H). ^13^C{^1^H} NMR (126 MHz, CDCl_3_): δ 140.0, 133.8, 129.8, 128.5, 42.9. IR (neat): 3019, 2917, 1445, 1198, 1088, 971, 949, 774, 738, 685 cm^–1^. HRMS (ESI-TOF) *m*/*z*: [M + H]^+^ Calcd for C_7_H_8_INOS 281.9445; Found 281.9431. Mp = 125.3–125.6 °C. CHN analysis: Calcd for C_7_H_8_INOS: C, 29.91; H, 2.87; N, 4.98. Found: C, 30.00; H, 2.70; N, 4.81.

#### *N*-Iodo-*S*-ethyl-*S*-phenyl Sulfoximine (**2b**)

**1b** (1 mmol, 138 mg), 1.5 equiv of (NH_4_)_2_CO_3_ (1.5 mmol, 144 mg), 2.3 equiv of DIB (2.3 mmol, 741 mg), 1.1 equiv of NIS (1.1 mmol, 248 mg), isolation method A: yellow solid (136 mg, 46%). ^1^H NMR (500 MHz, CDCl_3_): δ 7.76–7.84 (m, 2H), 7.65–7.70 (m, 1H), 7.57–7.64 (m, 2H), 3.36–3.53 (m, 2H), 1.25–1.30 (m, 3H). ^13^C{^1^H} NMR (126 MHz, CDCl_3_): δ 138.1, 133.7, 129.6, 129.1, 49.6, 8.7. IR (neat): 2993, 2958, 1676, 1441, 1409, 1372, 1277, 1231, 1203, 1171, 1088, 1041, 956, 766, 719, 687, 674 cm^–1^. HRMS (ESI-TOF) *m*/*z*: [M + H]^+^ Calcd for C_8_H_10_INOS 295.9601; Found 295.9598. Mp = 118.2–118.6 °C.

#### *N*-Iodo-*S*-(1-dodecyl)-*S*-phenyl Sulfoximine (**2c**)

**1c** (1 mmol, 278 mg), 1.5 equiv of (NH_4_)_2_CO_3_ (1.5 mmol, 144 mg), 2.3 equiv of DIB (2.3 mmol, 741 mg), 1.1 equiv of NIS (1.1 mmol, 248 mg), isolation method A: pale yellow solid (369 mg, 85%). ^1^H NMR (500 MHz, CDCl_3_): δ 7.77–7.83 (m, 2H), 7.65–7.70 (m, 1H), 7.57–7.64 (m, 2H), 3.44 (ddd, *J* = 14.0, 11.4, 5.1 Hz, 1H), 3.33 (ddd, *J* = 14.0, 11.3, 5.0 Hz, 1H), 1.74–1.85 (m, 1H), 1.57–1.68 (m, 1H), 1.15–1.35 (m, 18H), 0.88 (t, *J* = 6.9 Hz, 3H). ^13^C{^1^H} NMR (126 MHz, CDCl_3_): δ 138.9, 133.6, 129.6, 129.0, 55.2, 31.9, 29.6, 29.6, 29.5, 29.4, 29.3, 29.0, 28.1, 23.8, 22.7, 14.2. IR (neat): 2911, 2849, 1472, 1444, 1220, 1206, 1175, 1092, 1011, 967, 756, 749, 713, 686 cm^–1^. HRMS (ESI-TOF) *m*/*z*: [M + H]^+^ Calcd for C_18_H_30_INOS 436.1166; Found 436.1171. Mp = 93.1–93.5 °C.

#### *N*-Iodo-*S*-(2-butyl)-*S*-phenyl Sulfoximine (**2d**)

**1d** (1 mmol, 166 mg), 1.5 equiv of (NH_4_)_2_CO_3_ (1.5 mmol, 144 mg), 2.3 equiv of DIB (2.3 mmol, 741 mg), 1.1 equiv of NIS (1.1 mmol, 248 mg), isolation method B: yellow solid (282 mg, 87%), a mixture of diastereoisomers. ^1^H NMR (500 MHz, CDCl_3_): δ 7.72–7.81 (m, 4H), 7.64–7.70 (m, 2H), 7.55–7.63 (m, 4H), 3.28–3.41 (m, 2H), 2.19–2.29 (m, 1H), 1.92–2.02 (m, 1H), 1.38–1.53 (m, 2H), 1.37 (d, *J* = 6.9 Hz, 3H), 1.23 (d, *J* = 6.9 Hz, 3H), 0.98 (t, *J* = 7.5 Hz, 3H), 0.92 (t, *J* = 7.5 Hz, 3H). ^13^C{^1^H} NMR (126 MHz, CDCl_3_): δ 137.4, 137.3, 133.6, 133.6, 130.0, 130.0, 129.5 (2C), 62.2, 62.0, 24.4, 23.3, 14.4, 13.3, 11.3, 11.3. IR (neat): 2972, 2934, 1442, 1192, 1086, 969, 789, 761, 731 cm^–1^. HRMS (ESI-TOF) *m*/*z*: [M + H]^+^ Calcd for C_10_H_14_INOS 323.9914; Found 323.9917. Mp = 90.2–91.5 °C.

#### *N*-Iodo-*S*-(2,4-dimethylphenyl)-*S*-methyl Sulfoximine (**2e**)

**1e** (1 mmol, 152 mg), 1.5 equiv of (NH_4_)_2_CO_3_ (1.5 mmol, 144 mg), 2.3 equiv of DIB (2.3 mmol, 741 mg), 1.1 equiv of NIS (1.1 mmol, 248 mg), isolation method B: yellow solid (272 mg, 88%). ^1^H NMR (500 MHz, CDCl_3_): δ 7.86 (d, *J* = 8.2 Hz, 1H), 7.21 (dd, *J* = 8.2, 1.8 Hz, 1H), 7.16 (d, *J* = 1.8 Hz, 1H), 3.32 (s, 3H), 2.61 (s, 3H), 2.41 (s, 3H). ^13^C{^1^H} NMR (126 MHz, CDCl_3_): δ 144.7, 137.7, 134.9, 134.0, 131.0, 127.7, 41.6, 21.5, 19.9. IR (neat): 2919, 1599, 1449, 1198, 1143, 1054, 989, 943, 824, 757, 621 cm^–1^. HRMS (ESI-TOF) *m*/*z*: [M + H]^+^ Calcd for C_9_H_12_INOS 309.9757; Found 309.9757. Mp = 115.9–116.8 °C.

#### *N*-Iodo-*S*-(3,5-dimethylphenyl)-*S*-methyl Sulfoximine (**2f**)

**1f** (1 mmol, 152 mg), 1.5 equiv of (NH_4_)_2_CO_3_ (1.5 mmol, 144 mg), 2.3 equiv of DIB (2.3 mmol, 741 mg), 1.1 equiv of NIS (1.1 mmol, 248 mg), isolation method A: pale yellow solid (189 mg, 61%). ^1^H NMR (500 MHz, CDCl_3_): δ 7.45 (s, 2H), 7.28 (s, 1H), 3.30 (s, 3H), 2.43 (s, 6H). ^13^C{^1^H} NMR (126 MHz, CDCl_3_): δ 139.8, 139.4, 135.5, 125.8, 42.9, 21.4. IR (neat): 2918, 1605, 1451, 1194, 1107, 1003, 972, 959, 866, 848, 746, 684 cm^–1^. HRMS (ESI-TOF) *m*/*z*: [M + H]^+^ Calcd for C_9_H_12_INOS 309.9757; Found 309.9756. Mp = 116.2–116.7 °C.

#### *N*-Iodo-*S*-(4-methoxyphenyl)-*S*-methyl Sulfoximine (**2g**)

**1g** (1 mmol, 154 mg), 1.5 equiv of (NH_4_)_2_CO_3_ (1.5 mmol, 144 mg), 2.3 equiv of DIB (2.3 mmol, 741 mg), 1.1 equiv of NIS (1.1 mmol, 248 mg), isolation method B: yellow solid (272 mg, 87%). ^1^H NMR (500 MHz, CDCl_3_): δ 7.74–7.79 (m, 2H), 7.03–7.08 (m, 2H), 3.91 (s, 3H), 3.32 (s, 3H). ^13^C{^1^H} NMR (126 MHz, CDCl_3_): δ 163.9, 131.1, 130.6, 114.9, 55.9, 43.0. IR (neat): 3000, 2913, 1590, 1574, 1491, 1257, 1199, 1088, 1005, 969, 951, 938, 837, 800, 735, 706 cm^–1^. HRMS (ESI-TOF) *m*/*z*: [M + H]^+^ Calcd for C_8_H_10_INO_2_S 311.9550; Found 311.9546. Mp = 118.6–118.9 °C.

#### *N*-Iodo-*S*-(3-methoxyphenyl)-*S*-methyl Sulfoximine (**2h**)

**1h** (1 mmol, 154 mg), 1.5 equiv of (NH_4_)_2_CO_3_ (1.5 mmol, 144 mg), 2.3 equiv of DIB (2.3 mmol, 741 mg), 1.1 equiv of NIS (1.1 mmol, 248 mg), isolation method A: yellowish solid (152 mg, 49%). ^1^H NMR (500 MHz, CDCl_3_): δ 7.47–7.53 (m, 1H), 7.41 (ddd, *J* = 7.8, 1.8, 1.0 Hz, 1H), 7.34 (dd, *J* = 2.6, 1.8 Hz, 1H), 7.19 (ddd, *J* = 8.2, 2.6, 1.0 Hz, 1H), 3.90 (s, 3H), 3.32 (s, 3H). ^13^C{^1^H} NMR (126 MHz, CDCl_3_): δ 160.5, 141.2, 130.8, 120.4, 120.4, 112.8, 55.9, 42.8. IR (neat): 3023, 2921, 1676, 1594, 1479, 1243, 1201, 998, 971, 751 cm^–1^. HRMS (ESI-TOF) *m*/*z*: [M + H]^+^ Calcd for C_8_H_10_INO_2_S 311.9550; Found 311.9554. Mp = 125.1–125.5 °C.

#### *N*-Iodo-*S*-(2-methoxyphenyl)-*S*-methyl Sulfoximine (**2i**)

**1i** (1 mmol, 154 mg), 1.5 equiv of (NH_4_)_2_CO_3_ (1.5 mmol, 144 mg), 2.3 equiv of DIB (2.3 mmol, 741 mg), 1.1 equiv of NIS (1.1 mmol, 248 mg), isolation method A: pale yellow solid (213 mg, 68%). ^1^H NMR (500 MHz, CDCl_3_): δ 7.95 (dd, *J* = 8.0, 1.8 Hz, 1H), 7.63 (ddd, *J* = 8.4, 7.5, 1.8 Hz, 1H), 7.15 (ddd, *J* = 8.0, 7.5, 1.0 Hz, 1H), 7.07 (dd, *J* = 8.4, 1.0 Hz, 1H), 3.99 (s, 3H), 3.49 (s, 3H). ^13^C{^1^H} NMR (126 MHz, CDCl_3_): δ 156.9, 135.7, 131.7, 127.3, 120.9, 112.8, 56.7, 41.0. IR (neat): 3097, 3041, 3013, 2968, 2928, 2832, 1589, 1477, 1280, 1192, 1065, 990, 958, 760 cm^–1^. HRMS (ESI-TOF) *m*/*z*: [M + H]^+^ Calcd for C_8_H_10_INO_2_S 311.9550; Found 311.9543. Mp = 127.8–129.3 °C.

#### *N*-Iodo-*S*-(4-fluorophenyl)-*S*-methyl Sulfoximine (**2j**)

**1j** (1 mmol, 156 mg), 1.5 equiv of (NH_4_)_2_CO_3_ (1.5 mmol, 144 mg), 2.3 equiv of DIB (2.3 mmol, 741 mg), 1.1 equiv of NIS (1.1 mmol, 248 mg), isolation method B: yellow solid (272 mg, 87%). ^1^H NMR (500 MHz, CDCl_3_): δ 7.79–7.86 (m, 2H), 7.26–7.32 (m, 2H), 3.36–3.53 (m, 2H), 1.25–1.31 (m, 3H). ^13^C{^1^H} NMR (126 MHz, CDCl_3_): δ 165.7 (C-F, ^1^*J*_C-F_ = 256.4 Hz), 133.9 (s), 131.8 (C-F, ^3^*J*_C-F_ = 9.5 Hz), 116.8 (C-F, ^2^*J*_C-F_ = 22.7 Hz), 49.7 (s), 8.7 (s). ^19^F NMR (471 MHz, CDCl_3_): δ −104.3 (s, 1F). IR (neat): 3095, 3063, 2974, 1922, 1581, 1486, 1221, 1193, 1154, 1085, 986, 954, 843, 816, 768, 724, 694 cm^–1^. HRMS (ESI-TOF) *m*/*z*: [M + H]^+^ Calcd for C_8_H_9_FINOS 313.9506; Found 313.9499. Mp = 96.1–97.2 °C.

#### *N*-Iodo-*S*-(3,4-dichlorophenyl)-*S*-methyl Sulfoximine (**2k**)

**1k** (1 mmol, 193 mg), 1.5 equiv of (NH_4_)_2_CO_3_ (1.5 mmol, 144 mg), 2.3 equiv of DIB (2.3 mmol, 741 mg), 1.1 equiv of NIS (1.1 mmol, 248 mg), isolation method B: pale orange solid (290 mg, 83%). ^1^H NMR (500 MHz, CDCl_3_): δ 7.94 (d, *J* = 2.1 Hz, 1H), 7.64–7.70 (m, 2H), 3.34 (s, 3H). ^13^C{^1^H} NMR (126 MHz, CDCl_3_): δ 139.9, 138.9, 134.5, 131.8, 130.4, 127.4, 42.9. IR (neat): 3078, 3002, 1567, 1449, 1368, 1206, 1141, 1093, 1033, 984, 959, 893, 812, 746, 673 cm^–1^. HRMS (ESI-TOF) *m*/*z*: [M + H]^+^ Calcd for C_7_H_6_Cl_2_INOS 349.8665; Found 349.8665. Mp = 117.4–118.2 °C.

#### *N*-Iodo-*S*-(4-bromophenyl)-*S*-methyl Sulfoximine (**2l**)

**1l** (1 mmol, 203 mg), 1.5 equiv of (NH_4_)_2_CO_3_ (1.5 mmol, 144 mg), 2.3 equiv of DIB (2.3 mmol, 741 mg), 1.1 equiv of NIS (1.1 mmol, 248 mg), isolation method B: yellow solid (310 mg, 86%). ^1^H NMR (500 MHz, CDCl_3_): δ 7.73–7.77 (m, 2H), 7.68–7.73 (m, 2H), 3.32 (s, 3H). ^13^C{^1^H} NMR (126 MHz, CDCl_3_): δ 139.1, 133.1, 130.0, 129.2, 42.9. IR (neat): 3074, 3017, 2920, 1566, 1466, 1404, 1200, 1085, 1062, 1017, 975, 953, 824, 758, 715 cm^–1^. HRMS (ESI-TOF) *m*/*z*: [M + H]^+^ Calcd for C_7_H_7_BrINOS 359.8549; Found 359.8559. Mp = 95.9–96.8 °C.

#### *N*-Iodo-*S*-(4-cyanophenyl)-*S*-methyl Sulfoximine (**2m**)

**1m** (1 mmol, 149 mg), 1.5 equiv of (NH_4_)_2_CO_3_ (1.5 mmol, 144 mg), 2.3 equiv of DIB (2.3 mmol, 741 mg), 1.1 equiv of NIS (1.1 mmol, 248 mg), isolation method B: yellow solid (205 mg, 67%). ^1^H NMR (500 MHz, CDCl_3_): δ 7.95–8.00 (m, 2H), 7.89–7.93 (m, 2H), 3.36 (s, 3H). ^13^C{^1^H} NMR (126 MHz, CDCl_3_): δ 144.5, 133.5, 129.2, 117.6, 117.3, 42.7. IR (neat): 3090, 2992, 2911, 2231, 1396, 1206, 1178, 1087, 1027, 1006, 964, 845, 833, 785, 748 cm^–1^. HRMS (ESI-TOF) *m*/*z*: [M + H]^+^ Calcd for C_8_H_7_IN_2_OS 306.9397; Found 306.9407. Mp = 100.8–102.4 °C.

#### *N*-Iodo-*S*-methyl-*S*-(4-nitrophenyl) Sulfoximine (**2n**)

**1n** (1 mmol, 169 mg), 1.5 equiv of (NH_4_)_2_CO_3_ (1.5 mmol, 144 mg), 2.3 equiv of DIB (2.3 mmol, 741 mg), 1.1 equiv of NIS (1.1 mmol, 248 mg), isolation method B: yellow solid (280 mg, 86%). ^1^H NMR (500 MHz, CDCl_3_): δ 8.43–8.47 (m, 2H), 8.03–8.10 (m, 2H), 3.38 (s, 3H). ^13^C{^1^H} NMR (126 MHz, CDCl_3_): δ 150.9, 146.1, 129.9, 124.9, 42.9. IR (neat): 3099, 3004, 2918, 1605, 1520, 1343, 1209, 1185, 1088, 1021, 1004, 961, 853, 767, 739, 716, 679 cm^–1^. HRMS (ESI-TOF) *m*/*z*: [M + H]^+^ Calcd for C_7_H_7_IN_2_O_3_S 326.9295; Found 326.9306. Mp = 97.2–100.7 °C.

#### *N*-Iodo-*S*-methyl-*S*-(2-naphthyl) Sulfoximine (**2o**)

**1o** (1 mmol, 174 mg), 1.5 equiv of (NH_4_)_2_CO_3_ (1.5 mmol, 144 mg), 2.3 equiv of DIB (2.3 mmol, 741 mg), 1.1 equiv of NIS (1.1 mmol, 248 mg), isolation method B: pale yellow solid (162 mg, 49%). ^1^H NMR (500 MHz, CDCl_3_): δ 8.47 (d, *J* = 1.9 Hz, 1H), 8.00–8.06 (m, 2H), 7.93–7.98 (m, 1H), 7.76 (dd, *J* = 8.7, 1.9 Hz, 1H), 7.70 (ddd, *J* = 8.2, 6.9, 1.4 Hz, 1H), 7.65 (ddd, *J* = 8.0, 6.8, 1.4 Hz, 1H), 3.39 (s, 3H). ^13^C{^1^H} NMR (126 MHz, CDCl_3_): δ 136.8, 135.4, 132.5, 130.6, 130.2, 129.6, 129.5, 128.1, 127.9, 122.8, 42.9. IR (neat): 3003, 2921, 1624, 1590, 1504, 1346, 1267, 1208, 1070, 985, 954, 938, 811, 753, 637 cm^–1^. HRMS (ESI-TOF) *m*/*z*: [M + H]^+^ Calcd for C_11_H_10_INOS 331.9601; Found 331.9601. Mp = 93.4–94.9 °C.

#### *N*-Iodo-*S*-phenethyl-*S*-phenyl Sulfoximine (**2p**)

**1p** (1 mmol, 214 mg), 1.5 equiv of (NH_4_)_2_CO_3_ (1.5 mmol, 144 mg), 2.3 equiv of DIB (2.3 mmol, 741 mg), 1.1 equiv of NIS (1.1 mmol, 248 mg), isolation method B: orange oil (334 mg, 90%). ^1^H NMR (500 MHz, CDCl_3_): δ 7.81–7.87 (m, 2H), 7.64–7.70 (m, 1H), 7.56–7.63 (m, 2H), 7.22–7.27 (m, 2H), 7.17–7.22 (m, 1H), 7.07–7.11 (m, 2H), 3.72 (ddd, *J* = 14.0, 12.2, 5.0 Hz, 1H), 3.56 (ddd, *J* = 14.0, 12.0, 4.9 Hz, 1H), 3.13 (ddd, *J* = 13.9, 12.0, 5.0 Hz, 1H), 2.96 (ddd, *J* = 13.9, 12.2, 4.9 Hz, 1H). ^13^C{^1^H} NMR (126 MHz, CDCl_3_): δ 138.7, 137.1, 133.8, 129.7, 129.0, 128.9, 128.4, 127.1, 56.3, 29.8. IR (neat): 3059, 3026, 2924, 1602, 1581, 1495, 1445, 1398, 1196, 1088, 1003, 981, 730, 685 cm^–1^. HRMS (ESI-TOF) *m*/*z*: [M + H]^+^ Calcd for C_14_H_14_INOS 371.9914; Found 371.9919.

#### *N*-Iodo-*S*-(2-pentafluorophenyethyl)-*S*-phenyl Sulfoximine (**2q**)

**1q** (1 mmol, 304 mg), 1.5 equiv of (NH_4_)_2_CO_3_ (1.5 mmol, 144 mg), 2.3 equiv of DIB (2.3 mmol, 741 mg), 1.1 equiv of NIS (1.1 mmol, 248 mg), isolation method B: pale yellow solid (379 mg, 82%). ^1^H NMR (500 MHz, CDCl_3_): δ 7.78–7.88 (m, 2H), 7.67–7.74 (m, 1H), 7.56–7.66 (m, 2H), 3.66–3.79 (m, 1H), 3.52–3.64 (m, 1H), 3.14–3.25 (m, 1H), 3.01–3.14 (m, 1H). ^13^C{^1^H} NMR (126 MHz, CDCl_3_): δ 145.0 (C-F, ^1^*J*_C-F_ = 247.3 Hz, ^2^*J*_C-F_ = 11.9 Hz, ^3^*J*_C-F_ = 7.9 Hz, ^4^*J*_C-F_ = 3.8 Hz), 140.4 (C-F, ^1^*J*_C-F_ = 253.9 Hz, ^2^*J*_C-F_ = 13.3 Hz, ^3^*J*_C-F_ = 5.3 Hz), 138.0 (s), 136.3–138.7 (m), 134.0 (s), 129.7 (s), 128.8 (s), 110.5 (C-F, ^2^*J*_C-F_ = 18.1 Hz, ^3^*J*_C-F_ = 4.0 Hz), 52.7 (s), 17.3 (s). ^19^F NMR (471 MHz, CDCl_3_): δ −143.0 – (−142.9) (m, 2F), −155.5 (t, *J* = 20.7 Hz, 1F), −162.2 – (−162.0) (m, 2F). IR (neat): 2987, 1520, 1505, 1208, 1089, 982, 962, 938, 748, 721, 682 cm^–1^. HRMS (ESI-TOF) *m*/*z*: [M + H]^+^ Calcd for C_14_H_9_F_5_INOS 461.9443; Found 461.9446. Mp = 115.3–116.4 °C.

#### *N*-Iodo-*S*-pentafluorophenyl-*S*-phenethyl Sulfoximine (**2r**)

**1r** (1 mmol, 304 mg), 2.625 equiv of (NH_4_)_2_CO_3_ (2.625 mmol, 252 mg), 4.025 equiv of DIB (4.025 mmol, 1296 mg), 1.1 equiv of NIS (1.1 mmol, 248 mg), isolation method B: yellow solid (369 mg, 80%). ^1^H NMR (500 MHz, CDCl_3_): δ 7.21–7.28 (m, 2H), 7.11–7.21 (m, 3H), 3.77–4.01 (m, 2H), 3.12–3.34 (m, 2H). ^13^C{^1^H} NMR (126 MHz, CDCl_3_): δ 143.7–146.3 (m), 143.0–145.6 (m), 136.6–139.1 (m), 136.2, 128.9, 128.6, 127.3, 115.2–116.7 (m), 57.5, 29.6. ^19^F NMR (471 MHz, CDCl_3_): δ −135.8 (d, *J* = 23.3 Hz, 2F), −143.8 (t, *J* = 20.8 Hz, 1F), −158.1 (t, *J* = 20.5 Hz, 2F). IR (neat): 2926, 1639, 1517, 1482, 1230, 1208, 1093, 1026, 980, 743, 691 cm^–1^. HRMS (ESI-TOF) *m*/*z*: [M + H]^+^ Calcd for C_14_H_9_F_5_INOS 461.9443; Found 461.9451. Mp = 103.0–104.1 °C.

#### *N*-Iodo-*S*-phenyl-*S*-(3-phenylpropyl) Sulfoximine (**2s**)

**1s** (1 mmol, 228 mg), 1.5 equiv of (NH_4_)_2_CO_3_ (1.5 mmol, 144 mg), 2.3 equiv of DIB (2.3 mmol, 741 mg), 1.1 equiv of NIS (1.1 mmol, 248 mg), isolation method B: light yellow solid (256 mg, 66%). ^1^H NMR (500 MHz, CDCl_3_): δ 7.75–7.80 (m, 2H), 7.64–7.70 (m, 1H), 7.56–7.61 (m, 2H), 7.23–7.27 (m, 2H), 7.16–7.21 (m, 1H), 7.05–7.09 (m, 2H), 3.44 (ddd, *J* = 14.0, 11.0, 5.3 Hz, 1H), 3.32 (ddd, *J* = 14.0, 10.9, 5.1 Hz, 1H), 2.62–2.69 (m, 2H), 2.08–2.19 (m, 1H), 1.92–2.02 (m, 1H). ^13^C{^1^H} NMR (126 MHz, CDCl_3_): δ 139.7, 138.7, 133.7, 129.7, 129.0, 128.7, 128.4, 126.5, 54.3, 34.0, 25.3. IR (neat): 3052, 2983, 2944, 1605, 1579, 1495, 1445, 1206, 1092, 987, 745, 686 cm^–1^. HRMS (ESI-TOF) *m*/*z*: [M + H]^+^ Calcd for C_15_H_16_INOS 386.0070; Found 386.0087. Mp = 99.3–100.4 °C.

#### *N*-Iodo-*S*,*S*-diphenyl Sulfoximine (**2t**)

**1t** (1 mmol, 186 mg), 1.5 equiv of (NH_4_)_2_CO_3_ (1.5 mmol, 144 mg), 2.3 equiv of DIB (2.3 mmol, 741 mg), 1.1 equiv of NIS (1.1 mmol, 248 mg), isolation method A: yellow solid (180 mg, 53%). ^1^H NMR (500 MHz, CDCl_3_): δ 7.90–7.98 (m, 4H), 7.54–7.60 (m, 2H), 7.46–7.54 (m, 4H). ^13^C{^1^H} NMR (126 MHz, CDCl_3_): δ 139.4, 133.2, 129.5, 128.4. IR (neat): 3062, 1445, 1219, 1088, 976, 757, 722, 685 cm^–1^. HRMS (ESI-TOF) *m*/*z*: [M + H]^+^ Calcd for C_12_H_10_INOS 343.9601; Found 343.9598. Mp = 114.2–114.9 °C.

#### *N*-Iodo-dibenzothiophene Sulfoximine (**2u**)

**1u** (1 mmol, 184 mg), 1.875 equiv of (NH_4_)_2_CO_3_ (1.875 mmol, 180 mg), 2.875 equiv of DIB (2.875 mmol, 926 mg), 1.1 equiv of NIS (1.1 mmol, 248 mg), isolation method A: yellow solid (252 mg, 74%). ^1^H NMR (500 MHz, CDCl_3_): δ 7.95–8.00 (m, 2H), 7.79–7.83 (m, 2H), 7.63–7.68 (m, 2H), 7.53–7.59 (m, 2H). ^13^C{^1^H} NMR (126 MHz, CDCl_3_): δ 137.9, 134.0, 132.3, 130.1, 123.2, 121.8. IR (neat): 2987, 1587, 1444, 1195, 1195, 1122, 1066, 978, 948, 753, 709 cm^–1^. HRMS (ESI-TOF) *m*/*z*: [M + H]^+^ Calcd for C_12_H_8_INOS 341.9444; Found 341.9457. Mp = 180.0–181.3 °C.

#### *N*-Iodo-*S*-benzyl-*S*-methyl Sulfoximine (**2v**)

**1v** (1 mmol, 138 mg), 1.5 equiv of (NH_4_)_2_CO_3_ (1.5 mmol, 144 mg), 2.3 equiv of DIB (2.3 mmol, 741 mg), 1.1 equiv of NIS (1.1 mmol, 248 mg), isolation method B: brown solid (176 mg, 60%). ^1^H NMR (500 MHz, CDCl_3_): δ 7.42 (app. as s, 5H), 4.47–4.56 (m, 2H), 2.89 (s, 3H). ^13^C{^1^H} NMR (126 MHz, CDCl_3_): δ 130.6, 129.4, 129.3, 129.2, 60.9, 38.0. IR (neat): 3001, 2969, 2920, 1493, 1454, 1411, 1210, 1198, 981, 945, 778, 697 cm^–1^. HRMS (ESI-TOF) *m*/*z*: [M + H]^+^ Calcd for C_8_H_10_INOS 295.9601; Found 295.9595. Mp = 81.2–83.8 °C.

#### *N*-Iodo-*S*,*S*-dimethyl Sulfoximine (**2w**)

DMSO (1 mmol, 78 mg), 1.5 equiv of (NH_4_)_2_CO_3_ (1.5 mmol, 144 mg), 1.3 equiv of DIB (1.3 mmol, 419 mg), 1.1 equiv of I_2_ (1.1 mmol, 279 mg), isolation: orange-brown solid (68 mg, 31%). ^1^H NMR (500 MHz, CDCl_3_): δ 3.21 (s, 6H). ^13^C{^1^H} NMR (126 MHz, CDCl_3_): δ 42.1. IR (neat): 2999, 2918, 1409, 1301, 1167, 1016, 970, 930, 754, 682 cm^–1^. HRMS (ESI-TOF) *m*/*z*: [M + H]^+^ Calcd for C_2_H_6_INOS 219.9288; Found 219.9286. Mp = 118.4–119.0 °C.

#### *N*-Iodo-*S*-(1-dodecyl)-*S*-methyl Sulfoximine (**2x**)

**1x** (1 mmol, 216 mg), 1.5 equiv of (NH_4_)_2_CO_3_ (1.5 mmol, 144 mg), 2.3 equiv of DIB (2.3 mmol, 741 mg), 1.1 equiv of NIS (1.1 mmol, 248 mg), isolation method B: brown-red solid (156 mg, 42%). ^1^H NMR (500 MHz, CDCl_3_): δ 3.18–3.34 (m, 2H), 3.07 (s, 3H), 1.77–1.86 (m, 2H), 1.40–1.48 (m, 2H), 1.22–1.38 (m, 16H), 0.88 (t, *J* = 6.8 Hz, 3H). ^13^C{^1^H} NMR (126 MHz, CDCl_3_): δ 54.6, 40.2, 32.0, 29.7, 29.7, 29.6, 29.4, 29.3, 29.1, 28.3, 23.9, 22.8, 14.2. IR (neat): 2916, 2849, 1468, 1184, 998, 929, 719 cm^–1^. HRMS (ESI-TOF) *m*/*z*: [M + H]^+^ Calcd for C_13_H_28_INOS 374.1009; Found 374.1009. Mp = 51.5–51.9 °C.

#### *N*-Iodo-*S*,*S*-(di-1-octyl) Sulfoximine (**2y**)

**1y** (1 mmol, 259 mg), 1.5 equiv of (NH_4_)_2_CO_3_ (1.5 mmol, 144 mg), 2.3 equiv of DIB (2.3 mmol, 741 mg), 1.1 equiv of NIS (1.1 mmol, 248 mg), isolation method B: brown oil (211 mg, 51%). ^1^H NMR (500 MHz, CDCl_3_): δ 2.85–3.33 (m, 4H), 1.70–1.85 (m, 4H), 1.38–1.47 (m, 4H), 1.21–1.38 (m, 16H), 0.86–0.91 (m, 6H). ^13^C{^1^H} NMR (126 MHz, CDCl_3_): δ 52.4, 31.7, 29.0, 29.0, 28.3, 23.2, 22.6, 14.1. IR (neat): 2954, 2922, 2854, 1630, 1461, 1188, 982, 756, 722 cm^–1^. HRMS (ESI-TOF) *m*/*z*: [M + H]^+^ Calcd for C_16_H_34_INOS 416.1479; Found 416.1469.

#### *N*-Iodo-*S*-methyl-*S*-(2-pyridine) Sulfoximine (**2z**)

**1z** (1 mmol, 125 mg), 1.5 equiv of (NH_4_)_2_CO_3_ (1.5 mmol, 144 mg), 2.3 equiv of DIB (2.3 mmol, 741 mg), 1.1 equiv of NIS (1.1 mmol, 248 mg), isolation method A: yellow solid (187 mg, 66%). ^1^H NMR (500 MHz, CDCl_3_): δ 8.77 (ddd, *J* = 4.7, 1.8, 0.9 Hz, 1H), 8.12–8.17 (m, 1H), 7.97–8.03 (m, 1H), 7.57 (ddd, *J* = 7.6, 4.7, 1.2 Hz, 1H), 3.50 (s, 3H). ^13^C{^1^H} NMR (126 MHz, CDCl_3_): δ 157.6, 150.6, 138.1, 127.4, 123.4, 39.5. IR (neat): 3017, 2915, 1577, 1560, 1421, 1206, 1082, 984, 785, 752 cm^–1^. HRMS (ESI-TOF) *m*/*z*: [M + H]^+^ Calcd for C_6_H_7_IN_2_OS 282.9397; Found 282.9384. Mp = 103.0–105.4 °C.

### One-Pot Synthesis of *N*-Bromo and *N*-Chloro Sulfoximines **3** and **4**

This procedure is similar to the one-pot synthesis of *N*-iodo sulfoximines. A mixture of methyl phenyl sulfide (**1a**, 1 mmol, 124 mg), 5 mL of MeOH, 1.5 equiv of (NH_4_)_2_CO_3_ (1.5 mmol, 144 mg), and 2.3 equiv of DIB (2.3 mmol, 741 mg) was charged into a 10 mL round-bottom flask equipped with a magnetic stirrer. The flask was sealed with a glass stopper, and the reaction mixture was let to stir vigorously for 1 h. Then, 1.75 equiv of NBS (1.75 mmol, 312 mg) or 1.75 equiv of NCS (1.75 mmol, 235 mg) was added, and the stirring was continued for another hour. The reaction solvent was removed under reduced pressure; the residue was redissolved in small amounts of DCM and subjected to flash chromatography under pressure (nitrogen gas) through a short plug of SiO_2_ as a stationary phase and DCM as eluant. The progress of separation was monitored by TLC analysis. Fractions containing product **3** or **4** were collected into 50–100 mL flasks, and the solvent was removed under reduced pressure to obtain a lightly colored semisolid. It was redissolved in small amounts of DCM, triturated with large amounts of PE (or hexane), and evaporated to dryness under reduced pressure. The process was repeated 2–3 times to remove any residual PhI, resulting in pure products **3** (194 mg, 0.83 mmol, 83%) as a colorless solid, and **4** (120 mg, 0.63 mmol, 63%) as a colorless solid.

#### *N*-Bromo-*S*-methyl-*S*-phenyl Sulfoximine^43^ (**3**)

**1a** (1 mmol, 124 mg), 1.5 equiv of (NH_4_)_2_CO_3_ (1.5 mmol, 144 mg), 2.3 equiv of DIB (2.3 mmol, 741 mg), 1.75 equiv of NBS (1.75 mmol, 312 mg), isolation method B: colorless solid (194 mg, 83%). ^1^H NMR (500 MHz, CDCl_3_): δ 7.87–7.95 (m, 2H), 7.66–7.72 (m, 1H), 7.58–7.65 (m, 2H), 3.30 (s, 3H). ^13^C{^1^H} NMR (126 MHz, CDCl3): δ 137.9, 134.1, 129.8, 128.8, 42.7. IR (neat): 3004, 2913, 1446, 1415, 1222, 1088, 981, 964, 940, 777, 738, 683 cm^–1^.

#### *N*-Chloro-*S*-methyl-*S*-phenyl Sulfoximine^43^ (**4**)

**1a** (1 mmol, 124 mg), 1.5 equiv of (NH_4_)_2_CO_3_ (1.5 mmol, 144 mg), 2.3 equiv of DIB (2.3 mmol, 741 mg), 1.75 equiv of NCS (1.75 mmol, 235 mg), isolation method B: colorless solid (120 mg, 63%). ^1^H NMR (500 MHz, CDCl_3_): δ 7.92–7.98 (m, 2H), 7.69–7.74 (m, 1H), 7.60–7.67 (m, 2H), 3.27 (s, 3H). ^13^C{^1^H} NMR (126 MHz, CDCl_3_): δ 136.5, 134.3, 129.9, 129.2, 42.2. IR (neat): 3016, 2994, 2915, 1709, 1447, 1215, 1088, 1006, 990, 968, 936, 742, 682, 644 cm^–1^.

### Reactions with **2a**: Iodination and Oxidation Procedures

A mixture of **2a** (1.1–3.3 mmol), 5 mL of AcOH, and compound **5**, **7**, **9**, **11**, or **13** (1 mmol) was charged into a 10 mL round-bottom flask equipped with a magnetic stirrer. The flask was sealed with a glass stopper, and the reaction mixture was let to stir for a specific time (*t*) at a specific temperature (*T*) and monitored by TLC analysis. After completion, the reaction mixture was diluted with DCM and 10% aqueous solution HCl was added. Products were extracted with DCM three times; the organic phase was washed with 10% aqueous NaHSO_3_ or Na_2_S_2_O_3_ solution, 10% aqueous solution of HCl, and water, respectively, and dried over anhydrous MgSO_4_ or Na_2_SO_4_. Solvent was removed under reduced pressure to afford pure products **6**, **8**, **10**, **12**, or **14**.

#### 4-Iodoanisole^62^ (**6**)

**5** (1 mmol, 108 mg), 1.1 equiv of **2a** in one portion (1.1 mmol, 309 mg), extraction: white solid (202 mg, 86%). ^1^H NMR (500 MHz, CDCl_3_): δ 7.52–7.58 (m, 2H), 6.64–6.71 (m, 2H), 3.77 (s, 3H). ^13^C{^1^H} NMR (126 MHz, CDCl_3_): δ 159.6, 138.3, 116.5, 82.8, 55.4. IR (neat): 3005, 2967, 2938, 2838, 1584, 1567, 1484, 1454, 1285, 1241, 1175, 1102, 1025, 997, 829, 807, 784 cm^–1^.

#### 2,4,6-Triiodophenol^63^ (**8**)

**7** (1 mmol, 94 mg), 3.3 equiv of **2a** in one portion (3.3 mmol, 927 mg), extraction: pale white solid (465 mg, 99%). ^1^H NMR (500 MHz, CDCl_3_): δ 7.93 (s, 2H), 5.77 (s, 1H, O*H*). ^13^C{^1^H} NMR (126 MHz, CDCl_3_): δ 153.9, 146.5, 83.5, 83.5. IR (neat): 3428, 3049, 1539, 1432, 1369, 1293, 1258, 1229, 1170, 1133, 858, 697, 629 cm^–1^.

#### 1-Iodo-4-methoxynaphthalene^62^ (**10**)

**9** (1 mmol, 158 mg), 1.1 equiv of **2a** in portions over 2 h (1.1 mmol, 309 mg), extraction: pale yellow solid (276 mg, 97%). ^1^H NMR (500 MHz, CDCl_3_): δ 8.20–8.27 (m, 1H), 8.00–8.06 (m, 1H), 7.95 (d, *J* = 8.2 Hz, 1H), 7.56–7.63 (m, 1H), 7.48–7.55 (m, 1H), 6.59 (d, *J* = 8.1 Hz, 1H), 3.99 (s, 3H). ^13^C{^1^H} NMR (126 MHz, CDCl_3_): δ 156.3, 137.0, 134.7, 131.8, 128.2, 126.7, 126.1, 122.6, 105.7, 88.3, 55.8. IR (neat): 2932, 2837, 1828, 1584, 1503, 1454, 1416, 1364, 1315, 1258, 1238, 1155, 1082, 1027, 989, 807, 757, 712, 614 cm^–1^.

#### 2,6-Diiodo-4-nitro-phenol^63^ (**12**)

**11** (1 mmol, 139 mg), 2.2 equiv of **2a** in one portion (2.2 mmol, 618 mg), extraction: yellow solid (384 mg, 98%). ^1^H NMR (500 MHz, CDCl_3_): δ 8.60 (s, 2H). ^13^C{^1^H} NMR (126 MHz, CDCl_3_): δ 159.1, 142.3, 134.9, 80.9. IR (neat): 3368, 3068, 1576, 1502, 1441, 1398, 1312, 1228, 1114, 898, 739, 675 cm^–1^.

#### Diphenyl Disulfide^64^ (**14**)

**13** (1 mmol, 110 mg), 1.1 equiv of **2a** in portions over 1 h (1.1 mmol, 309 mg), extraction: white solid (109 mg, 100%). ^1^H NMR (500 MHz, CDCl_3_): δ 7.45–7.52 (m, 4H), 7.24–7.31 (m, 4H), 7.18–7.24 (m, 2H). ^13^C{^1^H} NMR (126 MHz, CDCl_3_): δ 137.1, 129.2, 127.6, 127.3. IR (neat): 3066, 2976, 2915, 1573, 1473, 1435, 1232, 1071, 1020, 995, 733, 684 cm^–1^.

### Synthesis of *N*-(Trifluoromethanesulfenyl) Sulfoximines **15** from *N*-Iodo Sulfoximines **2**

The method is similar to the known procedure.^43^ AgSCF_3_ was prepared according to the literature procedure.^65^

A mixture of **2a**, **2g**, **2n**, or **2t** (0.304–1 mmol) in dry MeCN (2 mL per 1 mmol of **2**) was charged into a 10 mL flask equipped with a magnetic stirrer. The flask was sealed with a septum under an argon atmosphere, and a solution of AgSCF_3_ (1.2 equiv, 3 mL of MeCN per 1.2 mmol of AgSCF_3_) was added. The reaction mixture was let to stir in the dark for a specified time (20–60 min) as judged by TLC analysis. After completion, MeCN was removed under reduced pressure; the crude residue was redissolved in small amounts of DCM and subjected to flash chromatography under pressure (nitrogen gas) through a short plug of SiO_2_ as stationary phase and DCM as eluant. Fractions containing product **15** were collected, and solvent was removed under reduced pressure to afford products **15a**, **15g**, **15n**, or **15t** as white solids.

#### *N*-(Trifluoromethanesulfenyl)-*S*-methyl-*S*-phenyl Sulfoximine^43^ (**15a**)

**2a** (1 mmol, 281 mg), 1.2 equiv of AgSCF_3_ (1.2 mmol, 251 mg), 60 min, flash (SiO_2_/DCM): white solid (195 mg, 76%). ^1^H NMR (500 MHz, CDCl_3_): δ 7.87–7.97 (m, 2H), 7.68–7.75 (m, 1H), 7.59–7.66 (m, 2H), 3.29 (s, 3H). ^13^C{^1^H} NMR (126 MHz, CDCl_3_): δ 137.9, 134.4, 130.5 (C-F, ^1^*J*_C-F_ = 312.4 Hz), 129.8, 128.4, 43.7. ^19^F NMR (471 MHz, CDCl_3_): δ −50.7 (s, 3F). IR (neat): 3062, 3028, 3006, 2926, 1581, 1449, 1209, 1111, 1087, 986, 960, 785, 747, 718, 687 cm^–1^.

#### *N*-(Trifluoromethanesulfenyl)-*S*-(4-methoxyphenyl)-*S*-methyl Sulfoximine^43^ (**15g**)

**2g** (1 mmol, 311 mg), 1.2 equiv of AgSCF_3_ (1.2 mmol, 251 mg), 60 min, flash (SiO_2_/DCM): white solid (267 mg, 94%). ^1^H NMR (500 MHz, CDCl_3_): δ 7.79–7.85 (m, 2H), 7.03–7.09 (m, 2H), 3.90 (s, 3H), 3.25 (s, 3H). ^13^C{^1^H} NMR (126 MHz, CDCl_3_): δ 164.2, 130.5 (C-F, ^1^*J*_C-F_ = 312.5 Hz), 130.4, 128.4, 114.9, 55.8, 43.8. ^19^F NMR (471 MHz, CDCl_3_): δ −50.7 (s, 3F). IR (neat): 3017, 2932, 2844, 1592, 1497, 1263, 1219, 1088, 1009, 978, 833, 803, 766, 705 cm^–1^.

#### *N*-(Trifluoromethanesulfenyl)-*S*-(4-nitrophenyl)-*S*-methyl Sulfoximine^43^ (**15n**)

**2n** (0.304 mmol, 99 mg), 1.2 equiv of AgSCF_3_ (0.365 mmol, 76 mg), 20 min, flash (SiO_2_/DCM): white solid (62 mg, 68%). ^1^H NMR (500 MHz, CDCl_3_): δ 8.44–8.50 (m, 2H), 8.10–8.16 (m, 2H), 3.36 (s, 3H). ^13^C{^1^H} NMR (126 MHz, CDCl_3_): δ 151.2, 143.9, 130.3 (C-F, ^1^*J*_C-F_ = 312.4 Hz), 130.0, 124.9, 43.6. ^19^F NMR (471 MHz, CDCl_3_): δ −50.4 (s, 3F). IR (neat): 3109, 3053, 3022, 2926, 1604, 1527, 1345, 1226, 1136, 1105, 1087, 990, 963, 739, 728 cm^–1^.

#### *N*-(Trifluoromethanesulfenyl)-*S*,*S*-diphenyl Sulfoximine^43^ (**15t**)

**2t** (0.515 mmol, 147 mg), 1.2 equiv of AgSCF_3_ (0.515 mmol, 108 mg), 20 min, flash (SiO_2_/DCM): white solid (88 mg, 65%). ^1^H NMR (500 MHz, CDCl_3_): δ 7.92–8.01 (m, 4H), 7.58–7.64 (m, 2H), 7.50–7.58 (m, 4H). ^13^C{^1^H} NMR (126 MHz, CDCl_3_): δ 139.0, 133.8, 130.5 (C-F, ^1^*J*_C-F_ = 312.5 Hz), 129.6, 128.5. ^19^F NMR (471 MHz, CDCl_3_): δ −50.4 (s, 3F). IR (neat): 3066, 1580, 1475, 1446, 1221, 1151, 1107, 1081, 953, 761, 730, 683 cm^–1^.

### Preparation of Other Sulfides

#### Synthesis of **1p**

A mixture of thiophenol (2.200 g, 20 mmol) and styrene (2.184 g, 21 mmol) was charged into a 100 mL round-bottom flask. The flask was sealed with a glass stopper, equipped with a magnetic stirrer, and let to stir overnight at 60 °C. After completion of the reaction as determined by TLC analysis, the crude product was purified by vacuum distillation, furnishing **1p** as a yellow oil in 90% yield.

#### Synthesis of **1q**

A mixture of thiophenol (1.533 g, 13.9 mmol) and pentafluorostyrene (2.980 g, 15.35 mmol) was charged into a 100 mL round-bottom flask. The flask was sealed with a glass stopper, equipped with a magnetic stirrer, and let to stir overnight at 60 °C. After completion of the reaction as determined by TLC analysis, the crude product was purified by vacuum distillation, furnishing **1q** as a yellow oil in 85% yield.

#### Synthesis of **1r**

A mixture of pentafluorothiophenol (4.000 g, 20 mmol) and styrene (2.080 g, 20 mmol) was charged into a 100 mL round-bottom flask. The flask was sealed with a glass stopper, equipped with a magnetic stirrer, and let to stir overnight at 60 °C. After completion of the reaction as determined by TLC analysis, the crude product was purified by vacuum distillation, furnishing **1r** as a yellow oil in 92% yield.

#### Synthesis of **1s**

To a solution of thiophenol (2.200 g, 20 mmol) in 80 mL of MeCN, K_2_CO_3_ (3.312 g, 24 mmol) and 1-bromo-3-phenylpropane (4.179 g, 21 mmol) were consecutively added, and the mixture was let to stir overnight at room temperature. After completion, as determined by TLC analysis, the reaction solvent was evaporated, and the residue was partitioned three times between DCM and water. The combined organic phase was dried over anhydrous MgSO_4_, and solvent was removed. The oily residue was purified by vacuum distillation, furnishing **1s** as a yellow oil in 94% yield.

#### Synthesis of **1y**

A mixture of 1-octanethiol (2.926 g, 20 mmol) and 1-octene (2.244 g, 20 mmol) was charged into a 100 mL round-bottom flask equipped with a magnetic stirrer. The flask was sealed with a glass stopper, and the reaction mixture was let to stir overnight at 60 °C. After completion, as determined by TLC analysis, the crude product was purified by vacuum distillation, furnishing **1y** as a yellow oil in 92% yield.

## Supporting Information Available

Experimental procedures and analytic data for the compounds described and copies of NMR spectra (PDF)

## Dedication

This paper is dedicated to Professor Antonio Togni, ETH Zurich, on the occasion of his 65th anniversary and his lifetime research and pedagogical achievements.

## References

[ref1] Tota A.; Zenzola M.; Chawner S. J.; John-Campbell S. St.; Carlucci C.; Romanazzi G.; Degennaro L.; Bull J. A.; Luisi R. Synthesis of NH-sulfoximines from sulfides by chemoselective one-pot N- and O-transfers. Chem. Commun. 2017, 53, 348–351. 10.1039/C6CC08891K. 27929152

[ref2] Xie Y.; Zhou B.; Zhou S.; Zhou S.; Wei W.; Liu J.; Zhan Y.; Cheng D.; Chen M.; Li Y.; Wang B.; Xue X.-s.; Li Z. Sulfimine-Promoted Fast O Transfer: One–step Synthesis of Sulfoximine from Sulfide. ChemistrySelect 2017, 2, 1620–1624. 10.1002/slct.201700132.

[ref3] Yu H.; Li Z.; Bolm C. Iron(II)-Catalyzed Direct Synthesis of NH Sulfoximines from Sulfoxides. Angew. Chem., Int. Ed. 2018, 57, 324–327. 10.1002/anie.201710498. 29155462

[ref4] Zenzola M.; Doran R.; Degennaro L.; Luisi R.; Bull J. A. Transfer of Electrophilic NH Using Convenient Sources of Ammonia: Direct Synthesis of NH Sulfoximines from Sulfoxides. Angew. Chem., Int. Ed. 2016, 55, 7203–7207. 10.1002/anie.201602320. PMC5074267 27126053

[ref5] Lohier J.-F.; Glachet T.; Marzag H.; Gaumont A.-C.; Reboul V. Mechanistic investigation of the NH-sulfoximination of sulphide. Evidence for λ^6^-sulfanenitrile intermediates. Chem. Commun. 2017, 53, 2064–2067. 10.1039/C6CC09940H. 28133647

[ref6] Zenzola M.; Doran R.; Luisi R.; Bull J. A. Synthesis of Sulfoximine Carbamates by Rhodium-Catalyzed Nitrene Transfer of Carbamates to Sulfoxides. J. Org. Chem. 2015, 80, 6391–6399. 10.1021/acs.joc.5b00844. 25989821

[ref7] Miao J.; Richards N. G. J.; Ge H. Rhodium-catalyzed direct synthesis of unprotected NH-sulfoximines from sulfoxides. Chem. Commun. 2014, 50, 9687–9689. 10.1039/C4CC04349A. 25016917

[ref8] Zhang G.; Tan H.; Chen W.; Shen H. C.; Lu Y.; Zheng C.; Xu H. Synthesis of NH-Sulfoximines by Using Recyclable Hypervalent Iodine(III) Reagents under Aqueous Micellar Conditions. ChemSusChem 2020, 13, 922–928. 10.1002/cssc.201903430. 31950602

[ref9] Davies T. Q.; Tilby M. J.; Ren J.; Parker N. A.; Skolc D.; Hall A.; Duarte F.; Willis M. C. Harnessing Sulfinyl Nitrenes: A Unified One-Pot Synthesis of Sulfoximines and Sulfonimidamides. J. Am. Chem. Soc. 2020, 142, 15445–15453. 10.1021/jacs.0c06986. 32841007 PMC7498162

[ref10] Sirvent J. A.; Lücking U. Novel Pieces for the Emerging Picture of Sulfoximines in Drug Discovery: Synthesis and Evaluation of Sulfoximine Analogues of Marketed Drugs and Advanced Clinical Candidates. ChemMedChem 2017, 12, 487–501. 10.1002/cmdc.201700044. 28221724 PMC5485063

[ref11] Borst M. L. G.; Ouairy C. M. J.; Fokkema S. C.; Cecchi A.; Kerckhoffs J. M. C. A.; de Boer V. L.; van den Boogaard P. J.; Bus R. F.; Ebens R.; van der Hulst R.; Knol J.; Libbers R.; Lion Z. M.; Settels B. W.; de Wever E.; Attia K. A.; Sinnema P.-J.; de Gooijer J. M.; Harkema K.; Hazewinkel M.; Snijder S.; Pouwer K. Polycyclic Sulfoximines as New Scaffolds for Drug Discovery. ACS Comb. Sci. 2018, 20, 335–343. 10.1021/acscombsci.7b00150. 29714998

[ref12] Mäder P.; Kattner L. Sulfoximines as Rising Stars in Modern Drug Discovery? Current Status and Perspective on an Emerging Functional Group in Medicinal Chemistry. J. Med. Chem. 2020, 63, 14243–14275. 10.1021/acs.jmedchem.0c00960. 32870008

[ref13] Frings M.; Bolm C.; Blum A.; Gnamm C. Sulfoximines from Medicinal Chemist’s Perspective: Physicochemical and in vitro Parameters Relevant for Drug Discovery. Eur. J. Med. Chem. 2017, 126, 225–245. 10.1016/j.ejmech.2016.09.091. 27821325

[ref14] Lücking U. Neglected sulfur(VI) pharmacophores in drug discovery: exploration of novel chemical space by the interplay of drug design and method development. Org. Chem. Front. 2019, 6, 1319–1324. 10.1039/C8QO01233D.

[ref15] Lücking U. Sulfoximines: A Neglected Opportunity in Medicinal Chemistry. Angew. Chem., Int. Ed. 2013, 52, 9399–9408. 10.1002/anie.201302209. 23934828

[ref16] Thota N.; Makam P.; Rajbongshi K. K.; Nagiah S.; Abdul N. S.; Chuturgoon A. A; Kaushik A.; Lamichhane G.; Somboro A. M.; Kruger H. G.; Govender T.; Naicker T.; Arvidsson P. I *N*-Trifluoromethylthiolated Sulfonimidamides and Sulfoximines: Anti-microbial, Anti-mycobacterial, and Cytotoxic Activity. ACS Med. Chem. Lett. 2019, 10, 1457–1461. 10.1021/acsmedchemlett.9b00285. 31620233 PMC6792286

[ref17] Chizema M.; Mabasa T. F.; Hoppe H. C.; Kinfe H. H. Design, synthesis, and antiplasmodial evaluation of a series of novel sulfoximine analogues of carbohydrate-based thiochromans. Chem. Biol. Drug Des. 2019, 93, 254–261. 10.1111/cbdd.13408. 30264436

[ref18] Glanchet T.; Franck X.; Reboul V. Late-Stage Sulfoximination: Improved Synthesis of the anticancer Drug Candidate Atuveciclib. Synthesis 2018, 50, 971–975. 10.1055/s-0037-1610316.

[ref19] Devendar P.; Yang G.-F. Sulfur-Containing Agrochemicals. Top. Curr. Chem. (Z) 2017, 375, 82 10.1007/s41061-017-0169-9. 28993992

[ref20] Gnamm C.; Jeanguenat A.; Dutton A. C.; Grimm C.; Kloer D. P.; Crossthwaite A. J. Novel diamide insecticides: Sulfoximines, sulfonimidamides and other new sulfonimidoyl derivatives. Bioorg. Med. Chem. Lett. 2012, 22, 3800–3806. 10.1016/j.bmcl.2012.03.106. 22552196

[ref21] Wiezorek S.; Lamers P.; Bolm C. Conversion and degradation pathways of sulfoximines. Chem. Soc. Rev. 2019, 48, 5408–5423. 10.1039/C9CS00483A. 31535112

[ref22] Bull J. A.; Degennaro L.; Luisi R. Straightforward Strategies for the Preparation of NH-Sulfoximines: A Serendipitous Story. Synlett 2017, 28, 2525–2538. 10.1055/s-0036-1590874.

[ref23] Barthelemy A.-L.; Magnier E. Recent trends in perfluorinated sulfoximines. C. R. Chim. 2018, 21, 711–722. 10.1016/j.crci.2018.01.004.

[ref24] Mulina O. M.; Ilovaisky A. I.; Terent’ev A. O. Oxidative Coupling with S–N Bond Formation. Eur. J. Org. Chem. 2018, 2018, 4648–4672. 10.1002/ejoc.201800838.

[ref25] Bizet V.; Hendriks C. M. M.; Bolm C. Sulfur imidations: access to sulfimides and sulfoximines. Chem. Soc. Rev. 2015, 44, 3378–3390. 10.1039/C5CS00208G. 25941981

[ref26] Bizet V.; Kowalczyk R.; Bolm C. Fluorinated sulfoximines: synthesis, properties and applications. Chem. Soc. Rev. 2014, 43, 2426–2438. 10.1039/c3cs60427f. 24549291

[ref27] Shen X.; Hu J. Fluorinated Sulfoximines: Preparation, Reactions and Applications. Eur. J. Org. Chem. 2014, 2014, 4437–4451. 10.1002/ejoc.201402086.

[ref28] Wang H.; Zhang D.; Bolm C. Photocatalytic Additions of 1-Sulfoximidoyl-1,2-Benziodoxoles to Styrenes. Chem. - Eur. J. 2018, 24, 14942–14945. 10.1002/chem.201803975. 30079969

[ref29] Wang C.; Wang H.; Bolm C. Sulfoximines with α-Ketoester Functionalities at Nitrogen from Cyanoacetates and Air. Adv. Synth. Catal. 2021, 363, 747–750. 10.1002/adsc.202001264.

[ref30] Wang H.; Cheng Y.; Becker P.; Raabe G.; Bolm C. Synthesis of Sulfoximidoyl-Containing Hypervalent Iodine(III) Reagents and Their Use in Transition-Metal-Free Sulfoximidations of Alkynes. Angew. Chem., Int. Ed. 2016, 55, 12655–12658. 10.1002/anie.201605743. 27444808

[ref31] Wang H.; Zhang D.; Sheng H.; Bolm C. Sulfoximidoyl-Containing Hypervalent Iodine(III) Reagents: 1-Sulfoximidoyl-1,2-benziodoxoles. J. Org. Chem. 2017, 82, 11854–11858. 10.1021/acs.joc.7b01535. 28745886

[ref32] Wang H.; Zhang D.; Bolm C. Sulfoximidations of Benzylic C–H bonds by Photocatalysis. Angew. Chem., Int. Ed. 2018, 57, 5863–5866. 10.1002/anie.201801660. 29575520

[ref33] Wang C.; Tu Y.; Ma D.; Bolm C. Photocatalytic Fluoro Sulfoximidations of Styrenes. Angew. Chem., Int. Ed. 2020, 59, 14134–14137. 10.1002/anie.202005844. PMC7496861 32415689

[ref34] Wang H.; Zhang D.; Cao M.; Bolm C. Electrophylic Sulfoximidations of Thiols by Hypervalent Iodine Reagents. Synthesis 2019, 51, 271–275. 10.1055/s-0037-1610369.

[ref35] Cao X.; Chen Z.; Gong S.; Pan K.; Zhou C.; Huang T.; Chai D.; Zhan Q.; Li N.; Zou Y.; Liu H.; Yang C. Designing versatile sulfoximine as accepting unit to regulate the photophysical properties of TADF emitters towards high-performance OLEDs. Chem. Eng. J. 2020, 399, 125648 10.1016/j.cej.2020.125648.

[ref36] Otocka S.; Kwiatkowska M.; Madalińska L.; Kiełbasiński P. Chiral Organosulfur Ligands/Catalysts with a Stereogenic Sulfur Atom: Applications in Asymmetric Synthesis. Chem. Rev. 2017, 117, 4147–4181. 10.1021/acs.chemrev.6b00517. 28191933

[ref37] Wojaczyńska E.; Wojaczyński J. Modern Stereoselective Synthesis of Chiral Sulfinyl Compounds. Chem. Rev. 2020, 120, 4578–4611. 10.1021/acs.chemrev.0c00002. 32347719 PMC7588045

[ref38] Wimmer A.; König B. *N*-Arylation of *NH*-Sulfoximines via Dual Nickel Photocatalysis. Org. Lett. 2019, 21, 2740–2744. 10.1021/acs.orglett.9b00698. 30938532 PMC6480096

[ref39] Li Z.; Frings M.; Yu H.; Bolm C. Organocatalytic Synthesis of Sulfoximidoyl-Containing Carbamates from Sulfoximines and Morita–Baylis–Hillman Carbonates. Org. Lett. 2019, 21, 3119–3122. 10.1021/acs.orglett.9b00772. 30986071

[ref40] Choi W.; Kim J.; Ryu T.; Kim K.-B.; Lee P. H. Synthesis of *N*-Imidoyl and *N*-Oxoimidoyl Sulfoximines from 1-Alkynes, *N*-Sulfonyl Azides, and Sulfoximines. Org. Lett. 2015, 17, 3330–3333. 10.1021/acs.orglett.5b01553. 26102299

[ref41] Xu J.; Song Q. Synthesis of fully-substituted 1,2,3-triazoles *via* copper(I)-catalyzed three-component coupling of sulfoximines, alkynes and azides. Org. Chem. Front. 2017, 4, 938–942. 10.1039/C6QO00725B.

[ref42] Priebbenow D. L.; Bolm C. C–H Activation of Methyl Arenes in the MnO_2_-Mediated Aroylation of *N*-Chlorosulfoximines. Org. Lett. 2014, 16, 1650–1652. 10.1021/ol5003016. 24588424

[ref43] Bohnen C.; Bolm C. *N*-Trifluoromethylthiolated Sulfoximines. Org. Lett. 2015, 17, 3011–3013. 10.1021/acs.orglett.5b01384. 26029817

[ref44] Teng F.; Cheng J.; Bolm C. Silver-Mediated *N*-Trifluoromethylation of Sulfoximines. Org. Lett. 2015, 17, 3166–3169. 10.1021/acs.orglett.5b01537. 26057854

[ref45] Kowalczyk R.; Edmunds A. J. F.; Hall R. G.; Bolm C. Synthesis of CF_3_-Substituted Sulfoximines from Sulfonimidoyl Fluorides. Org. Lett. 2011, 13, 768–771. 10.1021/ol103030w. 21235264

[ref46] Barthelemy A.-L.; Certal V.; Dagousset G.; Anselmi E.; Bertin L.; Fabien L.; Salgues B.; Courtes P.; Poma C.; El-Ahmad Y.; Magnier E. Optimization and Gram-Scale Preparation of S-Trifluoromethyl Sulfoximines and Sulfilimino Iminiums, Powerful Reagents for the Late Stage Introduction of the CF_3_ Group. Org. Process Res. Dev. 2020, 24, 704–712. 10.1021/acs.oprd.9b00403.

[ref47] Le T.-N.; Diter P.; Pégot B.; Bournaud C.; Toffano M.; Guillot R.; Vo-Thanh G.; Magnier E. *S*-Trifluoromethyl Sulfoximine as a Directing Group in *Ortho*-Lithiation Reaction toward Structural Complexity. Org. Lett. 2016, 18, 5102–5105. 10.1021/acs.orglett.6b02548. 27682457

[ref48] Bennai N.; Ibrahim N.; Marrot J.; Belkadi M.; Alami M.; Magnier E.; Anselmi E.; Messaoudi S. Synthesis of S-Trifluoromethyl S-Arylsulfoximine Thioglycosides through Pd-Catalyzed Migita Cross-Coupling. Eur. J. Org. Chem. 2020, 2020, 4972–4981. 10.1002/ejoc.202000821.

[ref49] Wang H.; Frings M.; Bolm C. Halocyclizations of Unsaturated Sulfoximines. Org. Lett. 2016, 18, 2431–2434. 10.1021/acs.orglett.6b00958. 27168417

[ref50] Zhang D.; Wang H.; Cheng H.; Hernández J. G.; Bolm C. An Iodine-Mediated Hofmann-Löffler-Freytag Reaction of Sulfoximines Leading to Dihydroisothiazole Oxides. Adv. Synth. Catal. 2017, 359, 4274–4277. 10.1002/adsc.201701178.

[ref51] Zheng W.; Tan M.; Yang L.; Zhou L.; Zeng Q. I_2_-Catalyzed N-Sulfonylation of Sulfoximines with Sulfinates in Water at Room Temperature. Eur. J. Org. Chem. 2020, 2020, 1764–1768. 10.1002/ejoc.202000120.

[ref52] Lynes W. I. N-Halosulfoximines. US 5,557,206, 1971.

[ref53] Jereb M.; Zupan M.; Stavber S. Effective and selective iodofunctionalisation of organic molecules in water using the iodine-hydrogen peroxide tandem. Chem. Commun. 2004, 2614–2615. 10.1039/B409919B. 15543306

[ref54] Stavber S.; Jereb M.; Zupan M. Selectfluor F-TEDA-BF_4_ mediated and solvent directed iodination of aryl alkyl ketones using elemental iodine. Chem. Commun. 2002, 488–489. 10.1039/b200240j. 12120554

[ref55] Jereb M.; Zupan M.; Stavber S. Hydrogen peroxide induced iodine transfer into alkenes. Green Chem. 2005, 7, 100–104. 10.1039/b407592g.

[ref56] Jereb M.; Hribernik L. Conversion of thiols into sulfonyl halogenides under aerobic and metal-free conditions. Green Chem. 2017, 19, 2286–2295. 10.1039/C7GC00556C.

[ref57] Zupanc A.; Jereb M. NaSH-HCl mediated reduction of sulfoxides into sulfides under organic solvent-free reaction conditions. Green Chem. Lett. Rev. 2020, 13, 341–348. 10.1080/17518253.2020.1838625.

[ref58] Meyer E. A.; Castellano R. K.; Diederich F. Interactions with Aromatic Rings in Chemical and Biological Recognition. Angew. Chem., Int. Ed. 2003, 42, 1210–1250. 10.1002/anie.200390319. 12645054

[ref59] Giese M.; Albrecht M.; Rissanen K. Anion−π Interactions with Fluoroarenes. Chem. Rev. 2015, 115, 8867–8895. 10.1021/acs.chemrev.5b00156. 26278927

[ref60] Carreño M. C.; Ruano J. L. G.; Sanz G.; Toledo M. A.; Urbano A. Mild and Regiospecific Nuclear Iodination of Methoxybenzenes and Naphthalenes with *N*-Iodosuccinimide in Acetonitrile. Tetrahedron Lett. 1996, 37, 4081–4084. 10.1016/0040-4039(96)00738-1.

[ref61] Bergström M.; Suresh G.; Naidu V. R.; Unelius C. R. *N*-Iodosuccinimide (NIS) in Direct Aromatic Iodination. Eur. J. Org. Chem. 2017, 2017, 3234–3239. 10.1002/ejoc.201700173.

[ref62] Zhou C.-Y.; Li J.; Peddibhotla S.; Romo D. Mild Arming and Derivatization of Natural Products via an In(OTf)_3_-catalyzed Arene Iodination. Org. Lett. 2010, 12, 2104–2107. 10.1021/ol100587j. 20387852

[ref63] Lista L.; Pezzella A.; Napolitano A.; d’Ischia M. Mild and efficient iodination of aromatic and heterocyclic compounds with the NaClO_2_/NaI/HCl system. Tetrahedron 2008, 64, 234–239. 10.1016/j.tet.2007.10.062.

[ref64] Leitemberger A.; Böhs L. M. C.; Rosa C. H.; Da Silva C.; Galetto F. Z.; Godoi M. Synthesis of Symmetrical Diorganyl Disulfides Employing WEB as an Eco-friendly Oxidative System. ChemistrySelect 2019, 4, 7686–7690. 10.1002/slct.201901385.

[ref65] Teverovskiy G.; Surry D. S.; Buchwald S. L. Pd-Catalyzed Synthesis of Ar–SCF_3_ Compounds under Mild Conditions. Angew. Chem., Int. Ed. 2011, 50, 7312–7314. 10.1002/anie.201102543. PMC3395331 21692157

