# Supplementary material for: One-Pot Synthesis of N-Iodo Sulfoximines from Sulfides
Source: J Org Chem. 2021 Mar 25;86(8):5991–6000. doi: 10.1021/acs.joc.1c00292 (PMC8154609; doi:10.1021/acs.joc.1c00292)
Supplement: Supplementary file 1 — jo1c00292_si_001.pdf [file jo1c00292_si_001.pdf]

SUPPORTING INFORMATION

One-Pot Synthesis of *N*-Iodo Sulfoximines from Sulfides

Anže Zupanc and Marjan Jereb*

University of Ljubljana, Faculty of Chemistry and Chemical Technology, Večna pot 113, 1000 Ljubljana, Slovenia

Table of contents

1. Photos of products 2

2. Copies of NMR spectra 3

^{1}H, $^{13}C\{^{1}H\}$ and ^{19}F NMR spectra of *N*-iodo sulfoximines **2** 4

^{1}H and $^{13}C\{^{1}H\}$ NMR spectra of *N*-bromo and *N*-chloro sulfoximines **3** and **4** 59

^{1}H and $^{13}C\{^{1}H\}$ NMR spectra of iodinated and oxidized products **6**, **8**, **10**, **12** and **14** 63

^{1}H, $^{13}C\{^{1}H\}$ and ^{19}F NMR spectra of *N*-(trifluoromethanesulfenyl) sulfoximines **15** 73

^{1}H NMR spectra of **2a** prepared by scale-up procedures 85

1. Photos of products

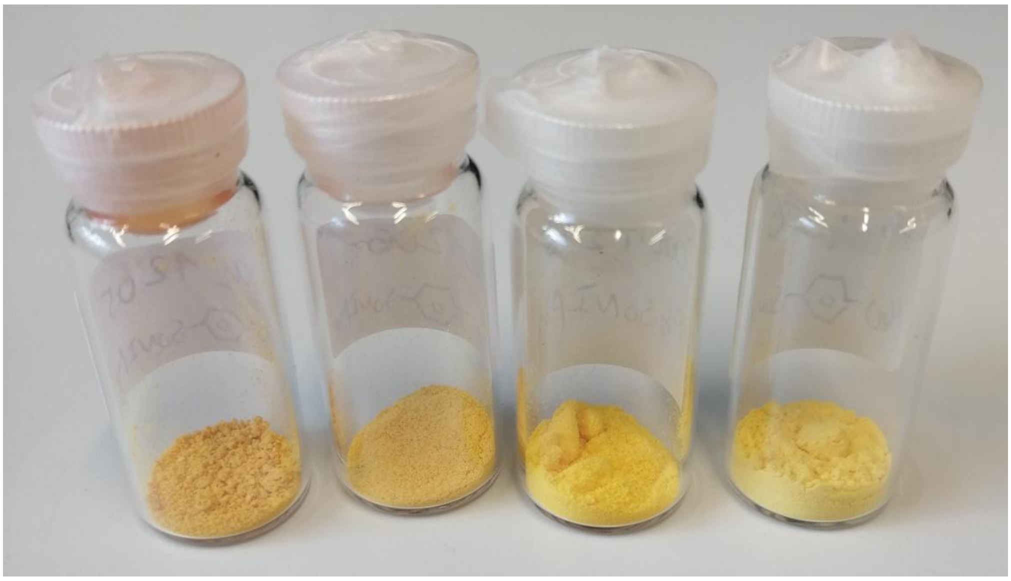

Figure S1. *N*-Iodo sulfoximines **2m**, **2l**, **2t**, and **2g** (from left to right).

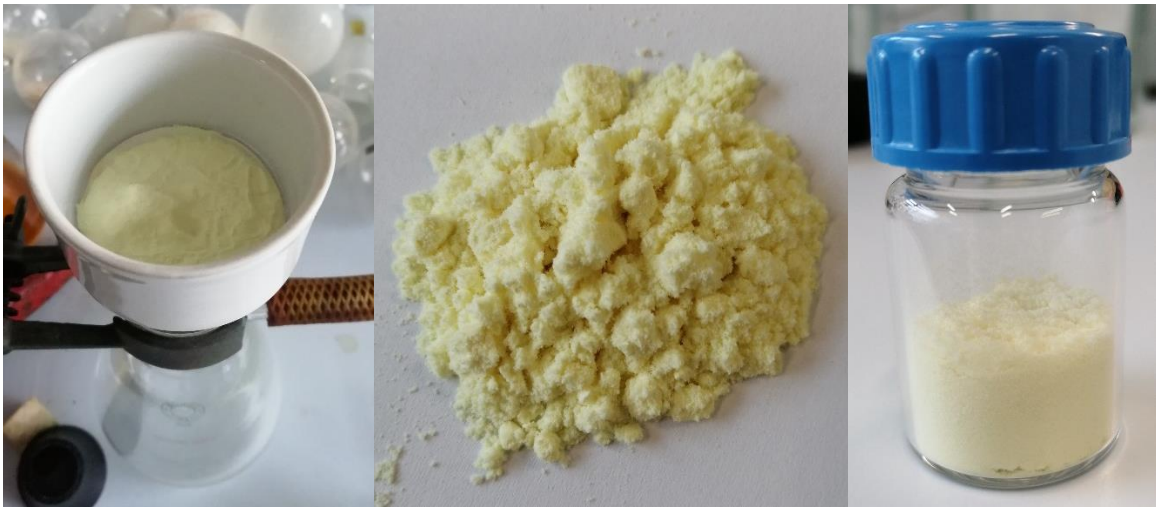

Figure S2. Product **2a** prepared with NIS on 10 mmol scale.

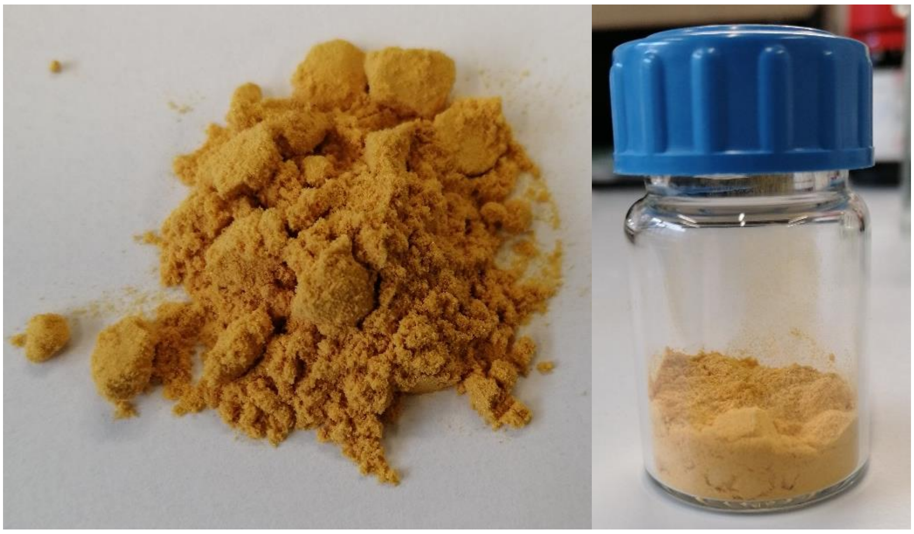

Figure S3. Product **2a** prepared with I_2 on 10 mmol scale.

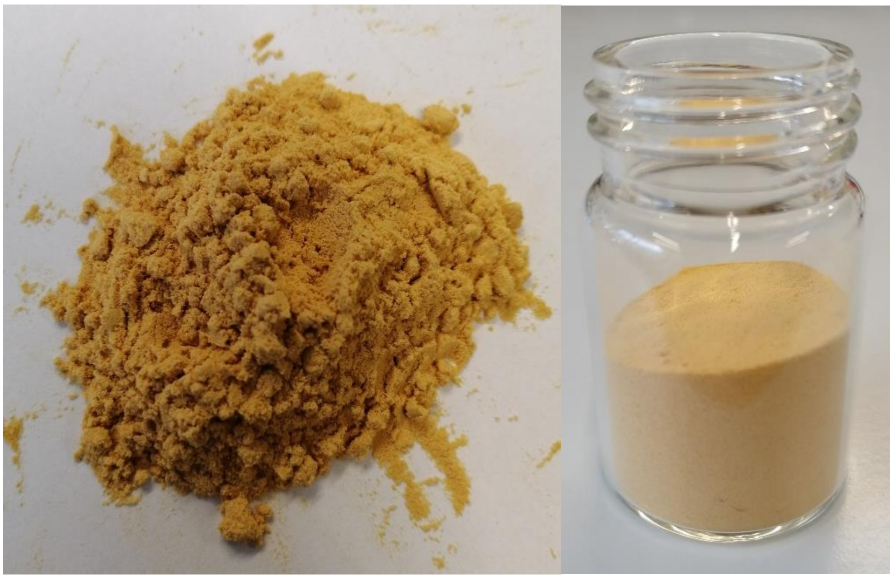

Figure S4. Product **2a** prepared with I_2 on 25 mmol scale.

2. Copies of NMR spectra

^{1}H, $^{13}C\{^{1}H\}$ and ^{19}F NMR spectra of *N*-iodo sulfoximines **2**

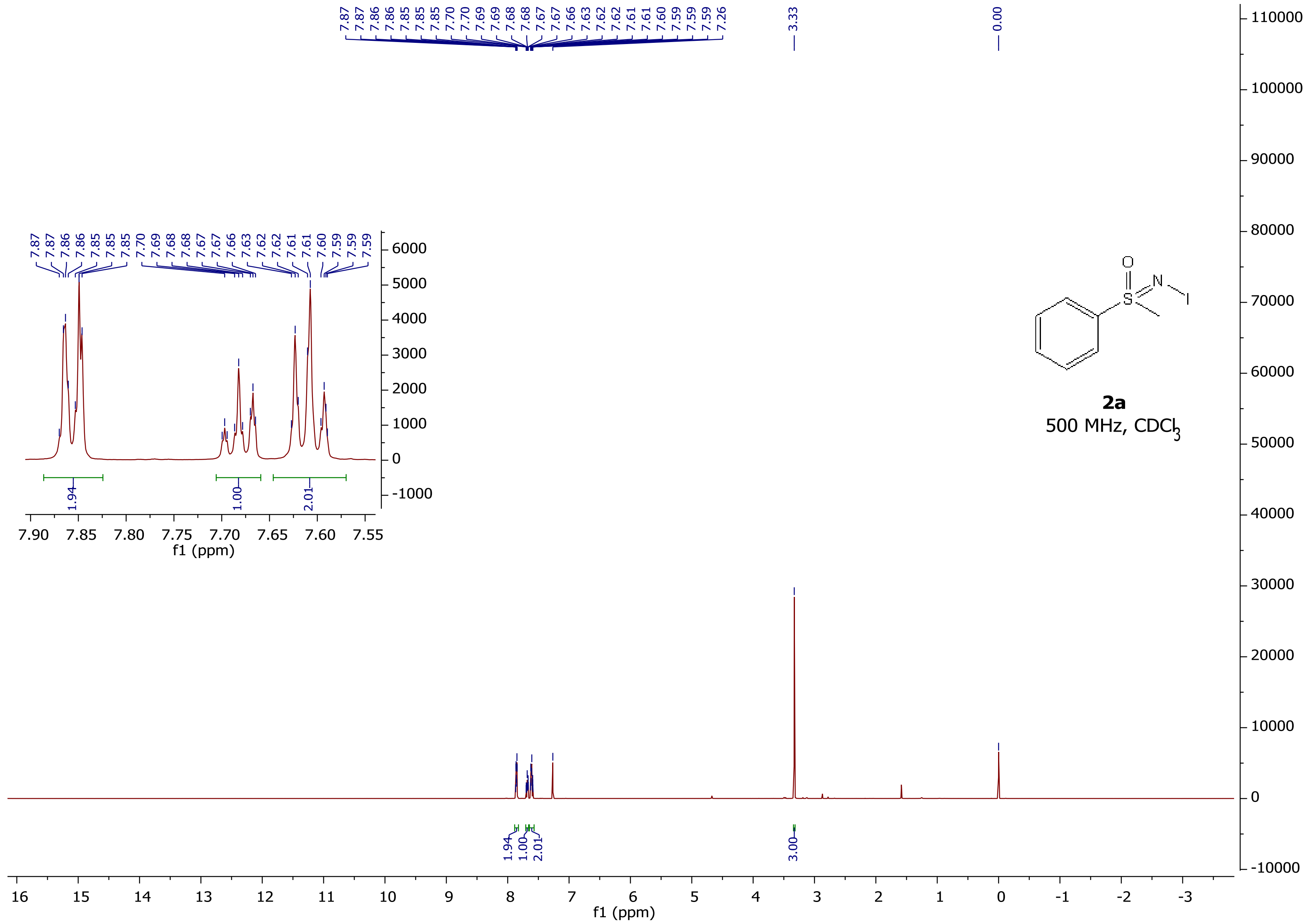

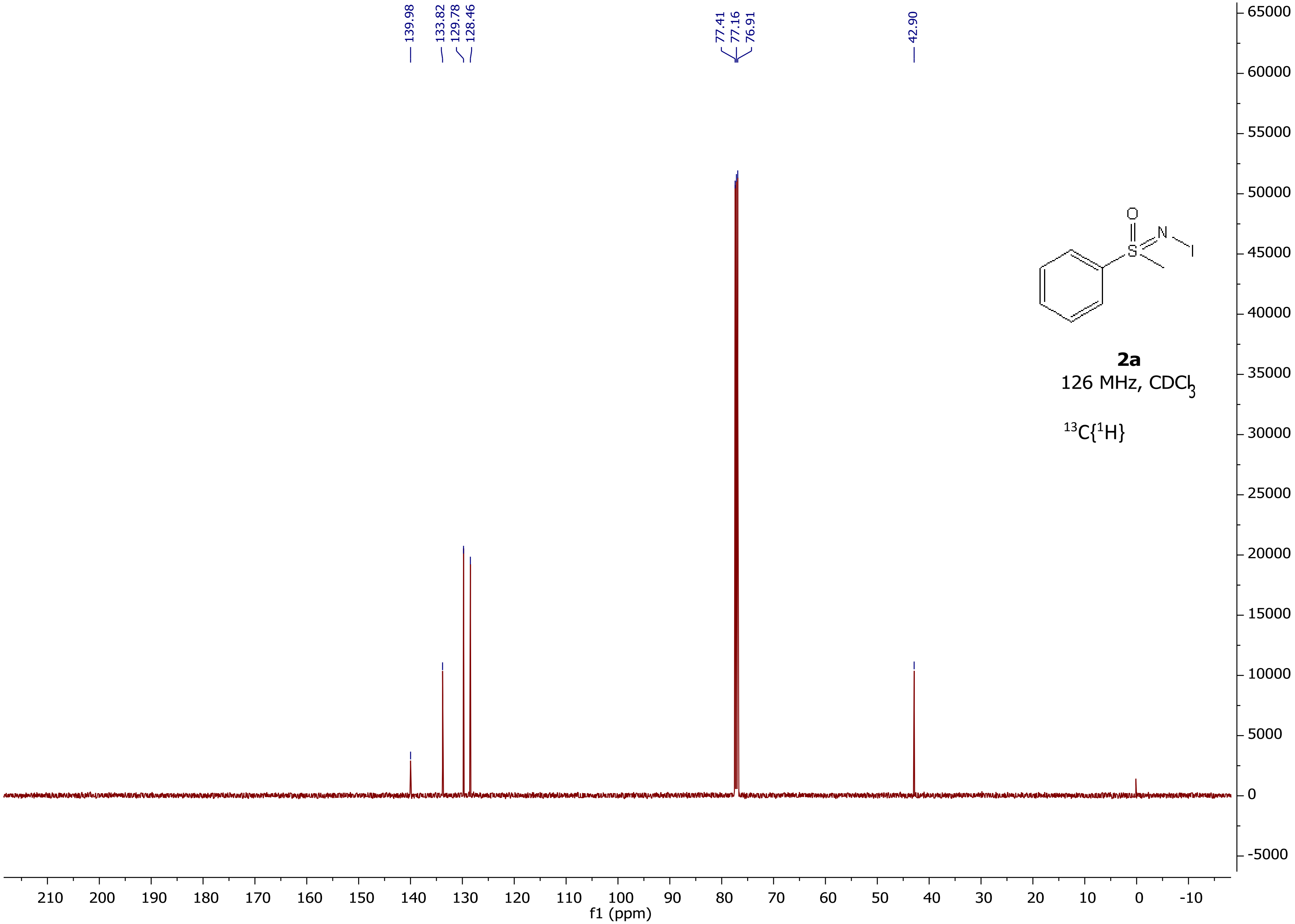
139.98
133.82
129.78
128.46
77.41
77.16
76.91
42.90
O
N
S
I
2a
126 MHz, CDCl3
13C{1H}
f1 (ppm)

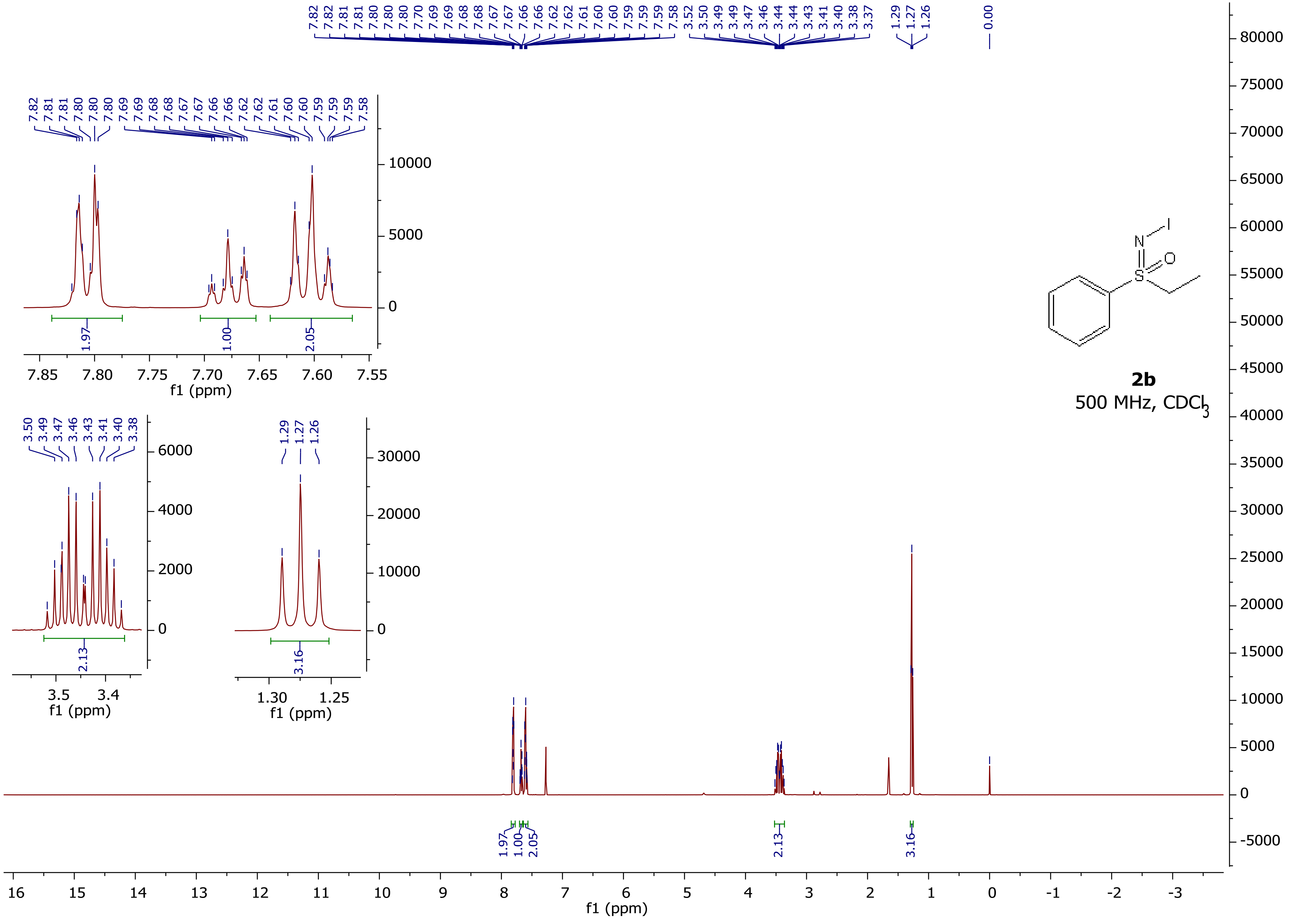
2b
500 MHz, CDCl3
f1 (ppm)

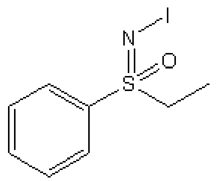

2b
126 MHz, $CDCl_3$

$^{13}C\{^{1}H\}$

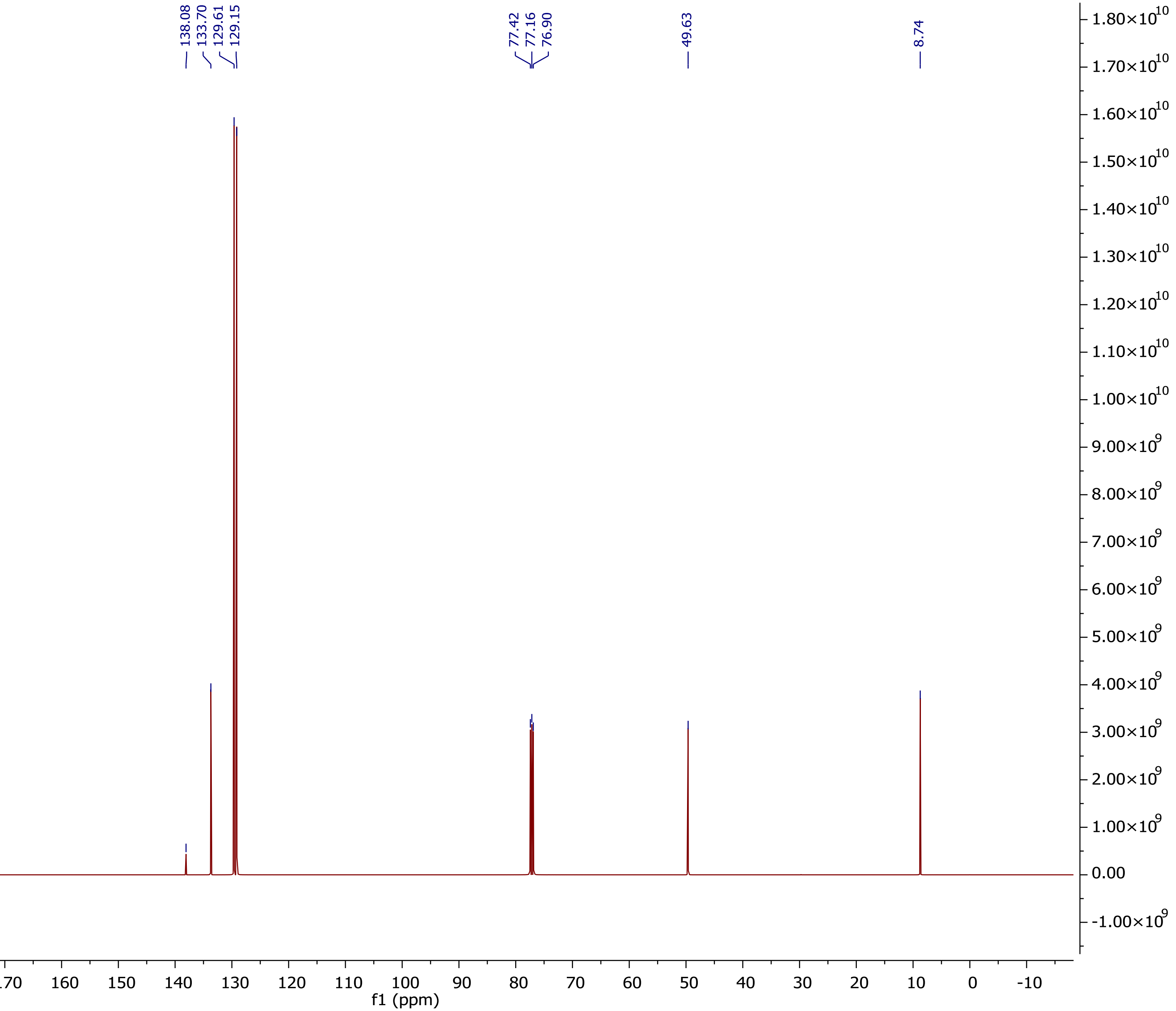

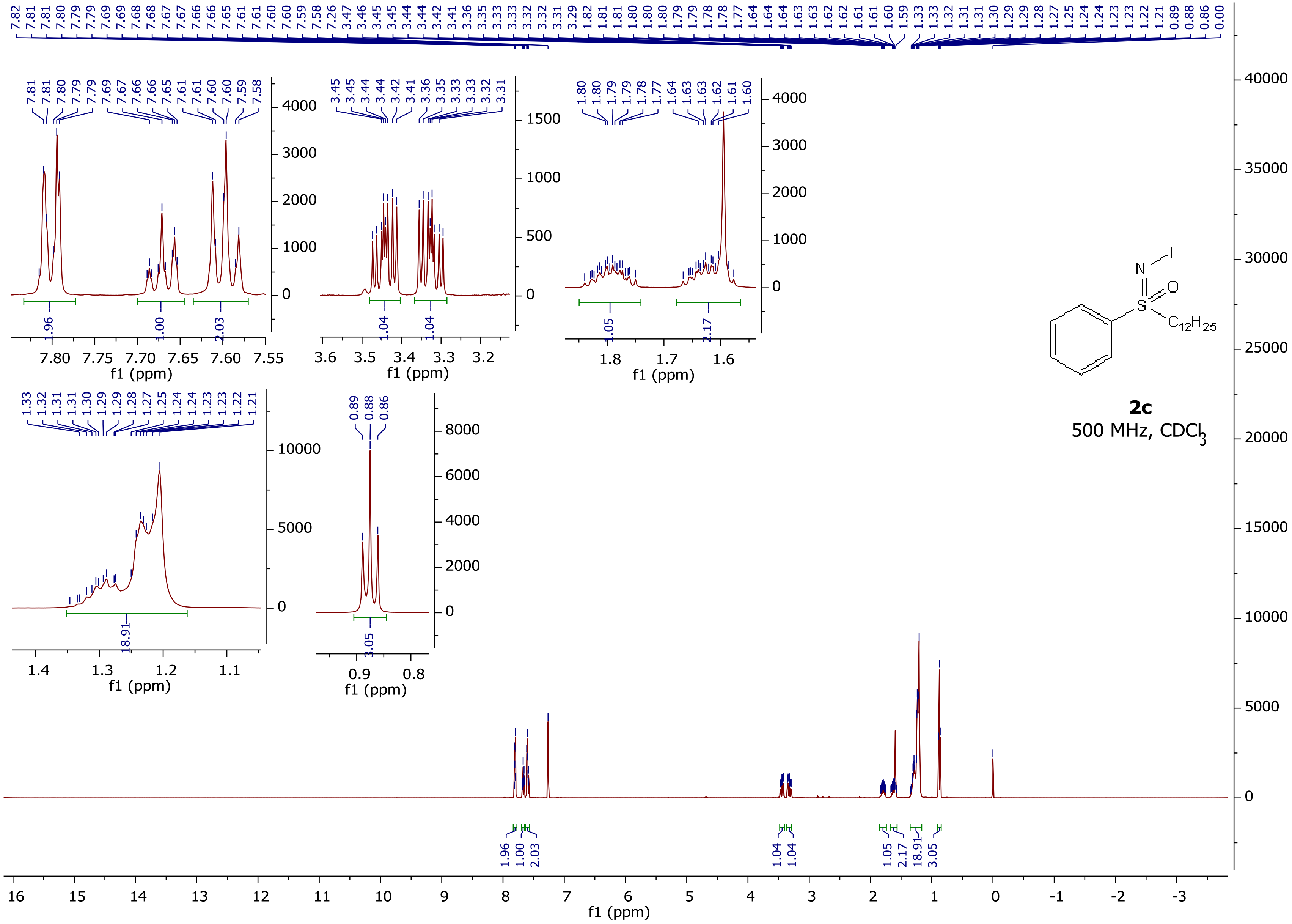

2c
500 MHz, CDCl3
f1 (ppm)

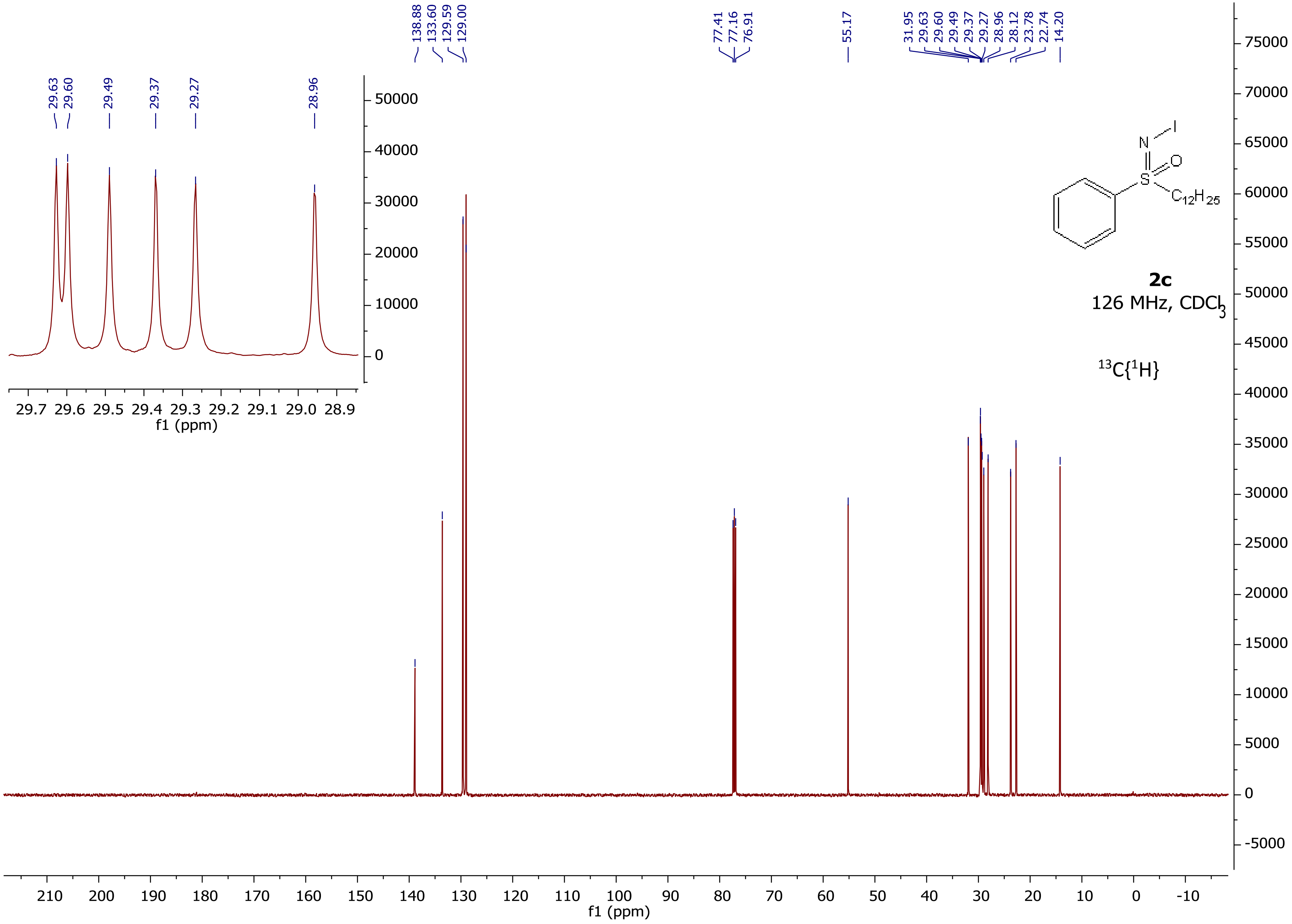

138.88
133.60
129.59
129.00
77.41
77.16
76.91
55.17
31.95
29.63
29.60
29.49
29.37
29.27
28.96
28.12
23.78
22.74
14.20
2c
126 MHz, CDCl3
13C{1H}
f1 (ppm)

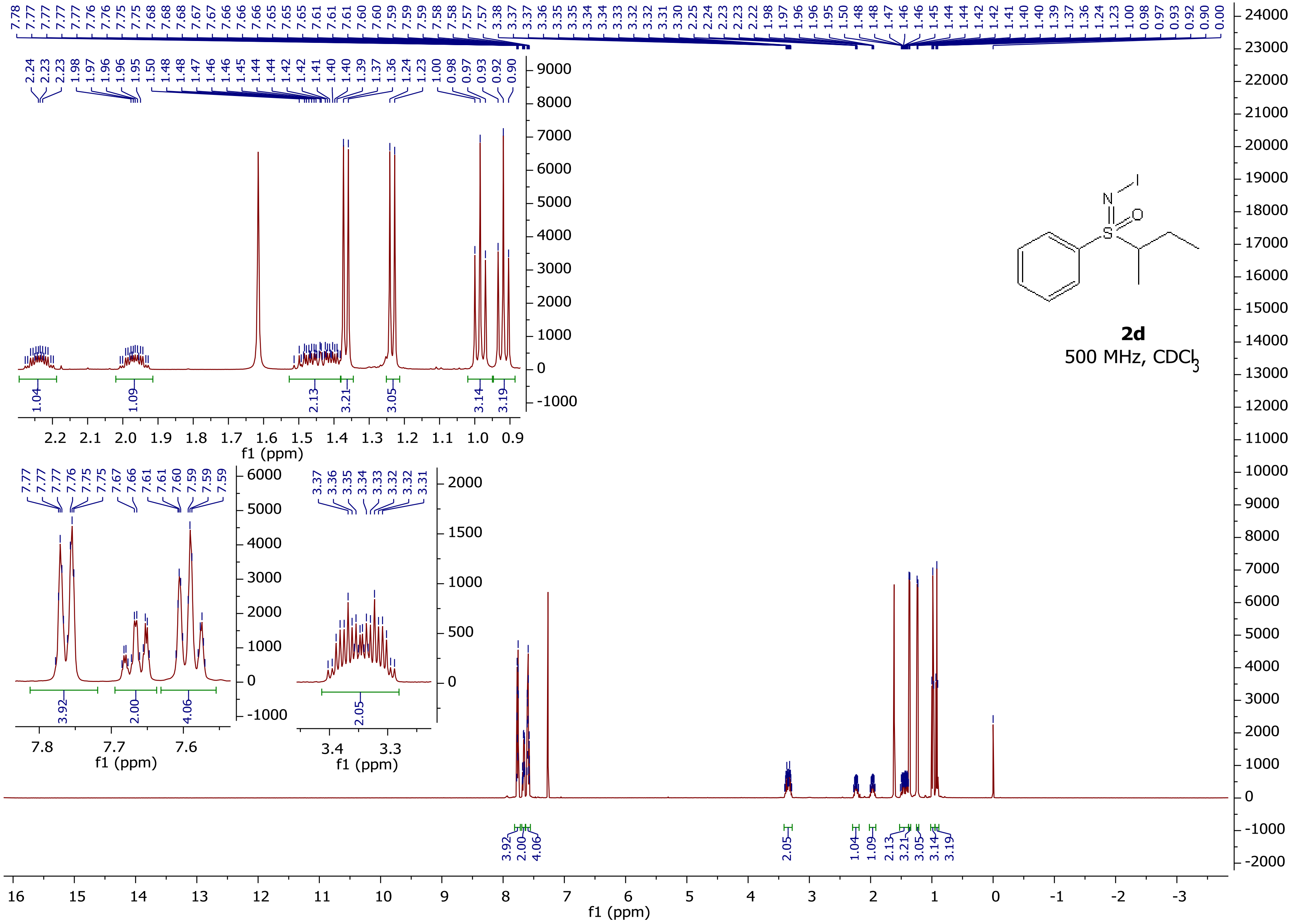
2d
500 MHz, CDCl3
f1 (ppm)

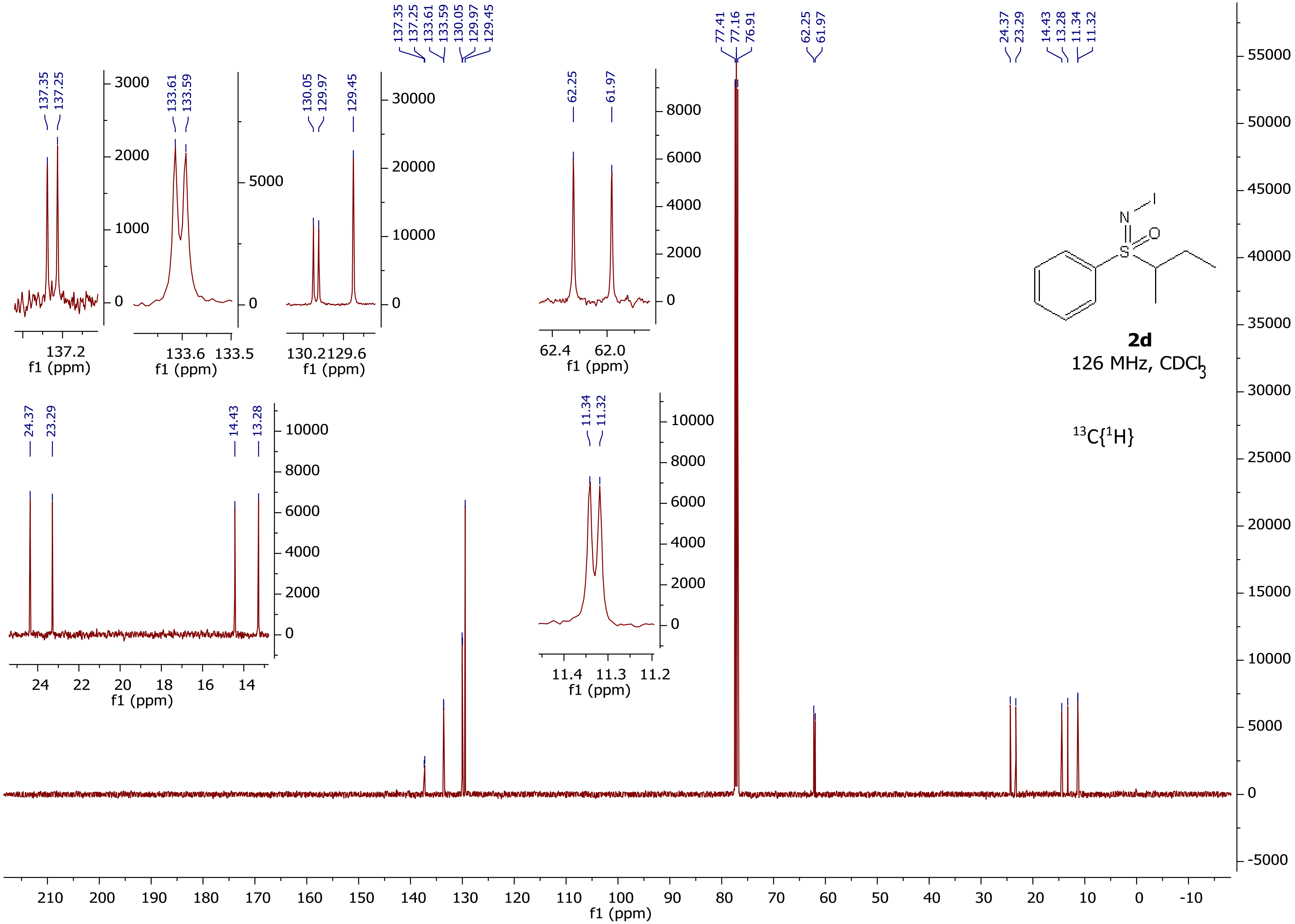
2d
126 MHz, CDCl3
13C{1H}
137.35
137.25
133.61
133.59
130.05
129.97
129.45
77.41
77.16
76.91
62.25
61.97
24.37
23.29
14.43
13.28
11.34
11.32
f1 (ppm)

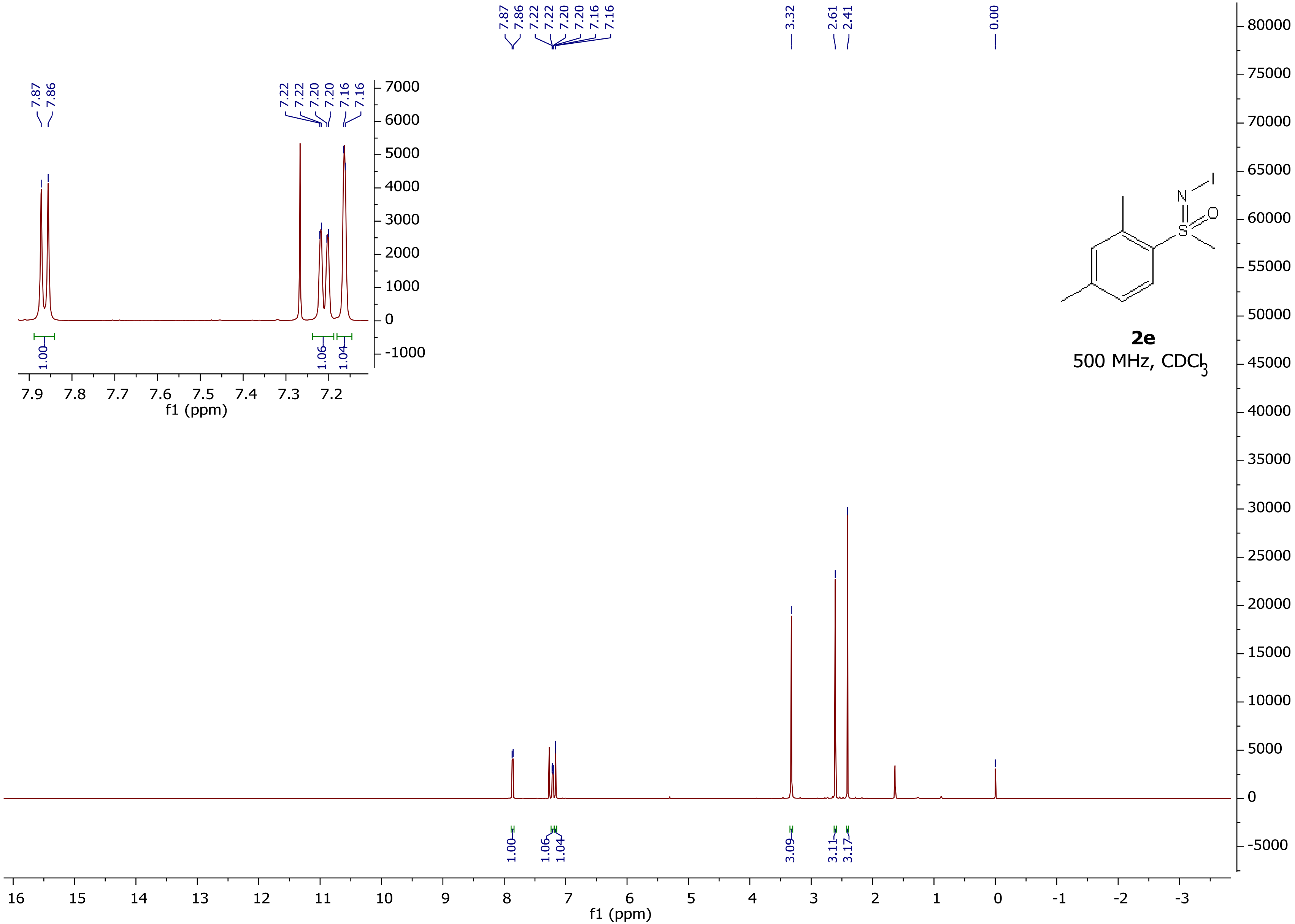
7.87
7.86
7.22
7.22
7.20
7.20
7.16
7.16
3.32
2.61
2.41
0.00
1.00
1.06
1.04
3.09
3.11
3.17
f1 (ppm)
2e
500 MHz, CDCl3

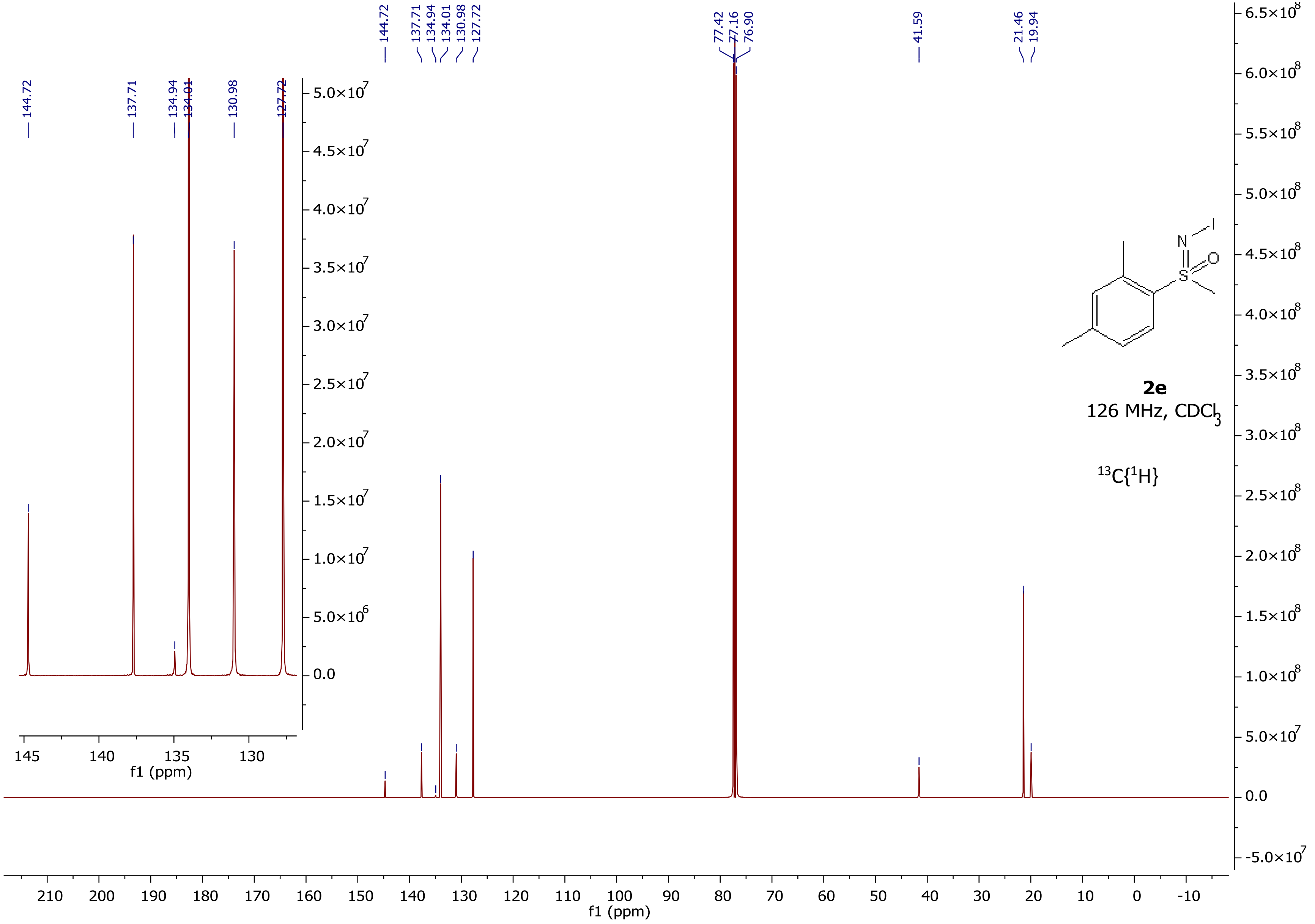
144.72
137.71
134.94
134.01
130.98
127.72
77.42
77.16
76.90
41.59
21.46
19.94
2e
126 MHz, CDCl3
13C{1H}
f1 (ppm)

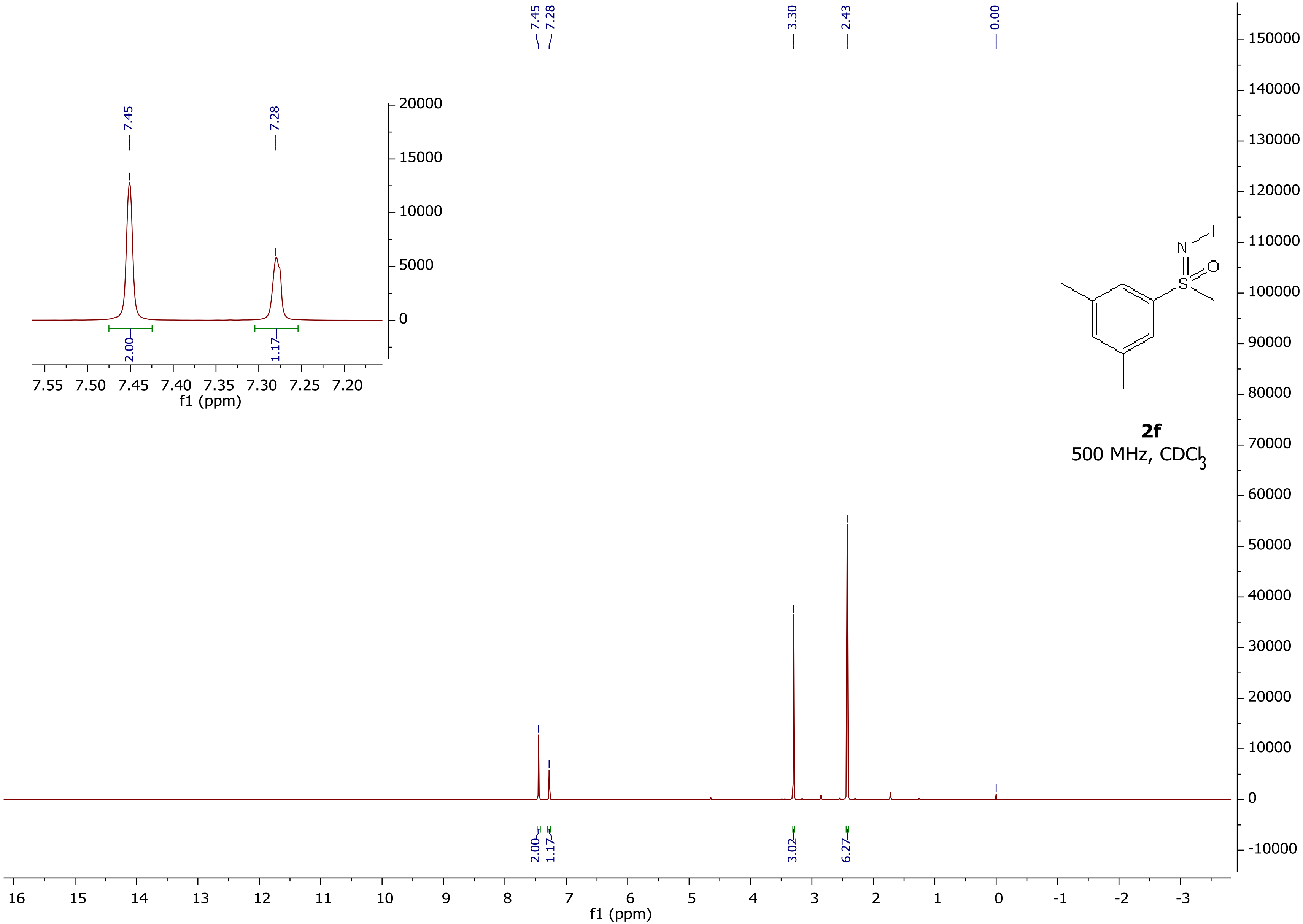
7.45
7.28
3.30
2.43
0.00
2.00
1.17
3.02
6.27
f1 (ppm)
2f
500 MHz, CDCl3

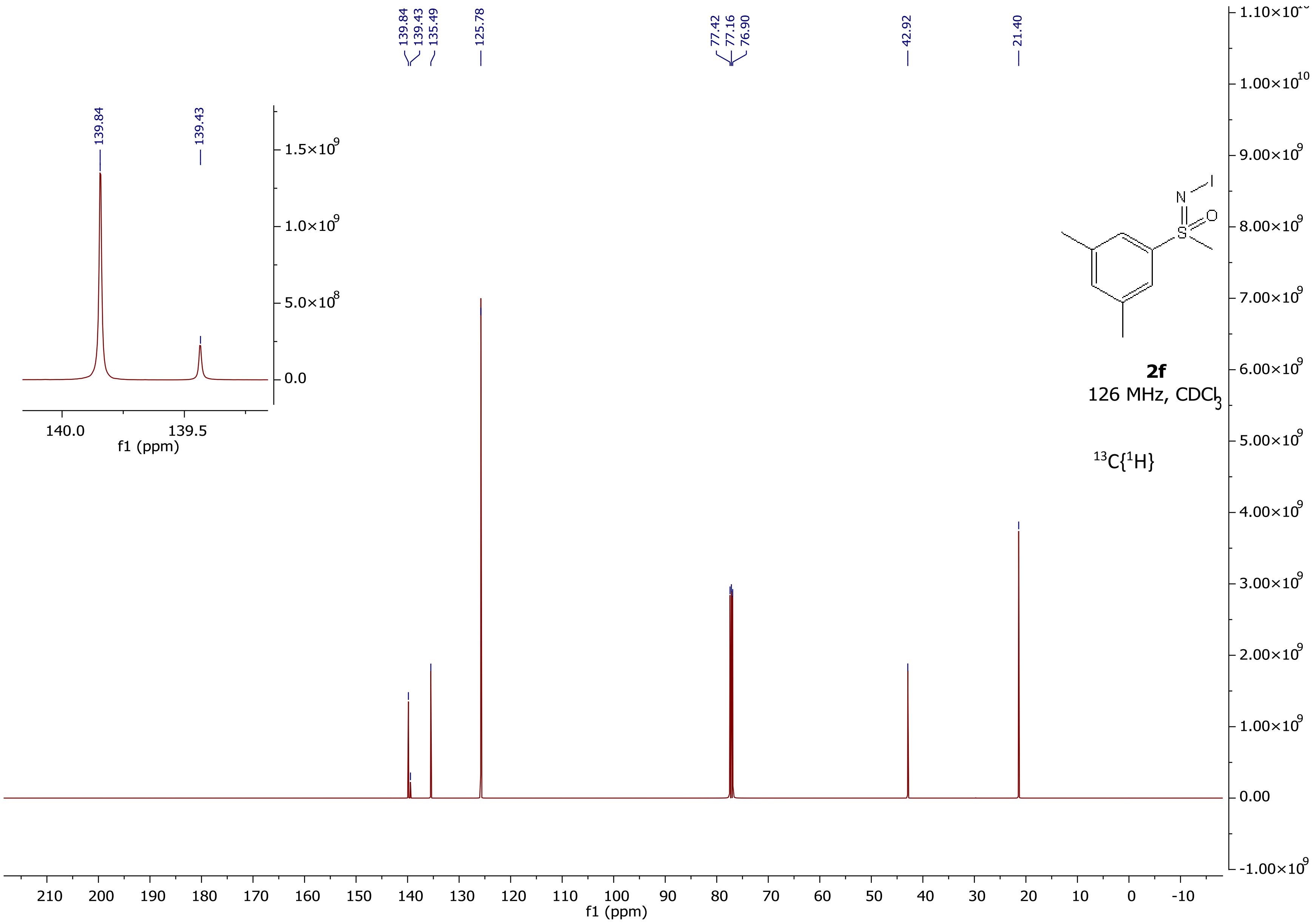

139.84
139.43
135.49
125.78
77.42
77.16
76.90
42.92
21.40
2f
126 MHz, CDCl3
13C{1H}
f1 (ppm)

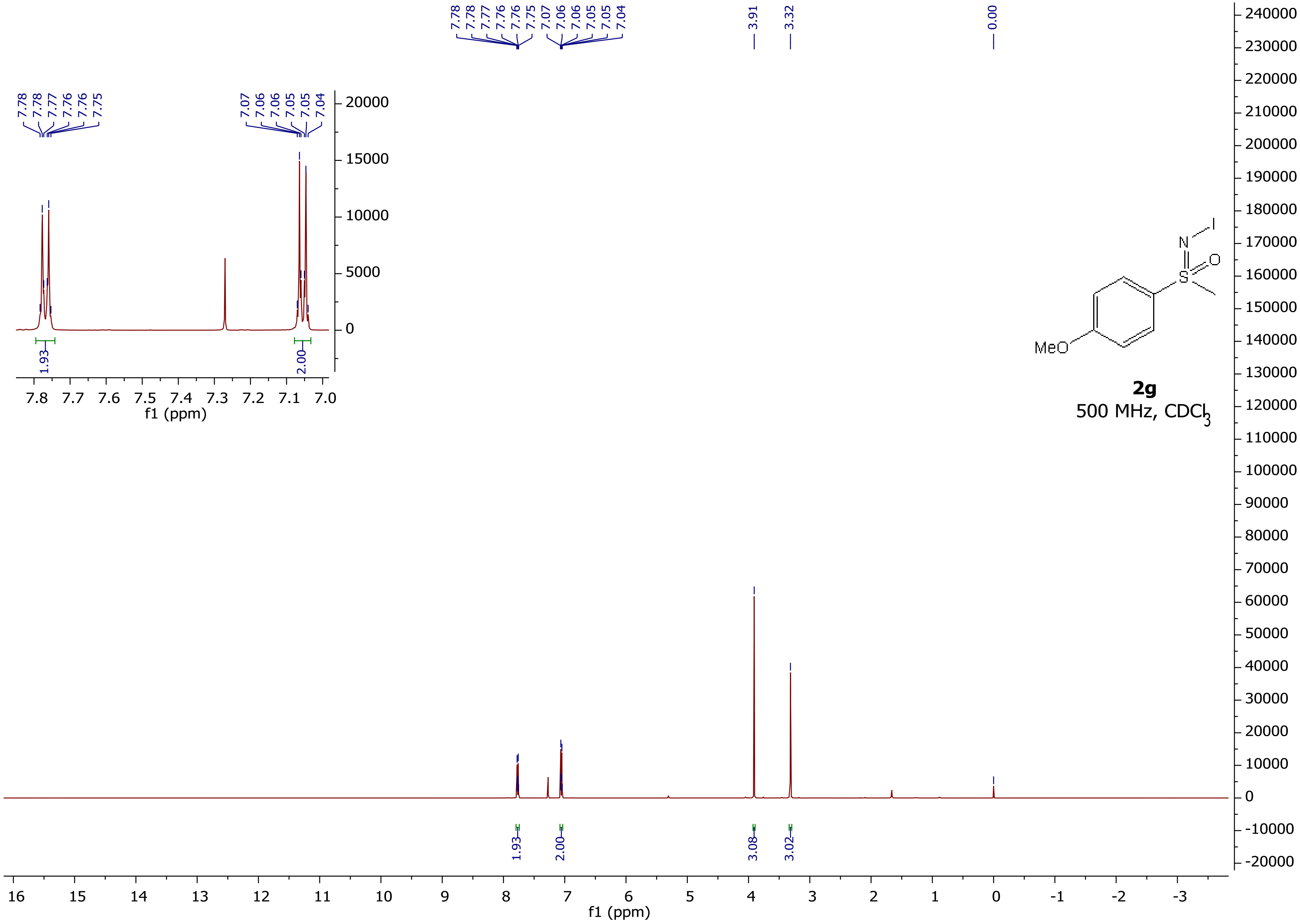
7.78
7.78
7.77
7.76
7.76
7.75
7.07
7.06
7.06
7.05
7.05
7.04
3.91
3.32
0.00
1.93
2.00
3.08
3.02
f1 (ppm)
2g
500 MHz, CDCl3
MeO
N
I
S
O

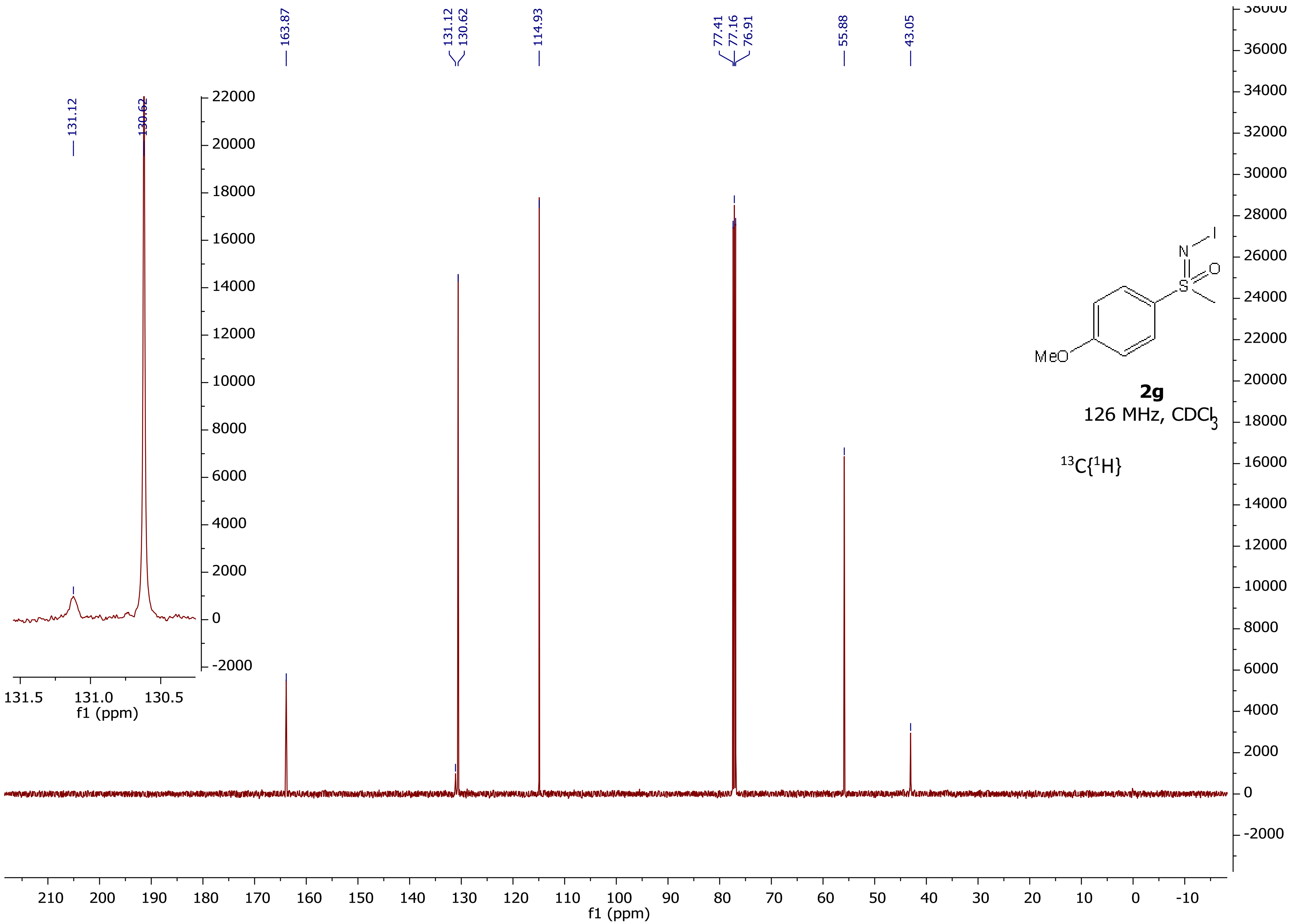

163.87
131.12
130.62
114.93
77.41
77.16
76.91
55.88
43.05
MeO
N
I
S
O
2g
126 MHz, CDCl3
13C{1H}
f1 (ppm)

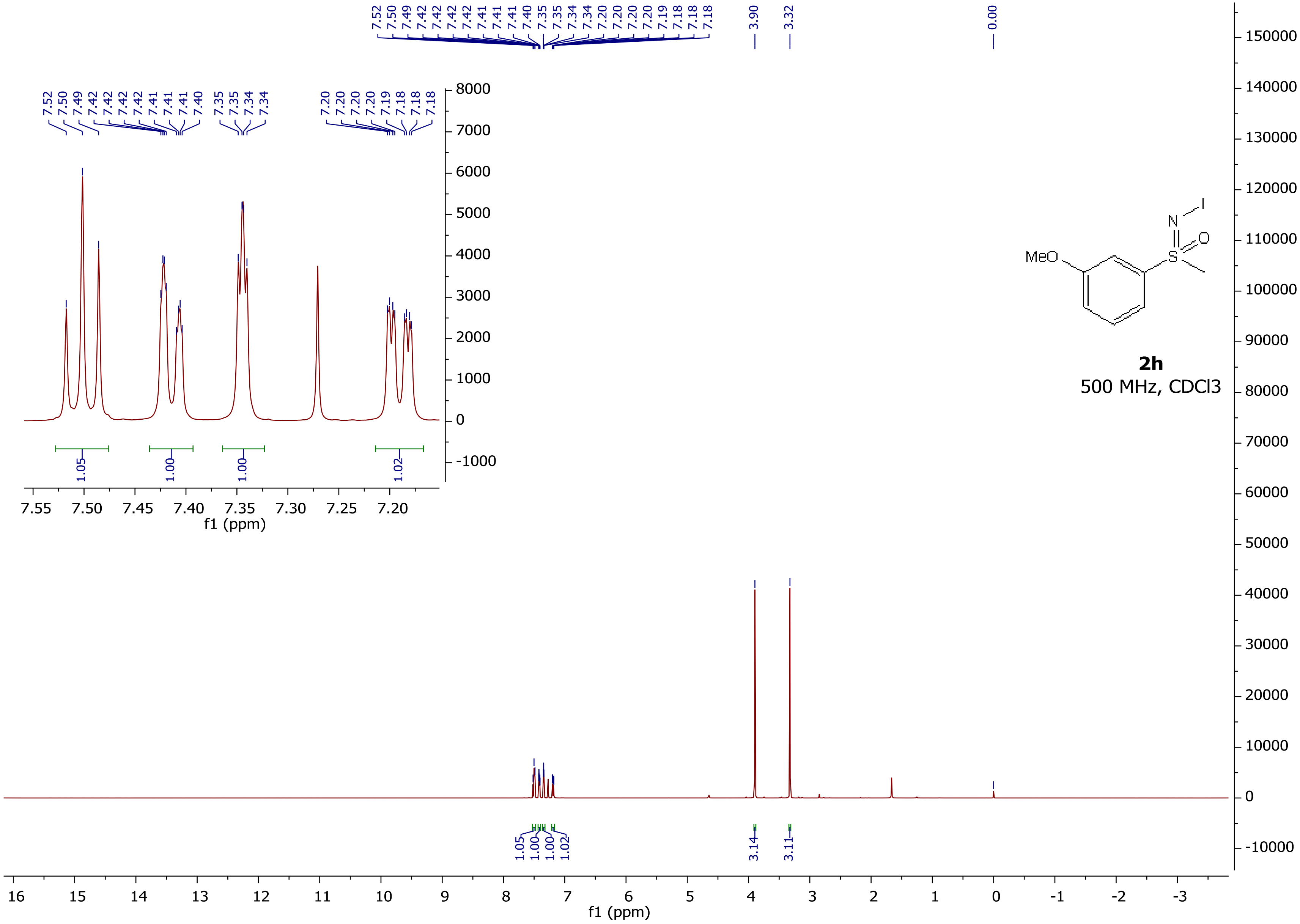

7.52 7.50 7.49 7.42 7.42 7.42 7.42 7.41 7.41 7.41 7.40 7.35 7.35 7.34 7.34 7.20 7.20 7.20 7.20 7.19 7.18 7.18 7.18
3.90
3.32
0.00
MeO
N
I
S
O
2h
500 MHz, CDCl3
1.05
1.00
1.00
1.02
3.14
3.11
f1 (ppm)

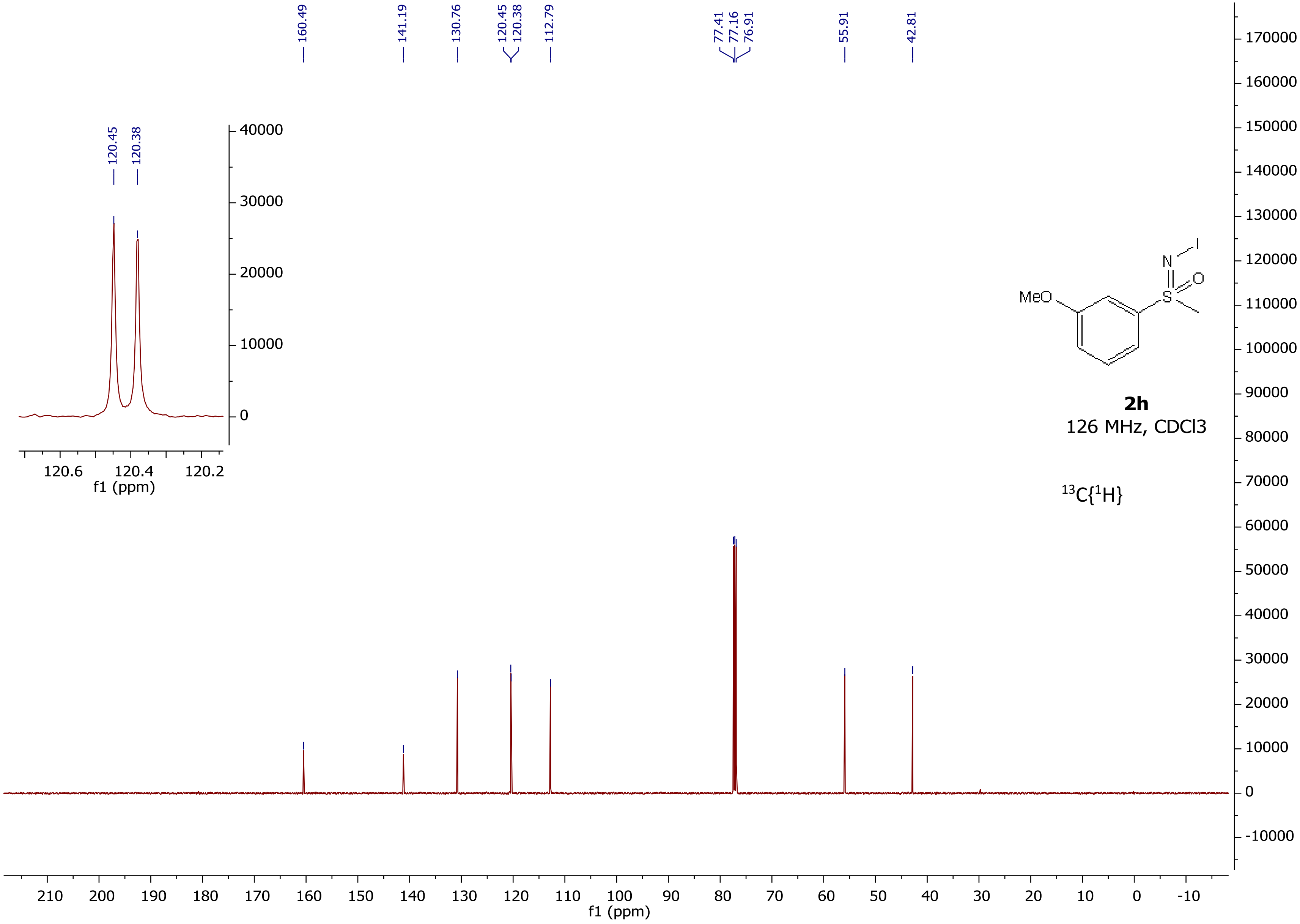
160.49
141.19
130.76
120.45
120.38
112.79
77.41
77.16
76.91
55.91
42.81
MeO
N
I
S
O
2h
126 MHz, CDCl3
$^{13}C\{^{1}H\}$
f1 (ppm)

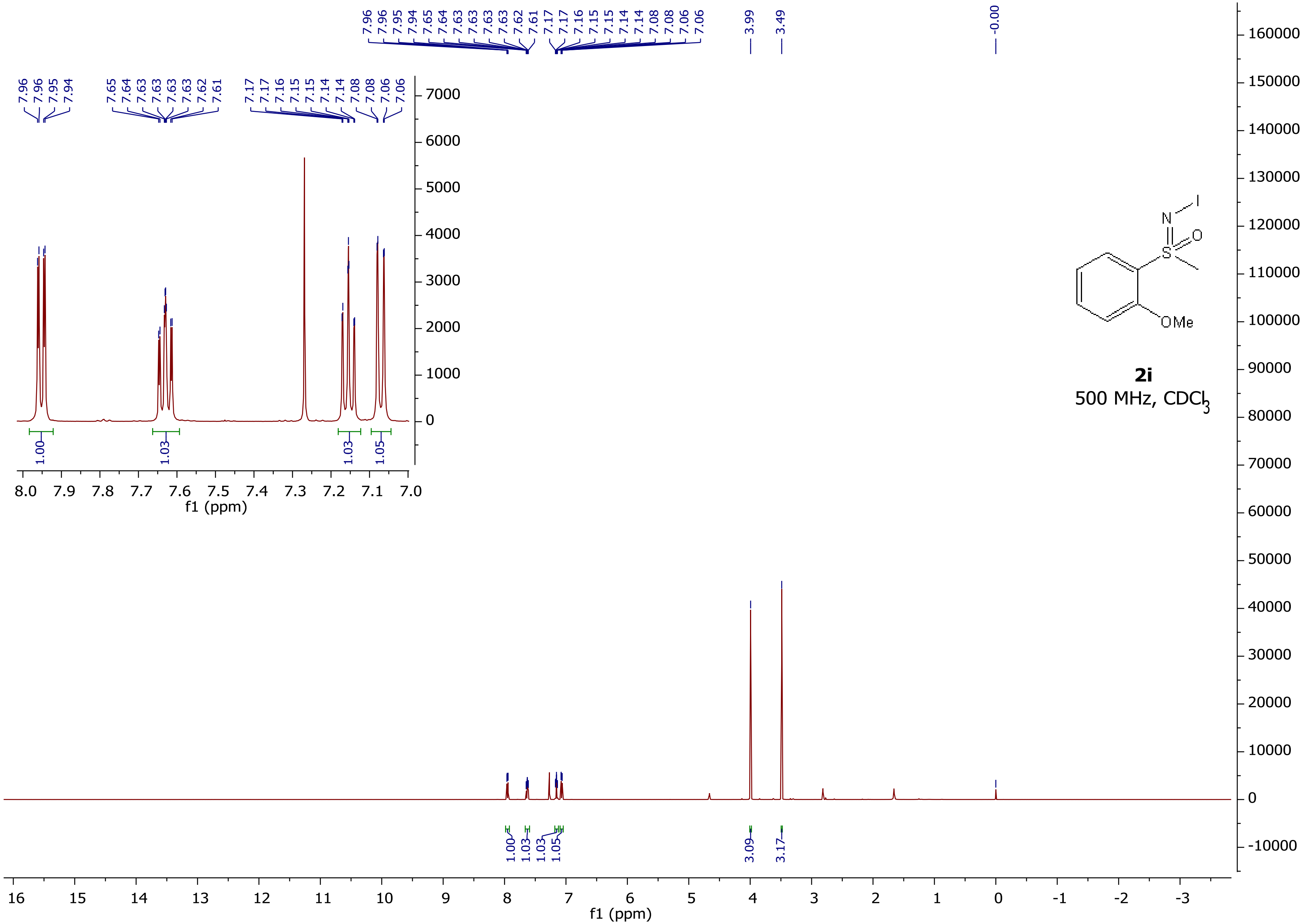

2i
500 MHz, CDCl3
OMe

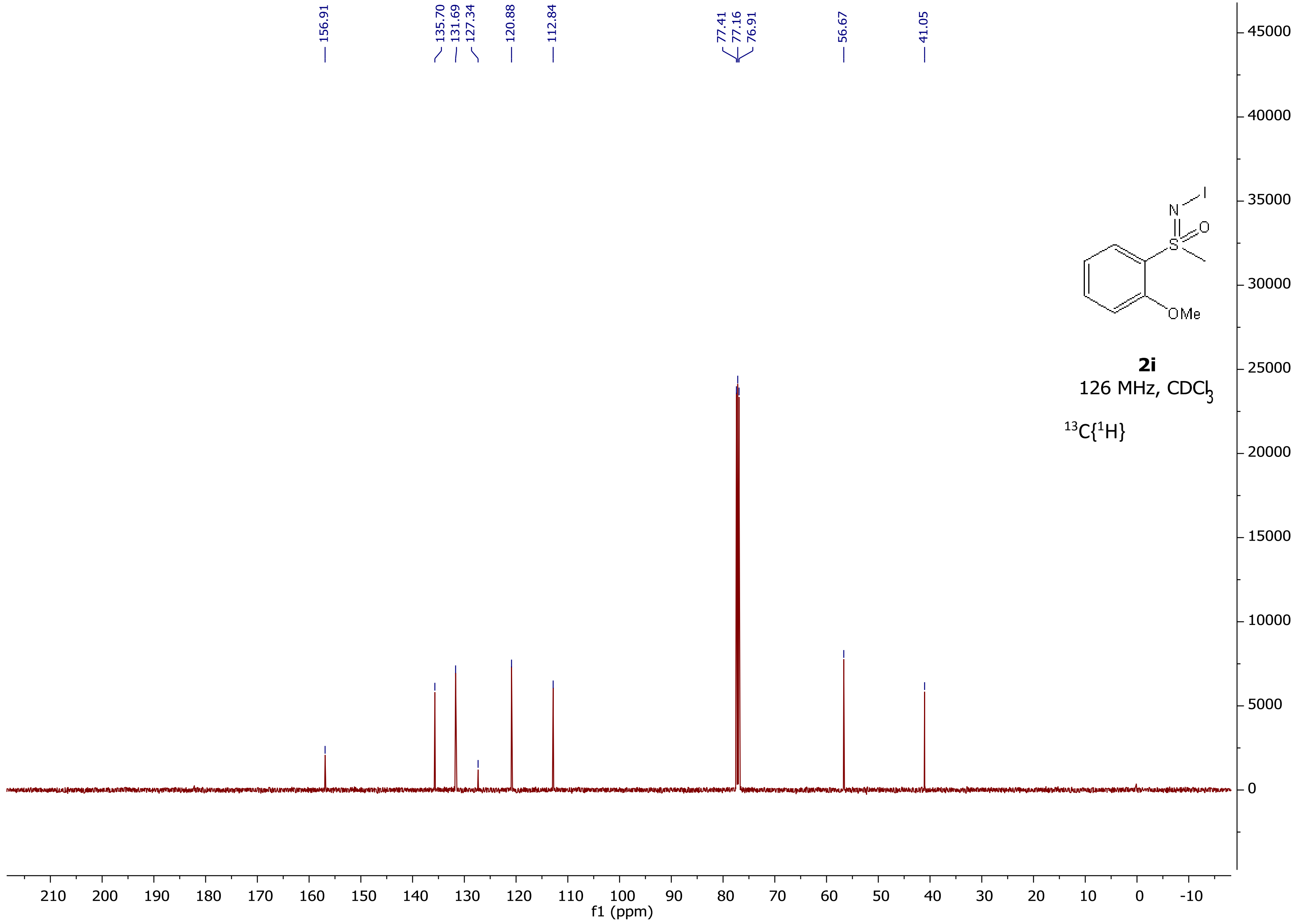
156.91
135.70
131.69
127.34
120.88
112.84
77.41
77.16
76.91
56.67
41.05
2i
126 MHz, CDCl3
13C{1H}
f1 (ppm)

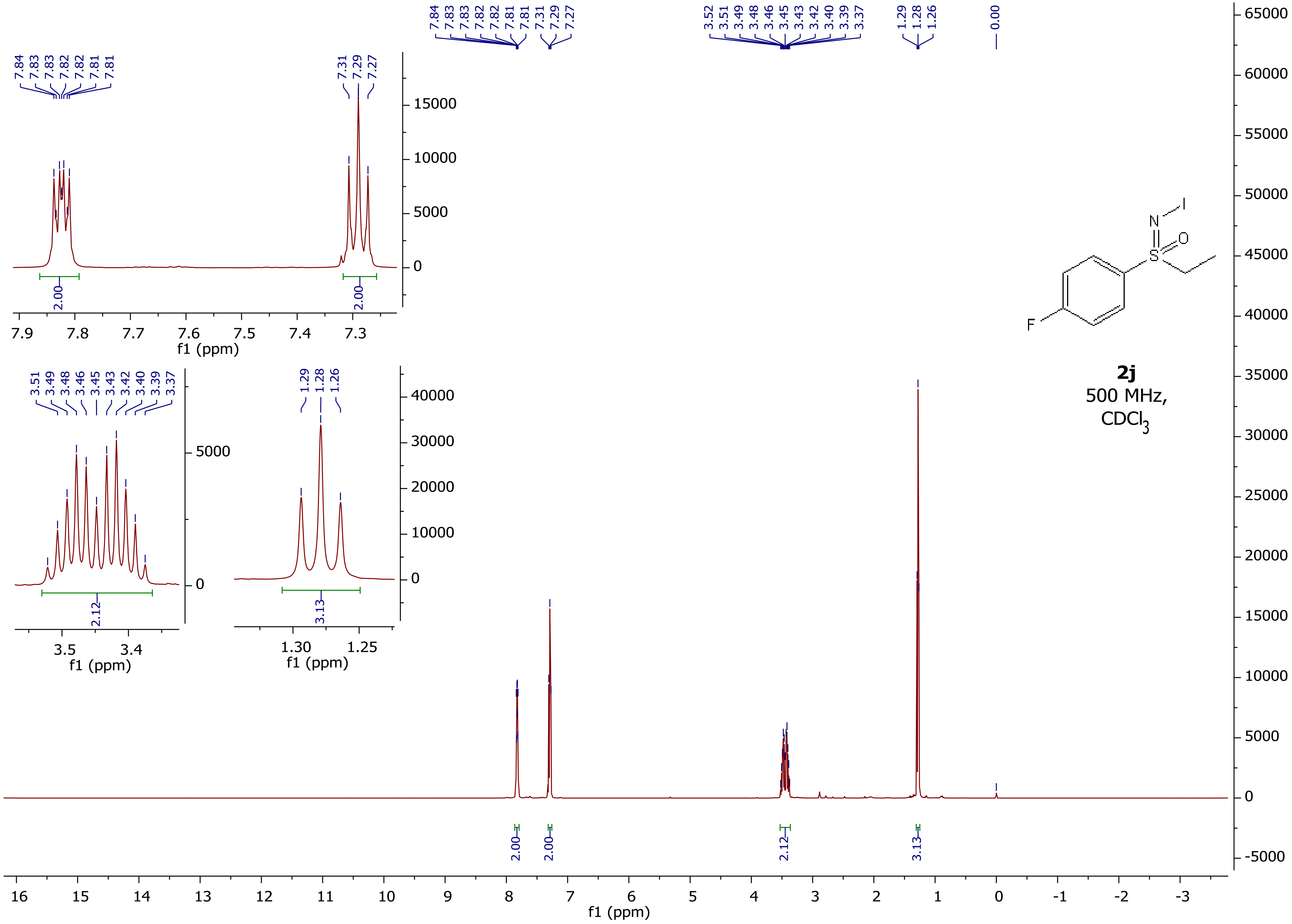

2j
500 MHz,
CDCl3
f1 (ppm)

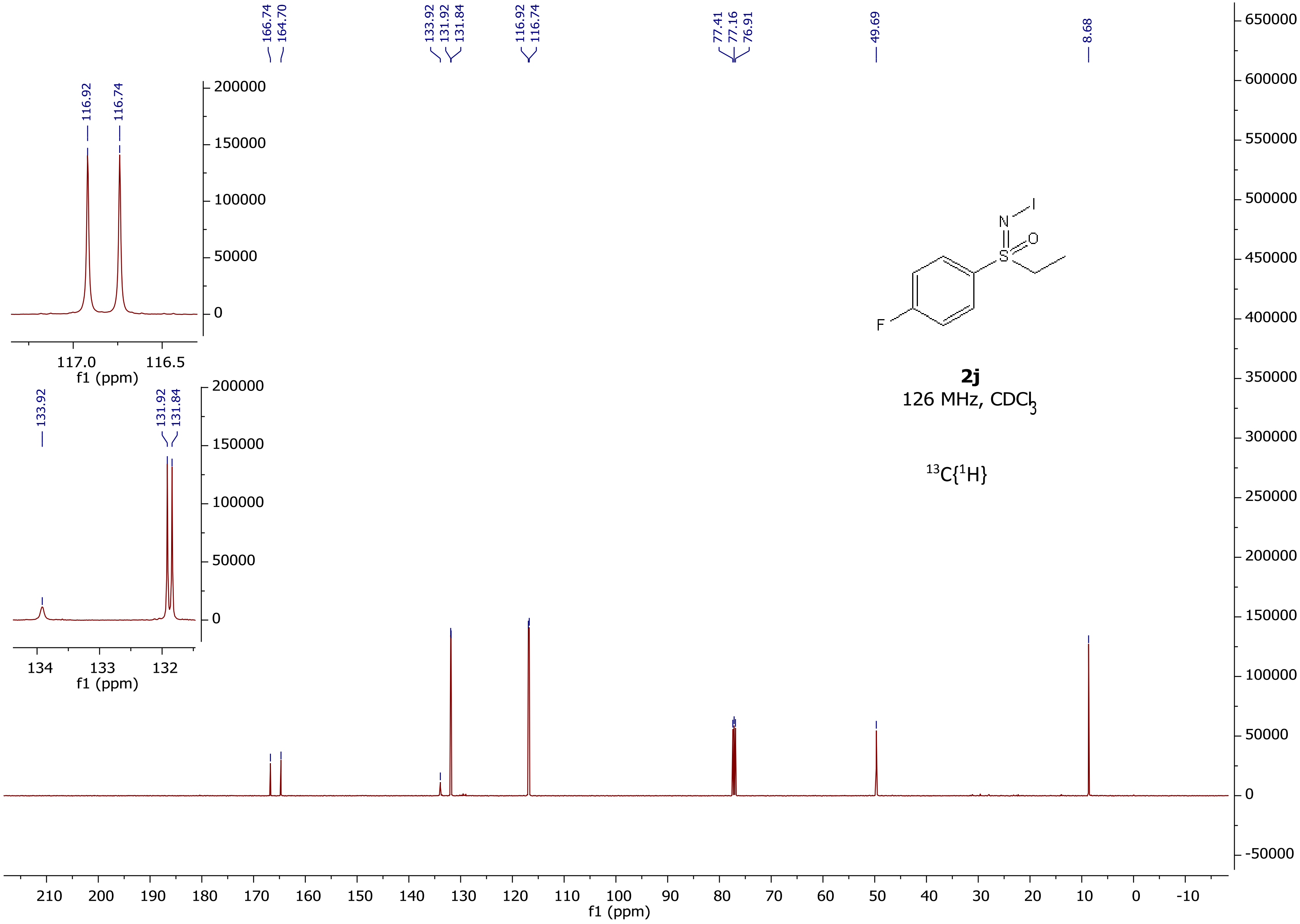

166.74
164.70
133.92
131.92
131.84
116.92
116.74
77.41
77.16
76.91
49.69
8.68
N
I
S
O
F
2j
126 MHz, CDCl3
13C{1H}
f1 (ppm)

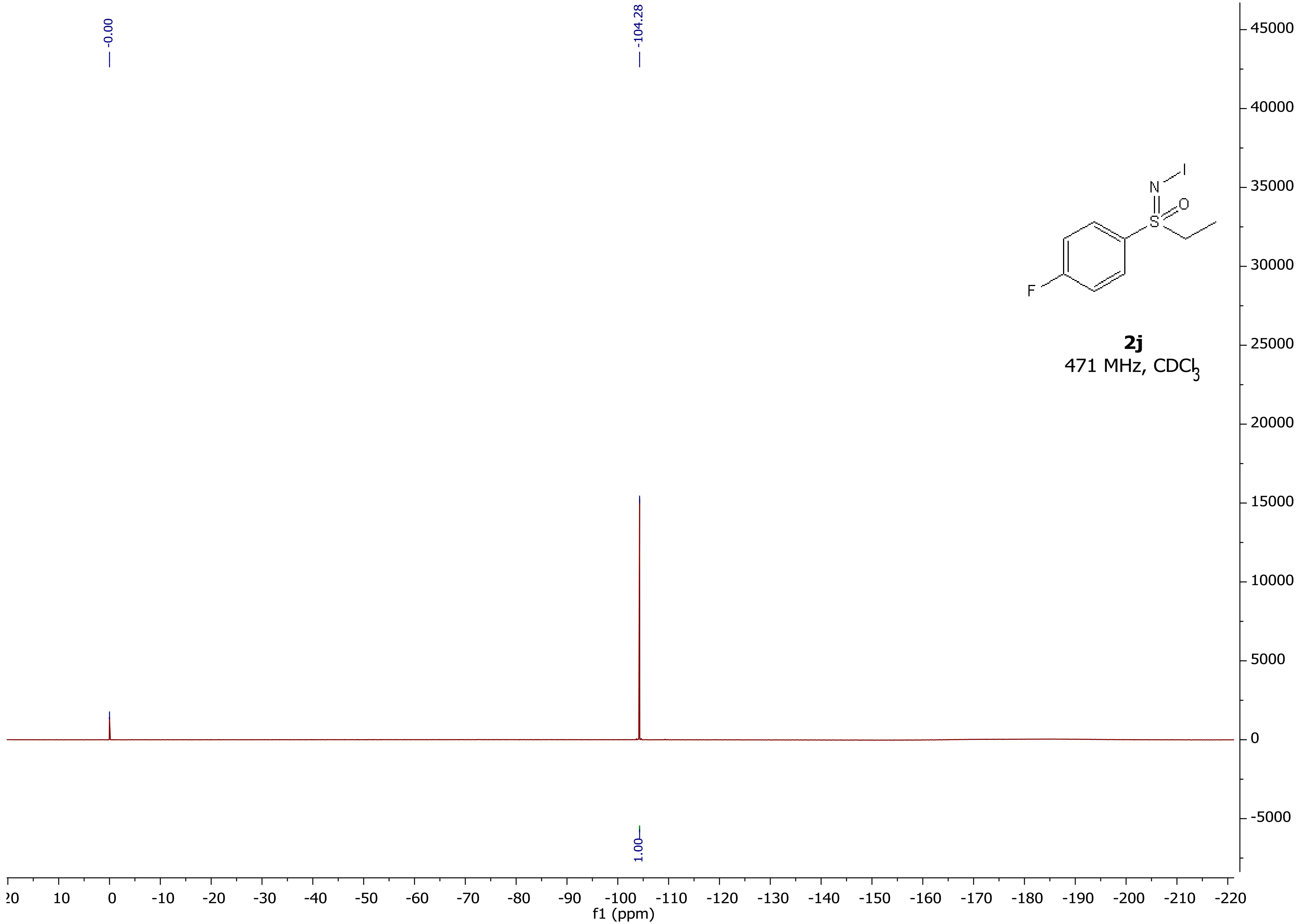
-0.00
-104.28
F
N
I
S
O
2j
471 MHz, CDCl3
1.00
45000
40000
35000
30000
25000
20000
15000
10000
5000
0
-5000
20
10
0
-10
-20
-30
-40
-50
-60
-70
-80
-90
-100
-110
-120
-130
-140
-150
-160
-170
-180
-190
-200
-210
-220
f1 (ppm)

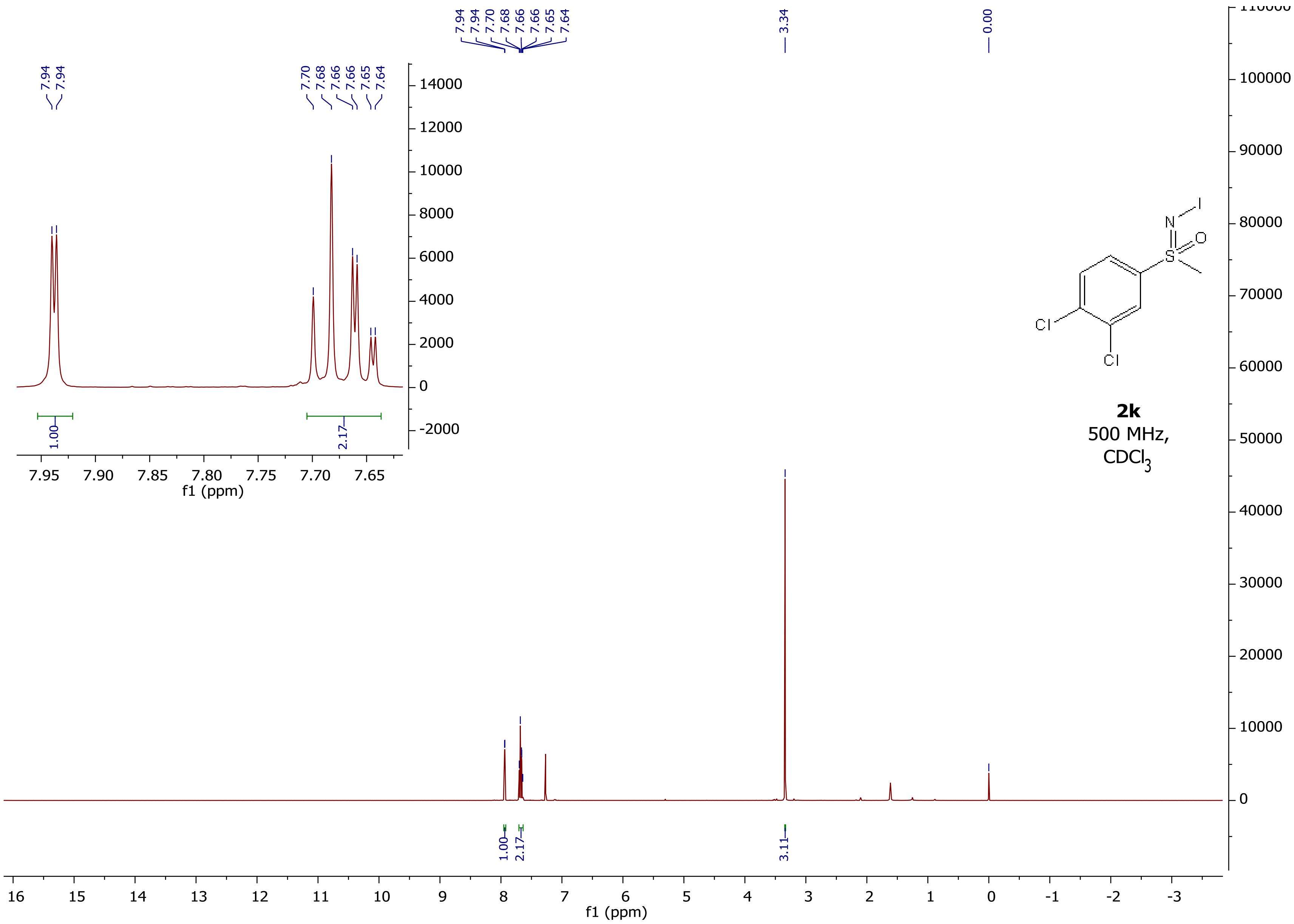
7.94
7.94
7.70
7.68
7.66
7.66
7.65
7.64
3.34
0.00
1.00
2.17
3.11
7.95 7.90 7.85 7.80 7.75 7.70 7.65
f1 (ppm)
16 15 14 13 12 11 10 9 8 7 6 5 4 3 2 1 0 -1 -2 -3
f1 (ppm)
N
I
S
O
Cl
Cl
2k
500 MHz,
CDCl3

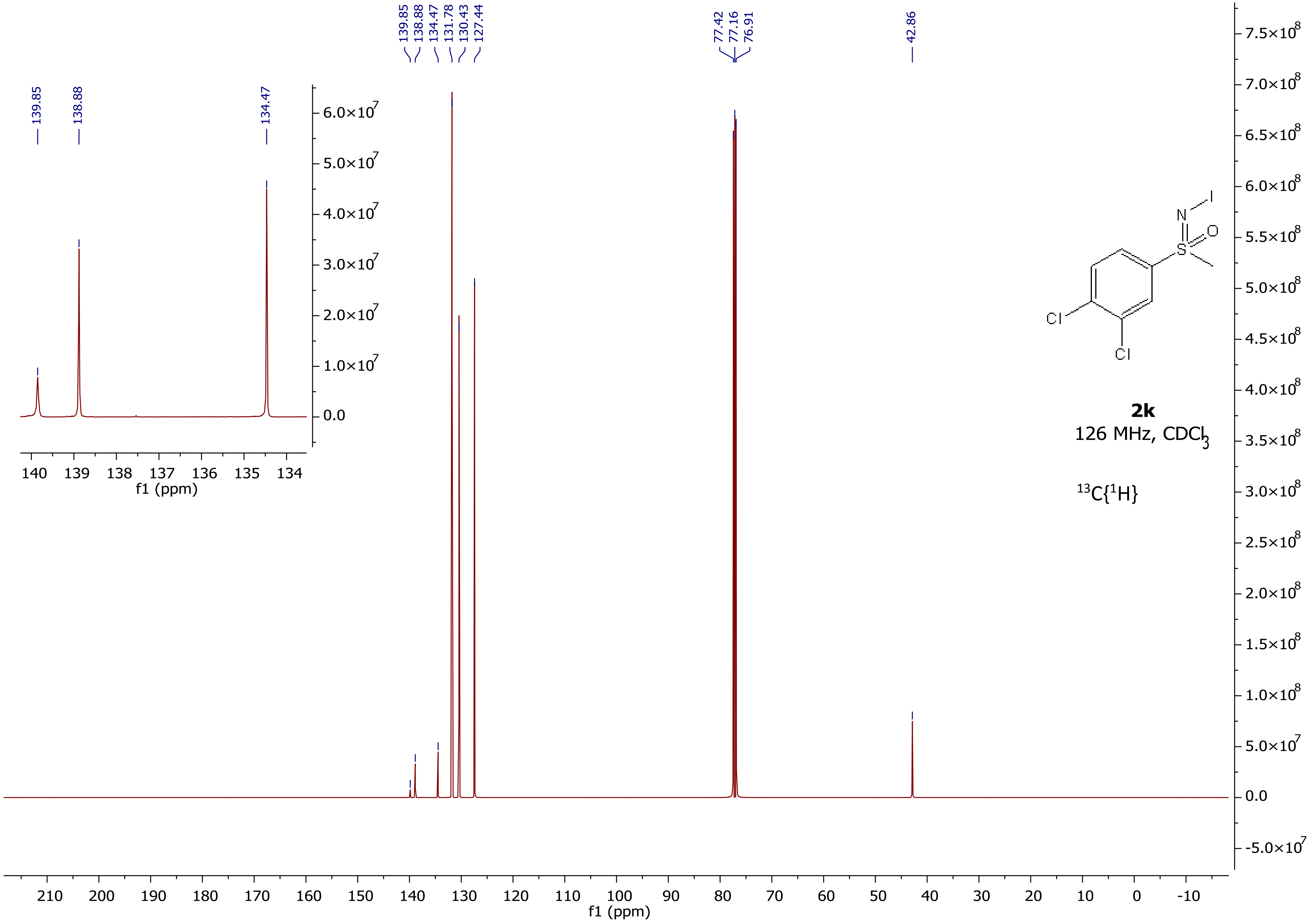
139.85
138.88
134.47
131.78
130.43
127.44
77.42
77.16
76.91
42.86
2k
126 MHz, CDCl3
13C{1H}
f1 (ppm)

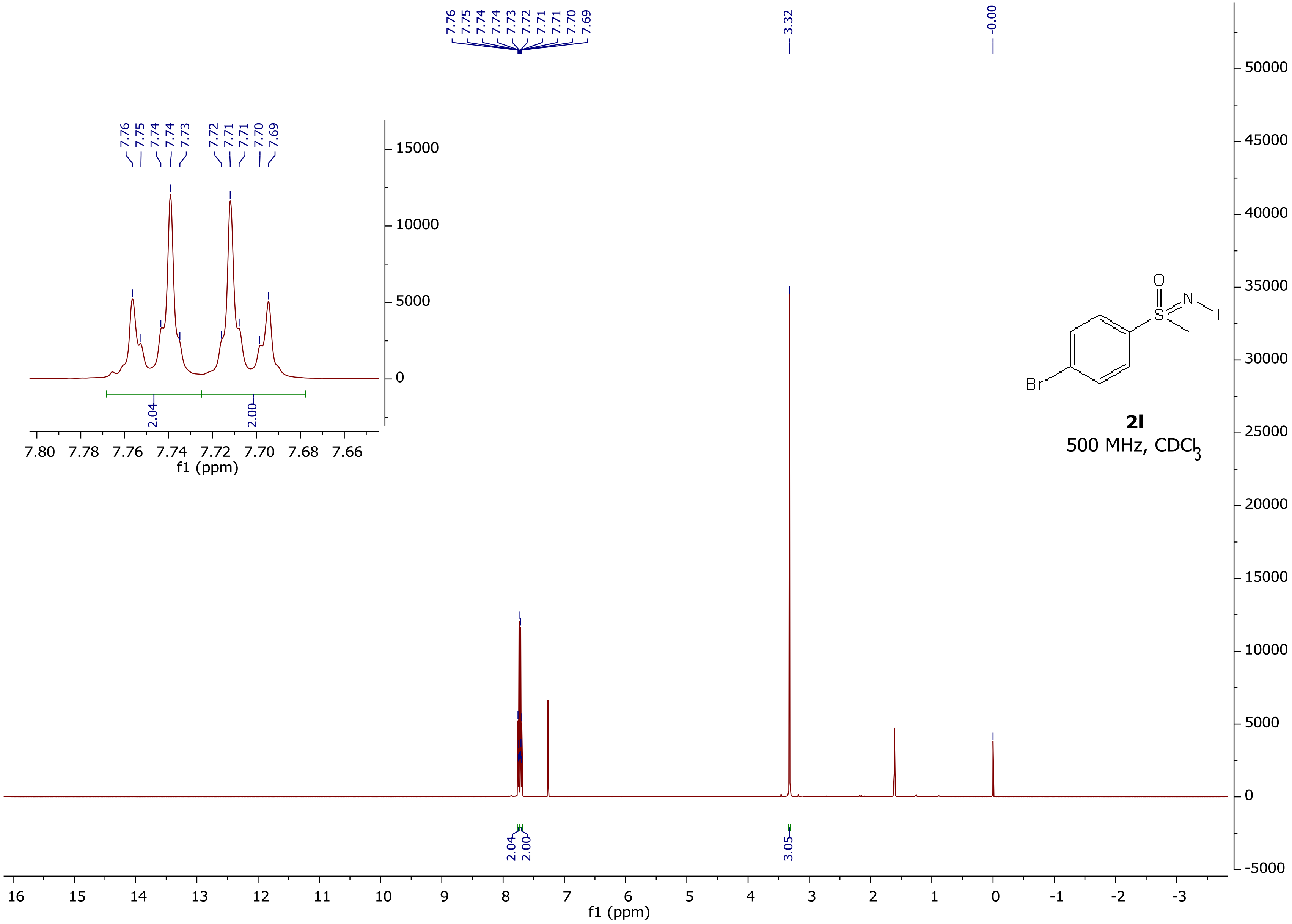
7.76
7.75
7.74
7.74
7.73
7.72
7.71
7.71
7.70
7.69
3.32
-0.00
2.04
2.00
3.05
f1 (ppm)
2l
500 MHz, CDCl3
Br
O
S
N
I

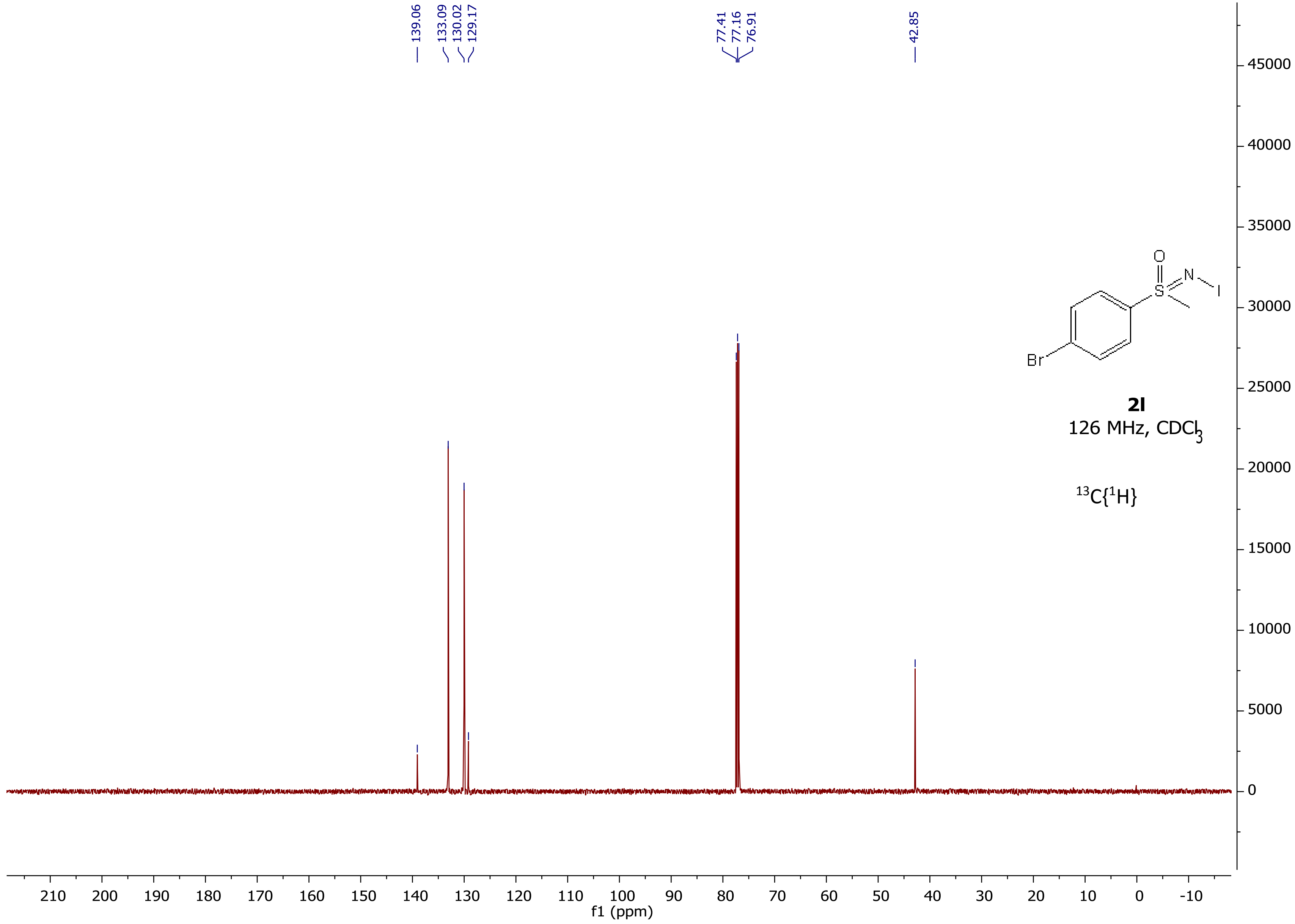
139.06
133.09
130.02
129.17
77.41
77.16
76.91
42.85
O
N
I
S
Br
2l
126 MHz, CDCl3
13C{1H}
f1 (ppm)

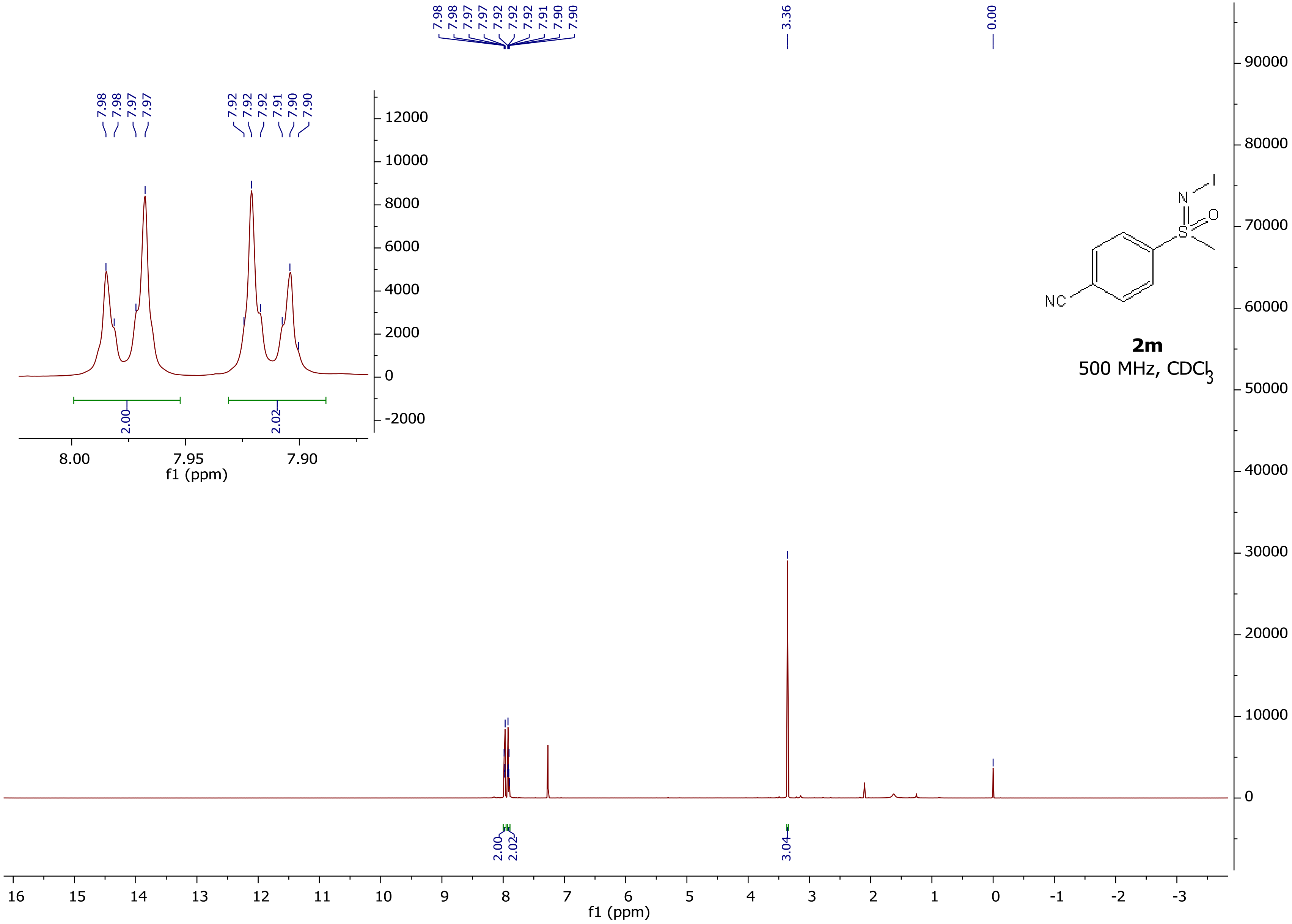

7.98
7.98
7.97
7.97
7.92
7.92
7.92
7.91
7.90
7.90
3.36
0.00
2m
500 MHz, CDCl3
NC
N
I
S
O
2.00
2.02
3.04
f1 (ppm)

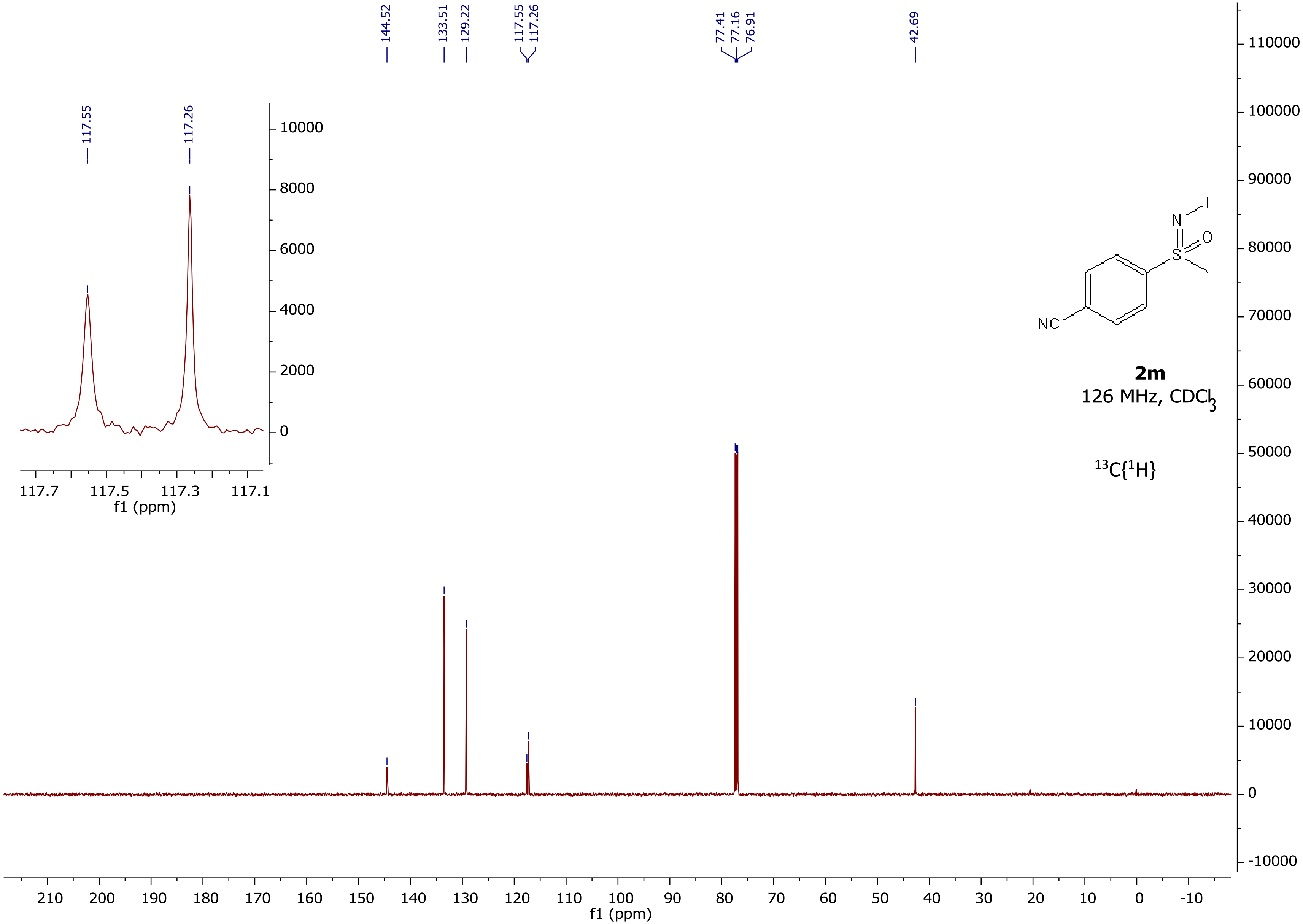

144.52
133.51
129.22
117.55
117.26
77.41
77.16
76.91
42.69
117.7
117.5
117.3
117.1
f1 (ppm)
NC
N
S
O
I
2m
126 MHz, CDCl3
13C{1H}
210
200
190
180
170
160
150
140
130
120
110
100
90
80
70
60
50
40
30
20
10
0
-10
f1 (ppm)

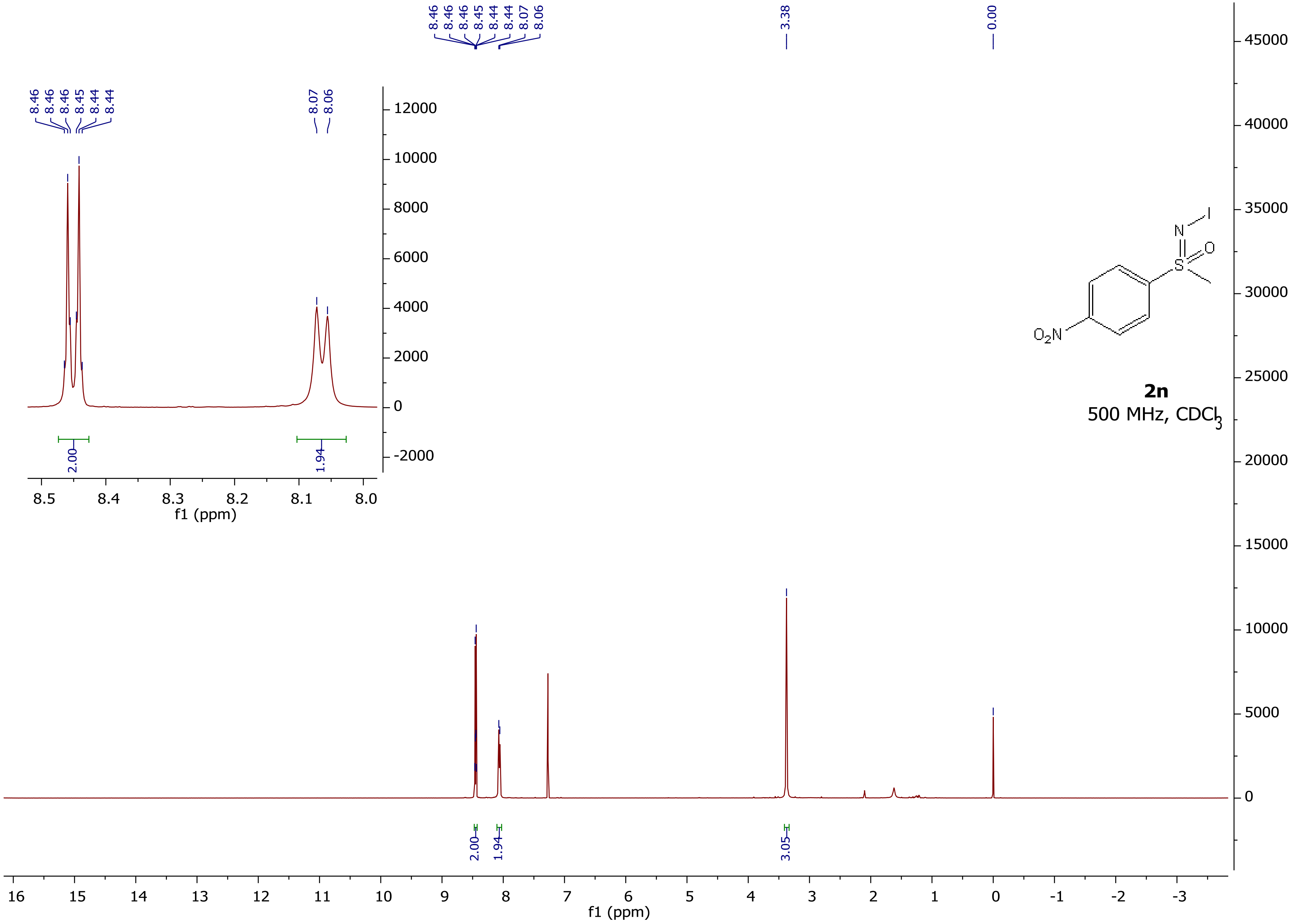
8.46
8.46
8.46
8.45
8.44
8.44
8.07
8.06
3.38
0.00
2n
500 MHz, CDCl3
O2N
N
I
S
O
2.00
1.94
3.05
f1 (ppm)

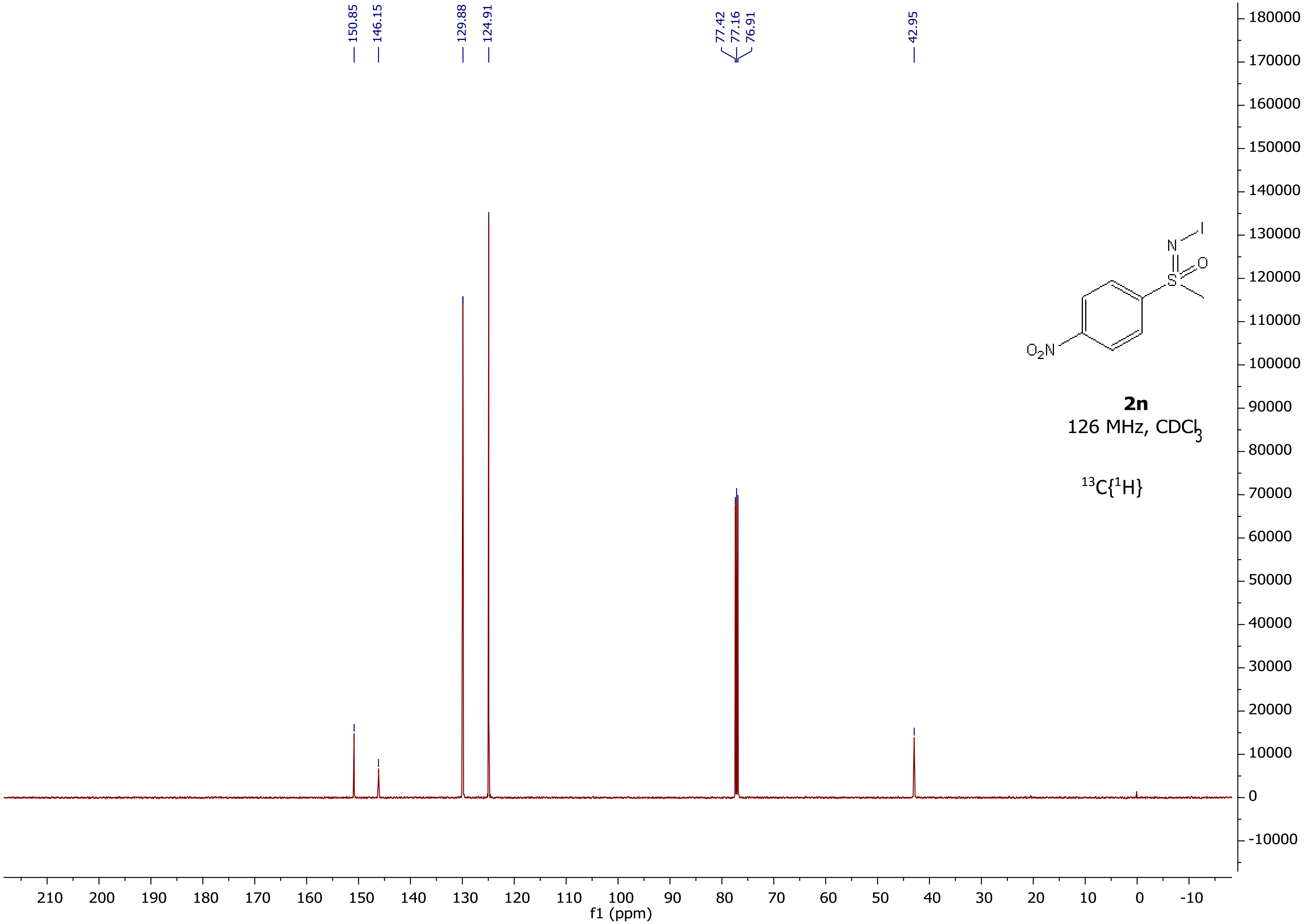
150.85
146.15
129.88
124.91
77.42
77.16
76.91
42.95
N
I
S
O
O2N
2n
126 MHz, CDCl3
13C{1H}
f1 (ppm)

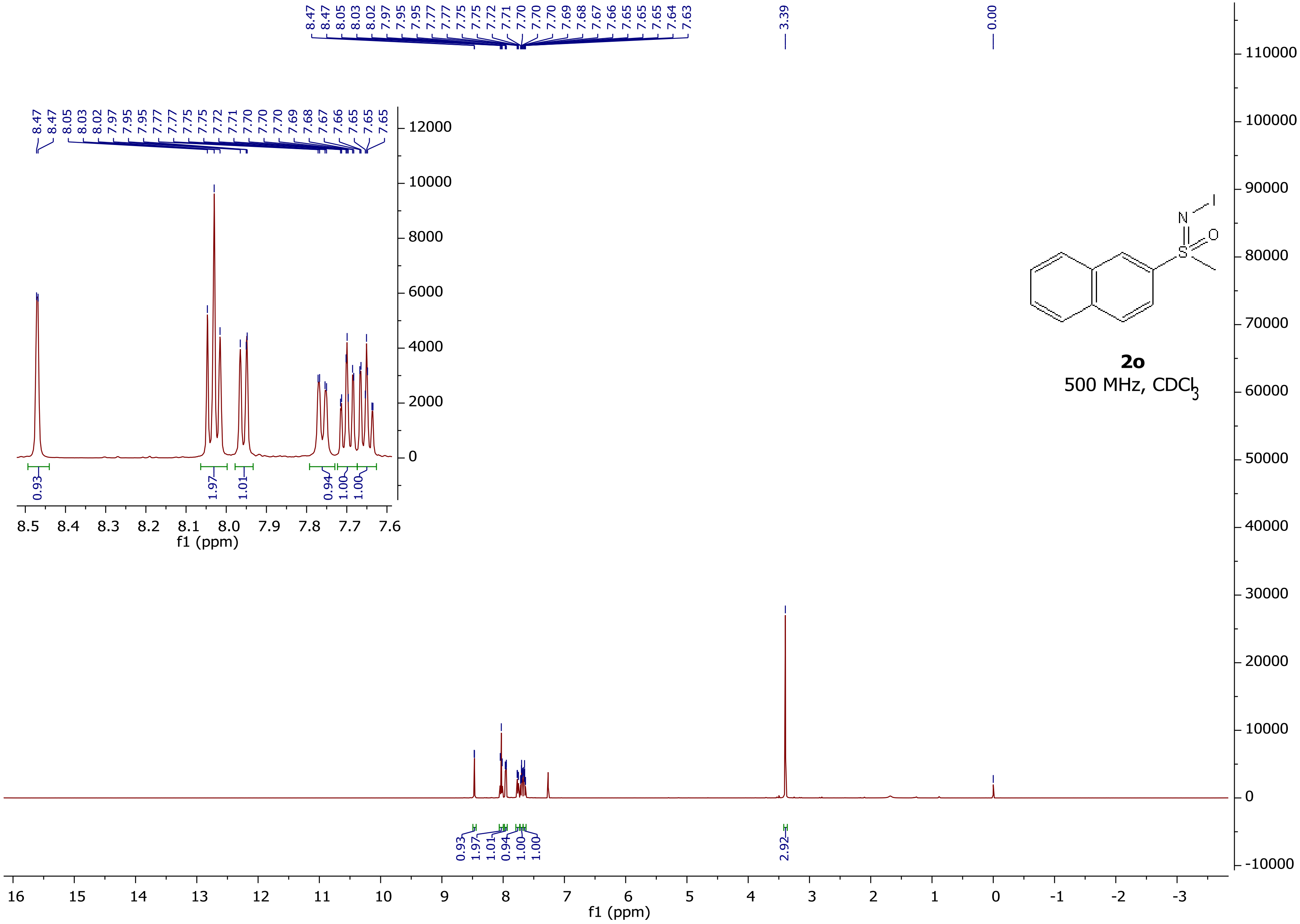
2o
500 MHz, CDCl3
f1 (ppm)

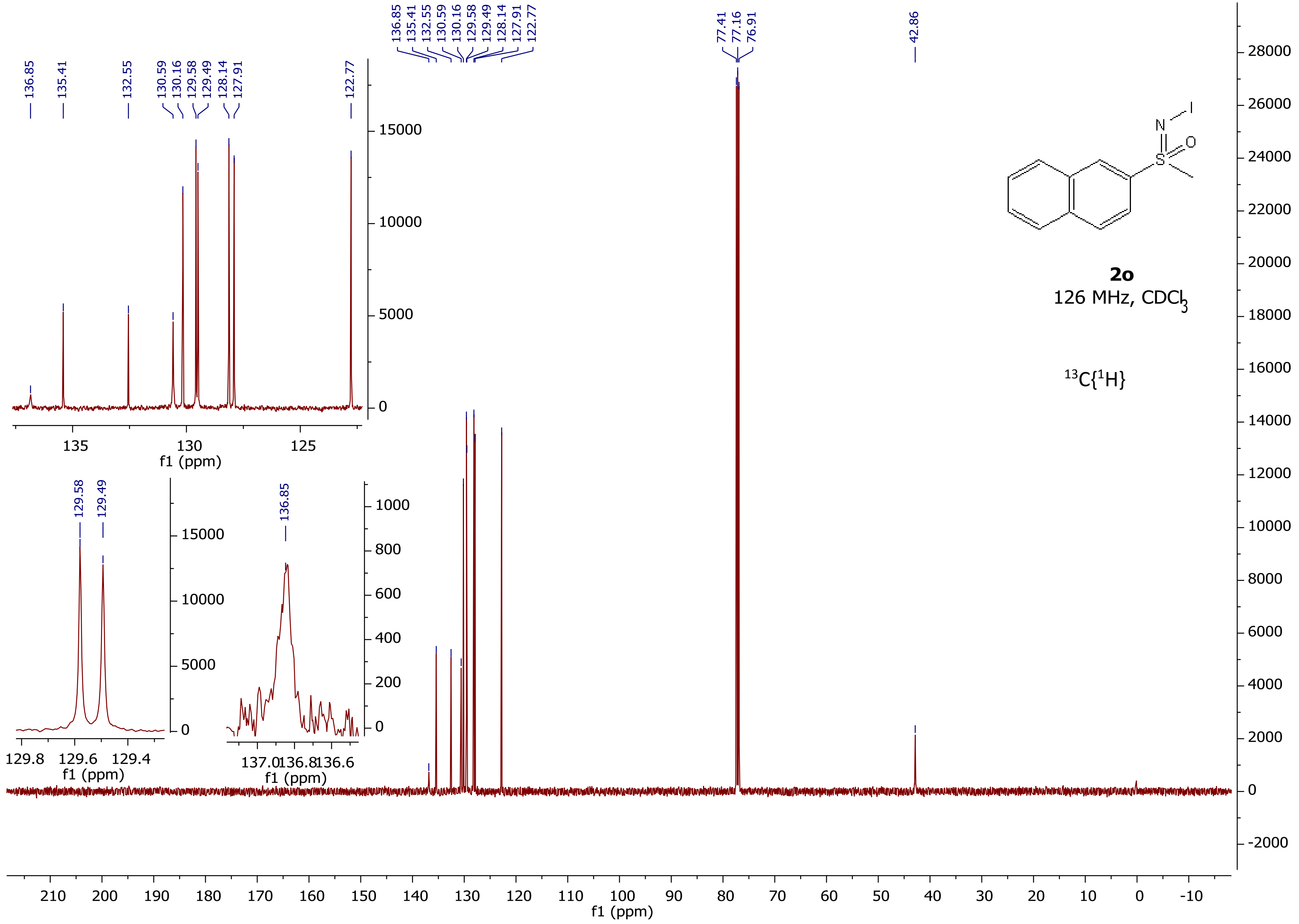

2o
126 MHz, CDCl3
13C{1H}
136.85
135.41
132.55
130.59
130.16
129.58
129.49
128.14
127.91
122.77
77.41
77.16
76.91
42.86
f1 (ppm)

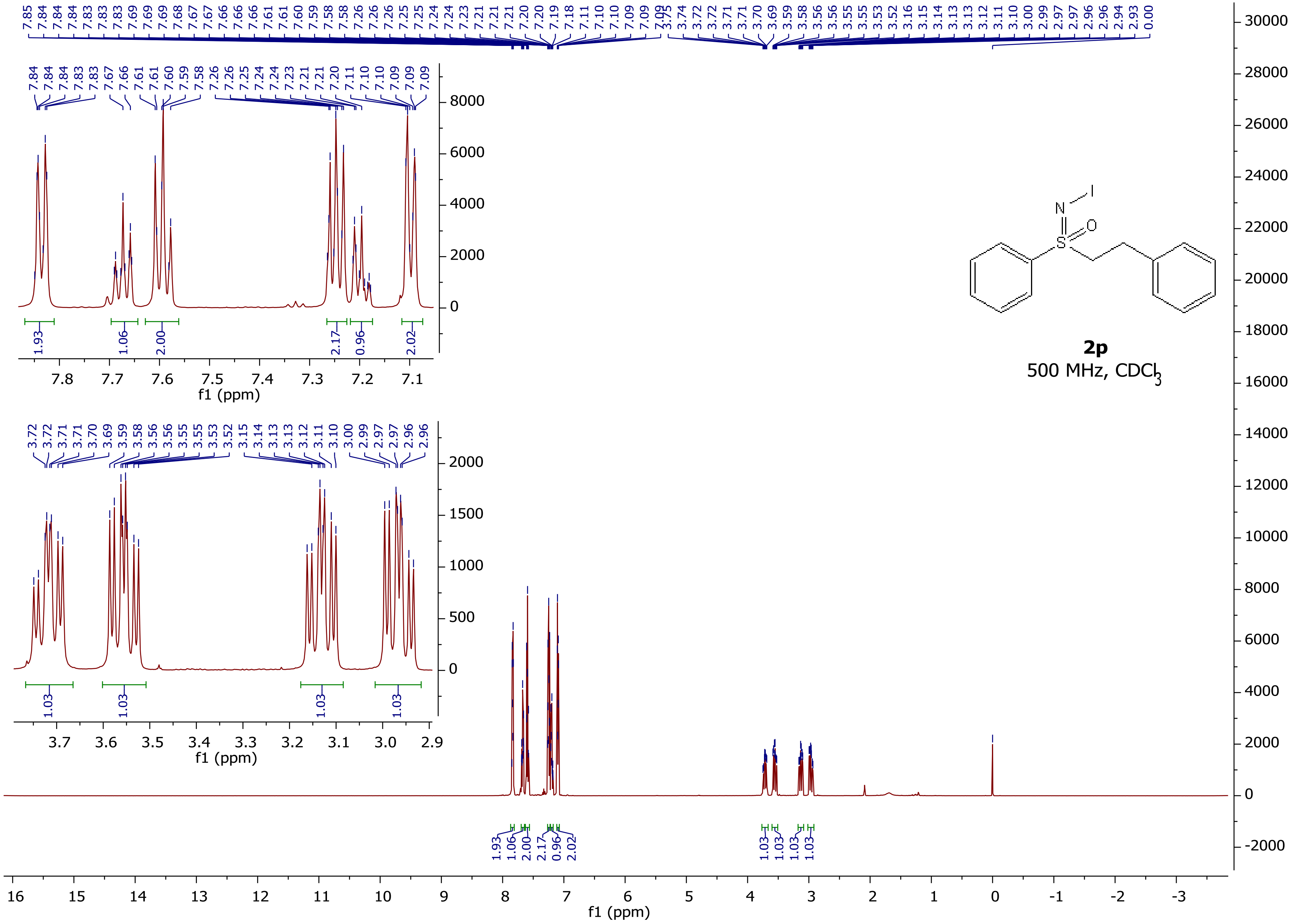
N
I
S
O
2p
500 MHz, CDCl3
f1 (ppm)

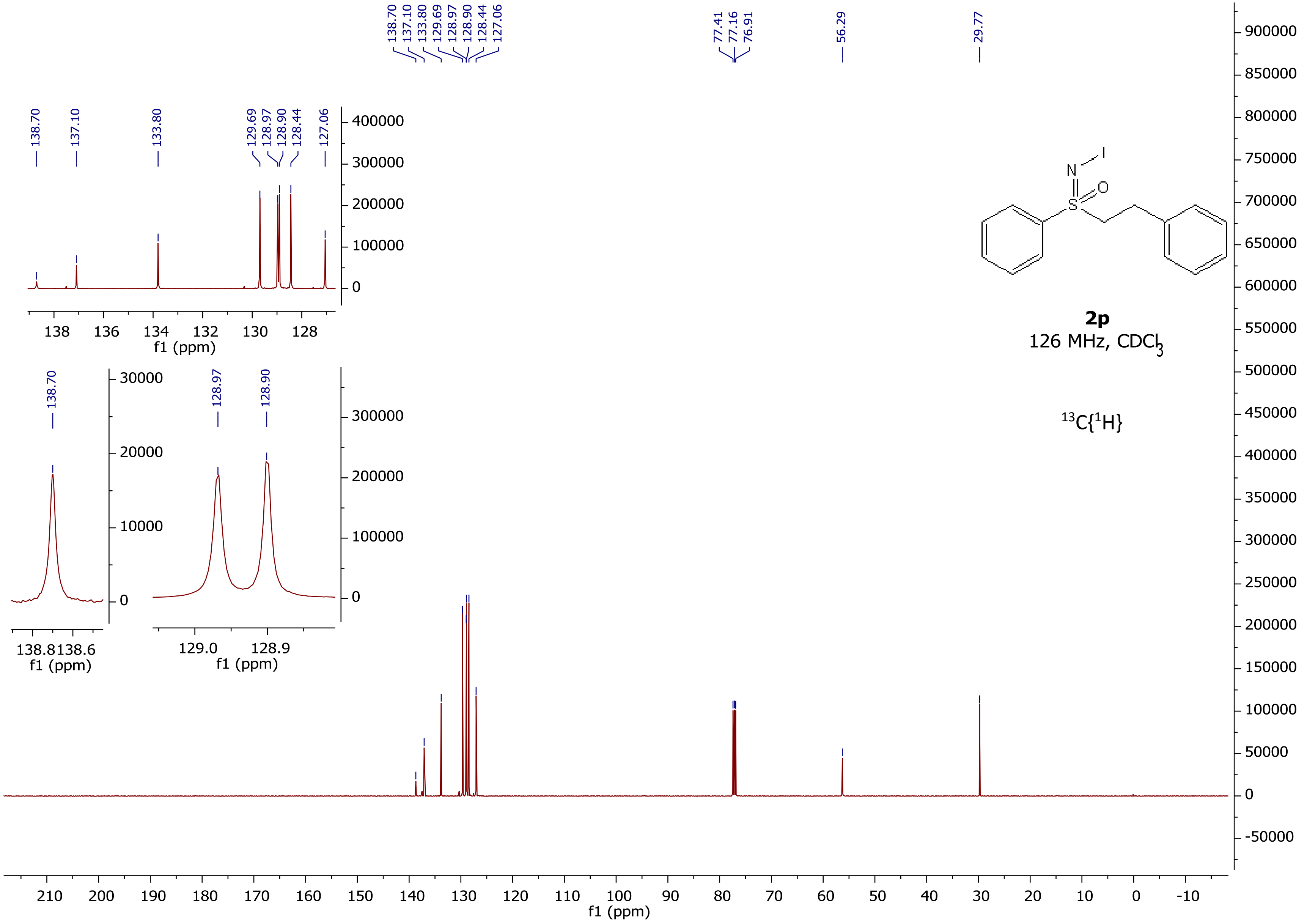

138.70
137.10
133.80
129.69
128.97
128.90
128.44
127.06
77.41
77.16
76.91
56.29
29.77
2p
126 MHz, CDCl3
13C{1H}
f1 (ppm)

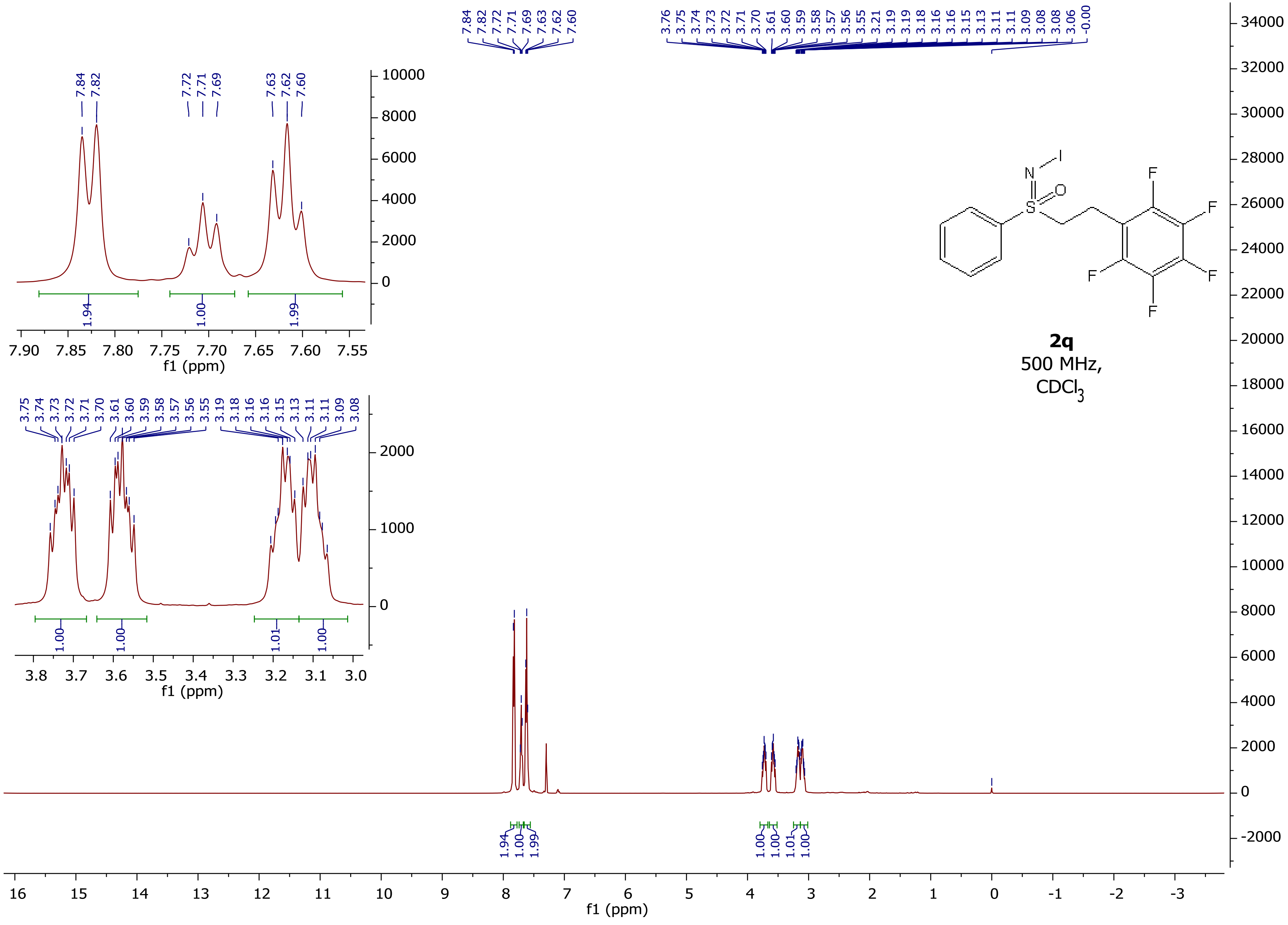
7.84 7.82 7.72 7.71 7.69 7.63 7.62 7.60
3.76 3.75 3.74 3.73 3.72 3.71 3.70 3.61 3.60 3.59 3.58 3.57 3.56 3.55 3.21 3.19 3.19 3.18 3.16 3.16 3.15 3.13 3.11 3.11 3.09 3.08 3.08 3.06 -0.00
1.94 1.00 1.99
1.00 1.00 1.01 1.00
f1 (ppm)
2q
500 MHz,
CDCl3

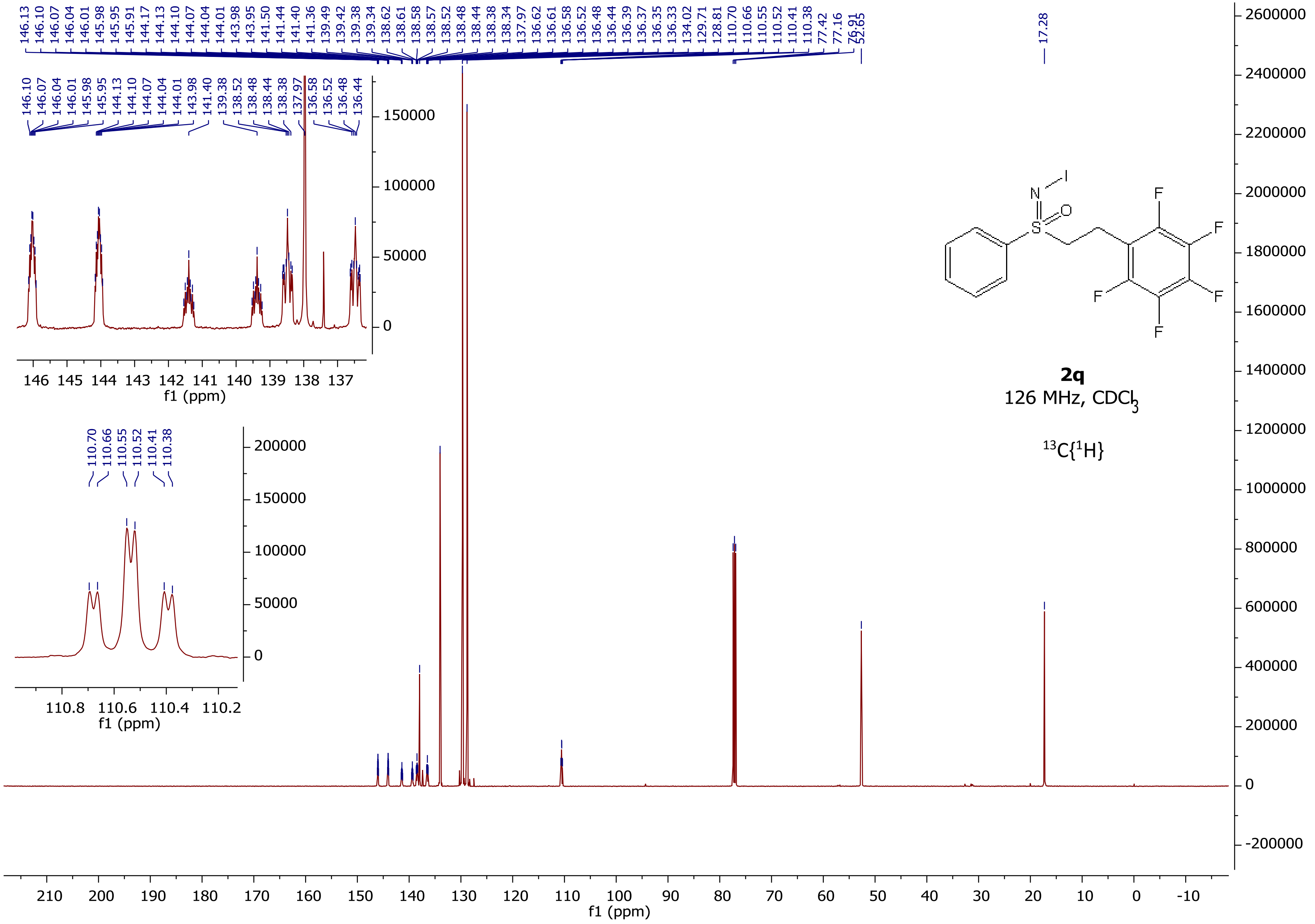

146.13
146.10
146.07
146.04
146.01
145.98
145.95
145.91
144.17
144.13
144.10
144.07
144.04
144.01
143.98
143.95
141.50
141.44
141.40
141.36
139.49
139.42
139.38
139.34
138.62
138.61
138.58
138.57
138.52
138.48
138.44
138.38
138.34
137.97
136.62
136.61
136.58
136.52
136.48
136.44
136.39
136.37
136.35
136.33
134.02
129.71
128.81
110.70
110.66
110.55
110.52
110.41
110.38
77.42
77.16
76.91
52.65
17.28
2q
126 MHz, CDCl3
13C{1H}
f1 (ppm)

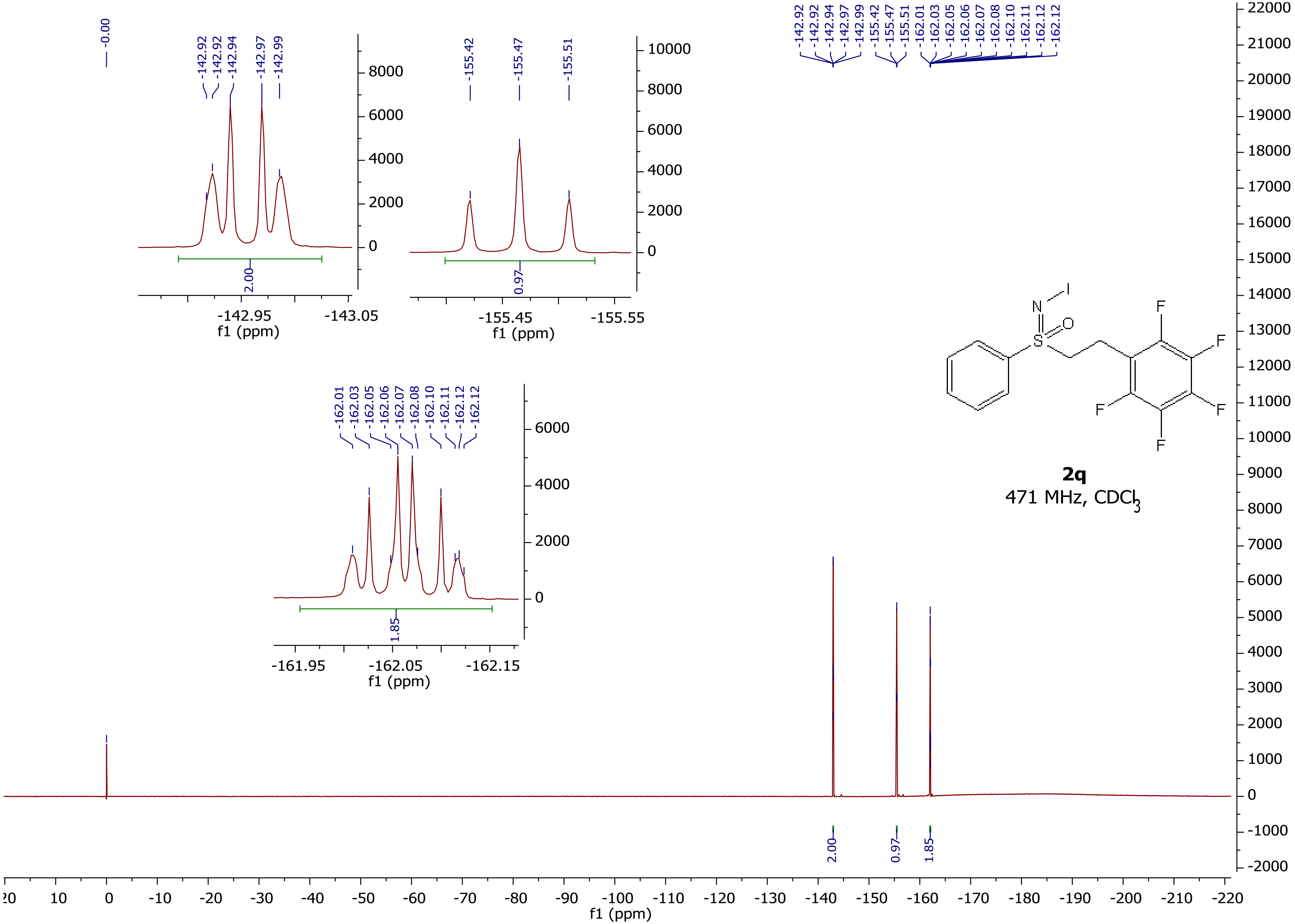
2q
471 MHz, CDCl3
f1 (ppm)

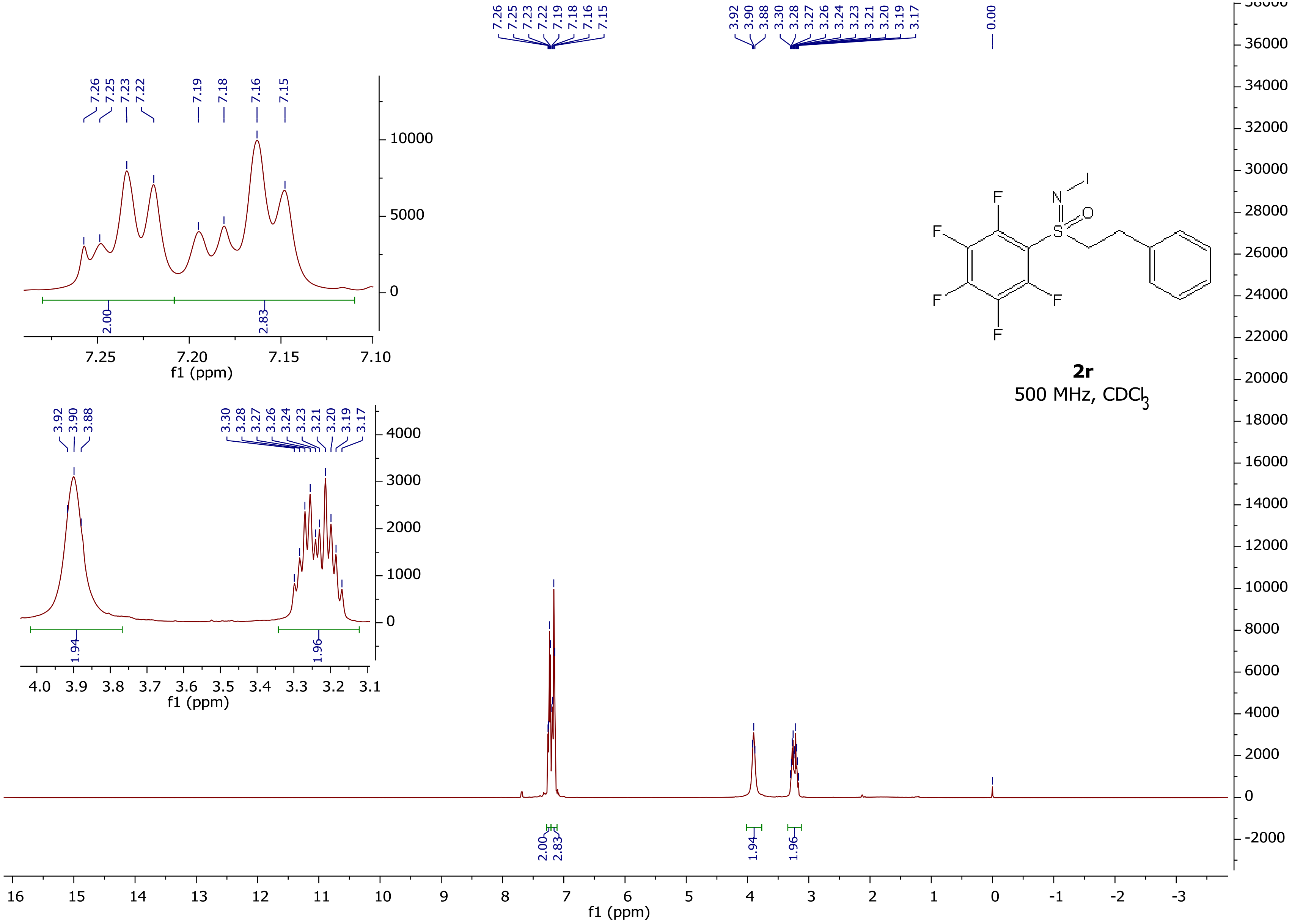

2r
500 MHz, $CDCl_3$

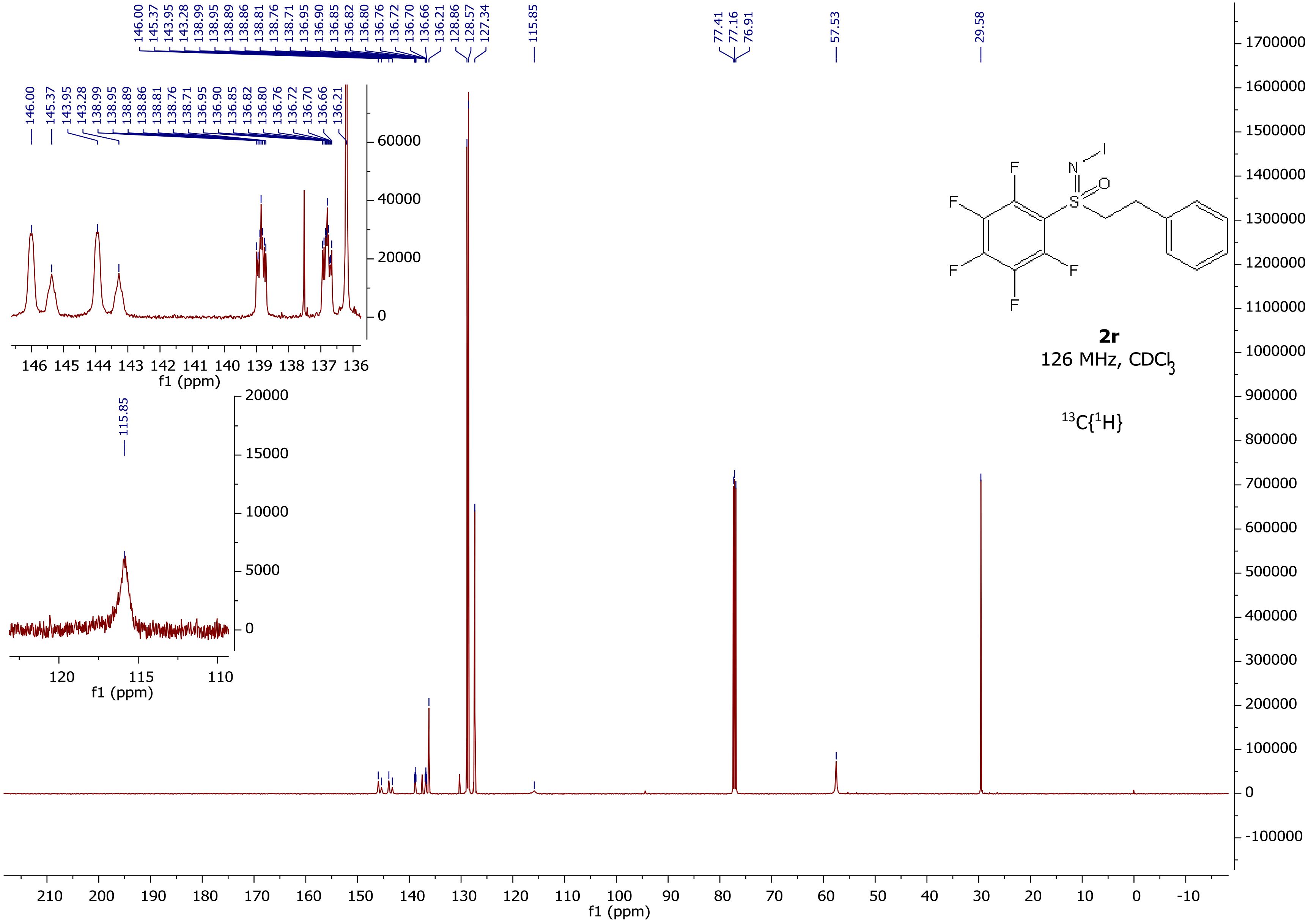

146.00
145.37
143.95
143.28
138.99
138.95
138.89
138.86
138.81
138.76
138.71
136.95
136.90
136.85
136.82
136.80
136.76
136.72
136.70
136.66
136.21
128.86
128.57
127.34
115.85
77.41
77.16
76.91
57.53
29.58
2r
126 MHz, CDCl3
13C{1H}
f1 (ppm)

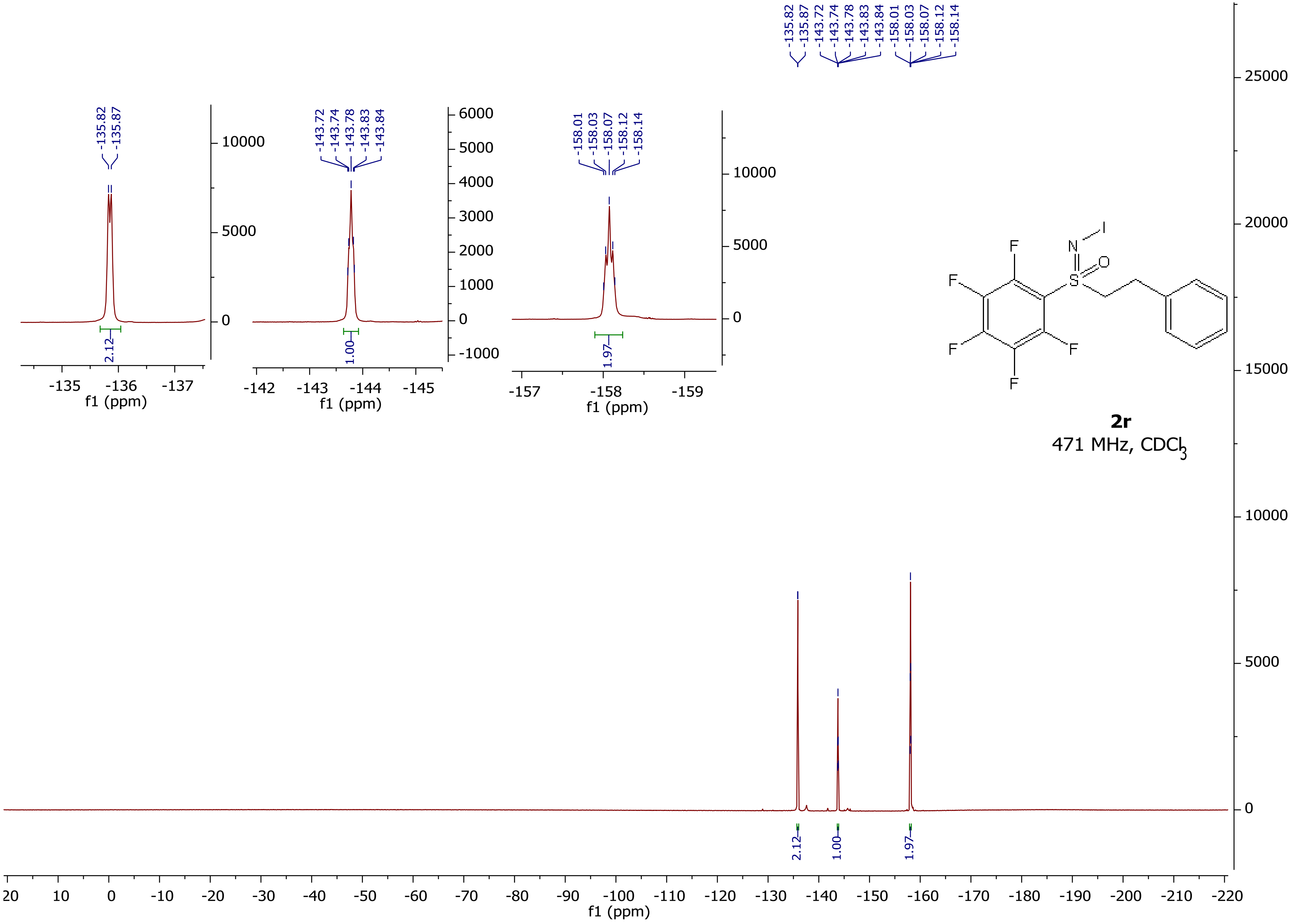
-135.82
-135.87
-143.72
-143.74
-143.78
-143.83
-143.84
-158.01
-158.03
-158.07
-158.12
-158.14
2.12
1.00
1.97
f1 (ppm)
2r
471 MHz, CDCl3

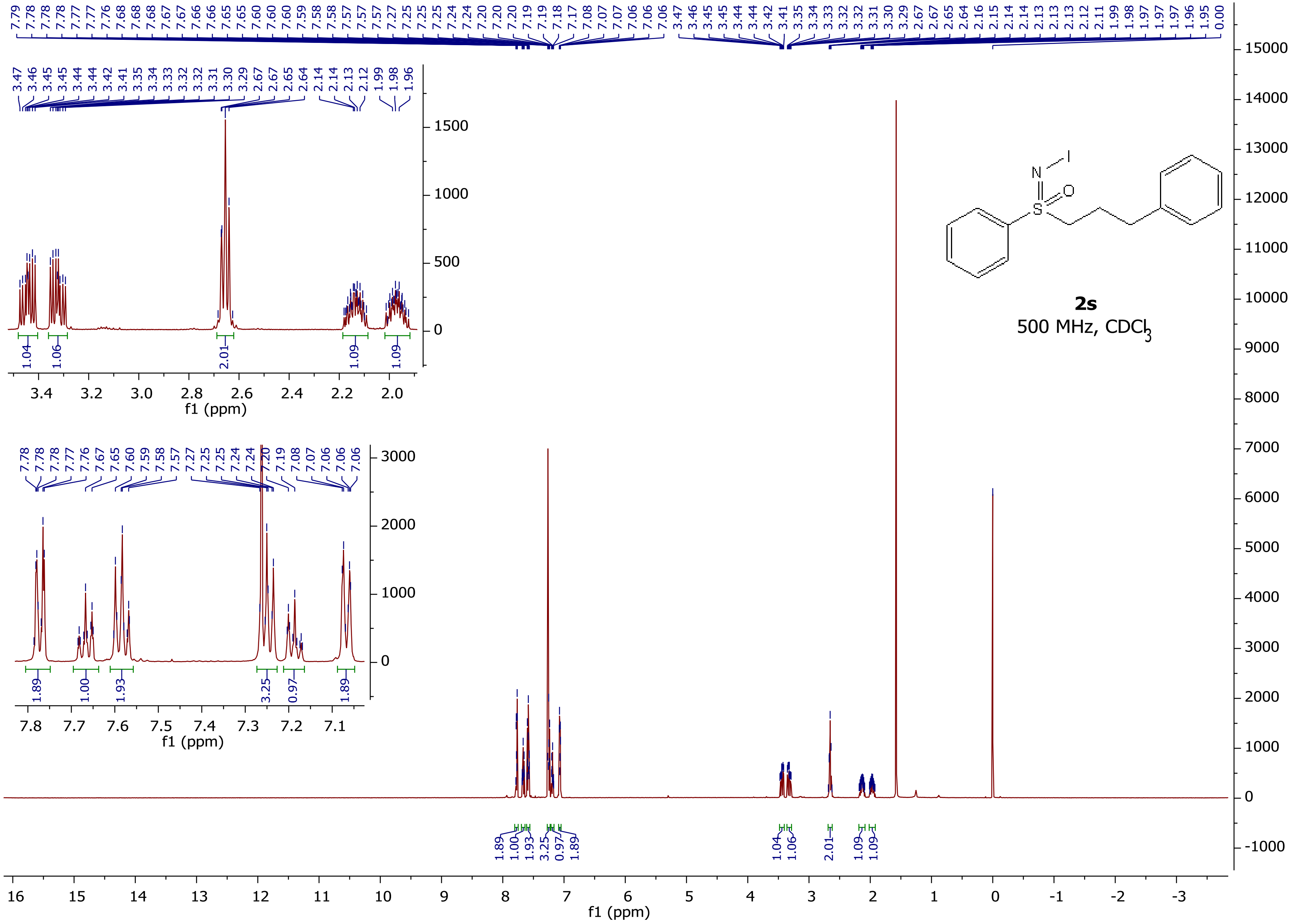
2s
500 MHz, CDCl3
f1 (ppm)

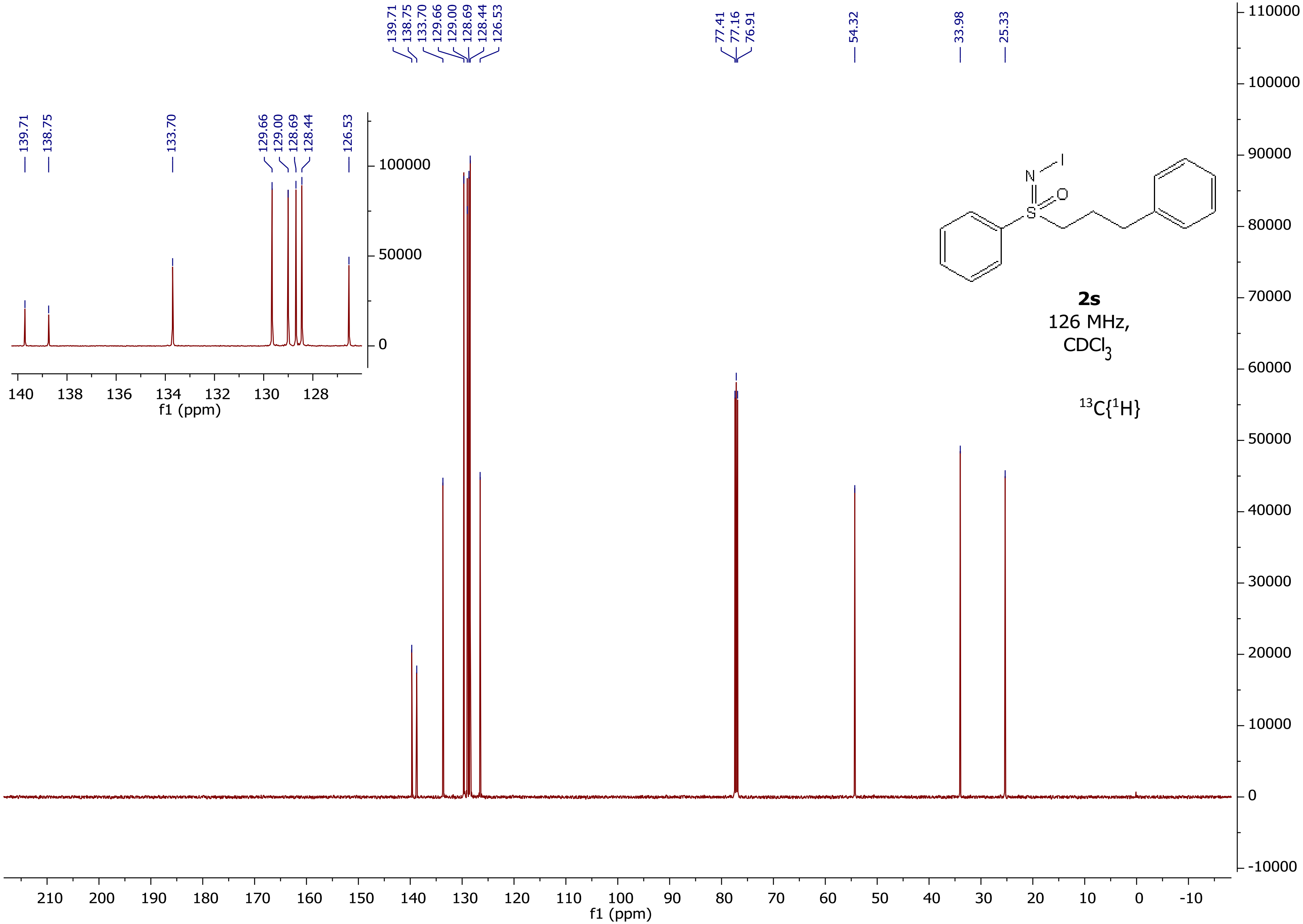

139.71
138.75
133.70
129.66
129.00
128.69
128.44
126.53
77.41
77.16
76.91
54.32
33.98
25.33
2s
126 MHz,
$CDCl_3$
$^{13}C\{^1H\}$
f1 (ppm)

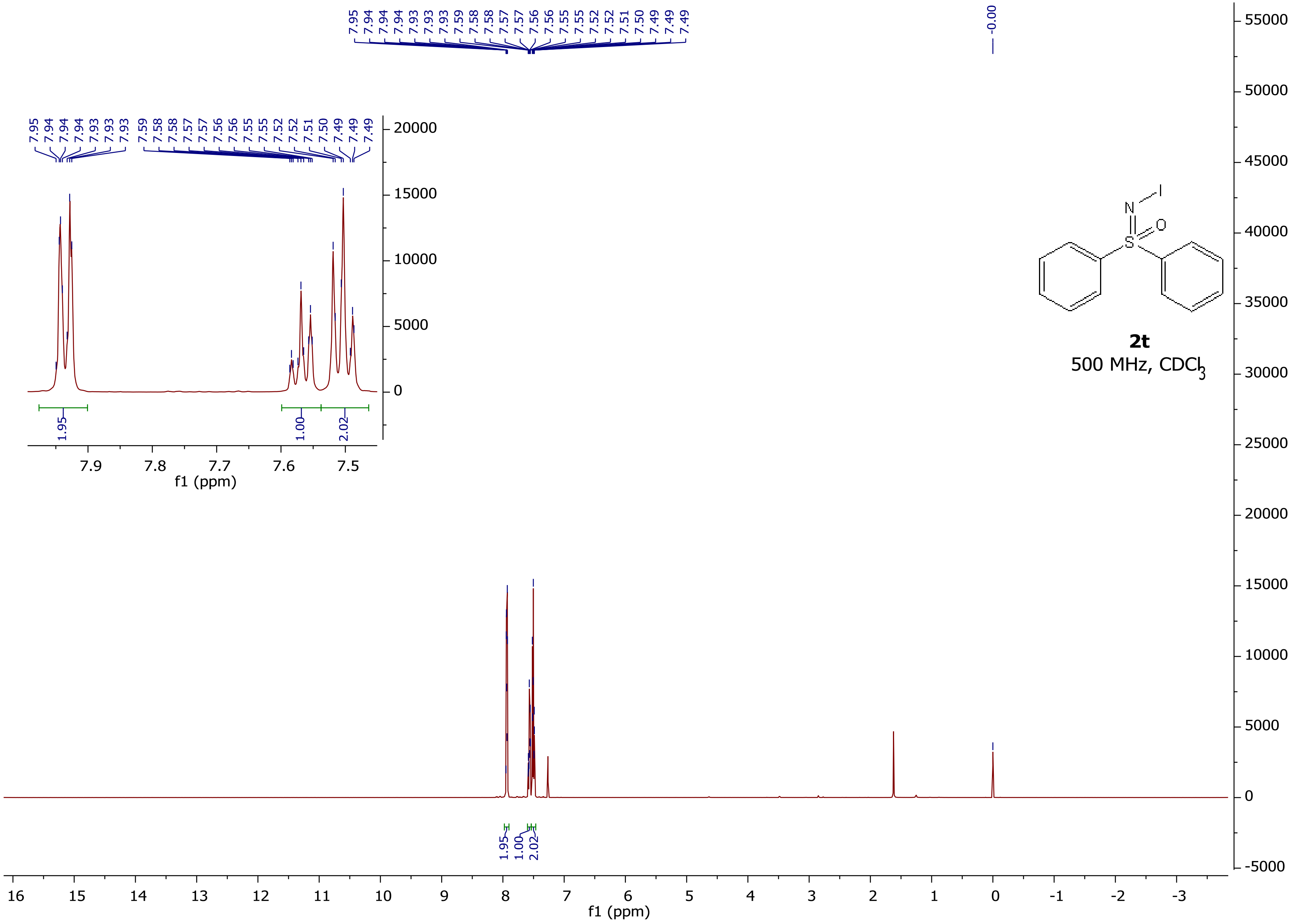

7.95 7.94 7.94 7.94 7.93 7.93 7.93 7.59 7.58 7.58 7.57 7.57 7.56 7.56 7.55 7.55 7.52 7.52 7.51 7.50 7.49 7.49 7.49
-0.00
1.95
1.00
2.02
f1 (ppm)
2t
500 MHz, $CDCl_3$

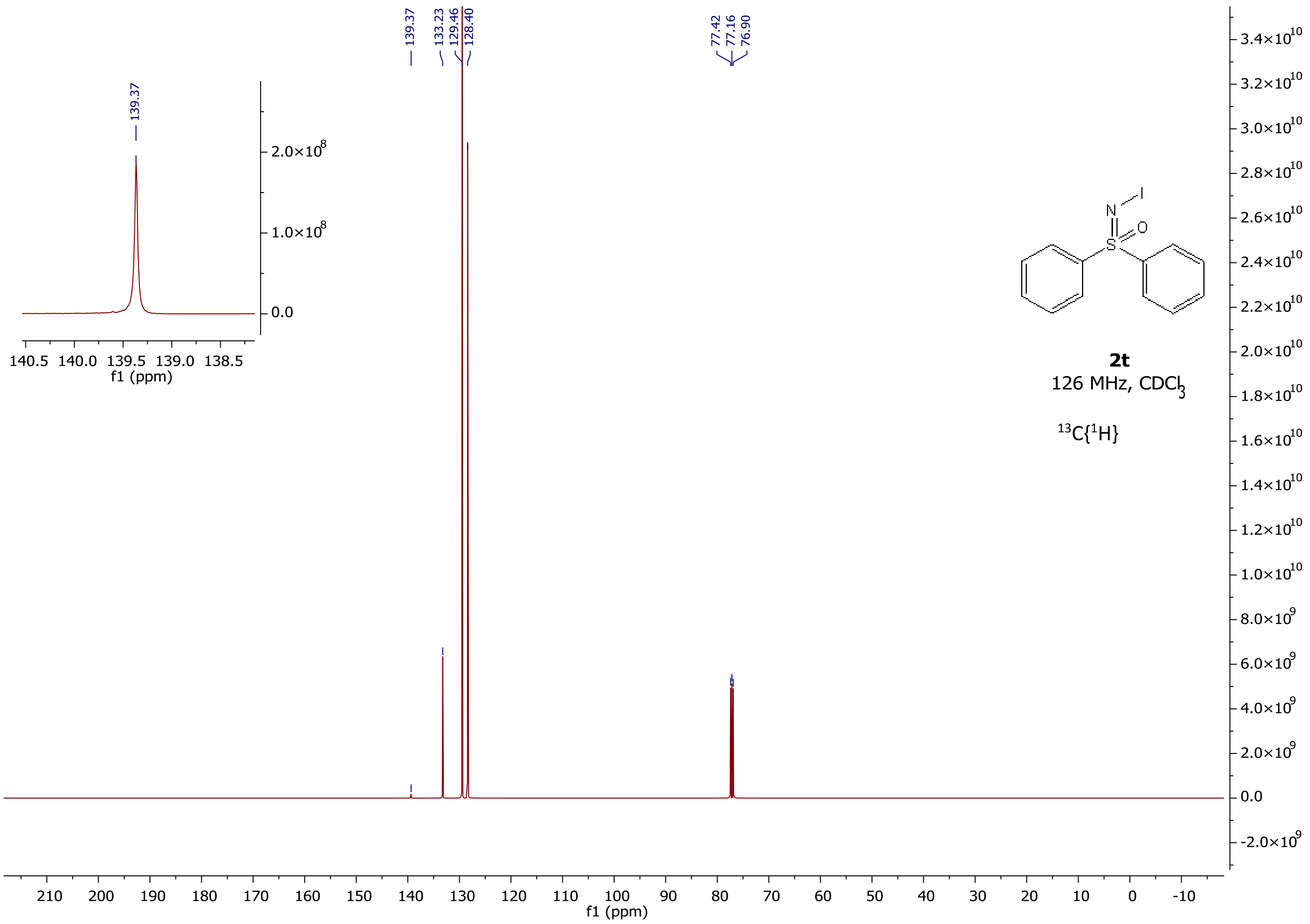
139.37
133.23
129.46
128.40
77.42
77.16
76.90
139.37
2t
126 MHz, CDCl3
13C{1H}
f1 (ppm)
f1 (ppm)

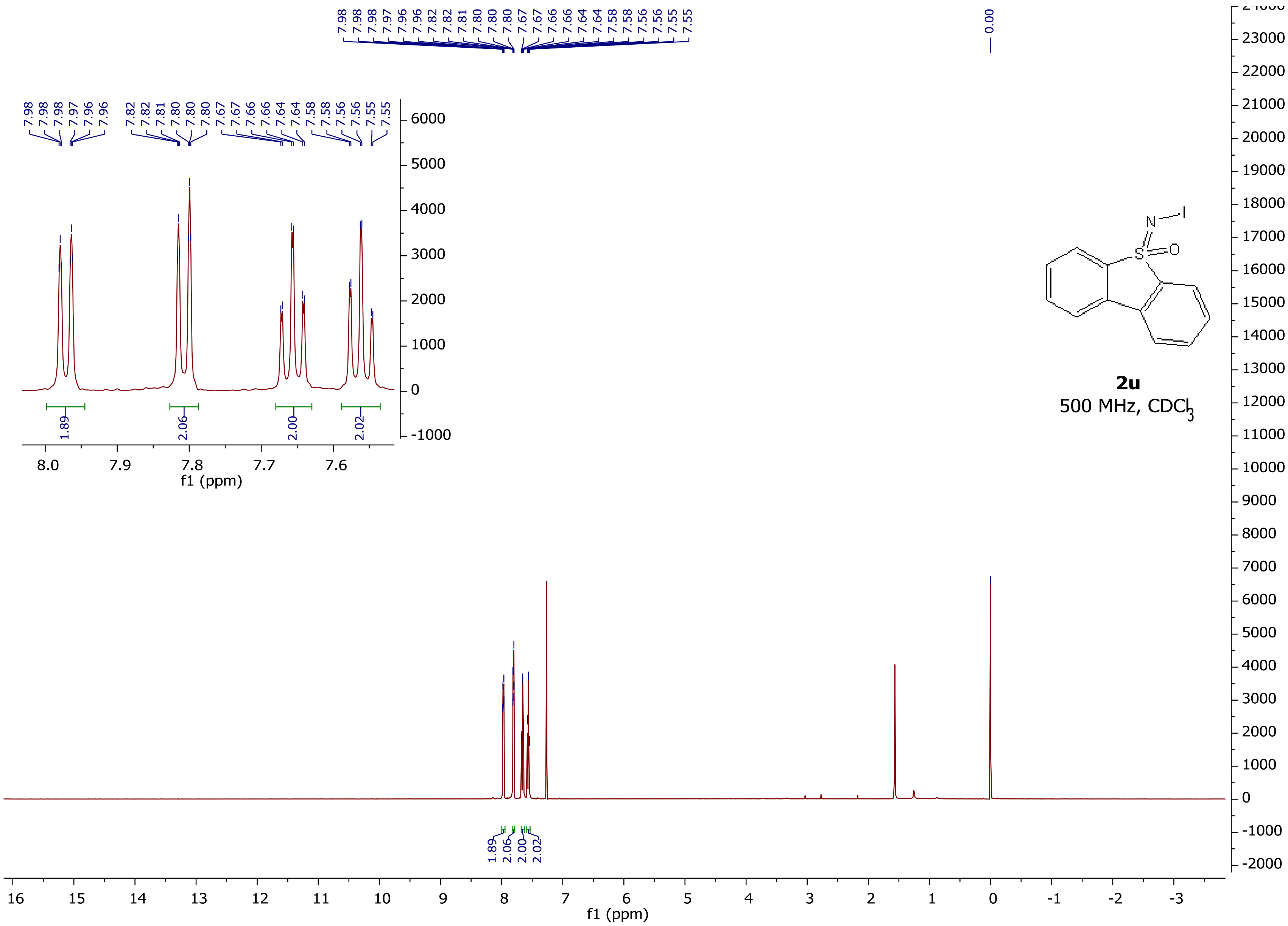
7.98 7.98 7.98 7.97 7.96 7.96 7.82 7.82 7.81 7.80 7.80 7.80 7.67 7.67 7.66 7.66 7.64 7.64 7.58 7.58 7.56 7.56 7.55 7.55
0.00
1.89
2.06
2.00
2.02
f1 (ppm)
2u
500 MHz, CDCl3

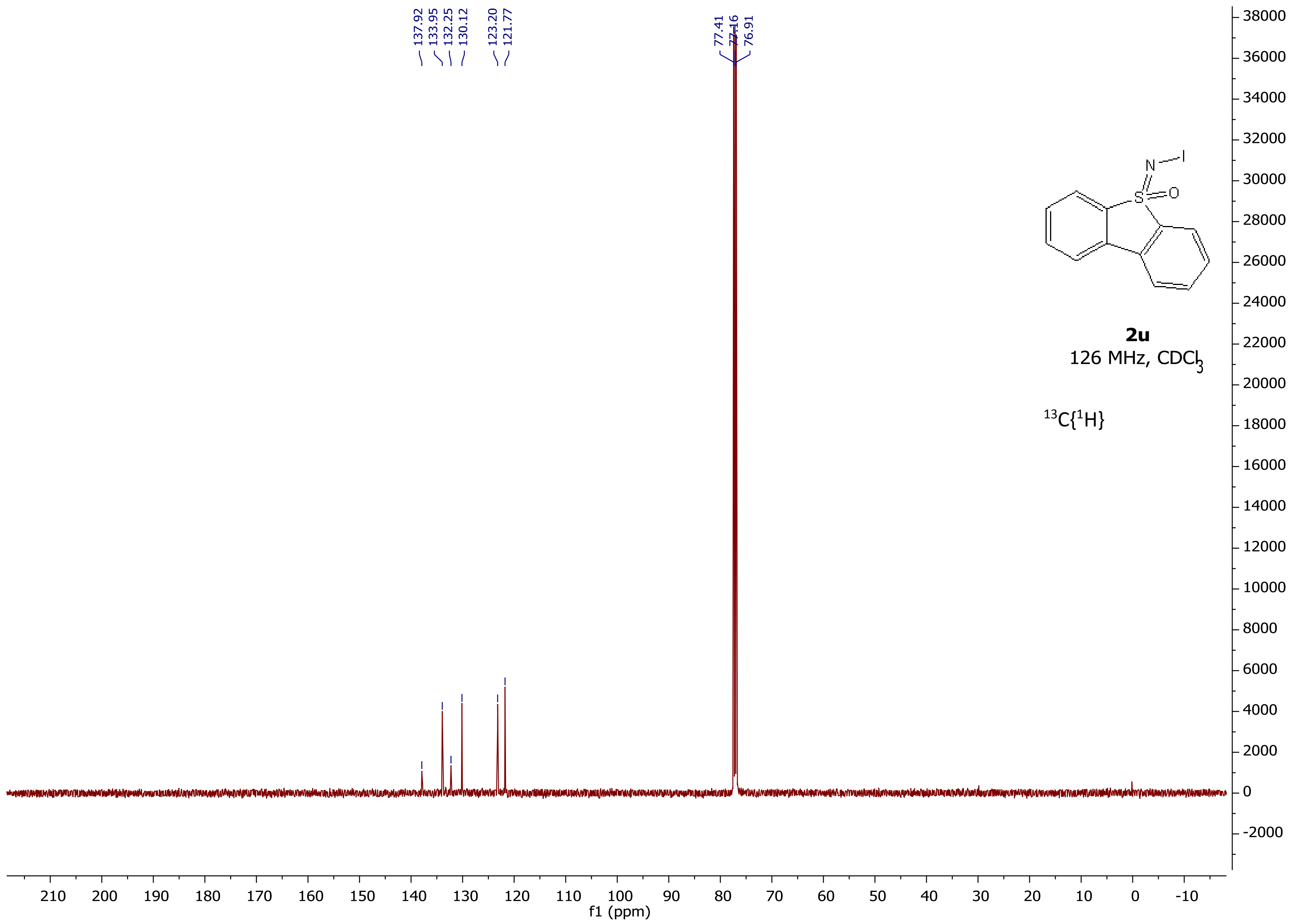
137.92
133.95
132.25
130.12
123.20
121.77
77.41
77.16
76.91
2u
126 MHz, CDCl3
13C{1H}
f1 (ppm)

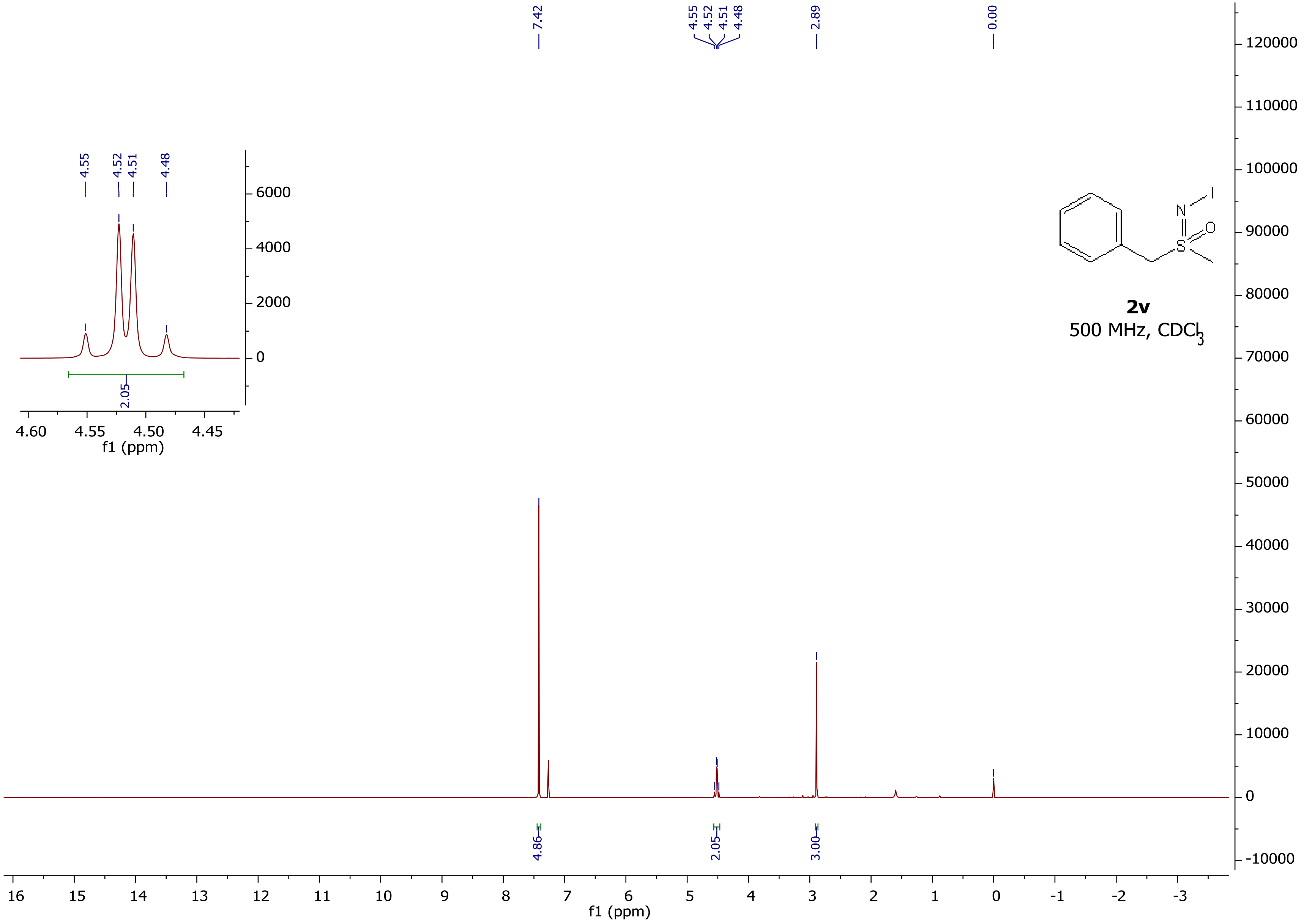
7.42
4.55
4.52
4.51
4.48
2.89
0.00
2v
500 MHz, CDCl3
N
I
O
S
4.86
2.05
3.00
f1 (ppm)

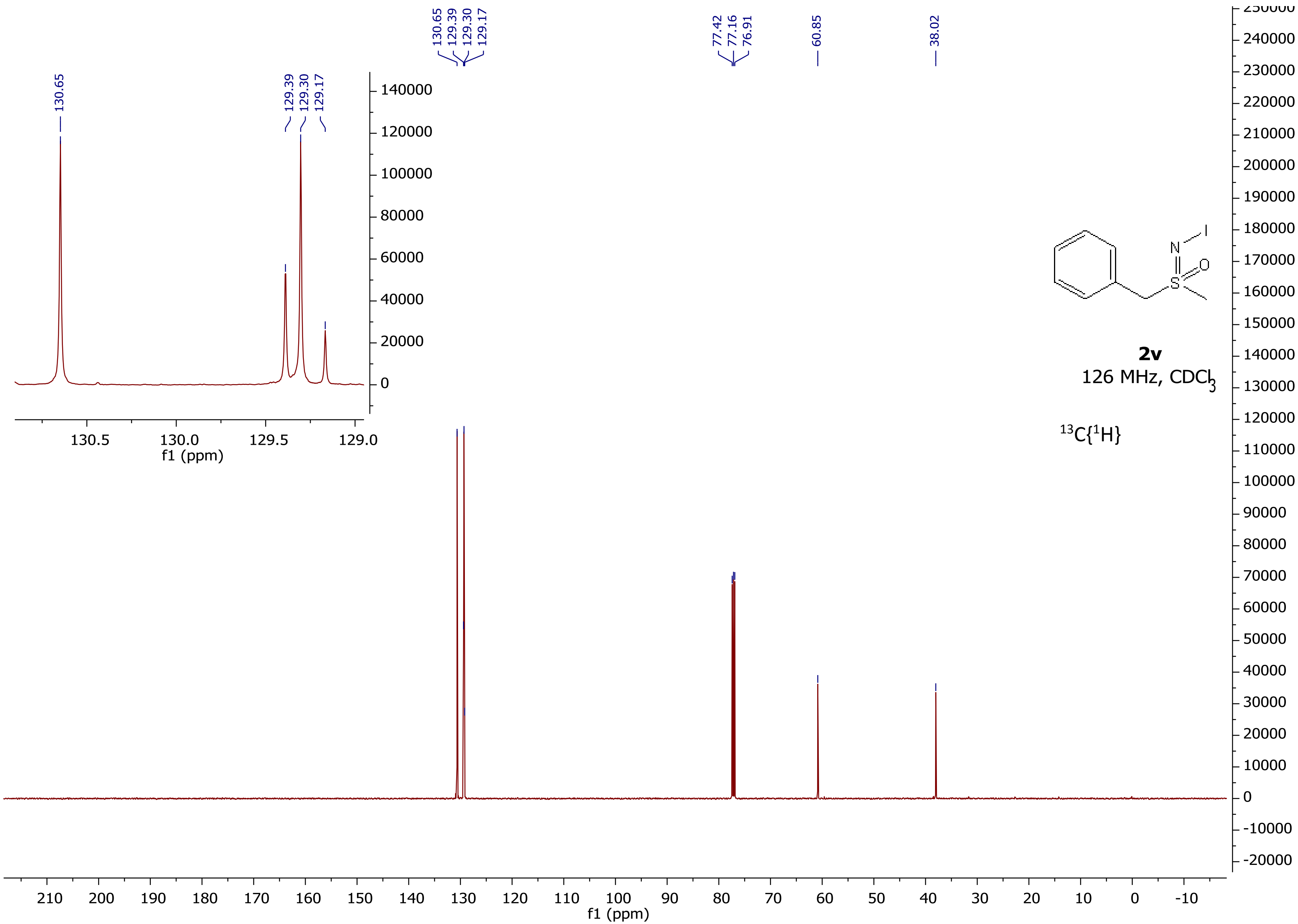
130.65
129.39
129.30
129.17
77.42
77.16
76.91
60.85
38.02
126 MHz, CDCl3
13C{1H}
f1 (ppm)

2v

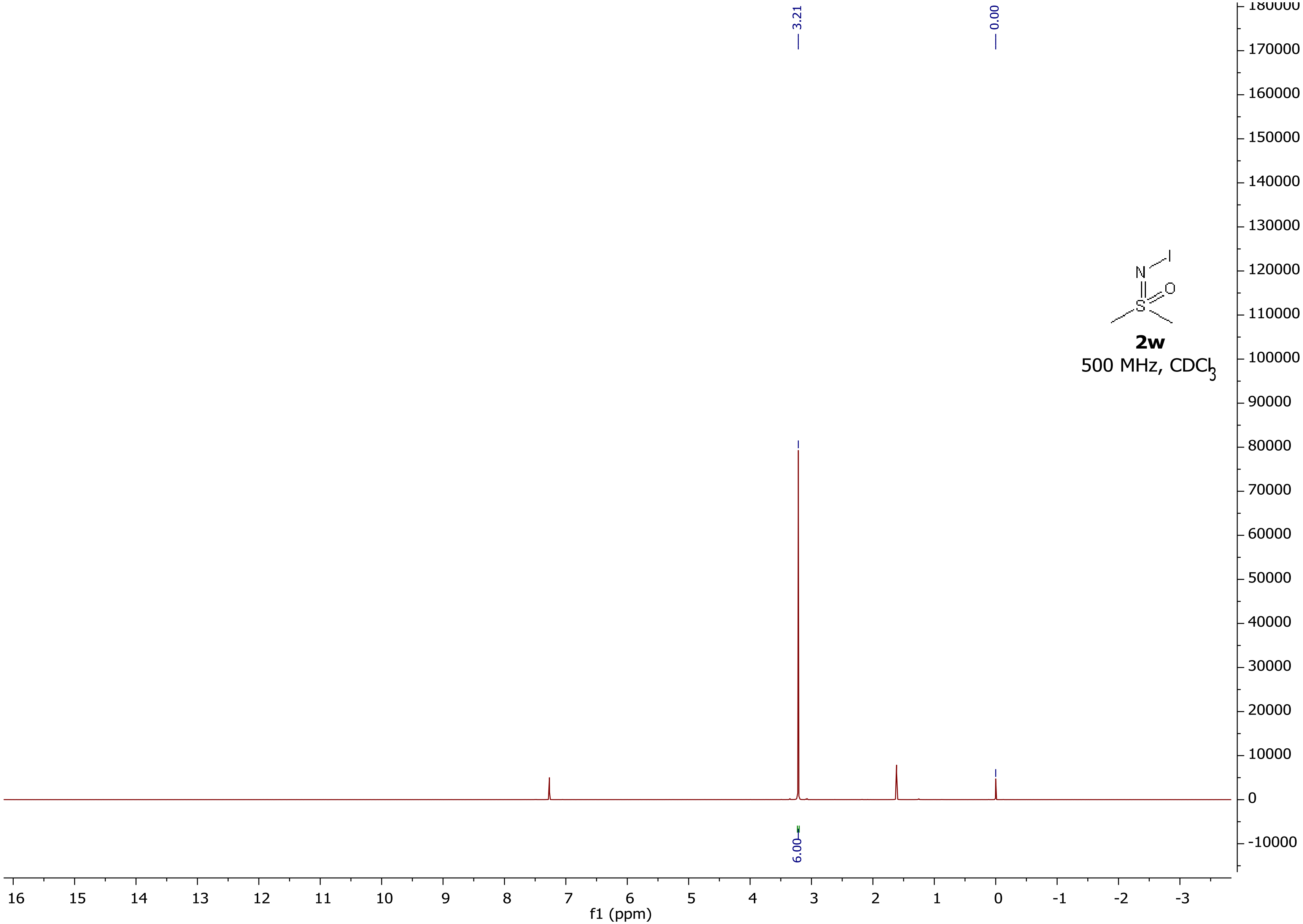

3.21
0.00
2w
500 MHz, $CDCl_3$
6.00
f1 (ppm)

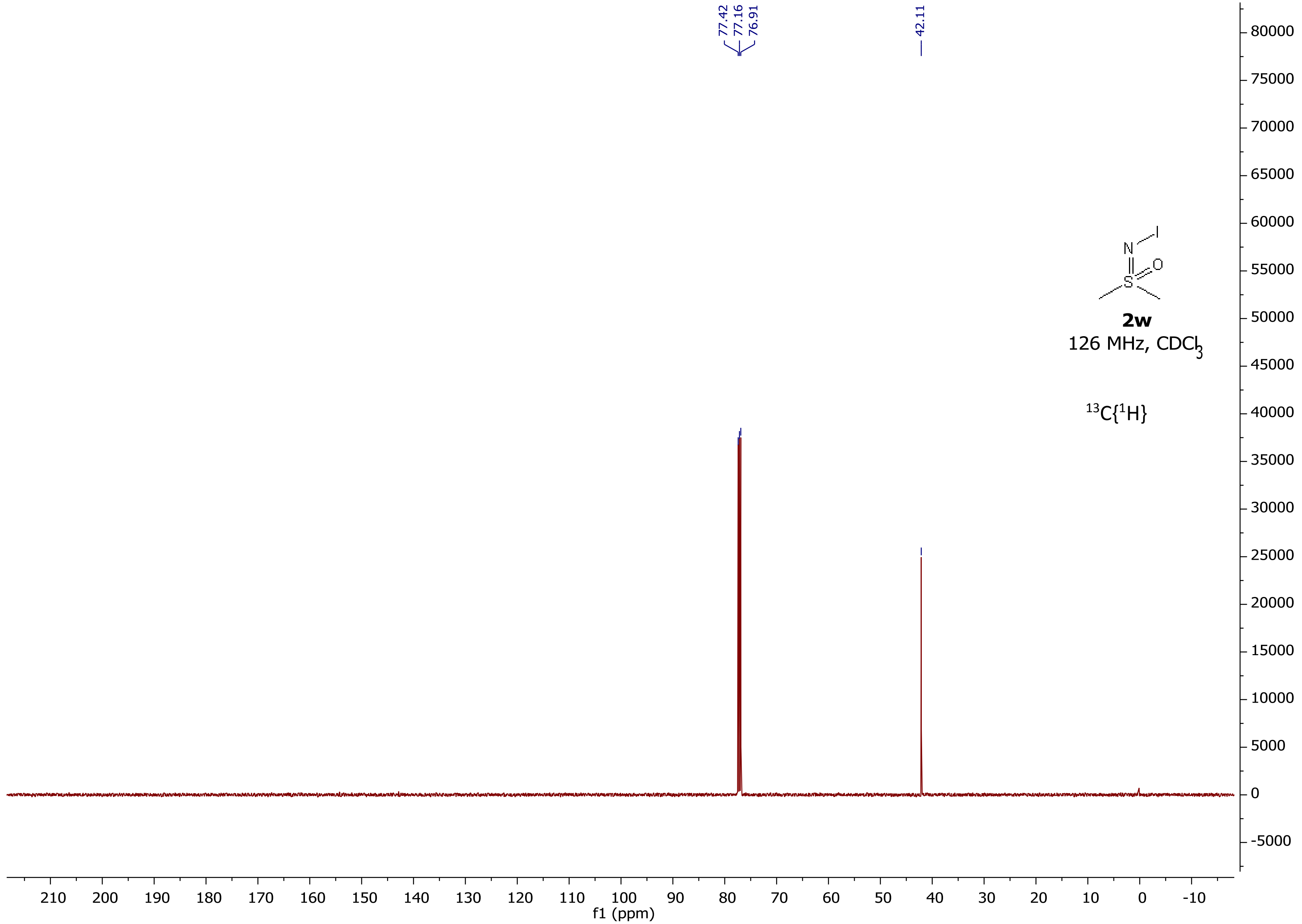
77.42
77.16
76.91
42.11
N
I
O
S
2w
126 MHz, CDCl3
13C{1H}
80000
75000
70000
65000
60000
55000
50000
45000
40000
35000
30000
25000
20000
15000
10000
5000
0
-5000
210 200 190 180 170 160 150 140 130 120 110 100 90 80 70 60 50 40 30 20 10 0 -10
f1 (ppm)

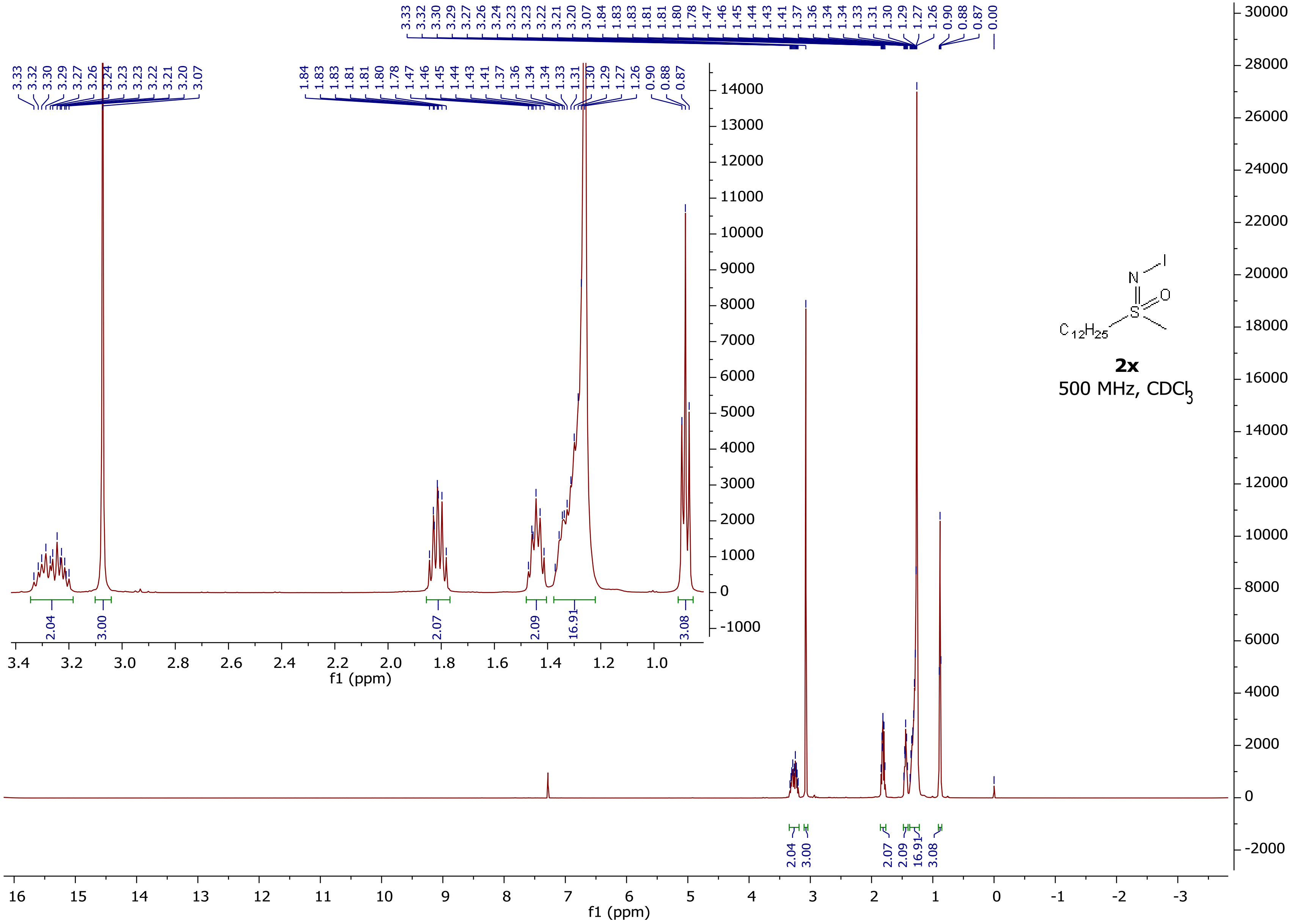

2x
500 MHz, CDCl3
C12H25
f1 (ppm)

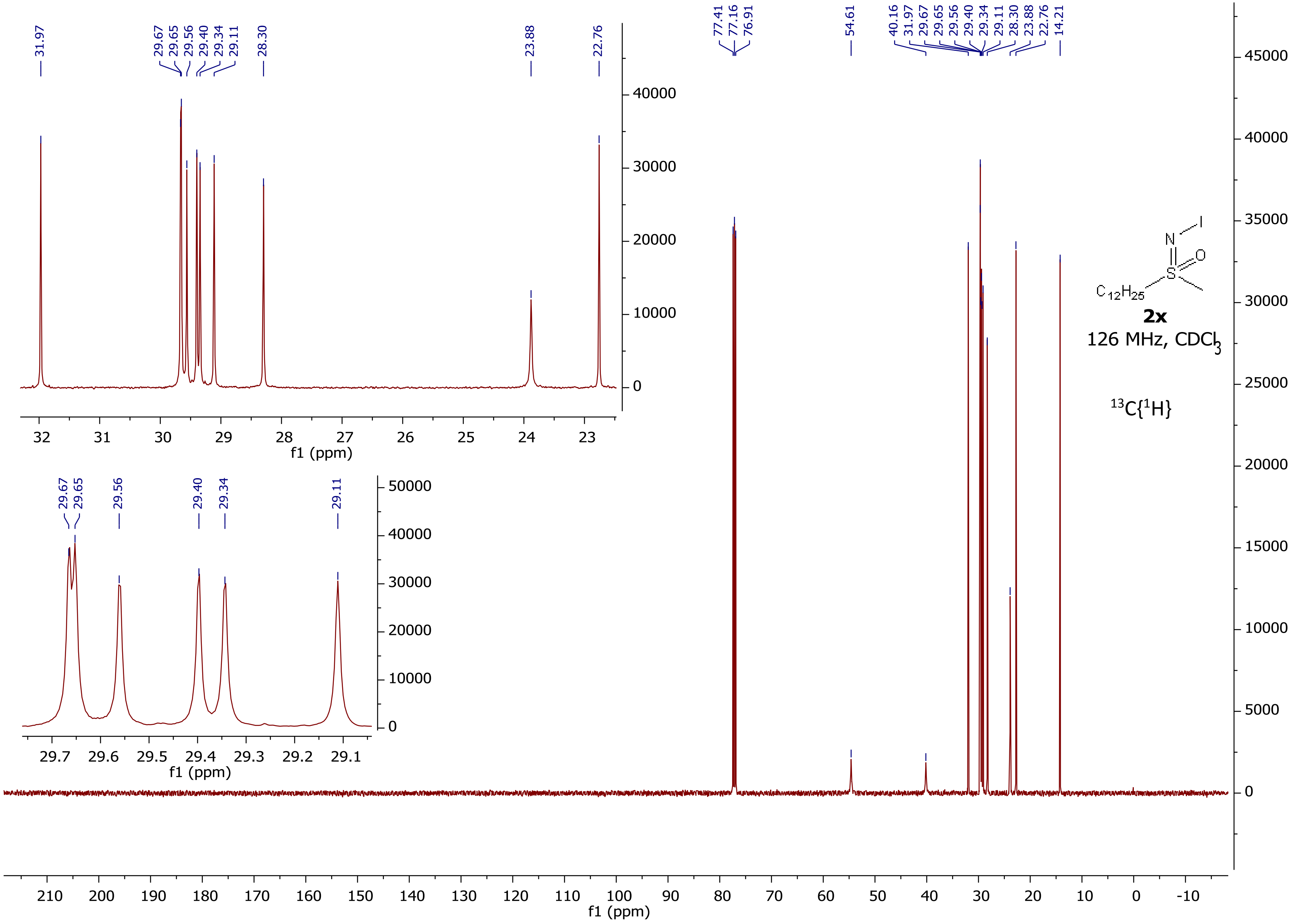

2x
126 MHz, CDCl3
13C{1H}
C12H25
N
I
S
O
77.41
77.16
76.91
54.61
40.16
31.97
29.67
29.65
29.56
29.40
29.34
29.11
28.30
23.88
22.76
14.21
f1 (ppm)

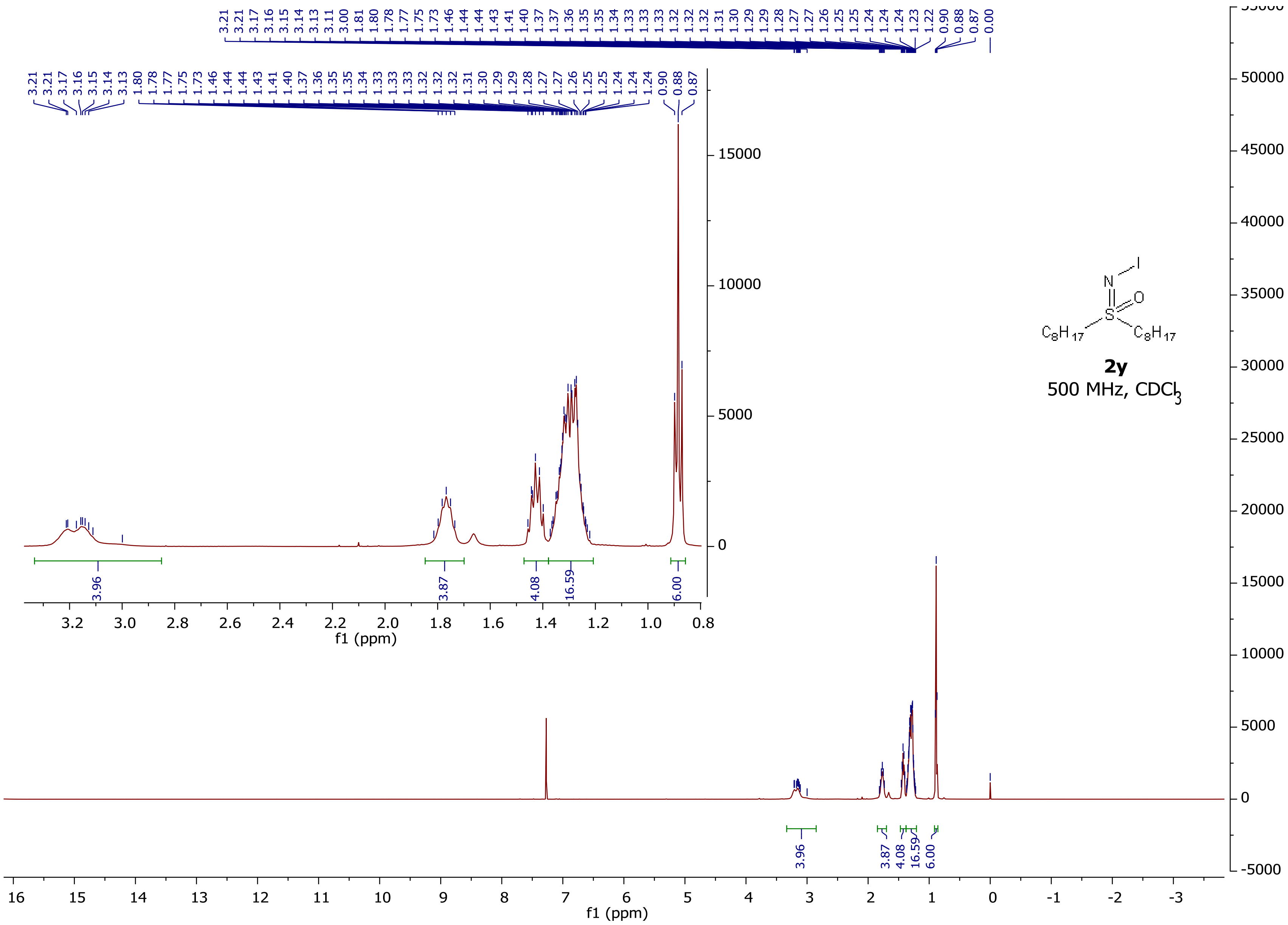
2y
500 MHz, CDCl3
f1 (ppm)

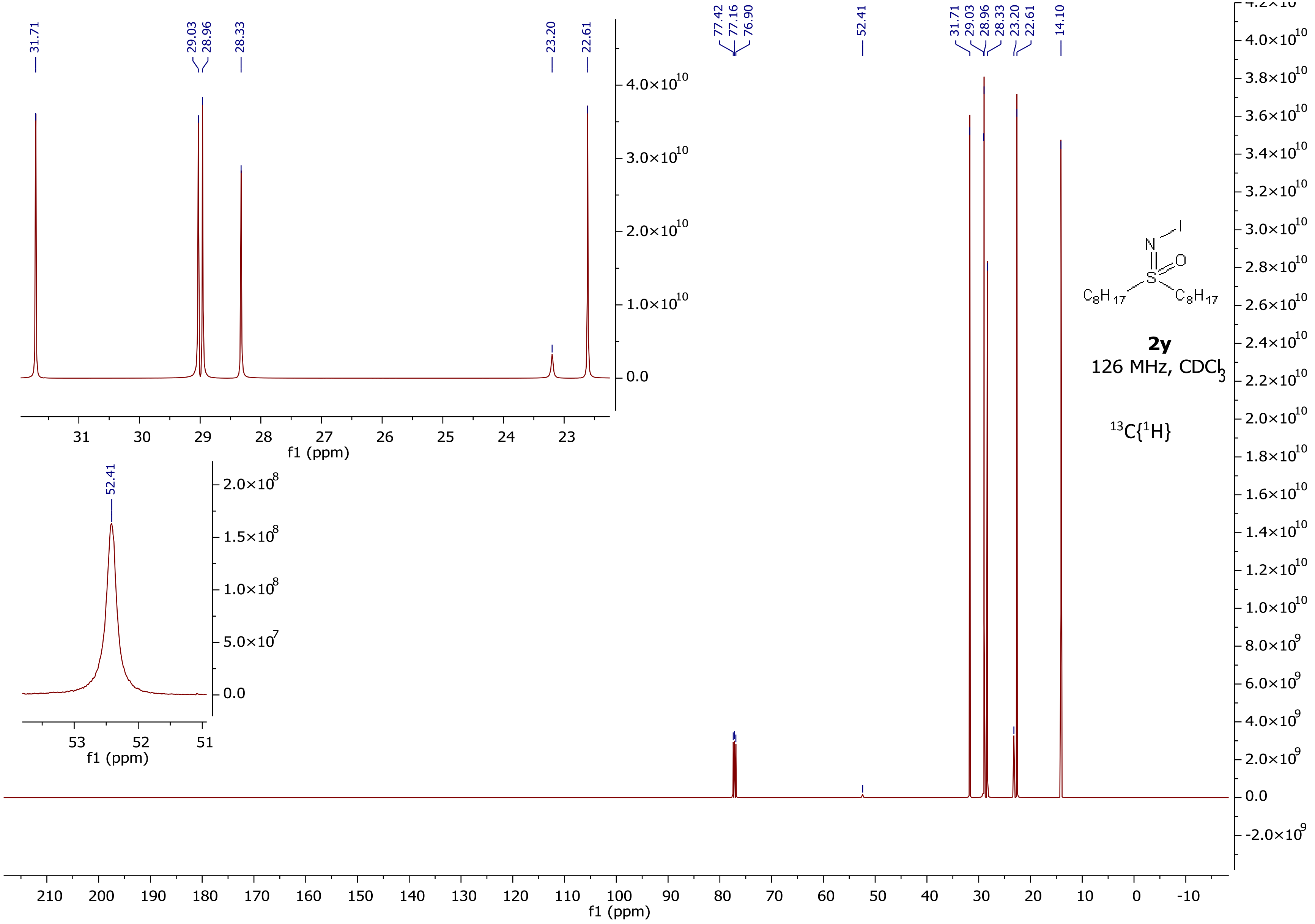

2y
126 MHz, CDCl3
13C{1H}
77.42
77.16
76.90
52.41
31.71
29.03
28.96
28.33
23.20
22.61
14.10
f1 (ppm)

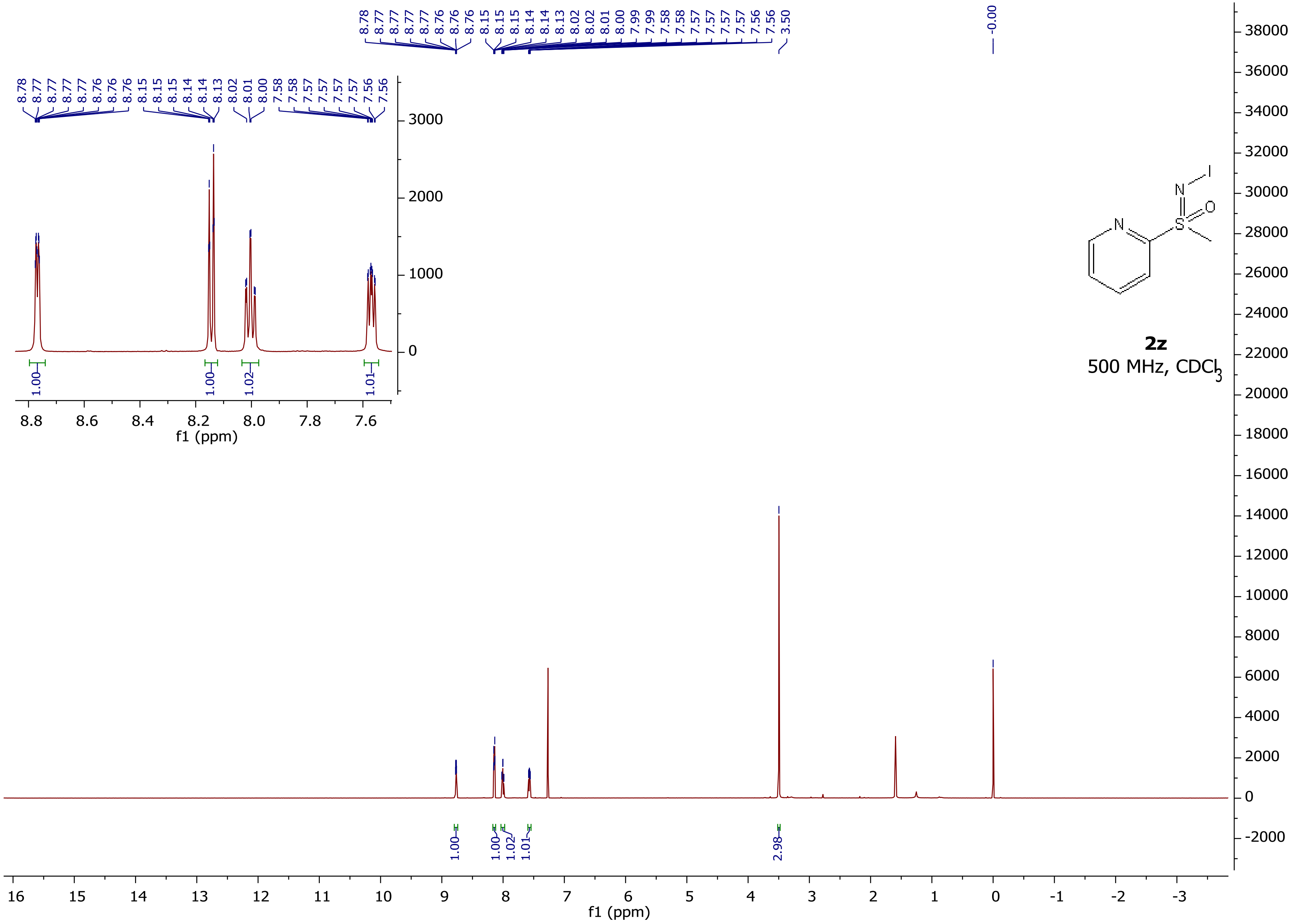
2z
500 MHz, CDCl3
f1 (ppm)

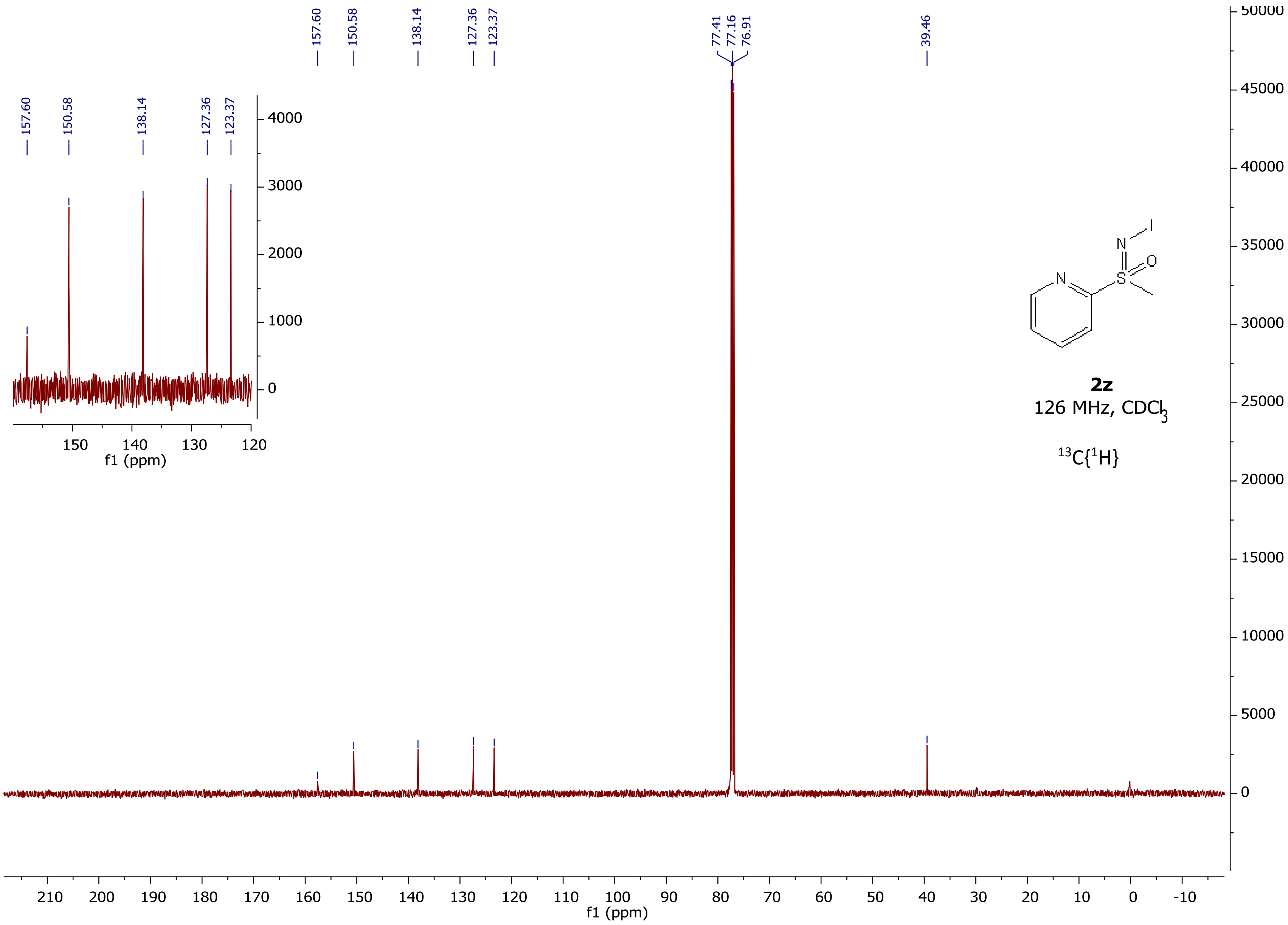
157.60
150.58
138.14
127.36
123.37
77.41
77.16
76.91
39.46
f1 (ppm)
2z
126 MHz, CDCl3
13C{1H}
N
S
O
I

^{1}H and $^{13}C\{^{1}H\}$ NMR spectra of *N*-bromo and *N*-chloro sulfoximines **3** and **4**

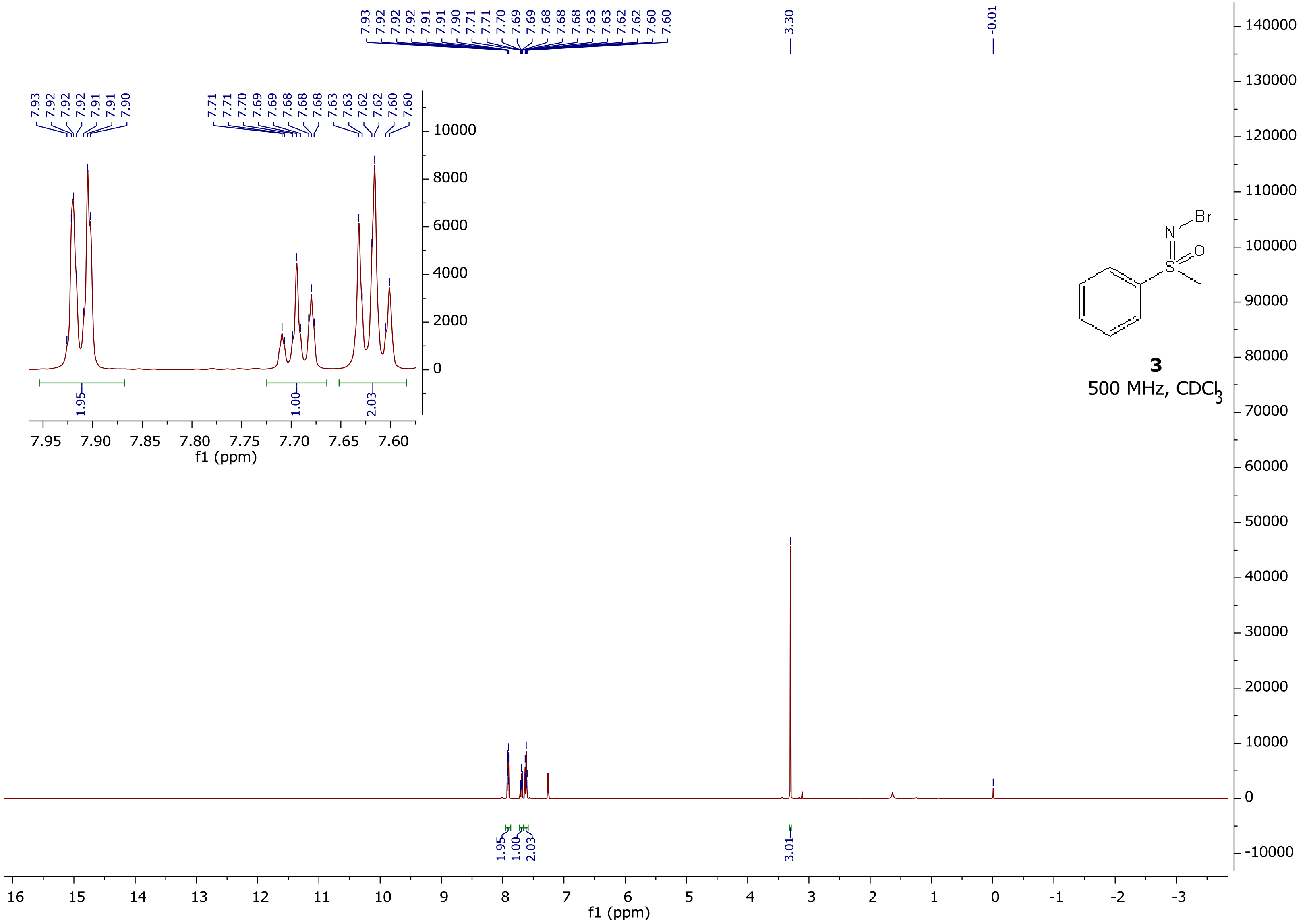

3
500 MHz, $CDCl_3$

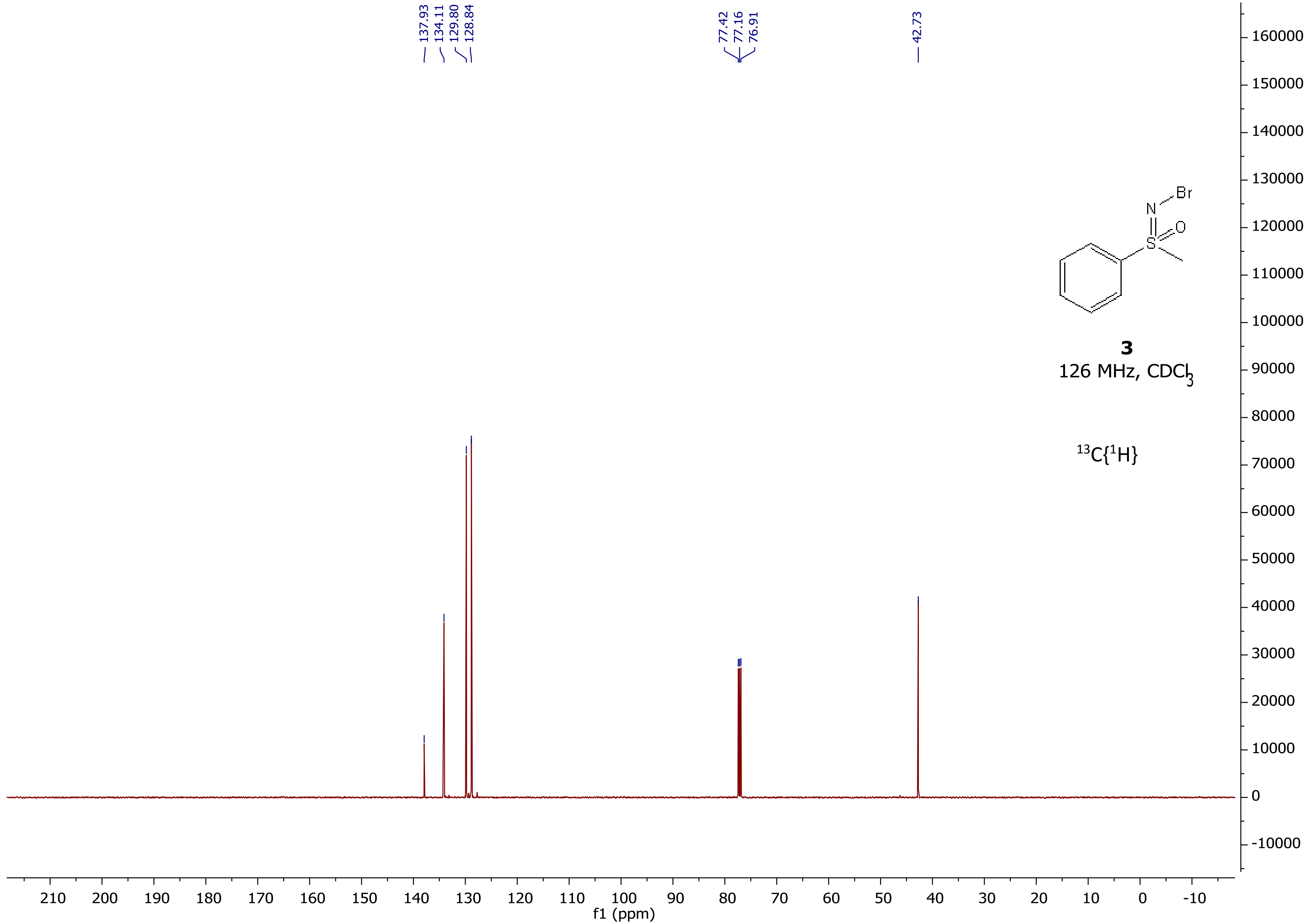
137.93
134.11
129.80
128.84
77.42
77.16
76.91
42.73
N
Br
S
O
3
126 MHz, CDCl3
13C{1H}
160000
150000
140000
130000
120000
110000
100000
90000
80000
70000
60000
50000
40000
30000
20000
10000
0
-10000
210 200 190 180 170 160 150 140 130 120 110 100 90 80 70 60 50 40 30 20 10 0 -10
f1 (ppm)

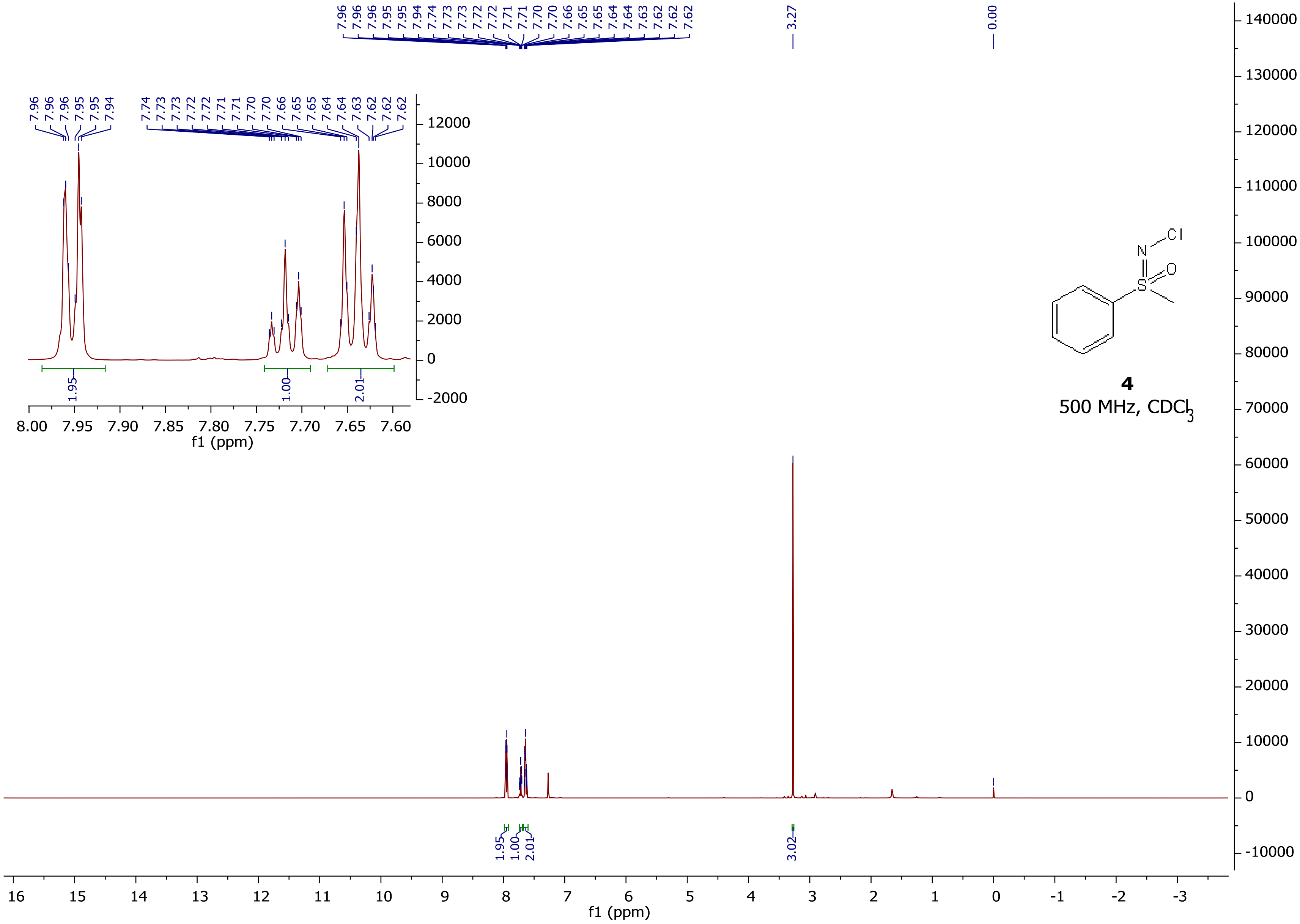

4
500 MHz, $CDCl_3$

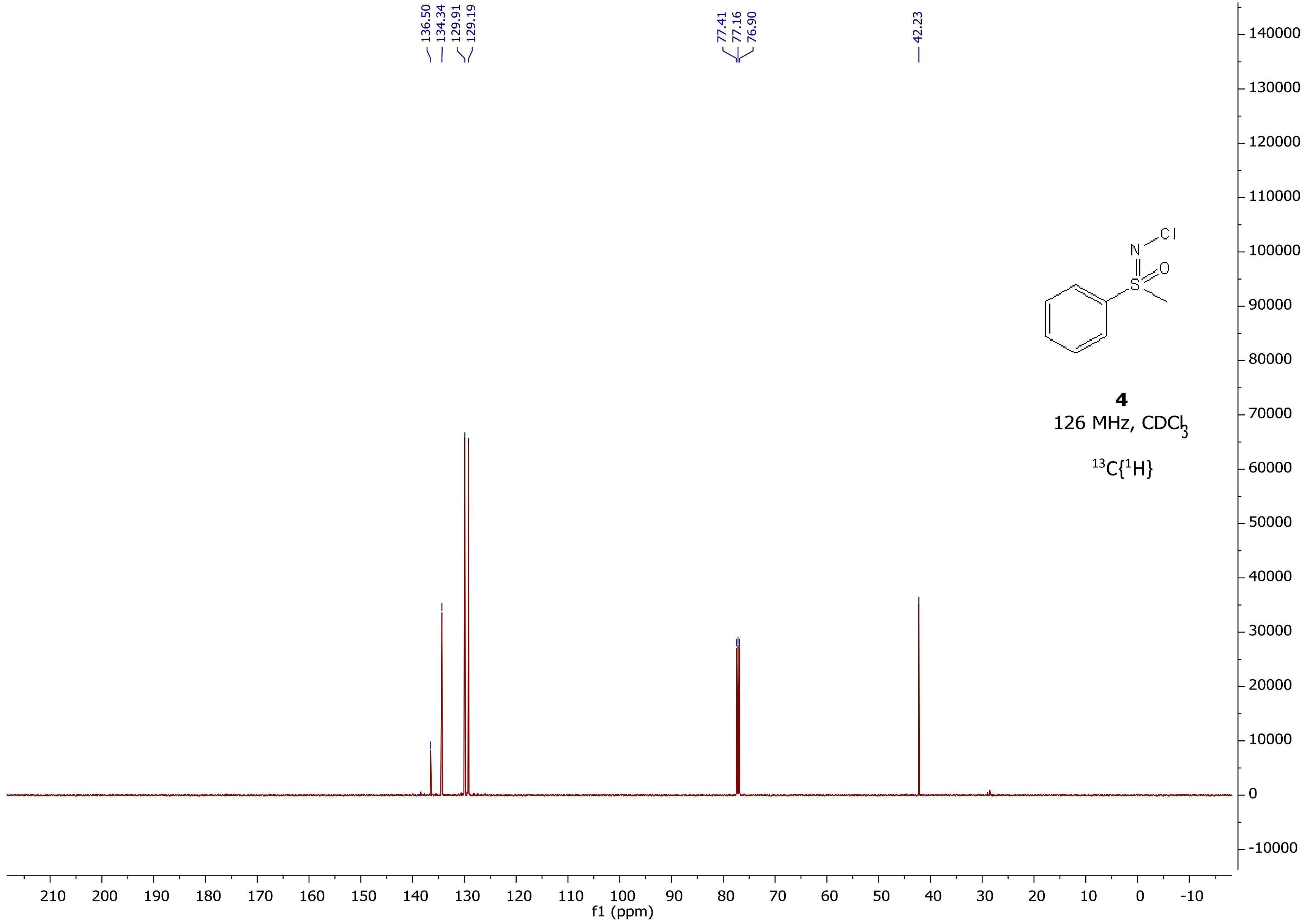
136.50
134.34
129.91
129.19
77.41
77.16
76.90
42.23
N
Cl
S
O
4
126 MHz, CDCl3
13C{1H}
f1 (ppm)

^{1}H and $^{13}C\{^{1}H\}$ NMR spectra of iodinated and oxidized products **6**, **8**, **10**, **12** and **14**

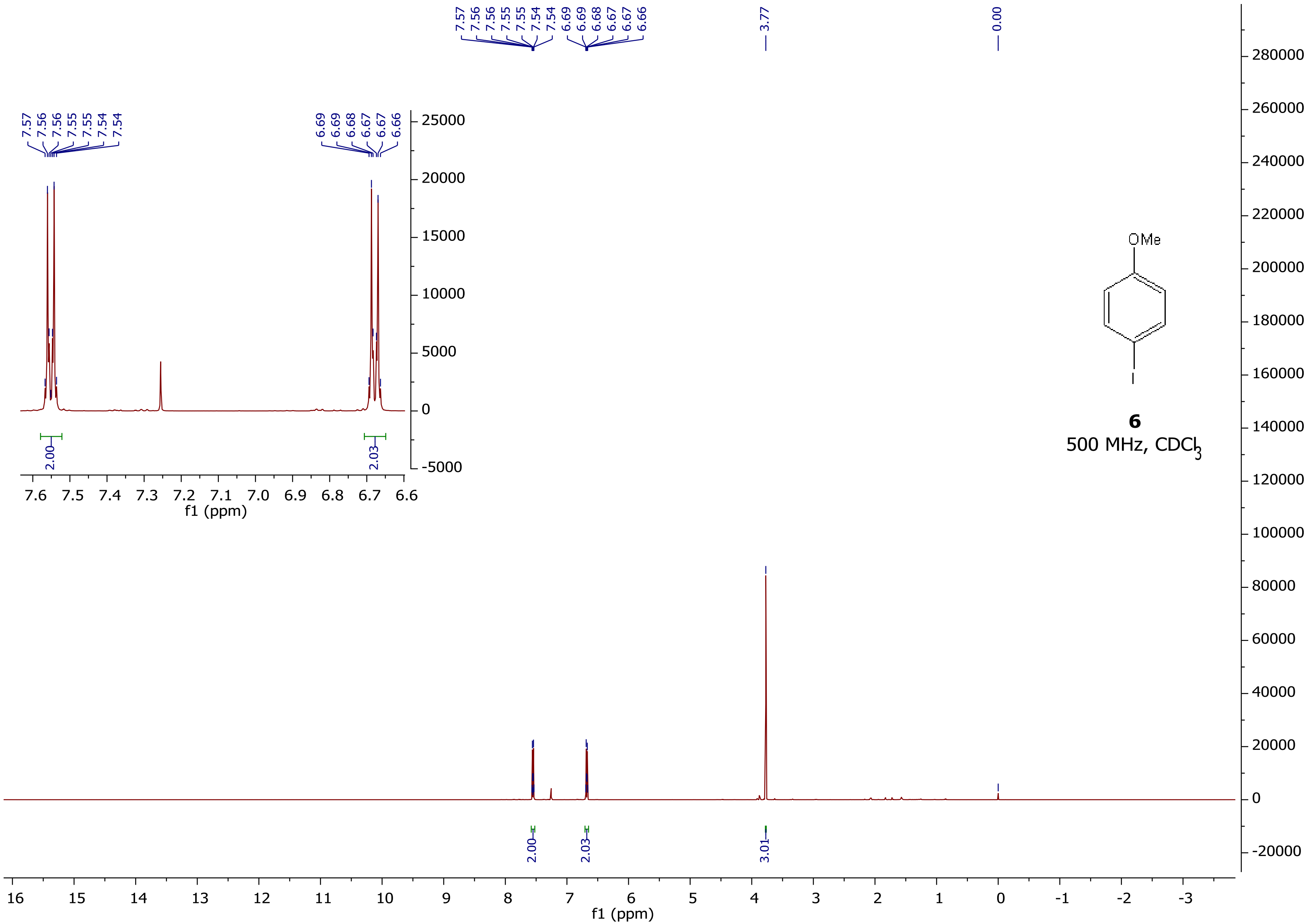

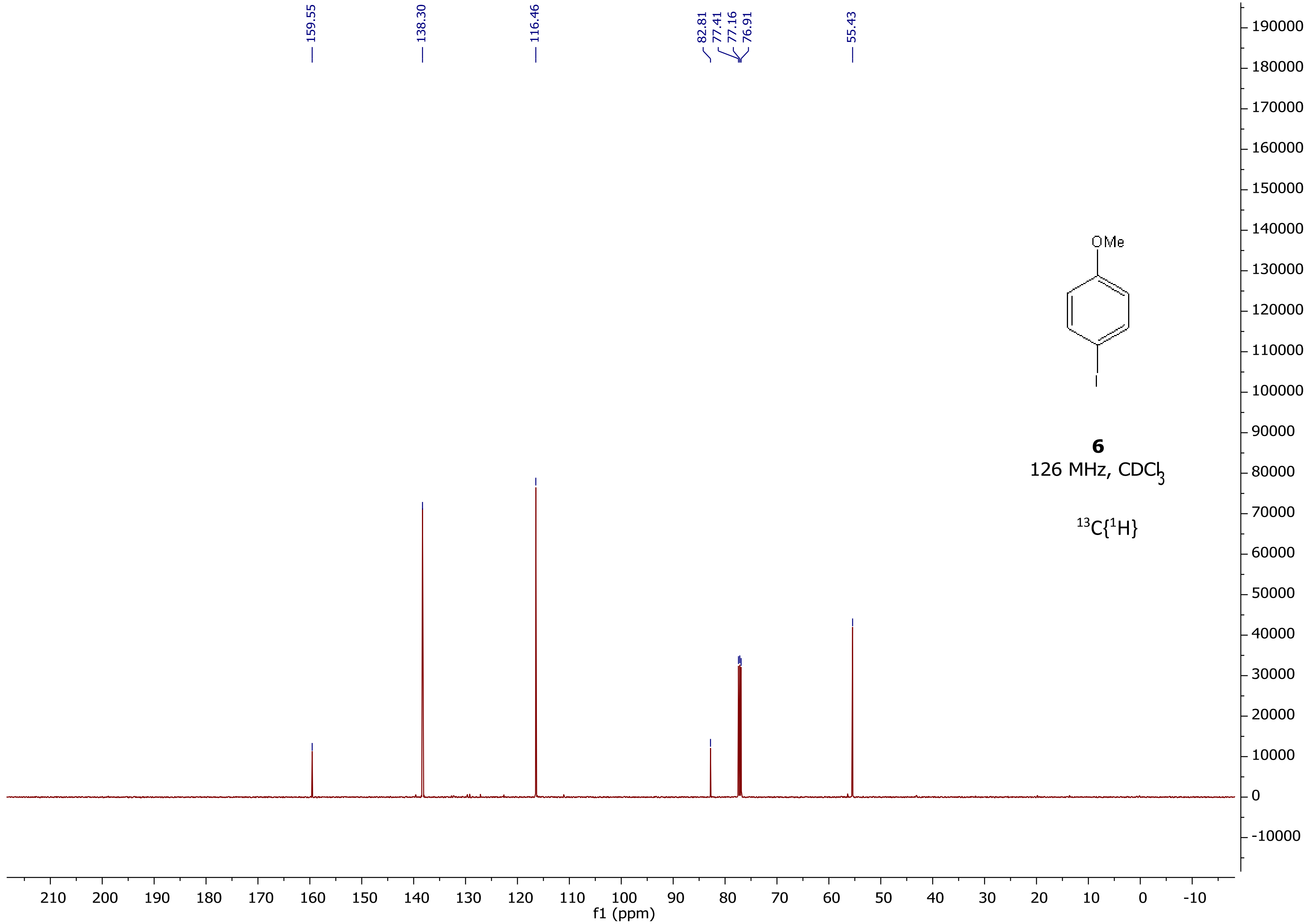
159.55
138.30
116.46
82.81
77.41
77.16
76.91
55.43
OMe
I
6
126 MHz, CDCl3
13C{1H}
f1 (ppm)

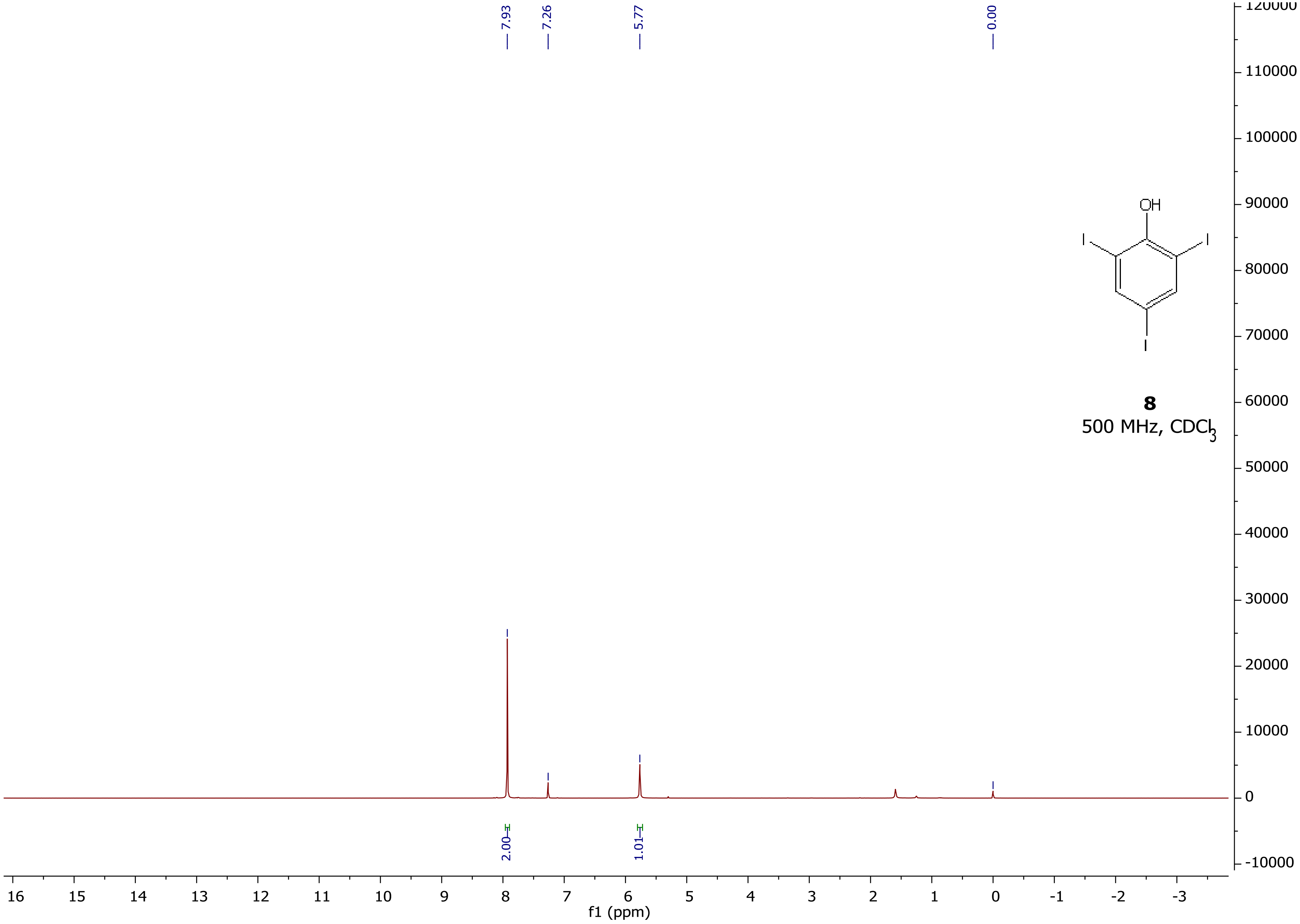
7.93
7.26
5.77
0.00
OH
I
I
I
8
500 MHz, CDCl3
2.00
1.01
f1 (ppm)

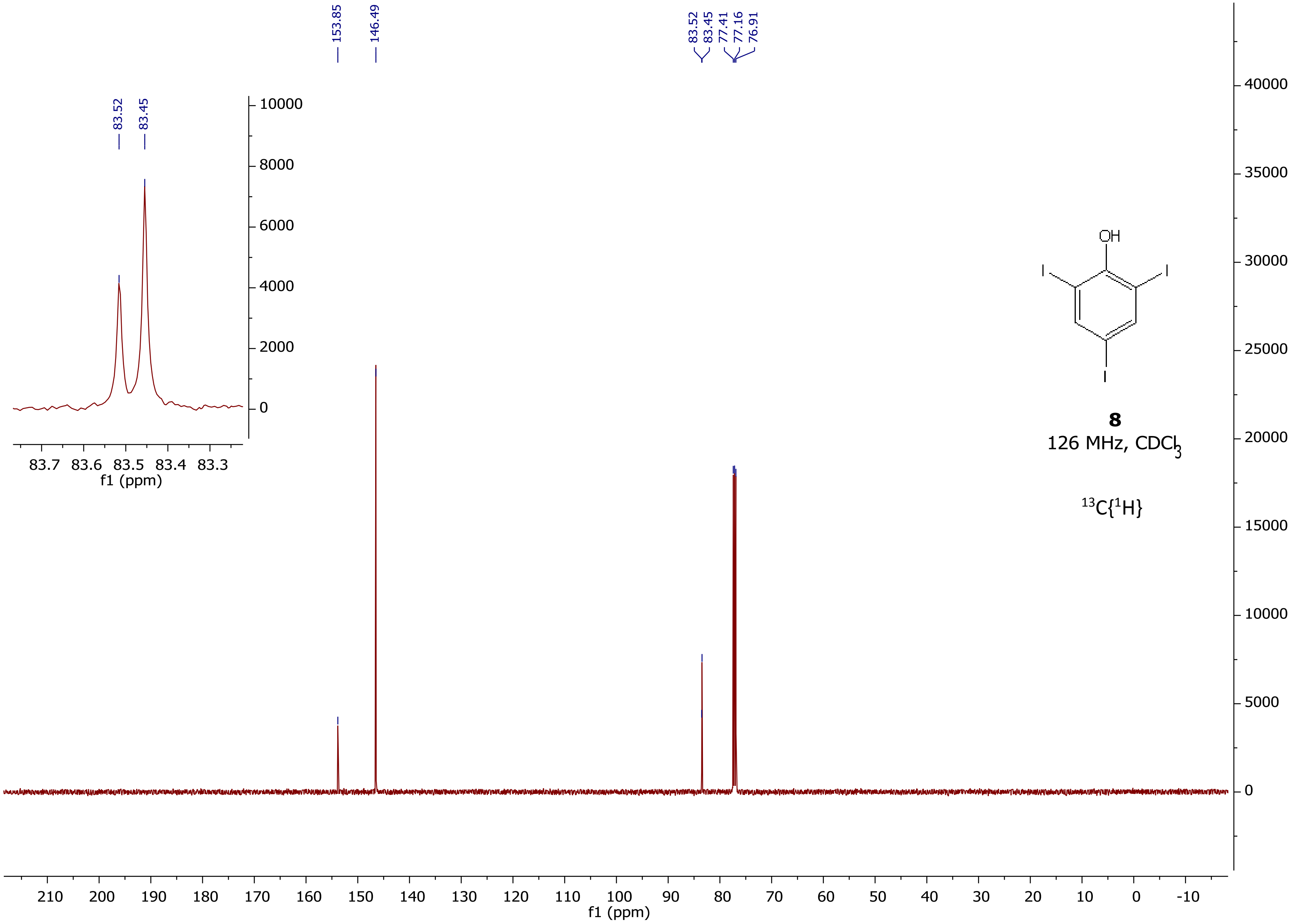
153.85
146.49
83.52
83.45
77.41
77.16
76.91
OH
I
I
I
8
126 MHz, CDCl3
13C{1H}
f1 (ppm)
83.7 83.6 83.5 83.4 83.3
10000
8000
6000
4000
2000
0
40000
35000
30000
25000
20000
15000
10000
5000
210 200 190 180 170 160 150 140 130 120 110 100 90 80 70 60 50 40 30 20 10 0 -10

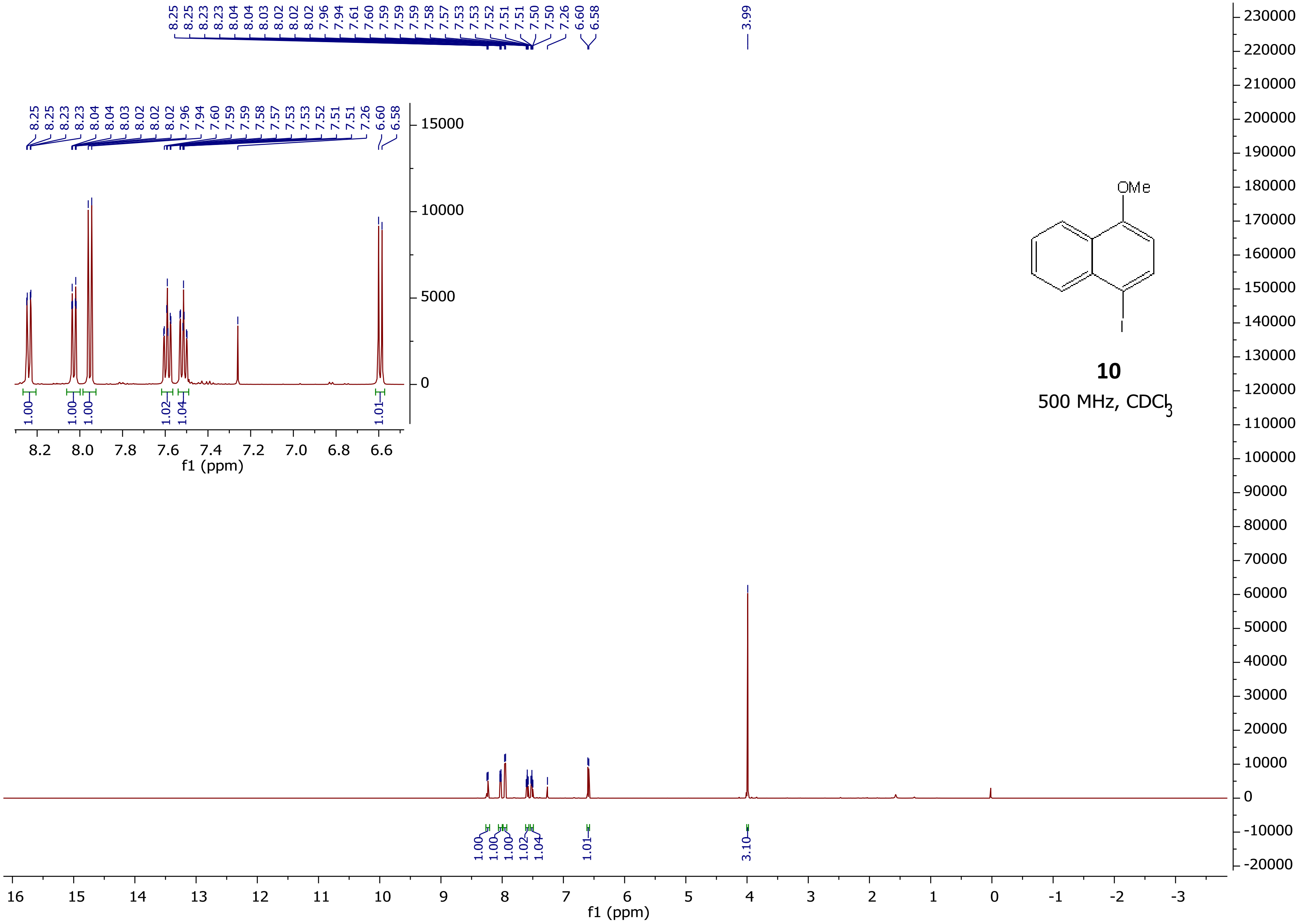
OMe
I
10
500 MHz, CDCl3
f1 (ppm)
3.99
3.10
1.00
1.00
1.00
1.02
1.04
1.01

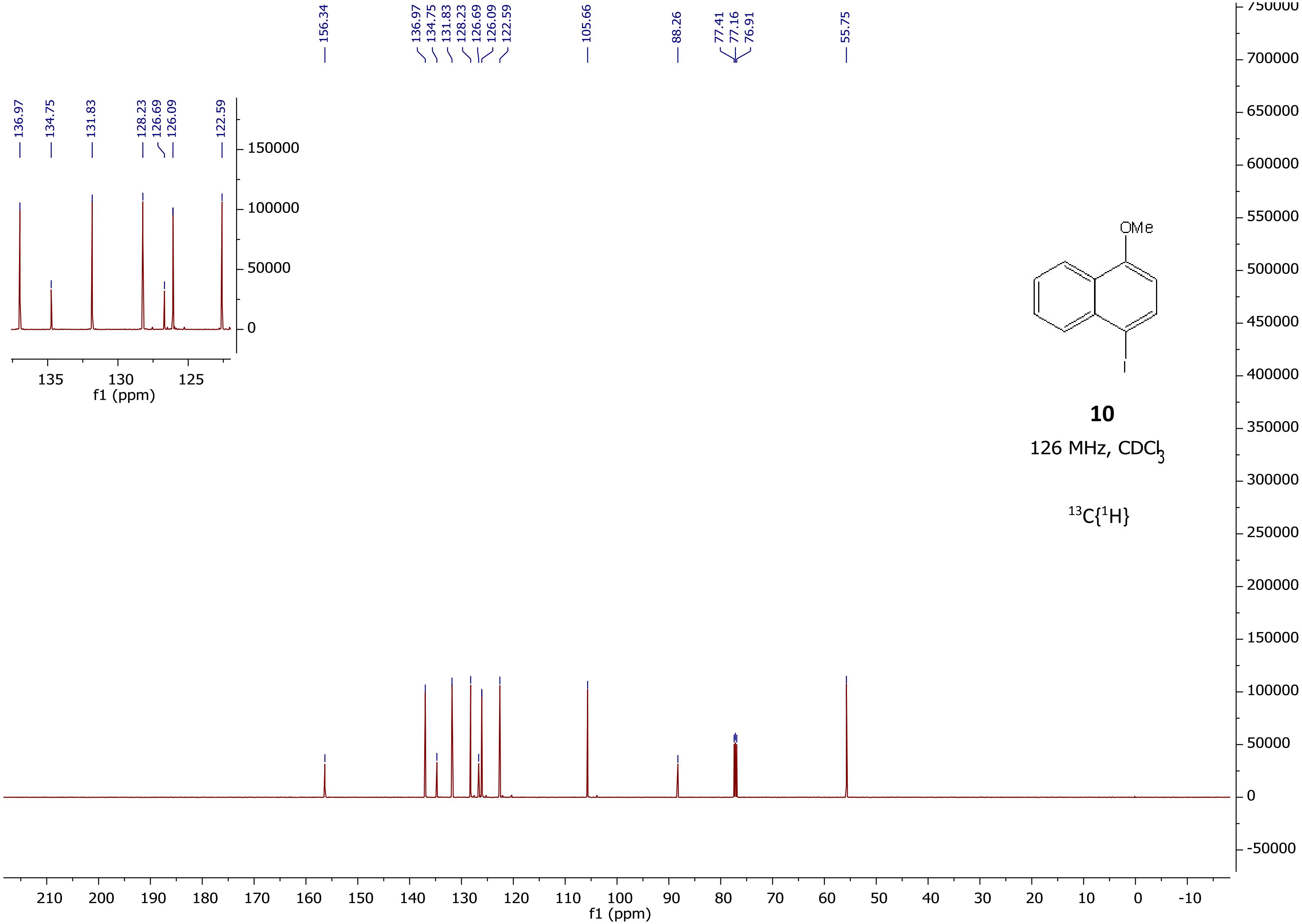

10

126 MHz, $CDCl_3$

$^{13}C\{^1H\}$

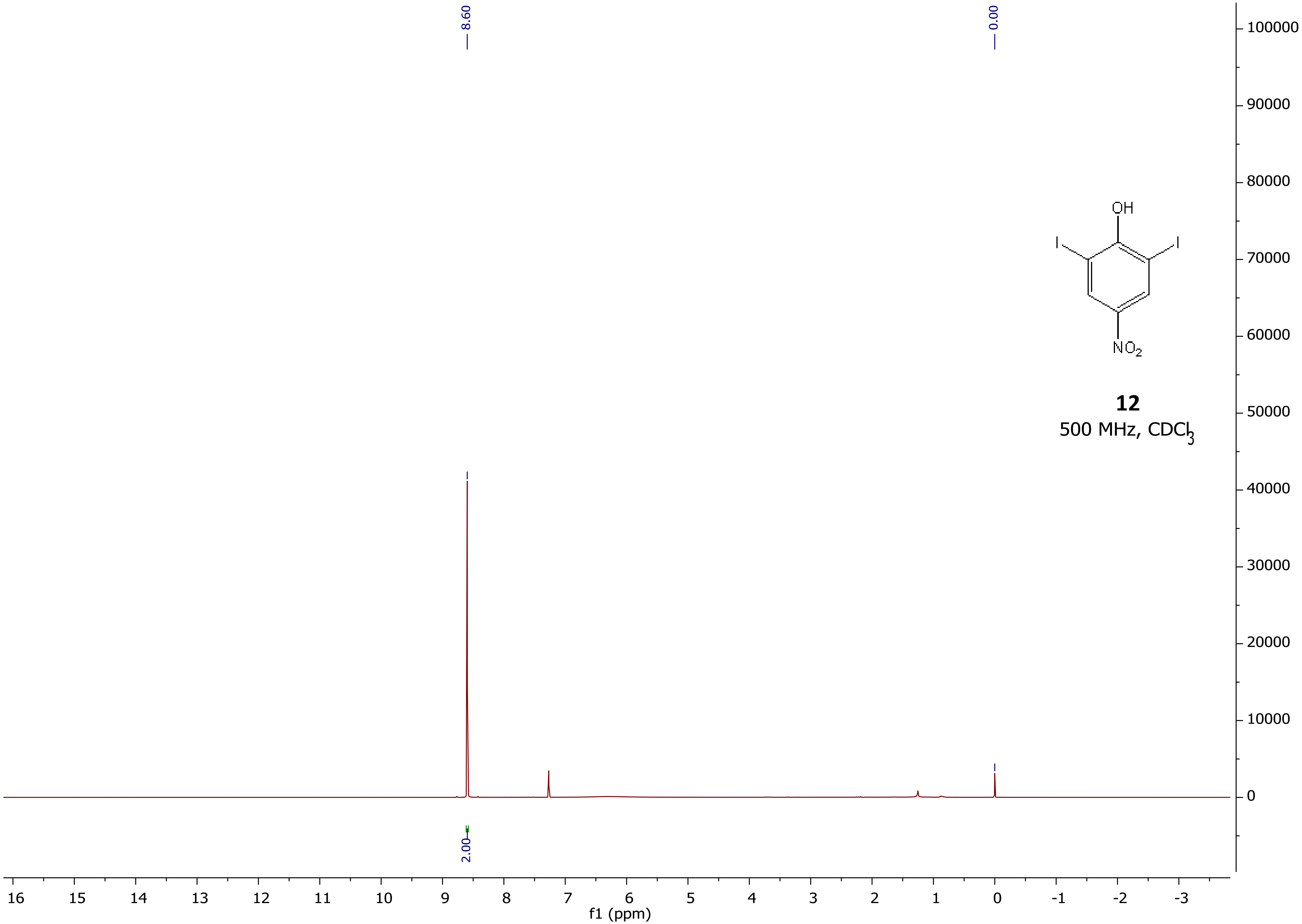
8.60
0.00
OH
I
I
NO2
12
500 MHz, CDCl3
2.00
f1 (ppm)

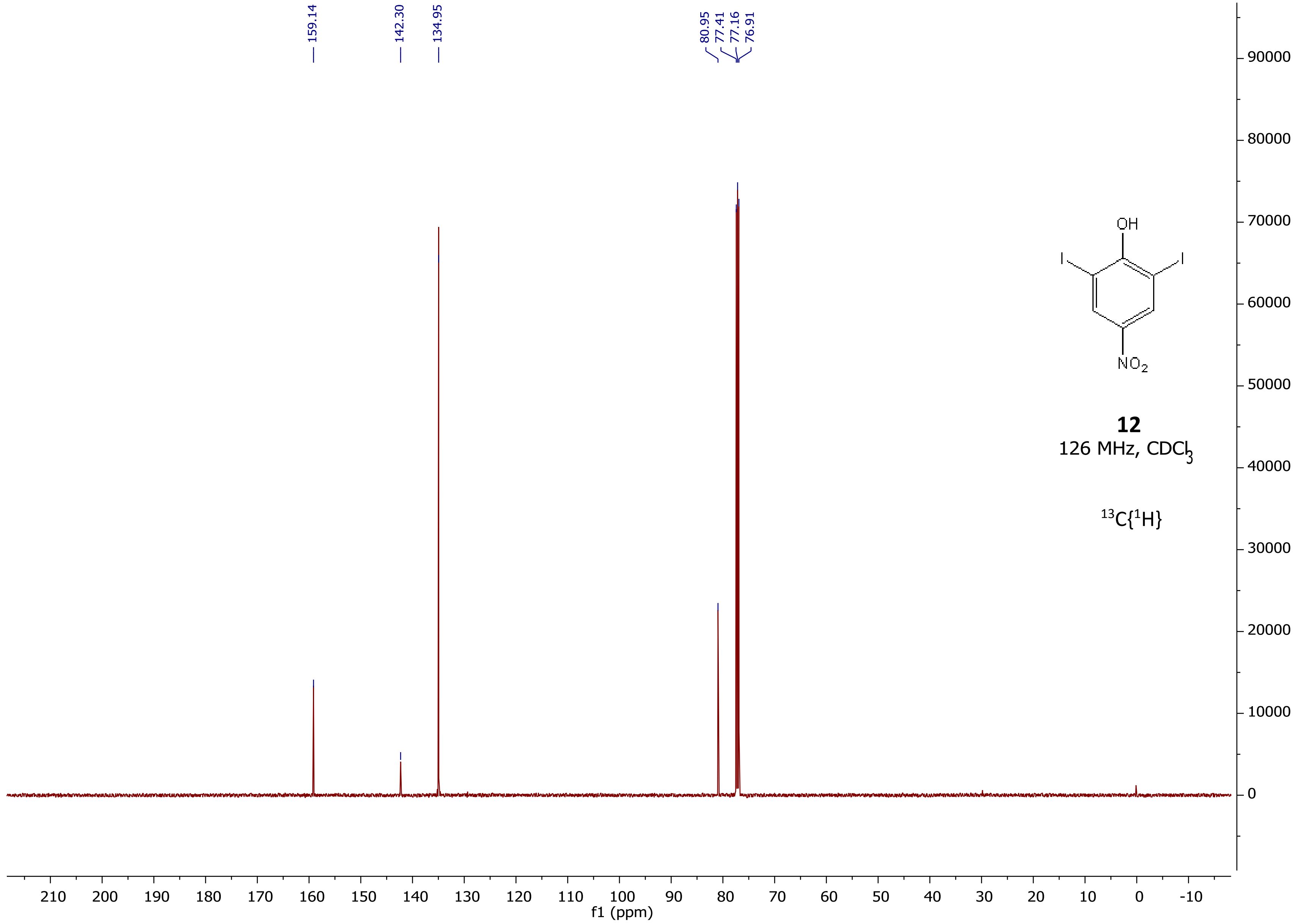
159.14
142.30
134.95
80.95
77.41
77.16
76.91
OH
I
I
NO2
12
126 MHz, CDCl3
13C{1H}
f1 (ppm)

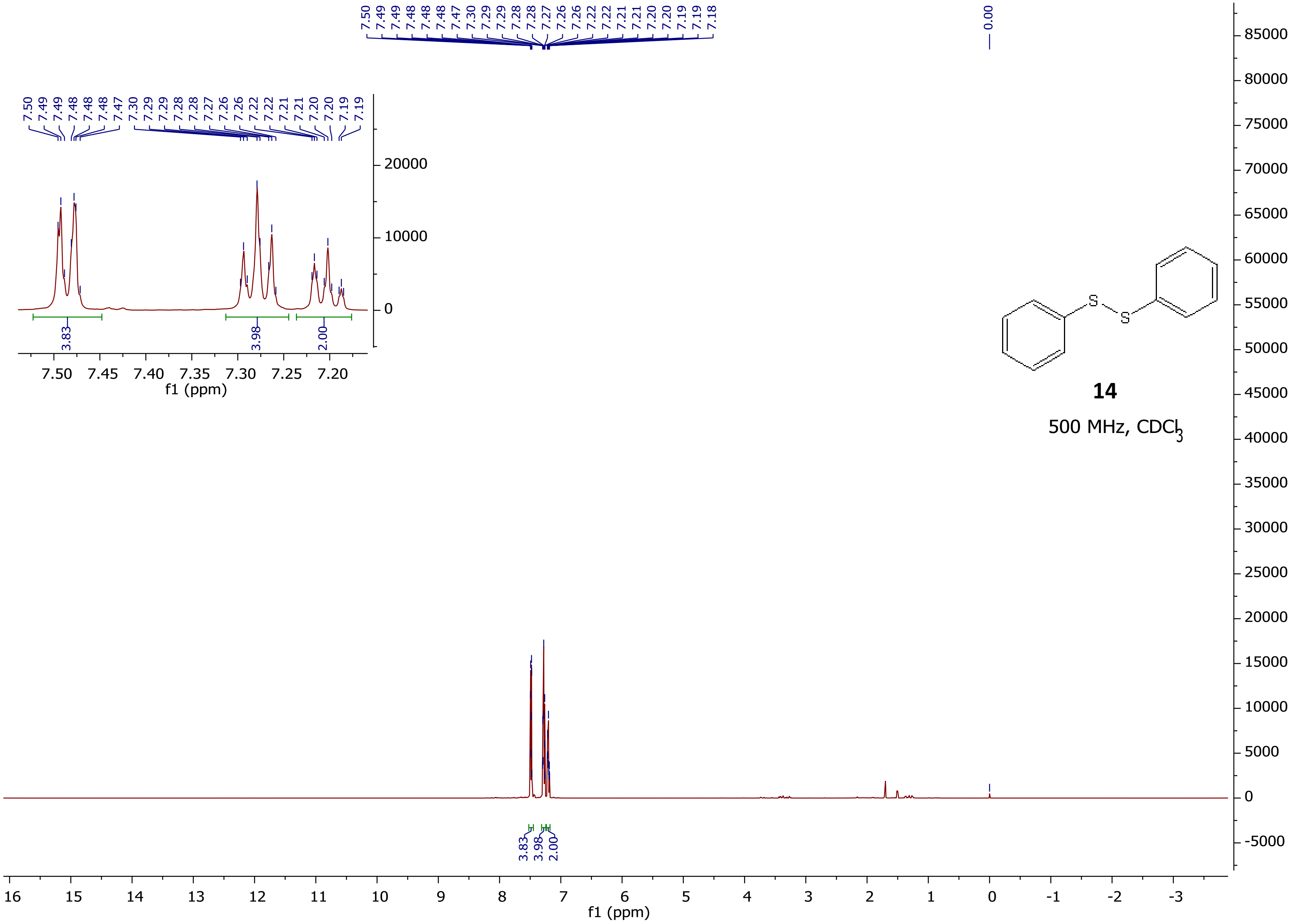

14

500 MHz, $CDCl_3$

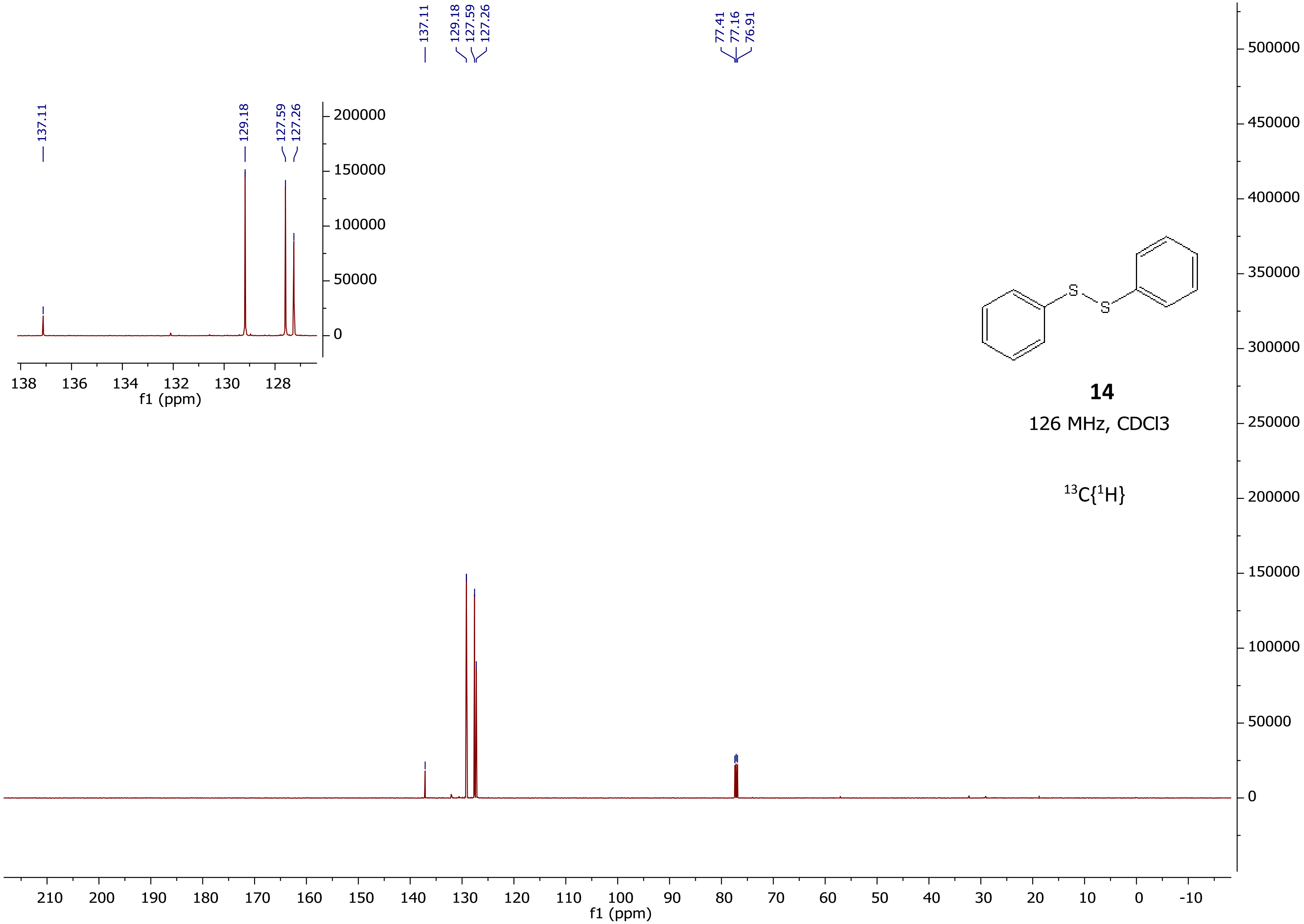
137.11
129.18
127.59
127.26
77.41
77.16
76.91
14
126 MHz, CDCl3
$^{13}C\{^{1}H\}$
f1 (ppm)

^{1}H, $^{13}C\{^{1}H\}$ and ^{19}F NMR spectra of *N*-(trifluoromethanesulfenyl) sulfoximines **15**

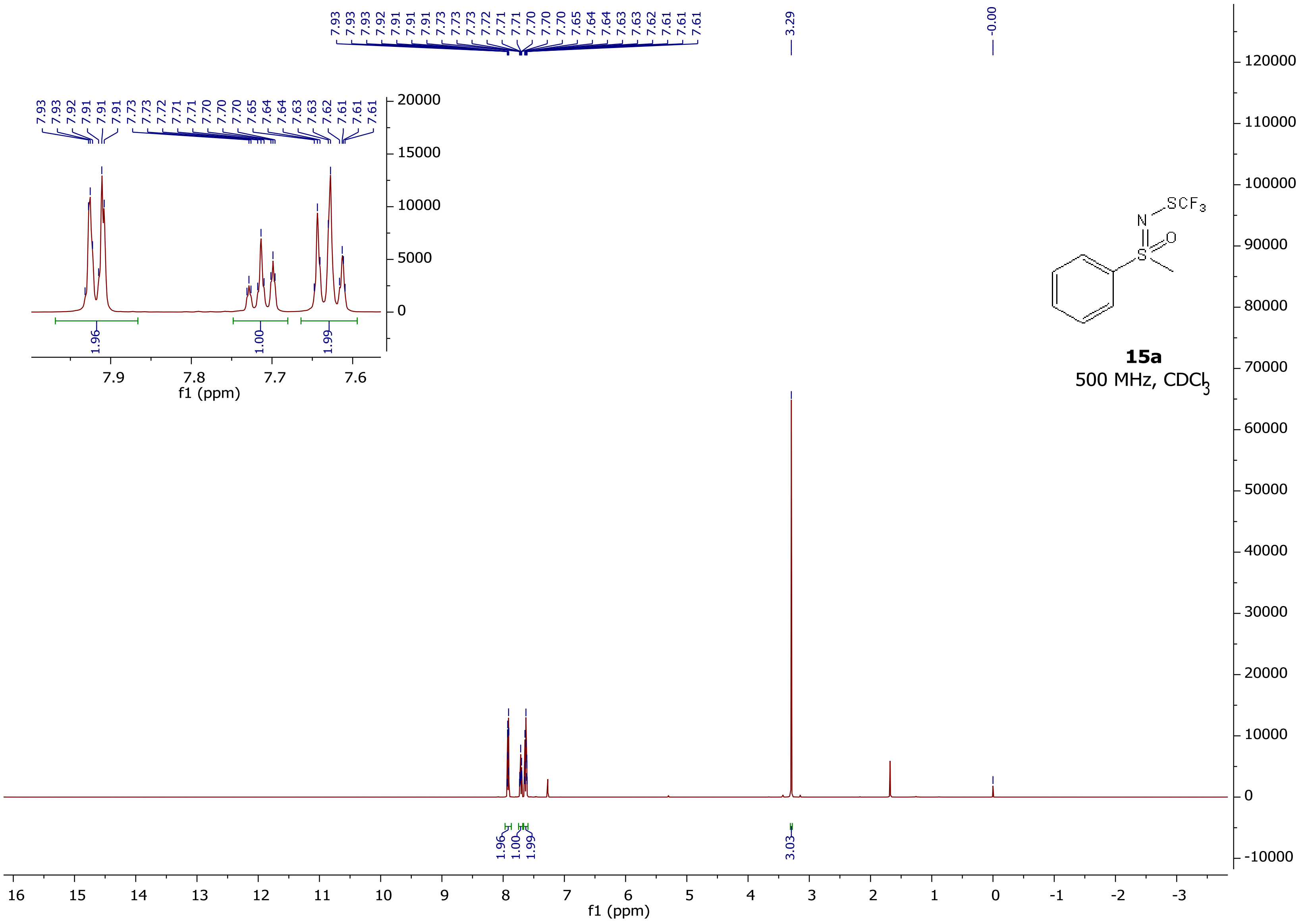

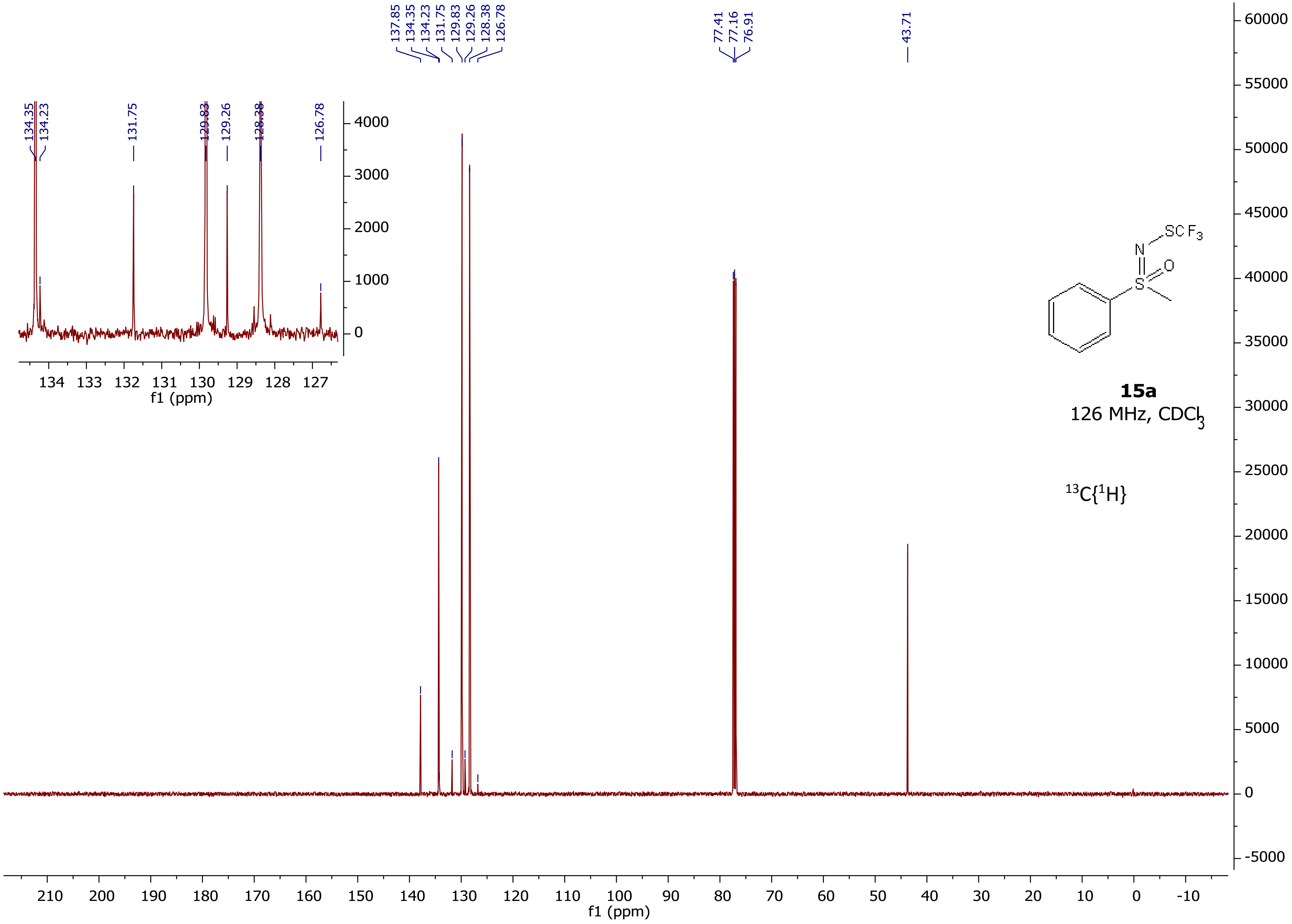
137.85
134.35
134.23
131.75
129.83
129.26
128.38
126.78
77.41
77.16
76.91
43.71
15a
126 MHz, CDCl3
13C{1H}
f1 (ppm)

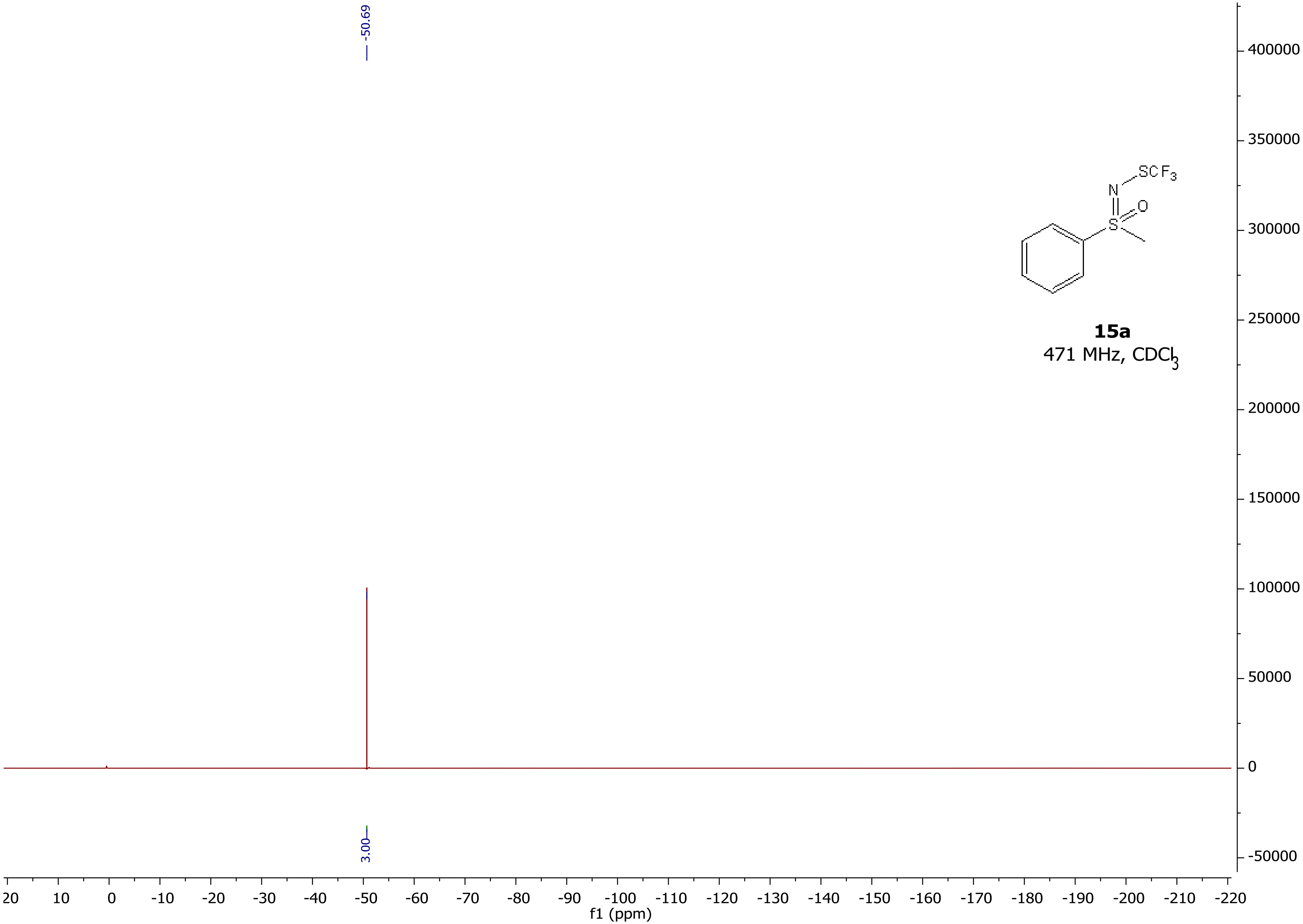

-50.69
SCF3
N
O
S
15a
471 MHz, CDCl3
3.00
f1 (ppm)

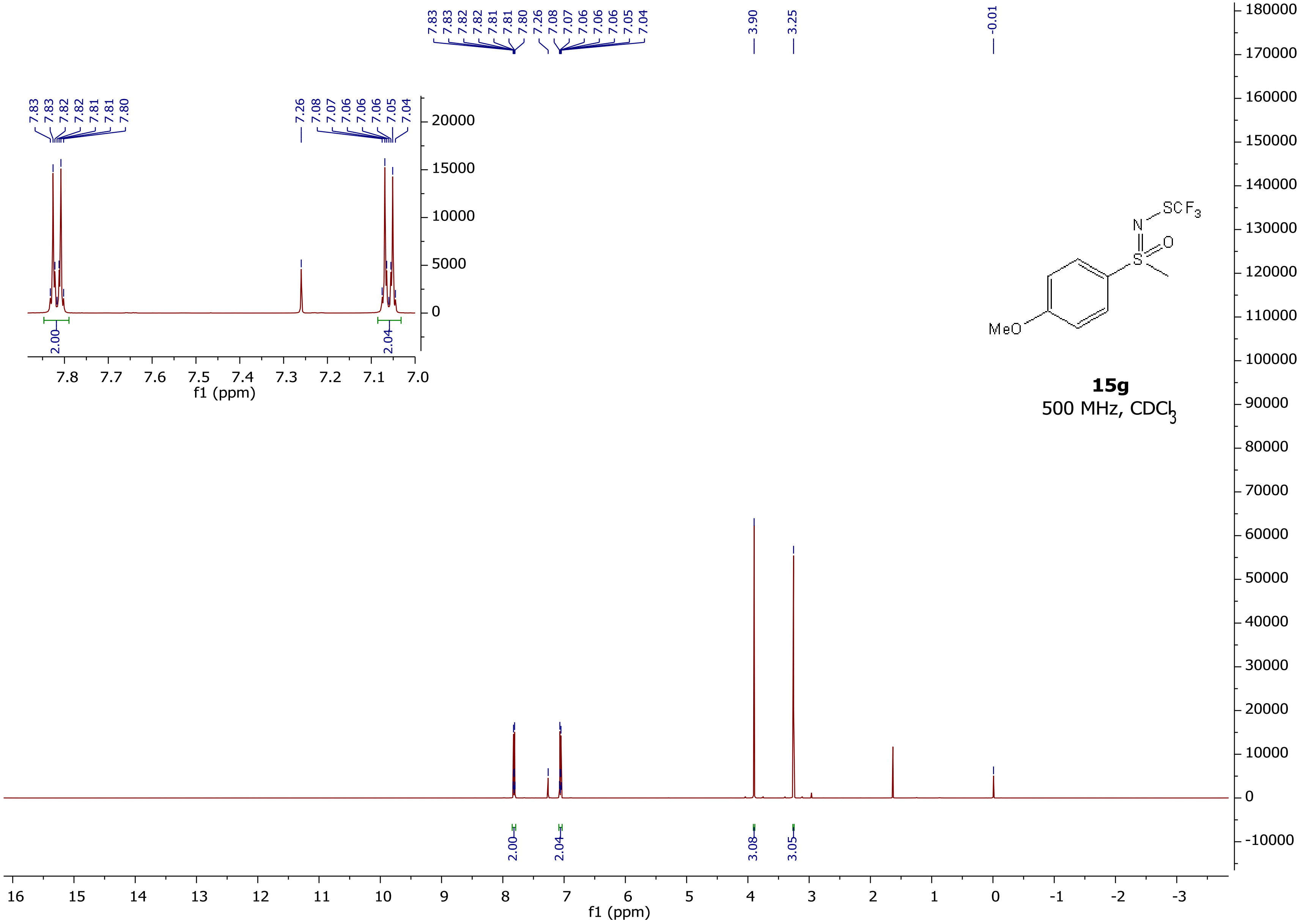

7.83
7.83
7.82
7.82
7.81
7.81
7.80
7.26
7.08
7.07
7.06
7.06
7.06
7.05
7.04
3.90
3.25
-0.01
2.00
2.04
3.08
3.05
f1 (ppm)
SCF3
N
S
O
MeO
15g
500 MHz, CDCl3

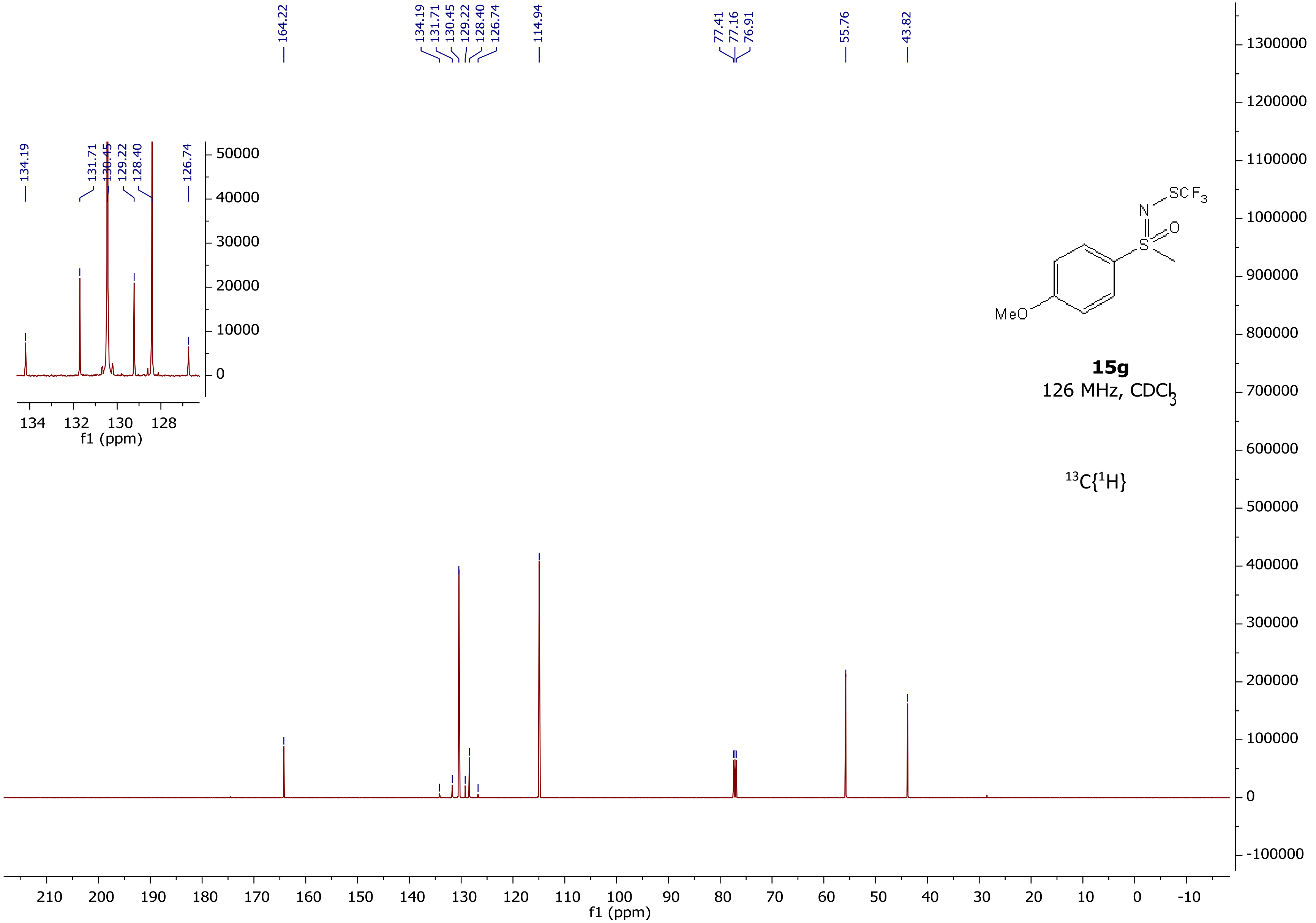
164.22
134.19
131.71
130.45
129.22
128.40
126.74
114.94
77.41
77.16
76.91
55.76
43.82
SCF3
N
O
S
MeO
15g
126 MHz, CDCl3
13C{1H}
f1 (ppm)

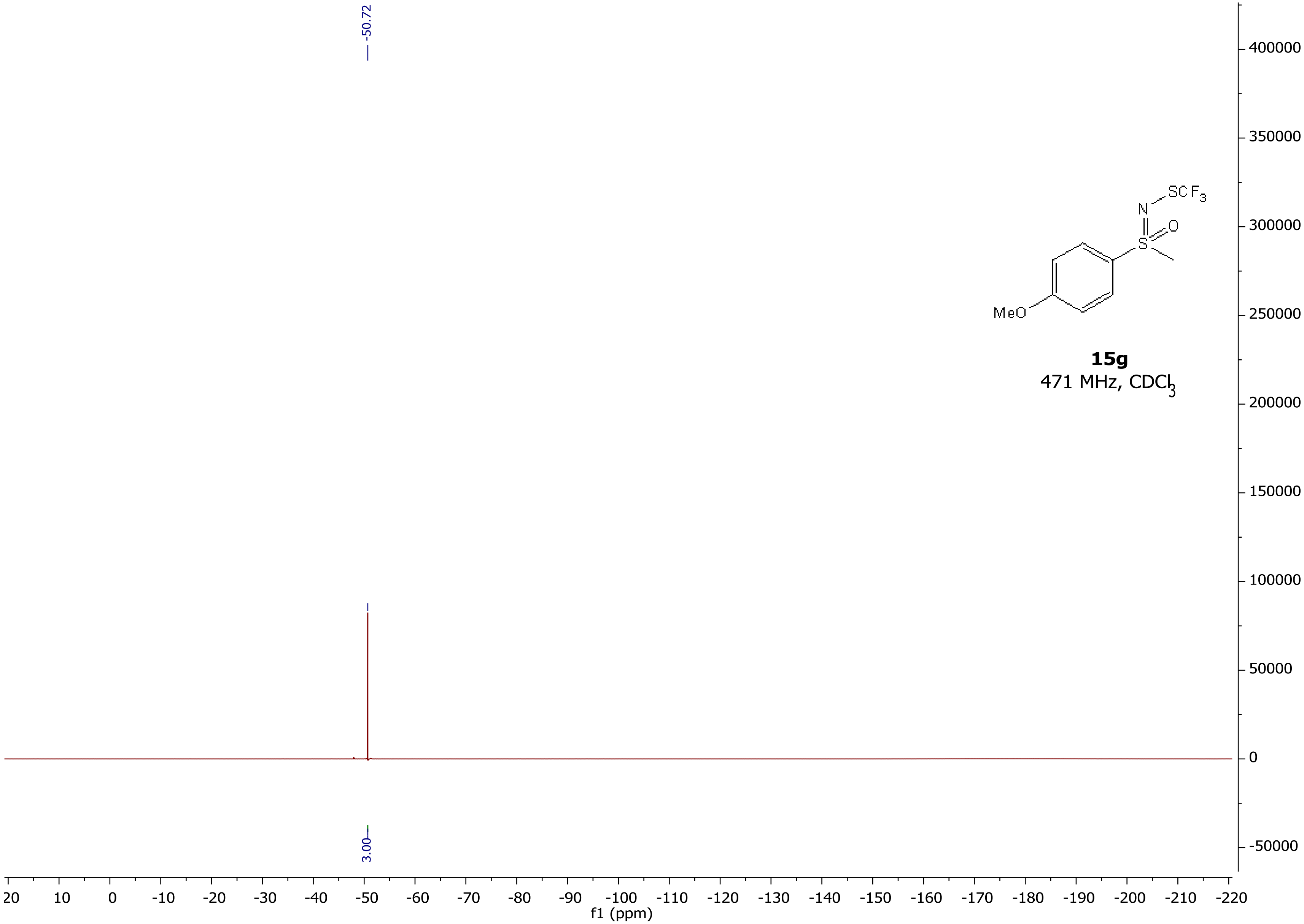

-50.72
SCF3
N
S
O
MeO
15g
471 MHz, CDCl3
3.00
f1 (ppm)

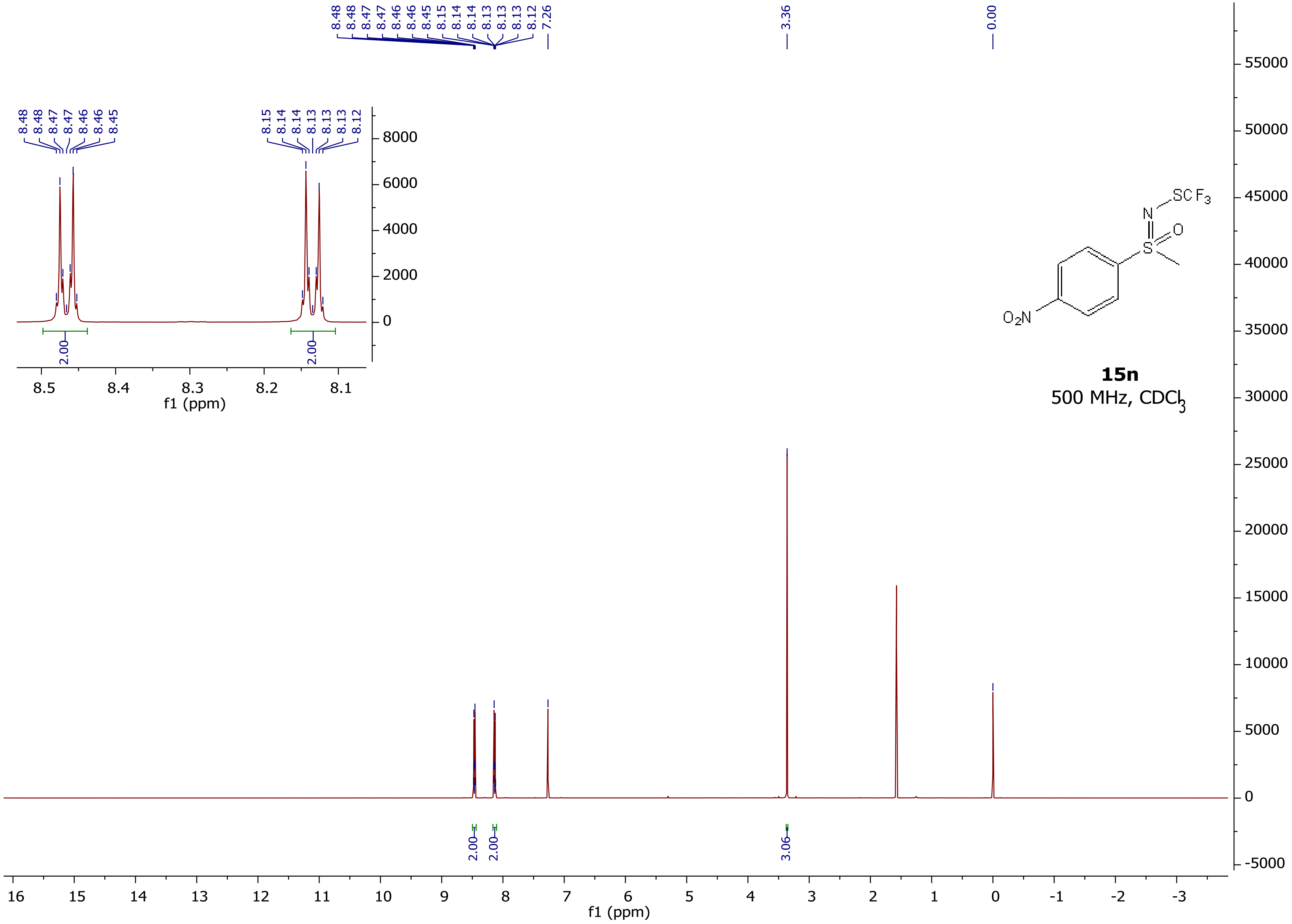

15n
500 MHz, $CDCl_3$

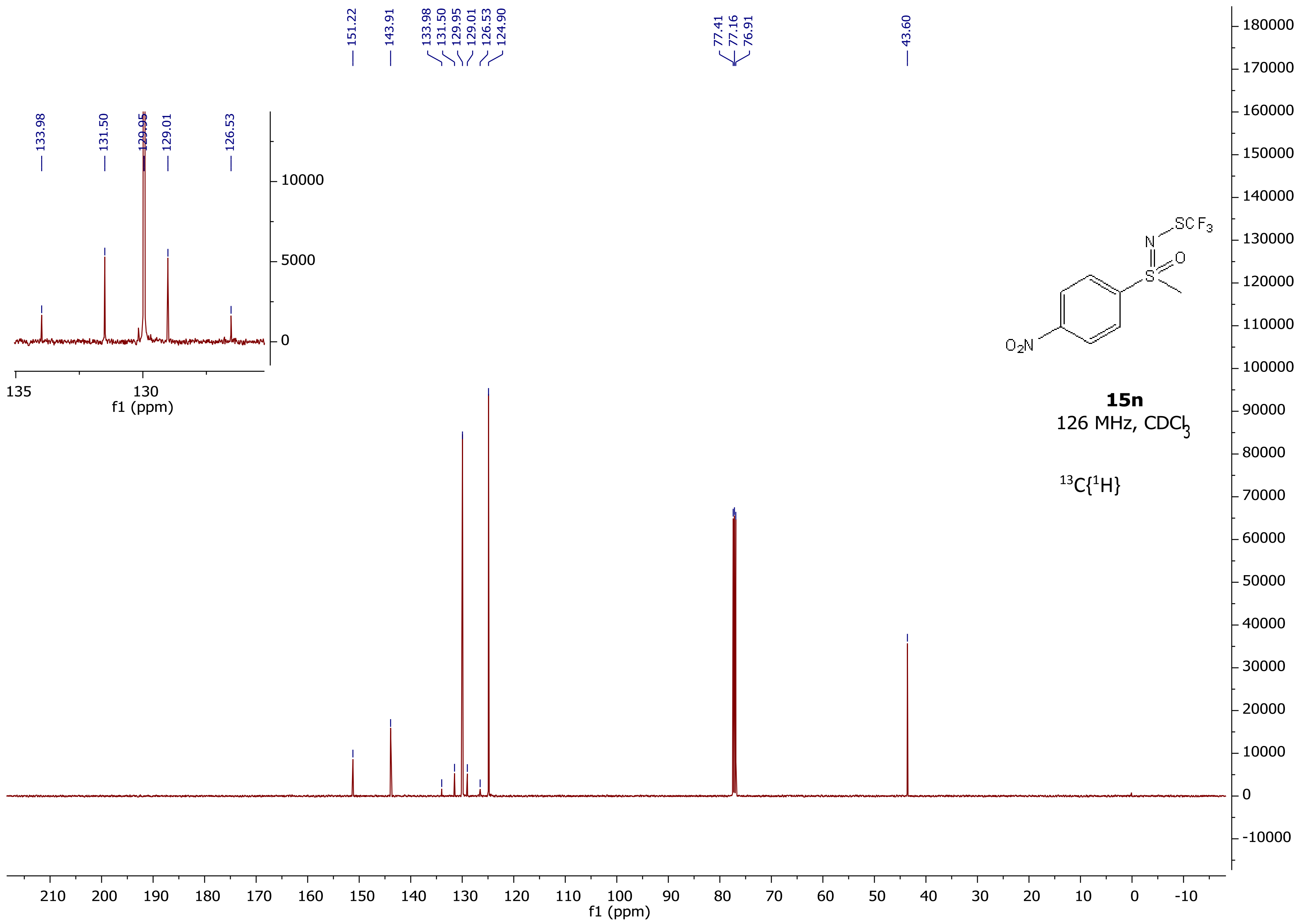

151.22
143.91
133.98
131.50
129.95
129.01
126.53
124.90
77.41
77.16
76.91
43.60
15n
126 MHz, CDCl3
13C{1H}
f1 (ppm)

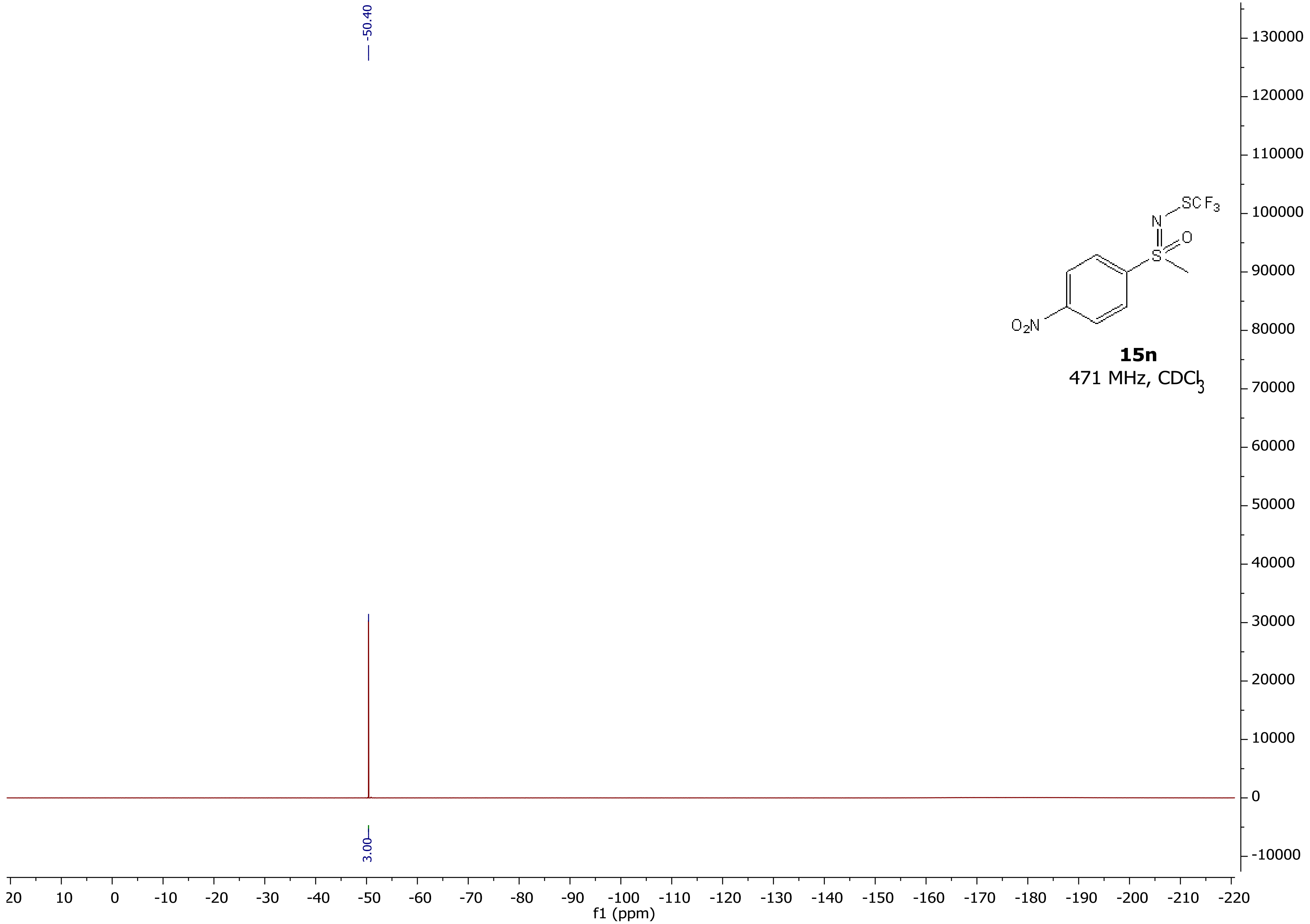
-50.40
SCF3
N
O
S
O2N
15n
471 MHz, CDCl3
3.00
f1 (ppm)

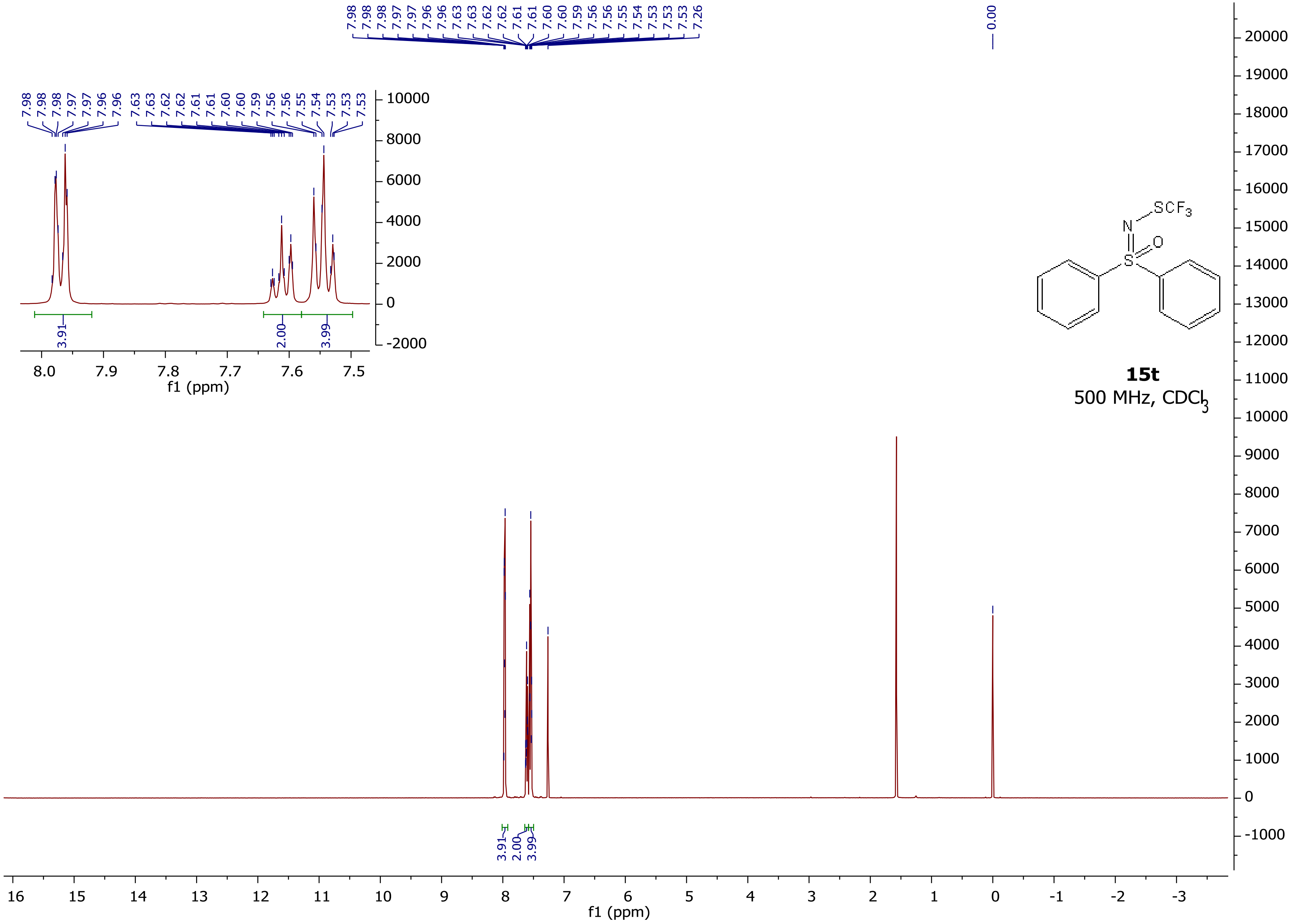
15t
500 MHz, CDCl3
f1 (ppm)

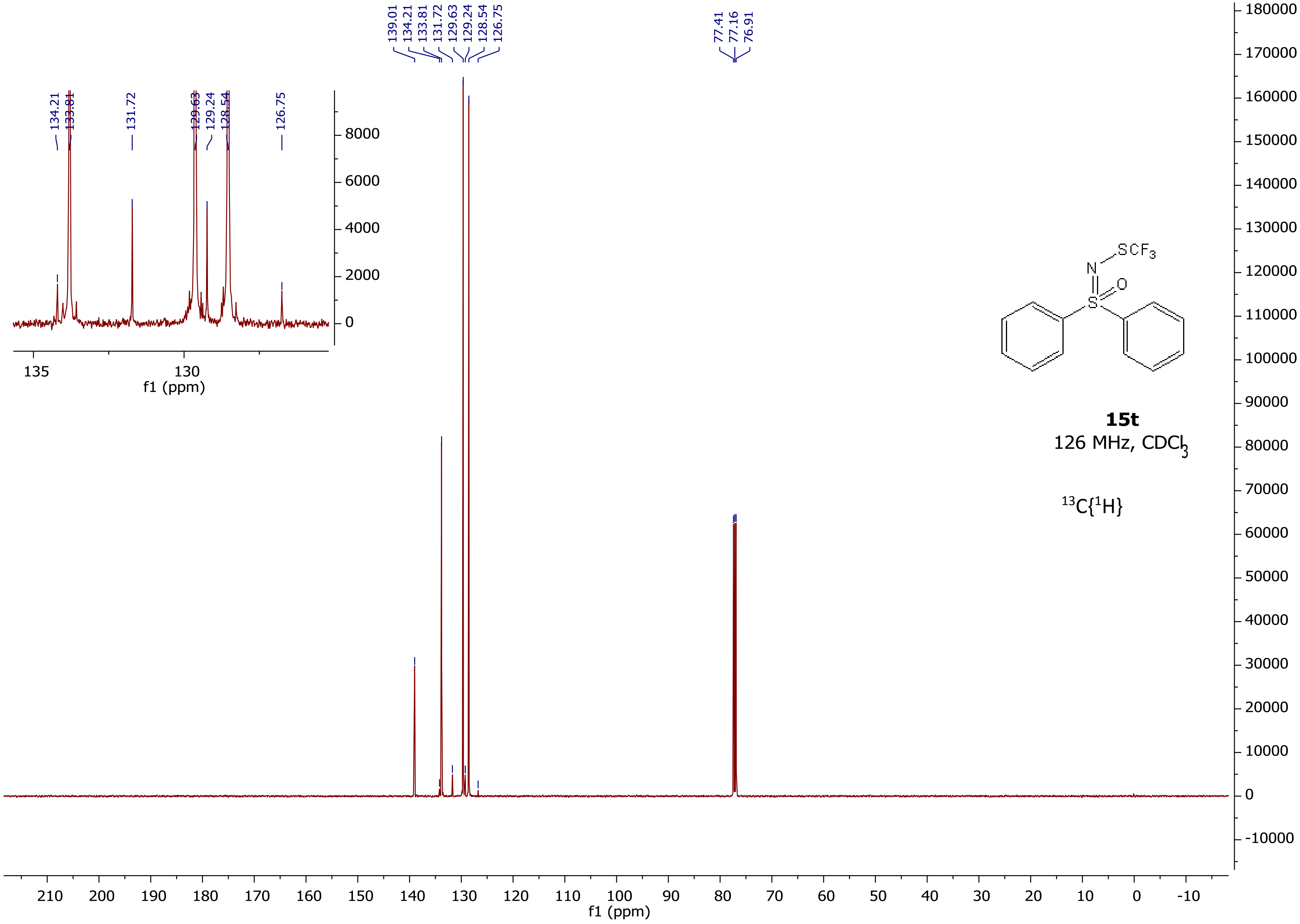
139.01
134.21
133.81
131.72
129.63
129.24
128.54
126.75
77.41
77.16
76.91
134.21
133.81
131.72
129.63
129.24
128.54
126.75
f1 (ppm)
f1 (ppm)
SCF3
N
S
O
15t
126 MHz, CDCl3
13C{1H}

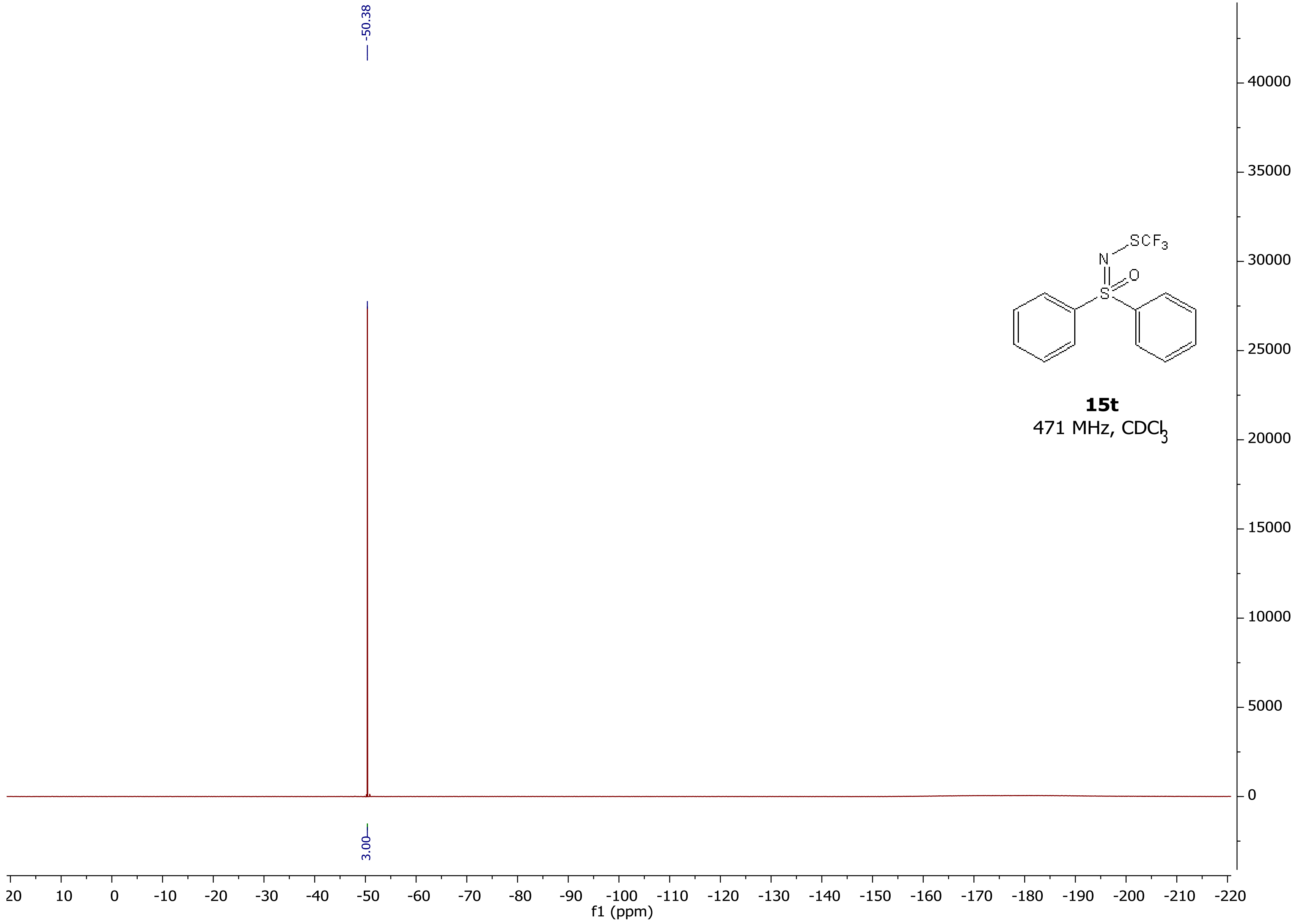
-50.38
SCF3
N
O
S
15t
471 MHz, CDCl3
3.00
f1 (ppm)

^{1}H NMR spectra of **2a** prepared by scale-up procedures

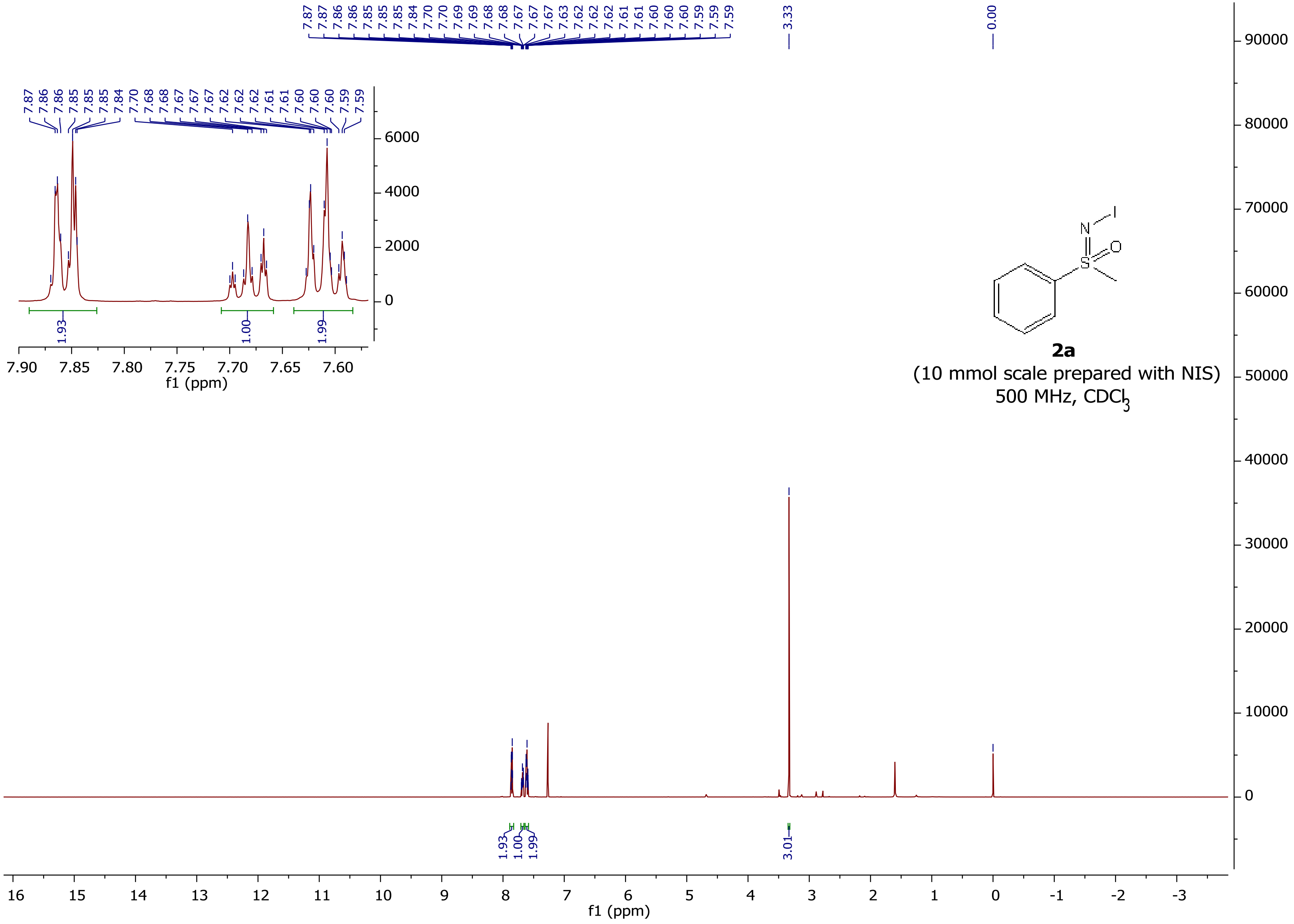

2a
(10 mmol scale prepared with NIS)
500 MHz, $CDCl_3$

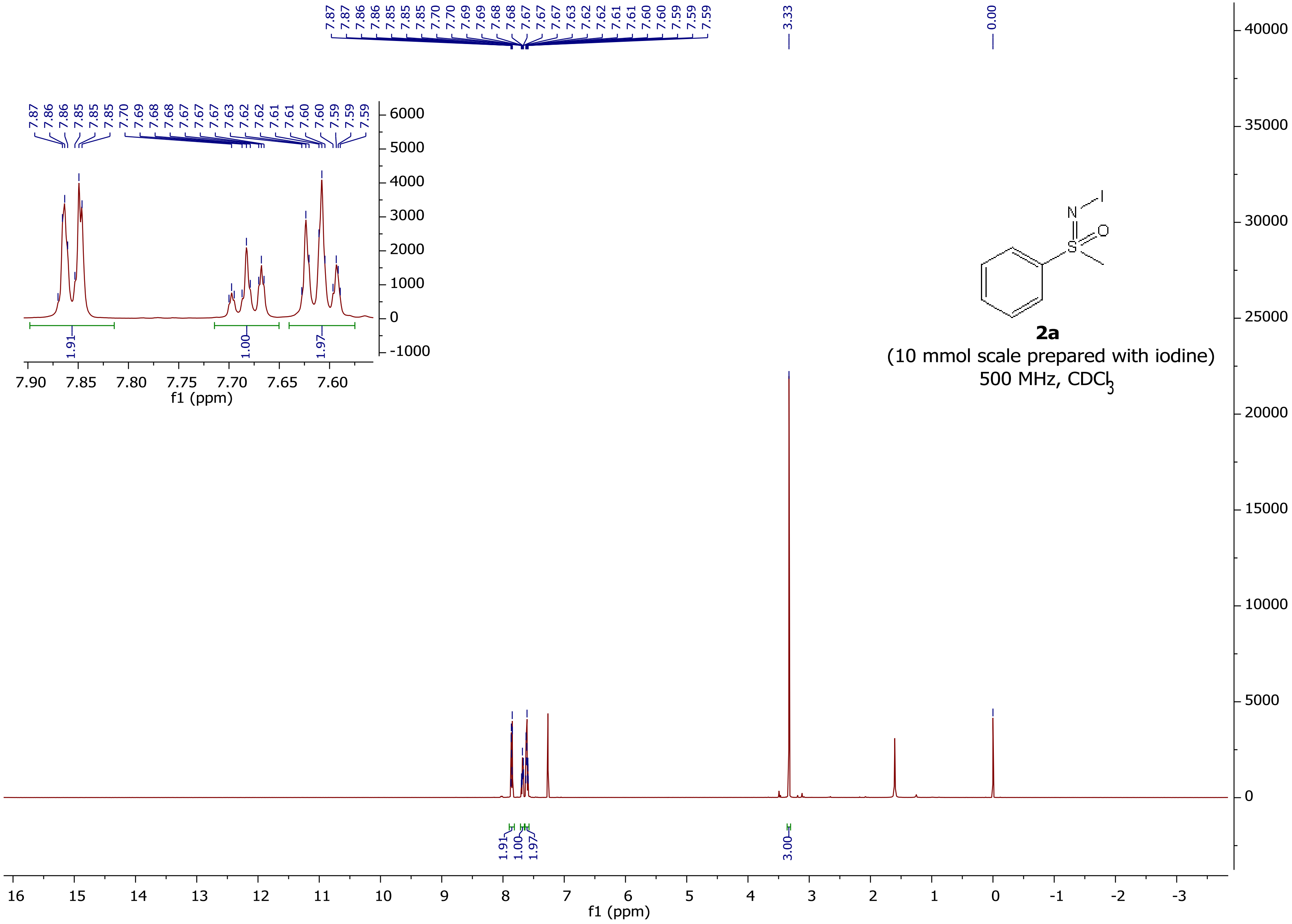

2a
(10 mmol scale prepared with iodine)
500 MHz, CDCl3
f1 (ppm)

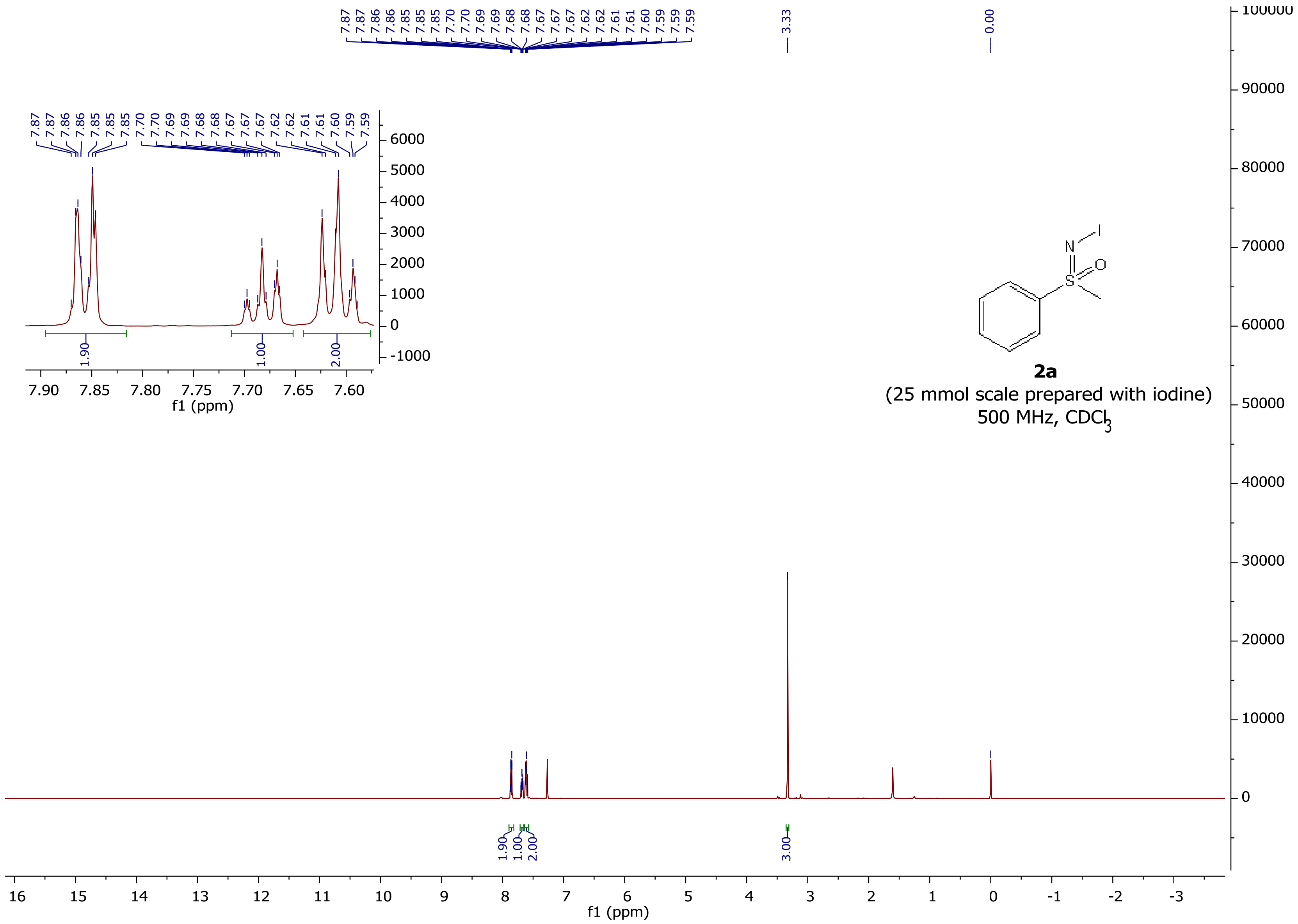
2a
(25 mmol scale prepared with iodine)
500 MHz, CDCl3
f1 (ppm)
7.87 7.87 7.86 7.86 7.85 7.85 7.85 7.70 7.70 7.69 7.69 7.68 7.68 7.67 7.67 7.67 7.62 7.62 7.61 7.61 7.60 7.59 7.59
3.33
0.00
1.90
1.00
2.00
3.00
